# Structure–Activity Relationship Studies of Chalcones and Diarylpentanoids with Antitumor Activity: Potency and Selectivity Optimization

**DOI:** 10.3390/ph16101354

**Published:** 2023-09-25

**Authors:** Joana Moreira, Joana B. Loureiro, Danilo Correia, Andreia Palmeira, Madalena M. Pinto, Lucília Saraiva, Honorina Cidade

**Affiliations:** 1 Laboratório de Química Orgânica e Farmacêutica, Departamento de Ciências Químicas, Faculdade de Farmácia, Universidade do Porto, Rua Jorge Viterbo Ferreira n° 228, 4050-313 Porto, Portugal; joana.m26@hotmail.com (J.M.); danilocorreia54@gmail.com (D.C.); apalmeira@ff.up.pt (A.P.); madalena@ff.up.pt (M.M.P.); 2 Centro Interdisciplinar de Investigação Marinha e Ambiental (CIIMAR), Universidade do Porto, Edifício do Terminal de Cruzeiros do Porto de Leixões, Av. General Norton de Matos s/n, 4050-208 Matosinhos, Portugal; 3 Laboratório Associado para a Química Verde (LAQV)/REQUIMTE, Laboratório de Microbiologia, Departamento de Ciências Biológicas, Faculdade de Farmácia, Universidade do Porto, Rua Jorge Viterbo Ferreira, 228, 4050-313 Porto, Portugal; up201407524@ff.up.pt

**Keywords:** chalcones, diarylpentanoids, antitumor activity, p53-MDM2, in silico studies

## Abstract

We previously reported that chalcone **CM-M345** (**1**) and diarylpentanoid **BP-C4** (**2**) induced p53-dependent growth inhibitory activity in human cancer cells. Herein, **CM-M345** (**1**) and **BP-C4** (**2**) analogues were designed and synthesized in order to obtain more potent and selective compounds. Compounds **16**, **17**, **19**, **20**, and **22**–**24** caused pronounced in vitro growth inhibitory activity in HCT116 cells (0.09 < GI_50_ < 3.10 μM). Chemical optimization of **CM-M345** (**1**) led to the identification of compound **36** with increased selectivity for HCT116 cells expressing wild-type p53 compared to its p53-null isogenic derivative and low toxicity to non-tumor HFF-1 cells. The molecular modification of **BP-C4** (**2**) resulted in the discovery of compound **16** with more pronounced antiproliferative activity and being selective for HCT116 cells with p53, as well as **17** with enhanced antiproliferative activity against HCT116 cells and low toxicity to non-tumor cells. Compound **16** behaved as an inhibitor of p53–MDM2 interaction, and compound **17** was shown to induce apoptosis, associated with an increase in cleaved PARP and decreased levels of the anti-apoptotic protein Bcl-2**.** In silico studies allowed us to predict the druglikeness and ADMET properties for **16** and **17**. Docking and molecular dynamics studies predicted that **16** could bind stably to the MDM2 binding pocket.

## 1. Introduction

One gene frequently mutated in human cancers is *TP53*, encoding the tumor suppressor protein p53, which is also known as the guardian of the genome [1]. The p53 protein is one of the most critically important tumor suppressors in human tumor biology. As a transcription factor, the p53 protein plays a key role in controlling cell proliferation, such as in the expression of various pro-apoptotic genes and genes involved in cell cycle arrest, senescence, and DNA repair. In fact, high levels of p53 stimulate the transcription of the gene encoding the cyclin-dependent kinase inhibitor (CKI) protein p21Waf1/Cip1 that binds to G1/S CDK and S CDK complexes, inhibiting their activity, thereby helping to block entry into the cell cycle [2]. Besides its involvement in the regulation of the cell cycle, p53 also plays a pivotal role in the induction of apoptosis, promoting the expression of proapoptotic factors, namely Bax and PUMA among others [3,4]. In this way, it prevents the accumulation of abnormal cells that can lead to the formation of tumors [5].

In normal cells that are not under stress, the p53 protein appears to be relatively unstable, with a short half-time [5]. p53 is often inactivated in cancers by different strategies, namely by interactions with murine double minute (MDM) proteins, MDM2 and MDMX, which are commonly overexpressed in cancers or by missense mutations, leading to loss of its wild-type (*wt*) tumor suppressor function [6]. Therefore, in the last few years, the search for small molecules able to reactivate the tumor suppressor function of p53, specifically through inhibition of the interaction between p53 and MDM2, has received great attention as a promising anticancer strategy [7]. Despite the effectiveness of several classes of p53-MDM2 interaction inhibitors, the displayed toxicity and the development of resistance have restricted their clinical use [8].

Chalcones are natural products with the chemical scaffold of 1,3-diphenyl-2-propen-1-one in common. These natural products represent one of the major classes of flavonoids that possess a wide range of biological activities, including anti-inflammatory, antioxidant, antidiabetic, antimicrobial, neuroprotective, and antitumor activities, among others [9,10,11,12,13,14,15,16]. Among these biological activities, the in vitro antiproliferative activity against cancer cell lines has been mostly reported, with it being associated with interfering with the activity of several mechanisms and targets related to carcinogenesis, namely aromatase, 17-β-hydroxysteroid dehydrogenase, proteasome, VEGF, MMP-2/9, JAK/STAT signaling pathways, CDC25B, microtubule (tubulin), cathepsin-K, topoisomerase-II, p53, and Wnt, among others [15,17,18]. Diarylpentanoids are chalcone analogues with two aromatic rings connected by a five-carbon bridge. These compounds are also known for having several biological activities [19], with their antitumor activity being one of the most studied [20]. It has been demonstrated that diarylpentanoids interfere with the p53 pathway [18] and inhibit p53-MDM2/X interaction [21]. Despite the promising antitumor activity that chalcones and diarylpentanoids exert, their low selectivity for cancer cells is a crucial limitation for the development of new anticancer drug candidates based on these scaffolds.

Following our goal of discovering new chalcone derivatives and their analogues with antitumor activity, we have identified several small molecules that display antiproliferative activity against a panel of cancer cells [21,22]. Among them, chalcone **CM-M345** (**1**) and diarylpentanoid **BP-C4** (**2**) revealed potent growth inhibitory activity against human cancer cells (2.1 ≤ GI50 ≤ 3.4 μM and GI50 = 6.25 μM, respectively) through potential activation of p53 by disrupting its interaction with MDM2 and MDM2/X, respectively [21,23]. Although the compounds **CM-M345** (**1**) and **BP-C4** (**2**) showed high antiproliferative activity, their selectivity for cancer cells was low. These studies support the idea that chalcone **CM-M345** (**1**) and **BP-C4** (**2**) represent a starting basis for the design of new promising compounds with improved antiproliferative properties through interference with the p53 pathway.

**Figure 1:**
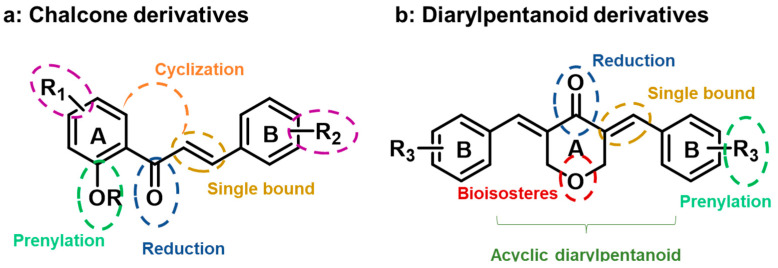
Design of **CM-M345** (**1**) (**a**) and **BP-C4** (**2**) (**b**) analogues.

The crucial need for novel antitumor agents with high selectivity toward cancer cells has prompted us to explore molecular modifications of chalcone **CM-M345** (**1**) and diarylpentanoid **BP-C4** (**2**) to perform structure–activity relationship (SAR) studies to obtain new, more selective and potent antiproliferative compounds. To fulfill this, different analogues of **CM-M345** (**1**) and **BP-C4** (**2**) were planned to use distinct strategies (Figure 1). Hence, small libraries of chalcone and diarylpentanoid derivatives were synthesized and evaluated for their antiproliferative activity on colorectal cancer HCT116 cells expressing p53 as well as in human normal fibroblasts HFF-1 cells to assess their selectivity toward cancer cells. To verify if the antiproliferative activity of **CM-M345** (**1**) and **BP-C4** (**2**) was p53-dependent, the growth inhibitory activity of all synthesized compounds was also tested in p53-null isogenic derivative of HCT116, in which p53 was knocked out (HCT116 p53^−/−^). The p53-MDM2 inhibitory effect of the compound showing the highest selectivity for HCT116 p53^+/+^ was further tested using yeast-based assays. To gain further insights into the mechanism of action, studies of its effect on cell cycle progression and apoptosis induction were also carried out. In silico studies were performed to predict the druglikeness and ADMET profile. Computational docking studies were also carried out in order to predict docking poses and residues involved in the p53–MDM2 potential interaction for the compound identified as a potential p53–MDM2 interaction inhibitor.

## 2. Results and Discussion

### 2.1. Synthesis

**Figure 2:**
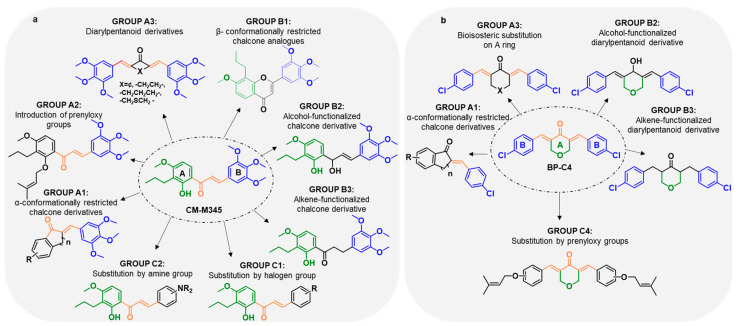
Different groups of synthetized compounds. (**a**) Groups of chalcone **CM-M345** (**1**) analogues; (**b**) groups of diarylpentanoid **BP-C4** (**2**) analogues.

To perform the SAR studies, several analogues of chalcone **CM-M345** (**1**, Figure 2a) and diarylpentanoid **BP-C4** (**2**, Figure 2b) with modifications on the aromatic rings or on the linker connecting these rings were synthesized and classified into three groups: A, B, and C as shown in Figure 2a,b. **CM-M345** (**1**) analogues with a similar 3,4,5-trimethoxyphenyl B group (Group A) were subdivided into three subgroups, namely those with a α-conformationally restricted A ring (Group A1), a prenylated A ring (Group A2), and the corresponding diarylpentanoids (Group A3) (Figure 2a). With these structural modifications, we intend to check the effect of molecular rigidification and extension by prenylation, as well as the substitution of the three atoms linker by a five atoms bridge with acyclic and cyclic moieties in the biological activity of **CM-M345** (**1**). Moreover, the synthesis of Group B included derivatives with modifications at the linker, namely β-conformationally restricted chalcone analogues (flavones, Group B1), alcohol-functionalized chalcone derivatives (Group B2), and alkene-functionalized chalcone derivatives (dihydrochalcone, Group B3) to evaluate the impact of molecular rigidification of the linker and the enone moiety in the antiproliferative activity. Group C comprised derivatives with different substitution patterns at the B aromatic ring, including electron-withdrawing (halogen, Group C1) and electron-donating (amine, Group C2) groups. Similar molecular modifications approaches were planned for the **BP-C4** (**2**) derivatives (Figure 2b). However, the molecular modifications were adapted to the diarylpentanoid family and the already published SAR was taken into consideration [21]. Thus, Group A of **BP-C4** (**2**) derivatives included subgroup A1, comprising chalcones with an α-conformationally restricted A ring and a new group, Group A3 consisted of **BP-C4** (**2**) analogues planned by substitution of the cyclic C5 bridge of **BP-C4** (**2**) by 3-oxopenta-1,4-diene moiety, or by other cyclic C5 bridges, namely with tetrahydro-4*H*-thiopyran-4-one, cyclohexanone, and cyclopentanone moieties. Alcohol-functionalized (Group B2) and alkene-functionalized (Group B3) diarylpentanoids were included in Group B. Groups C1 and C2 were excluded taking into account the results previously published by our group [21]. Group C4 consisted of prenylated analogues of **BP-C4** (**2**).

#### 2.1.1. Synthesis of Compounds of Group A

**Scheme 1:**
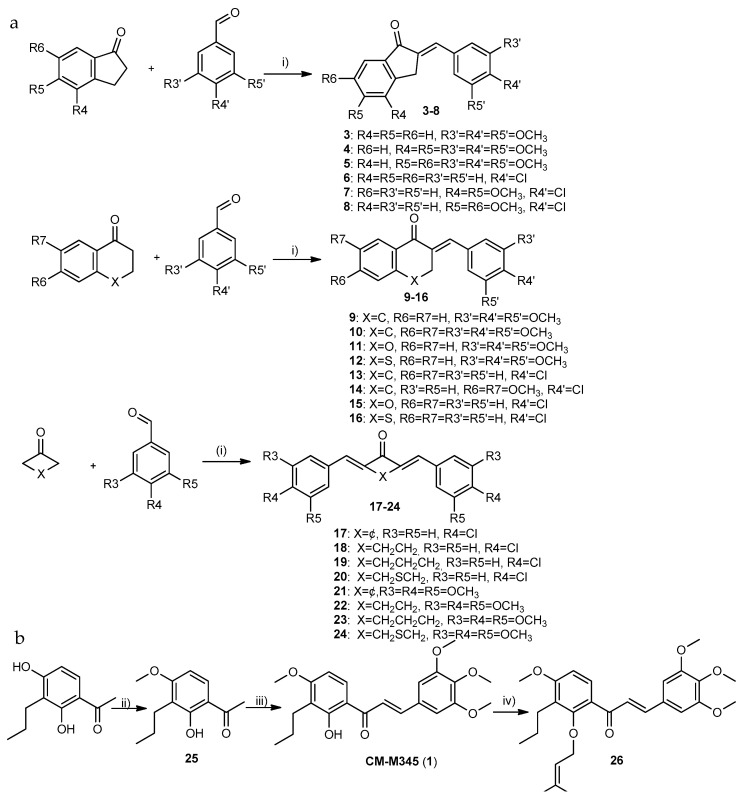
Synthesis of compounds of group A. (**a**) Synthesis of compounds of group A1 and A3; (**b**) Synthesis of compounds of group A2. Reaction conditions: (i) NaOH, MeOH, reflux; (ii) (CH_3_)_2_SO_4_, K_2_CO_3_, acetone, reflux; (iii) 3,4,5-trimethoxybenzaldehyde, NaOH, MeOH, reflux; and (iv) TBAOH.H_2_O, CHCl_3_/toluene (10:7), prenyl bromide, rt.

Derivatives of group A1 (**3**–**16**, Scheme 1a) and A3 (**17**–**23**, Scheme 1a) were obtained by the Claisen–Schmidt condensation of ketones with 3,4,5-trimetoxy- or 4-chlorobenzaldehyde as building blocks with yields ranging from 23% to 90%. The synthesis of compound **26** (group A2) was accomplished in a three-step process (Scheme 1b). Firstly, the methylation of 1-(2,4-dihydroxy-3-propylphenyl)ethan-1-one with dimethyl sulfate in the presence of anhydrous potassium carbonate and anhydrous acetone gave rise to 1-(2-hydroxy-4-methoxy-3-propylphenyl)ethan-1-one (**25**) (Scheme 1b). Then, chalcone **CM-M345** (**1**) was prepared by the base-catalyzed aldol reaction of 1-(2-hydroxy-4-methoxy-3propylphenyl)ethan-1-one (**25**) with 3,4,5-trimethoxybenzaldehyde as building blocks, as previously reported [23]. The prenylation of this chalcone (**CM-M345** (**1**)) was carried out by the reaction with prenyl bromide in the presence of tetrabutylammonium hydroxide at room temperature giving rise to **26** with 66% yield (Scheme 1b).

#### 2.1.2. Synthesis of Compounds of Group B

**Scheme 2:**
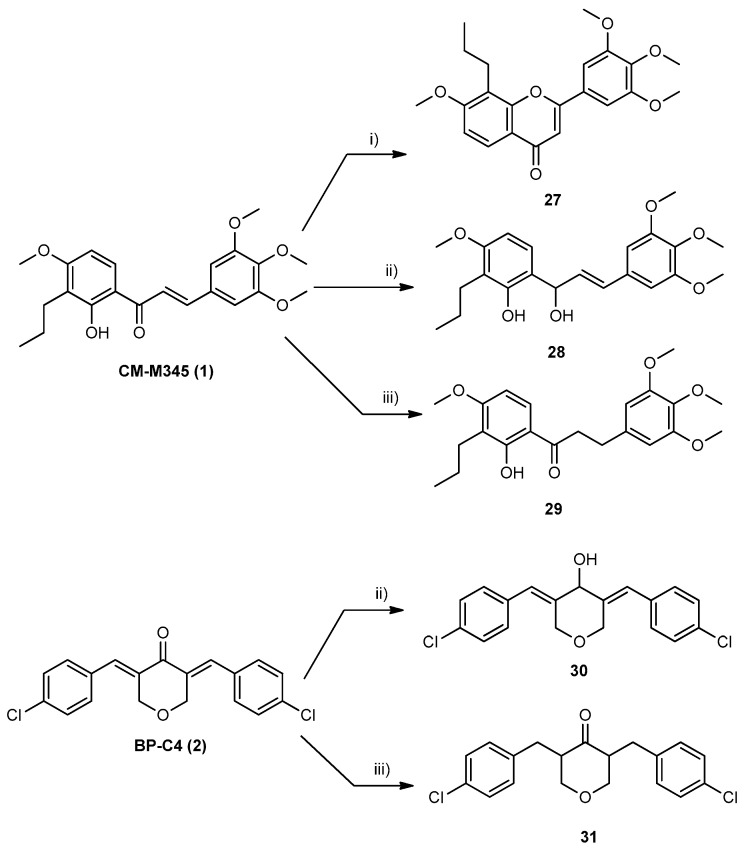
Synthesis of compounds of group B. Reaction conditions: (i) I_2_ (cat.), DMSO, reflux, N_2_; (ii) NaBH_4_, MeOH, 30 °C; and (iii) Pd/C, H_2_, toluene, rt.

Flavone derivative **27** was synthesized from chalcone **CM-M345** (**1**, Scheme 2). Cyclization of chalcone **CM-M345** (**1**) into corresponding flavone **27** was carried out using DMSO/I_2_ as a catalyst with 60% yield. Additionally, we modified the enone moiety (in **CM-M345** (**1**) and **BP-C4** (**2**)) by selectively reducing either the carbonyl group, or the double bonds. In the literature, several methods have been reported for the reduction of the α,β-unsaturated system, but the same is not true for selective reduction. To the best of our knowledge, sodium borohydride is used as a selective reducing agent for reducing carbonyl to the hydroxyl group of chalcone derivatives without affecting the -ene part of enone [24], and catalytic hydrogenation is one of the most reported for the selective reduction of the double bond of enone moiety [25]. Thus, alcohol-functionalized chalcone **28** and alcohol-functionalized diarylpentanoid **30** were obtained from the reduction of the carbonyl ketone system of chalcone or diarylpentanoid, respectively, by NaBH_4_ and methanol as the solvent (Scheme 2), according to the procedure reported by Aramini et al. [24]. The reduction of the carbonyl group resulted in the disappearance of the characteristic yellow color of the precursor and the structure of **28** and **30** was confirmed by the appearance of a H-1 proton, obtained by the reduction of the carbonyl group, at δ 5.01 and 4.91 ppm, respectively (^1^H NMR).

To obtain the alkene-functionalized derivatives, we needed to reduce the carbonyl group in **CM-M345** (**1**) and **BP-C4** (**2**) to methylene units. Thus, the selective reduction of the double bond of α,β-unsaturated ketone system was accomplished by catalytic hydrogenation in a H_2_ atmosphere with 10% Pd/C as the catalyst and toluene as the solvent to obtain dihydrochalcone **29** and diarylpentanoid **31** (Scheme 2). Once again, the reduction of the double bonds resulted in the disappearance of the characteristic yellow color of the precursor. The confirmation of the success of double bond reduction was accomplished by NMR. For compound **29**, the characteristic signals of benzylic protons (δ_H_ 3.00 *t*, *J* = 7.1 Hz, H-2‴) and protons at α position of the carbonyl group (δ_H_ 3.24 *t*, *J* = 7.5 Hz, H-1‴) are observed. For compound **31**, instead of signals of an olefinic proton detected in the ^1^H (δ_H_ 7.77 *sl*, H-1″) NMR spectrum of **BP-C4** (**2**) used as the precursor, the characteristic signals of two benzylic protons (δ_H_ 3.17 *q* (*J* = 7.2 Hz) and 2.36 *q* (*J* = 7.2 Hz), H-1″a and H-1″b) and one proton at α position of the carbonyl group (δ_H_ 2.94–2.83 *m*, H-2, −6) are observed.

#### 2.1.3. Synthesis of Compounds of Group C

**Scheme 3:**
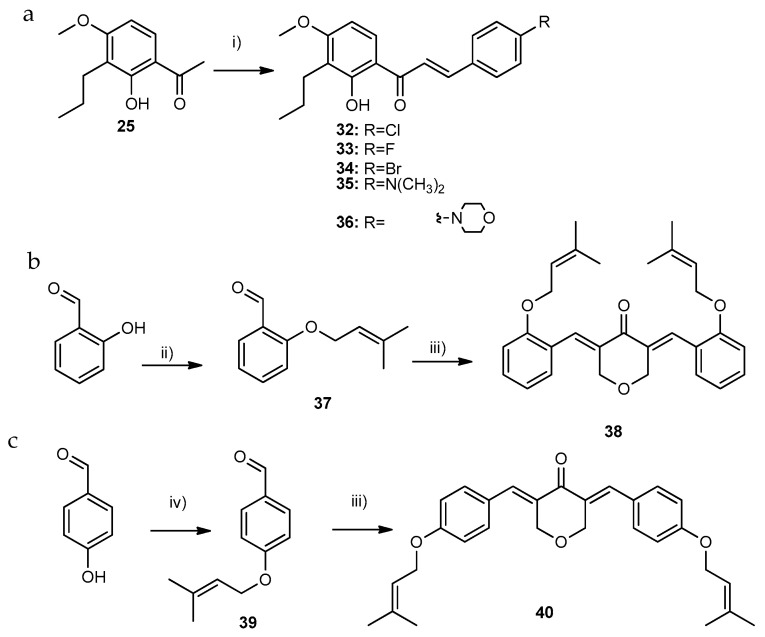
Synthesis of group C compounds. (**a**) Synthesis of compounds of group C1 and C2; (**b**,**c**) Synthesis of compounds of group C4. Reaction conditions: (i) substituted benzaldehyde, NaOH, MeOH, reflux; (ii) TBAOH.H_2_0, CHCl_3_/toluene (10:7), prenyl bromide, rt; (iii) tetrahydro-4*H*-pyran-4-one, NaOH, MeOH, reflux; and (iv) anhydrous acetone, prenyl bromide, anhydrous K_2_CO_3_, reflux.

A total of five compounds containing different substitutions on the two aromatics rings, namely halogen (**32**–**34**, group C1) or amine (**35** and **36**, group C2) groups, were synthesized by base catalyzed aldol reactions of 1-(2-hydroxy-4-methoxy-3-propyl phenyl)ethan-1-one (**25**, Scheme 3a). Prenylated derivatives of **BP-C4** (**2**) were synthesized according to the strategy illustrated on Scheme 3b,c. Firstly, 2- or 4-hydroxybenzaldehyde were prenylated by the reaction with prenyl bromide in the presence of tetrabutylammonium hydroxide at room temperature (Scheme 3b) or K_2_CO_3_ at reflux (Scheme 3c), respectively. Afterwards, compounds **38** and **40** were prepared by aldol condensation with appropriately substituted benzaldehyde and tretahydro-4*H*-pyran-4-one.

The structure elucidation of compounds **3**, **5**, **6**, **8**, **9**, **11**, **13**, **15**–**24**, **28**, and **32** was established by ^1^H and ^13^C nuclear magnetic resonance (NMR) techniques (Figures S1, S3, S4, S6, S7, S9, S11, S13–S22, S25, and S29—Supporting Information), and the data were in accordance with the previously reported data [23,26,27,28,29,30,31,32,33,34,35,36,37,38,39,40]. The structure elucidation of new compounds **4**, **7**, **10**, **12**, **14**, **26**, **27**, **30**, 3**1**, **33**–**36**, **38**, and **40** was established on basis of NMR (Figures S2, S5, S8, S10, S12, S23, S24, S26–S28, and S30–S35—Supporting Information) and high-resolution mass spectrometry (HRMS) (Figures S36–S47—Supporting Information). ^13^C NMR assignments were determined by 2D heteronuclear single quantum correlation (HSQC) and heteronuclear multiple bond correlation (HMBC) experiments.

### 2.2. Biological Activity Evaluation

#### 2.2.1. Growth Inhibitory Activity of **3**–**40** in Human Cancer Cells

To study the in vitro antitumor potential of chalcone **CM-M345** (**1**) and diarylpentanoid **BP-C4** (**2**) analogues, the sulforhodamine B (SRB) assay was used to evaluate the antiproliferative activity in human colorectal HCT116 cancer cells. To assess their cytotoxicity toward normal cells, the antiproliferative effect of these compounds was also tested in human fibroblasts HFF-1 cells. In order to confirm if inhibition of proliferation was associated with p53 activation in HCT116 p53^+/+^ cells, all compounds were also tested in HCT116 cells in which p53 was knocked out (HCT116 p53^−/−^) and the selective index was calculated and compared to the compounds **CM-M345** (**1**) and **BP-C4** (**2**), for which p53-dependent antiproliferative activity was previously reported by our research group [21,23] (Table 1).

Most compounds displayed GI_50_ values lower than 10 μM in HCT116 p53^+/+^ cells, with three **BP-C4** (**2**) analogues (**18**–**20**) and four **CM-M345** (**1**) analogues (**16**, **17**, **19,** and **20**) presenting a GI_50_ value lower than the hit compounds previously reported: **CM-M345** (**1**) [23] and **BP-C4** (**2**) [21] (Table 1). Notably, diarylpentanoid **17**, possessing a chlorine group at the *para* position in both aromatic rings as **BP-C4** but a 3-oxo-penta-1,4-diene moiety instead of a chroman-4-one moiety in the linker connecting both aromatic rings, displayed a marked increase in selectivity for HCT116 cells when compared to non-tumorigenic HFF-1 cells (the GI_50_ value in HFF-1 cells was 12-fold higher than the HCT116 p53^+/+^ cells) (Table 1). Importantly, the obtained selectivity index (Table 1) showed a notable degree of selectivity of diarylpentanoid **17** for cancer cells, higher than chalcone **CM-M345** (**1**) and diarylpentanoid **BP-C4** (**2**), and the anticancer drug etoposide. Additionally, chalcones **16** and **36** were selective for p53^+/+^ HCT116 cells over cells with deleted p53, showing a selective index higher than **CM-M345** (**1**) and **BP-C4** (**2**) (Table 1). Notably, for **BP-C4** (**2**) analogues, chalcone **16**, which also possess a chlorophenyl group as **BP-C4**, showed nine-fold higher antiproliferative activity and seven-fold increased selectivity for p53, when compared to **BP-C4** (**2**). The overall results suggest that the presence of at least one 4-chlorophenyl group, as with compounds **16** and **17**, seems to be important to obtain compounds with selectivity for p53^+/+^ HCT116 cells over cells with deleted p53 and normal cells as HFF-1 cells.

**Figure 3:**
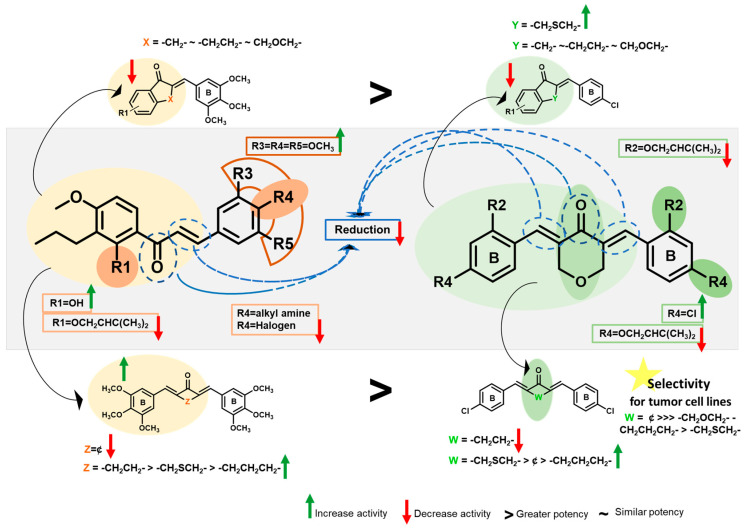
Putative SAR for synthesized chalcone and diarylpentanoid derivatives with antiproliferative activity in HCT116 p53^+/+^ cells.

For group A1, when comparing **CM-M345** (**1**, GI_50_ = 2.6 ± 0.2 µM) with α-conformationally restricted chalcones **3**–**5**, and **9**–**12** (6.3 > GI_50_ > 3.8 µM), a reduction in the growth inhibitory effect was obtained. For most of the **BP-C4** (**2**) analogues (**7**, **8**, **10**, and **11**; 8.6 > GI_50_ > 6.7 µM), this molecular modification resulted in comparable activity, except for **6** and **13**, with them showing no activity at the highest concentration tested (30 > GI_50_ > 21.5 µM) and for **16**, which showed pronounced enhancement of the activity (GI_50_ value of 0.69 µM) (Figure 3). The overall results suggested that the molecular rigidification involving C-α may not be favorable for increasing the antiproliferative activity of chalcone derivatives with a 3,4,5-trimethoxyphenyl group as B ring but is an interesting approach to obtain potent compounds for α-conformationally restricted chalcones with chlorine at C-4 with a thiochromone moiety. Comparing the effects of α-conformationally restricted chalcones with the same scaffold and different B aromatic rings, it seems that the presence of a 3,4,5-trimethoxyphenyl group (**3**–**5**, and **9**–**11**) is more favorable than a 4-chlorophenyl group (**6**–**8**, and **13**–**15**), except for compounds with a thiochroman-4-one moiety (**12**: GI_50_ = 5.7 µM vs **16**: GI_50_ = 0.69 µM) (Figure 3). Additionally, the substitution of the 2′-hydroxyl group by a prenyloxy group at the A ring in group A2 (compound **26**) led to a reduction in activity compared to **CM-M345** (**1**) (Figure 3). Interestingly, these results are not in accordance with what was expected considering that we have previously demonstrated that *O*-prenylation of 2′-hydroxy-3,4,4′,5,6′-pentamethoxychalcone resulted in a marked increase in antiproliferative activity [41,42].

For group A3, comparing the effects of **CM-M345** (**1**) and diarylpentanoids with 3,4,5-trimethoxyphenyl groups (**21**–**24**), it seems that the presence of a C5 bridge with a cyclic moiety (**22**–**24**) is also favorable for activity, but the presence of a C5 bridge with an acyclic moiety (**21**) is associated with the loss of activity. Interestingly, comparing the effects of diarylpentanoids with the same C5 bridges and different phenyl groups, once again it seems that the presence of 3,4,5-trimethoxyphenyl groups (**22**–**24**) is much more favorable than 4-chlorophenyl groups (**18**–**20**), except for diarylpentanoid with a penta-1,4-dien-3-one C5 bridge (**21**: GI_50_ > 30 µM vs **17**: GI_50_ = 2.18 µM) (Figure 3). In addition, comparing the effects of diarylpentanoids with the same phenyl groups and different C5 bridges, it seems that the presence of a tetrahydro-4*H*-pyran-4-one moiety (**BP-M345** (**1**): GI_50_ = 0.17 µM; results previously reported by our group [21]) is associated with higher activity than those with a cyclohexanone moiety (**23**: GI_50_ = 2.3 µM) but similar activity to those with a tetrahydro-4*H*-thiopyran-4-one moiety (**24**: GI_50_ = 0.31 µM) (Figure 3). Moreover, the diarylpentanoid with a cyclopentanone moiety (**22**: GI_50_ = 0.09 µM) seems to be the most active, and the presence of a penta-1,4-dien-3-one moiety dramatic decreases the antiproliferative activity (**21**: GI_50_ > 30 µM). For diarylpentanoids with 4-chlorophenyl groups, it was observed that the compounds with a tetrahydro-4*H*-thiopyran-4-one moiety (**20**: GI_50_ = 1.13 µM) exhibited the best activity, and the presence of a C5 bridge with a cyclopentanone moiety leads to the loss of antiproliferative activity (**18**: GI_50_ > 30 µM) (Figure 3). Interestingly, only for this group were compounds obtained with selectivity for cancer cells (**17**: SI2 = 12.43, and **19**: SI2 = 4.55, Table 1), with it being evident that the presence of a C5 bridge with an acyclic moiety, penta-1,4-dien-3-one, and 4-chlorophenyl groups as the B ring is the most favorable for selectivity of the compounds for the HCT116 cancer cell line.

In group B1, the synthesis of flavone **27** (GI_50_ > 30 µM) led to the loss of antiproliferative activity compared to chalcone **CM-M345** (**1**, GI_50_ > 2.6 µM). In groups B2 and B3, compounds **28**–**31**, resulting from the selective reduction of the enone moiety, showed significantly lower antiproliferative activity than **CM-M345** (**1**) and **BP-C4** (**2**) (Figure 3), suggesting that unsaturation is important for activity. Our results are consistent with previous reports showing that this structural feature is important for antiproliferative activity.

For group C1, the presence of halogen groups at the *para*-position (**32**–**34**) is associated with a decrease in the growth inhibitory effect (18.51 > GI_50_ > 9.25 µM) compared with the hit compound **CM-M345** (**1**, GI_50_ = 2.6 µM) (Figure 3). This reduction in antiproliferative effect is also observed for *para*-aminated chalcone derivatives of group C2 (**35** and **36**; 10.7 > GI_50_ > 5.1 µM) (Figure 3). The reduction in the antiproliferative activity caused by this molecular modification was in accordance with the previously reported for diarylpentanoids by our research group [21]. Concerning compounds of group C3, as for the prenylation of **CM-M345** (**1**), the substitution of chlorine by prenyloxy groups at both aromatic rings (compounds **38** and **40**) was unfavorable for activity, with it being associated with a reduction in (**40**: GI_50_ = 9.51 µM) or the loss (**38**: GI_50_ > 30 µM) of activity compared with hit compound **BP-C4** (**2**, Figure 3).

#### 2.2.2. Effect of Compounds **16** and **36** on the p53–MDM2 Interaction Analyzed Using Yeast-Based Assays

**Figure 4:**
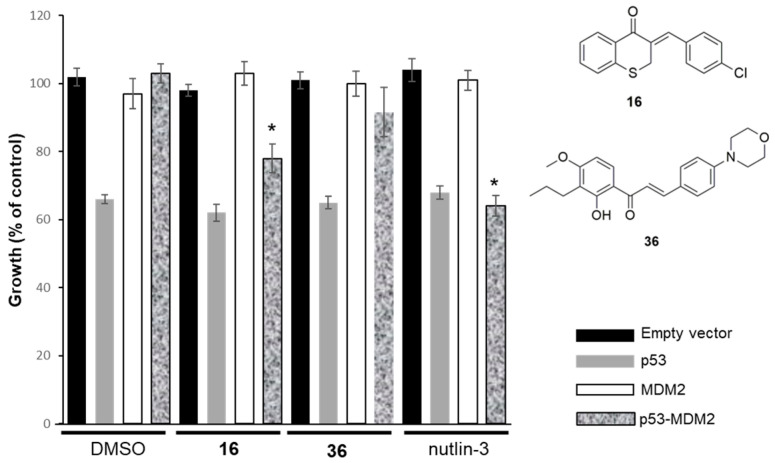
Effects of 10 µM of **16** and **36** and 10 µM of nutlin-3 on the growth of the control yeast (empty vectors), yeast expressing only p53, yeast expressing only MDM2, and yeast co-expressing p53 and MDM2. The results are plotted by setting as 100% growth the number of CFUs obtained with untreated control yeast. The data are the mean S.E.M. of four–five independent experiments; values obtained with yeast co-expressing p53 and MDM2 treated with compound significantly different from DMSO (* *p* < 0.05).

As compounds **16** and **36** showed selectivity for p53^+/+^ HCT116 cells over cells with deleted p53 and low cytotoxicity in HFF-1 cells, its mode of action was investigated. The possible activation of p53 by inhibiting its interaction with MDM2 was investigated using the yeast-based screening assay previously developed by our group [43]. In this assay, compounds that inhibit p53–MDM2 interaction will re-establish the p53-induced growth-inhibitory effect, which was abolished by MDM2 [43]. The results obtained showed that only compound **16** behaved as a potential inhibitor of the p53-MDM2 interaction, at 10 μM, without interfering with the growth of the control yeast (vectors) or yeast cells expressing p53 only (Figure 4). Contrarily, the activity of **36** seems not to be related to the inhibition of p53–MDM2 interaction.

#### 2.2.3. Study of the Mechanism of Action of Compound **17** in Human Cancer Cells

**Figure 5:**
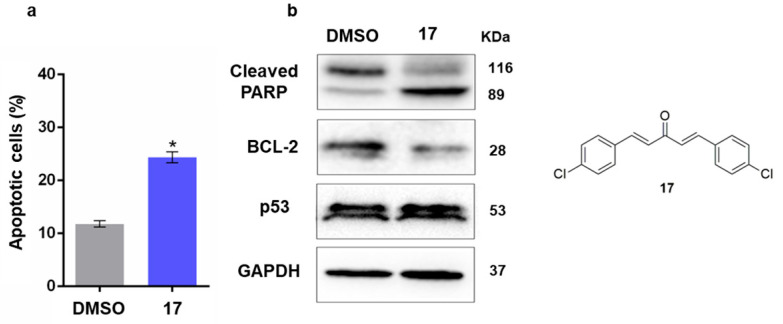
Compound **17** induces a p53-independent apoptotic cell death in colorectal cancer cells. (**a**) Effect of 5 µM **17** on apoptosis after 48 h treatment; the percentage of annexin-positive cells was analyzed by flow cytometry; the data are the mean ± SEM of three independent experiments. The values are significantly different from DMSO (* *p* < 0.05, unpaired Student’s *t*-test). (**b**) Effect of 5 µM **17**, after 48 h of treatment, on the protein expression of p53, PARP, Bcl-2 and p53. The immunoblots are representative of three independent experiments. GAPDH was used as a loading control.

Considering that compound **17** displayed selectivity for HCT116 p53^+/+^ cells when compared to non-tumorigenic HFF-1 cells, the underlying molecular mechanism of action was further investigated. At 5 µM, **17** induced apoptosis, as demonstrated by the increase in annexin-positive cells (Figure 5a), through activation of a caspase pathway, as indicated by the PARP cleavage, and also involving mitochondria, as evidenced by the depletion of the protein levels of the anti-apoptotic protein Bcl-2 (Figure 5b), in HCT116 p53^+/+^ cells. In accordance with the no significant differences in the GI_50_ values of **17** in p53^+/+^ and p53^−/−^ HCT116 cells, **17** did not interfere with the p53 protein levels in HCT116 p53^+/+^ cells (Figure 5b), demonstrating a p53-independent cytotoxic effect by **17**.

Altogether, these results indicated that compound **17** triggered caspase- and mitochondrial-mediated apoptosis not dependent on p53.

### 2.3. In Silico Studies

#### 2.3.1. Predicting Druglikeness

In silico studies were performed in order to predict the druglikeness properties of the most promising compounds (**16** and **17**). The druglikeness was evaluated using the SwissADME web server (SwissADME, http://www.swissadme.ch/ (accessed on 1 January 2023)) according to the following chemical features: (i) unsaturation, inferred by the fraction of carbon sp^3^ (Fsp3); (ii) flexibility, inferred by the number of rotatable bonds (RB); (iii) polarity, inferred by polar surface area (PSA); (iv) lipophilicity, inferred by the log P; and (v) solubility, inferred by log S. For each compound, the above-mentioned molecular descriptors/physicochemical properties were calculated (Table 2). The obtained mean values of each chemical feature were compared with the range of values preconized by the druglikeness guidelines.

The MW of the compounds (286.78–303.18 g mol^−1^) were in the range of values preconized by the druglikeness guidelines (150 < MW < 500), slightly lower than the mean MW value of synthetic drugs (325.2 g mol^−1^) [44] as well as the mean MW value (313 g mol^−1^) reported for orally bioavailable cancer drugs (Table 2) [45]. The fraction of aromatic heavy atoms (Far = AHA/ HA) and the number of RBs and aromatic characters, estimated by unsaturation (Fsp^3^), are widely employed to infer molecular flexibility. The analysis of these molecular descriptors revealed that, while the number of RBs are in accordance with the values preconized by the druglikeness guidelines, the Fsp^3^ value did not (0 < RB < 9; 0.25 < Fps^3^ < 1). The only feature that did not fall within the preconized limits in druglike chemical space for compounds was unsaturation. Nevertheless, the high unsaturation degree is a characteristic of the diarylpentanoid scaffold and per se cannot invalidate this library. The number of HBAs, the number of HBDs, and PSAs all contribute to molecular polarity. Regarding these data, it was observed that HBA and PSA values for both compounds differed from the literature for synthetic drugs (mean HBA of 4.8; mean HBD of 1.6; 20 < PSA < 125 Å^2^) [44]. The log P and log S were used to predict lipophilicity and solubility, respectively (Table 2). Considering the druglikeness guidelines’ range of values (0 < Log P < 5; Log S > −4 [45]), compounds have a mean Log P value (4.28 < mean Log P value < 4.92) within the limits but considering that reported synthetic drugs have a mean of 2.4, **17** showed high mean log P values [44], and compounds might face problems of solubility (−5.75 < mean log P value < −5.45).

Also using SwissADME web server (SwissADME, http://www.swissadme.ch/ (accessed on 1 January 2023)), several druglikeness rules were evaluated, namely Ghose, Veber, Egan, and Muegge rules), as well as bioavailability score and synthetic accessibility (Table 3). Compounds **16** and **17** were also analyzed using the PreADMET web server (PreADME, https://preadmet.bmdrc.kr/ (accessed on 1 January 2023)) for featured prediction of other druglikeness properties, including CMC-like, Lead-like, WDI-like, and MDDR-like rules (Table 3). The acceptable value of violations for a druglike molecule is 1 and the synthetic accessibility range is from 1 (very easy) to 10 (very difficult).

#### 2.3.2. ADMET Properties Prediction

The ADMET properties of the compounds were calculated using the SwissADME web server (SwissADME, http://www.swissadme.ch/ (accessed on 1 January 2023 and 8 September 2023)) and the PreADMET web server (PreADMET, https://preadmet.bmdrc.kr/ (accessed on 1 January 2023)). The calculated ADMET properties of **16** and **17** included the parameters of oral absorption, namely gastrointestinal (GI) absorption (High = good absorption), the percentage of human oral absorption (%HIA > 70% = well absorbed), Caco-2 cell permeability in nm·s^−1^ (Caco-2 > 70 nm·s^−1^ = high permeability), parameters of distribution, namely blood–brain barrier (BBB) permeation (No = is not able to permeate the BBB), P-gp substrate (No = is not able to be subtract of P-gp), plasma protein binding (PPB > 90% = chemical strongly bound), parameters of metabolism, namely the ability to inhibit one of nine major isoforms of CYP450 (CYP1A2, CYP2C19, CYP2C9, CYP2D6, CYP3A4, CYP2C19, CYP2C9, CYP2D6, and CYP3A4; No = is not able to inhibit CYP450), parameters of toxicity, namely carcinogenic activity in a mouse or rat (Negative = clear evidence of carcinogenic activity; Positive = no evidence of carcinogenic activity), capability of blocking hERG K^+^ channels, and properties of skin permeation (Log K_p_). The calculated values of ADMET properties of compounds **16** and **17** are presented in Table 4.

**Figure 6:**
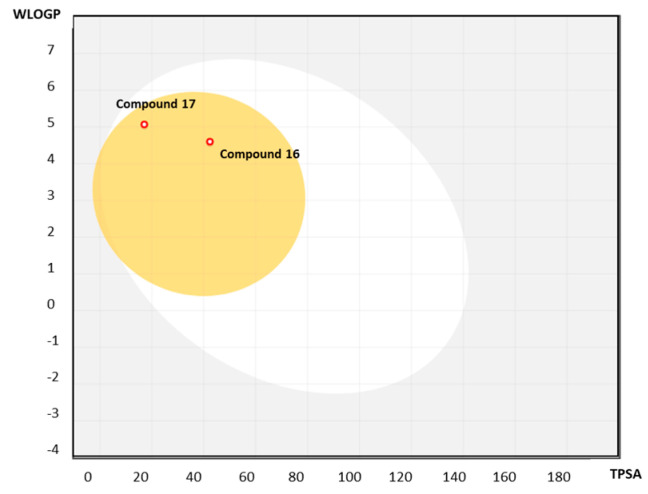
BOILED-Egg predictive model for compounds **16** and **17**. The white region is the physicochemical space of molecules with the highest probability of being absorbed by the gastrointestinal tract, and the yellow region (yolk) is the physicochemical space of molecules with the highest probability of permeating to the brain. The yolk and white areas are not mutually exclusive.

Considering the absorption parameters, chalcone **16** and diarylpentanoid **17** have a high probability of being highly absorbed (GI absorption = high; HIA = 100%) and presented middle permeability in Caco-2 cells (56.90–57.50 nm·s^−1^). Interestingly, both compounds have a high probability of not being subtracts of the P-gp efflux pump. P-gp is found in a variety of organs, including the BBB and the brush border membrane of the small intestine [46]. The presence of this efflux pump in the small intestine’s brush border may pump out orally absorbed anticancer agents, reducing the drug’s bioavailability. Given the data obtained for **16** and **17** for GI absorption and P-gp, we can speculate that these compounds may have an interesting bioavailability. Analyzing the remaining distribution parameters, **16** and **17** have a good probability of being BBB permeates as well as a good probability of establishing chemicals strongly bound with plasma protein (PPB = 100%). Interestingly, using the Brain Or IntestinaL EstimateD permeation method (BOILED-Egg) (Figure 6), which allow the simultaneous estimation of the brain access and gastrointestinal absorption of molecules, it is possible to predict that compounds **16** and **17** have a high probability of permeating through the BBB to access the CNS, with them not being predicted to be substrates of the P-glycoprotein (red point) (Figure 6).

Considering the ability to inhibit isoforms of CYP450, **16** and **17** were predicted to inhibit at least five of the nine major isoforms. Lastly, compound **17** has no evidence of carcinogenic activity, and both compounds have a medium risk of blocking hERG K^+^ channels.

#### 2.3.3. Docking and Molecular Dynamics Studies

A well-defined hydrophobic surface pocket in MDM2 mediates p53–MDM2 interactions, and at least three hydrophobic residues of the α-helix in p53 (Phe-19, Trp-23, and Leu-26) play a crucial role in the interaction [47,48]. The binding of MDM2 to p53 is established by van der Waals interactions and by polar interactions between the nitrogen of the p53 Trp23 and MDM2 Leu54 carbonyl group and the p53 Phe19 backbone amide and the MDM2 Gln72 carbonyl group [49].

**Figure 7:**
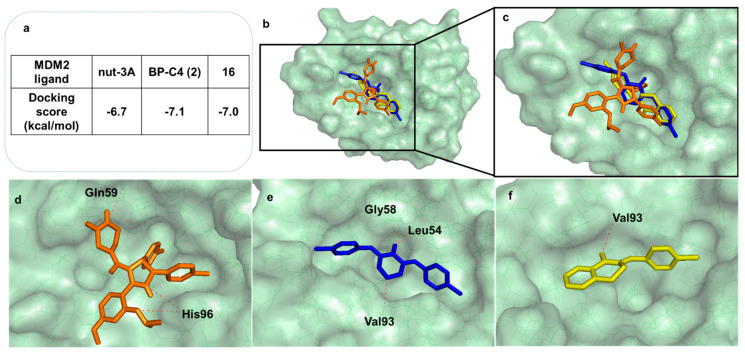
Docking results of compound **16** onto the MDM2 target. (**a**) Docking scores of positive control Nutlin-3, hit compound **BP-C4** (**2**), and **16** onto the MDM2 target. (**b**) All tested compounds docked in MDM2 (pale green transparent solid surface). (**c**) Predicted binding poses of Nutlin-3A (orange sticks), **BP-C4** (**2**) (blue sticks), and **16** (yellow sticks) in the binding site of MDM2 (pale green transparent solid surface). (**d**) Predicted binding poses of Nutlin-3A (represented in orange sticks) in the binding site of MDM2. (**e**) Predicted binding poses of **BP-C4** (**2**) (represented in blue sticks) in the binding site of MDM2. (**f**) Predicted binding poses of **16** (represented in yellow sticks) in the binding site of MDM2. MDM2 is represented as a pale green transparent solid surface. Hydrogen interactions are depicted with a dashed red line. Residues evolved in polar interactions are labelled. (For interpretation of the references to color in this figure legend, the reader is referred to the web version of this article.).

A docking study of compound **16** on MDM2 (PDB id: 4HG7) was carried out to better characterize the activity profile of compound **16**. AutoDock Vina was selected to predict docking conformations and scores since it has been reported as the best software for predicting crystallographic p53-MDM2 inhibitor poses (RMSD< 1.0 Å) by redocking tests [50]. The results are represented in Figure 7. Compound **16**, which was identified as a p53–MDM2 interaction inhibitor in the yeast assay, presented a similar docking score to nutlin-3A (**16**: −7.0 kcalmol^−1^; nutlin-3A: −6.7 kcalmol^−1^), a known p53-MDM2 inhibitor [51], as well as the hit compound **BP-C4** (**1**) (**16**: −7.0 kcalmol^−1^; nutlin-3A: −7.1 kcalmol^−1^).

To further understand the activity profile of **16** in comparison to **BP-C4** (**2**), docking poses and residues involved in the potential p53–MDM2 interaction were also examined (Figure 7b–f). Figure 7d depicts the binding conformation of crystallographic nutlin-3A into MDM2. The most stable binding poses of **BP-C4** into MDM2 as suggested by the docking protocol are presented in Figure 7e. Like **BP-C4** (**2**), **16** was predicted to bind stably to the MDM2 binding pocket, establishing one hydrogen interaction with Val-93 (Figure 7f). Additionally, **16** also establishes non-polar interactions with MDM2. These interactions are also mentioned as being critical for p53–MDM2 interaction [52]. It should be noted from the docking results that halogenated rings are predicted to orient the molecule in the binding site, as previously reported by our group [21].

Molecular docking using a rigid target may not be enough for the prediction of the structure of the ligand:target complex. In fact, the use of the more computationally demanding but accurate MD techniques adds great value to docking [53]. MD can be used after a docking study in order to more accurately predict the ligand binding mechanism and compute more precisely the intermolecular interaction, thus improving the structures of the ligand:target complexes that resulted from a docking study [54].

In order to further comprehend the energetic and geometric pattern of the compound **16**:MDM2 complex in an aqueous environment, a 5 ns MD simulation was performed based on the most stable complex structure obtained from the docking study considering the effects of both target flexibility and explicit solvation.

**Figure 8:**
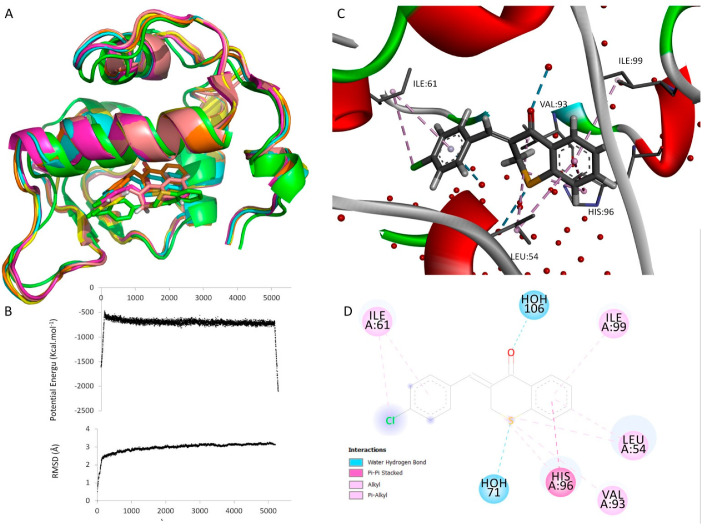
Analysis of MDM2 bound to compound 16 after docking and molecular dynamics simulation. (**A**) Superposition of the compound **16**:MDM2 complexes during the MD simulations (t0 = green, t1000 = pink, t2000 = yellow, t3000 = salmon, t4000 = orange, and t5200 = blue). (**B**) Potential energy plot (top) and RMSD value plot (bottom) of the complex during MD simulation. (**C**) Detailed 3D view of the binding mode of compound X to MDM2 after MD. Non-covalent interactions are represented as broken lines. Residues involved in those interactions are labeled. Water molecules are represented as red spheres. (**D**) Two-dimensional diagram representing the intermolecular interactions between compound **16** and MDM2 after MD. Dashed lines represent the different interactions and their color represents the interaction type. Colored circles represent residues with their three-letter code, chain identifier and residue number. The solvent accessible surface of an interacting residue is represented by a halo around the residue. The diameter of the circle is proportional to the solvent accessible surface.

When MD was performed on compound **16** top docked conformation, this compound assumed a similar location and it is positioned in the same area along the 5200 ps simulation, as illustrated in Figure 8A, indicating that it can be stably positioned in the docking site.

The outcome of the MD simulations demonstrated the stability of the complex and offered more details regarding compound **16’s** mechanism of binding. The MD simulation result indicates that the complex structure is essentially in a stable condition because the potential energy at the end of the simulation is lower than the initial conformation, suggesting more stability of the system, and the RMSD tends to be steady and fluctuates around 3Å (Figure 8B).

After the MD, compound **16** went deeper into the binding groove and this could significantly increase the binding affinity. Indeed, the 2,3-dihydro-4*H*-1-benzothiopyran-4-one scaffold penetrates further into the MDM2 cavity (Figure 8A,C), with concomitant adjustment of the nearby residues Ile-99, His-96, and Leu-54 (Figure 8C). A π-stacking interaction is formed between His-96 and the aromatic ring of the 2,3-dihydro-4*H*-1-benzothiopyran-4-one scaffold. Moreover, π–alkyl interactions are established between the aromatic rings of compound **16** and the residues Ile-61, Ile-99, and Leu-54. Also, alkyl interactions are established between the halogen atom of the ligand (chloride and sulfur) and residues Ile-61, His-96, and Val-93 (Figure 8D). All of these residues have already been described as important in the binding of MDM2 to p53 [55] and/or to inhibitors [55,56].

#### 2.3.4. Quantitative Structure–Activity Relationship (QSAR)

In recent decades, QSAR investigations have been used to identify the characteristics of small molecules that are important for activity and to predict the activity of novel compounds [57]. As a result, a QSAR model was developed to emphasize the descriptors that are important for the growth inhibition activity of chalcones and diarylpentanoid derivatives on the HCT116 p53^+/+^ tumor cell line. A QSAR model also enables efforts to be focused on the synthesis of molecules with higher chances of exhibiting the desired activity [58]. Comprehensive Descriptors for Structural and Statistical Analysis (CODESSA 2.7.2) software program, which has hundreds of descriptors, was used in this study to develop a 2D-QSAR model. The heuristic method starts with the preselection of descriptors and eliminates any that are not available for all structures, have a small magnitude variation across all structures, are correlated pairwise, or have no statistical significance. The heuristic technique is a useful resource for finding the most relevant set of descriptors, with no limitations on the size of the data collection [59].

The squared standard error (S^2^), Fisher’s value (F), and the correlation coefficient (R^2^) were used to assess the reliability of the regression equation [59]. Five descriptors were utilized to construct the QSAR equation because the requirements of the QSAR mandate that there must be one descriptor for every five molecules used to construct the model [60].

The multilinear regression analysis using the heuristic method for 25 compounds in the five-parameter model is given in Equation (1):

pGI_50_ = −0.9136 (±1.7354) −3.0874 (±1.2789) × ABIC2 + 13.643 (±8.1444) × RNSA + 4.6709 (±1.7685) × XYS/XYR + 1.0132 × 10^−3^ (±4.5879 × 10^−4^) × WPSA-2-W-PPSA + 5.6120 (±4.6556) × MPCO (n = 25, R^2^ = 0.7217, F = 9.85, S^2^ = 0.1029, Q^2^ = 0.6650) (1)

where ABIC2 stands for average bonding information content (order 2), RNSA stands for the relative number of sulfur atoms, XYS/XYR stands for XY Shadow/XY Rectangle, WPSA-2-W-PPSA stands for WPSA-2 Weighted PPSA (PPSA2 × TMSA/1000), and MPCO stands for minimal partial charge for an oxygen atom.

Despite the modest number of molecules utilized to develop the model, the best QSAR model presented a squared correlation coefficient (R^2^) of 0.7217, a Fisher value of 9.85, and an S^2^ of 0.1029, which suggests that the model has statistical stability and validity. R^2^ is a measure of how well a regression equation fits the data. As a high R^2^ was obtained (R^2^ = 0.7217) for the test set data, it was ensured that the model fits the data well [61]. The F test value is the degree of statistical significance and measures the proportion of variation explained by the model to variance brought on by regression error. According to the Fisher value, the QSAR model is significant at a 95% level [59]. The model has quality and little fluctuation around the regression line, as indicated by the low squared standard error (S^2^ = 0.1029)[62]. Two distinct forms of validation criteria were used to analyze the dependability of the final QSAR model: internal leave-one-out (LOO) cross-validation and external validation using a test set [59]. With an average deviation from the experimental values of 0.38, the model was able to forecast the behavior of an external test set [63]. Additionally, the cross-validation R^2^ (Q^2^ = 0.6650) from the LOO internal validation process is higher than 0.5 and smaller than the overall R^2^, as expected, and the difference between R^2^ and Q^2^ is lower than 0.3, indicating that the model is not overfitted [64].

It is feasible to gain some understanding of the structural properties that are probably important for the growth inhibitory activity of the examined compounds by analyzing the descriptors in the regression model. The potency of the compounds was shown to be significantly influenced by five variables. Descriptors which appear in this model are topological (average bonding information content of order 2), constitutional (relative number of sulfur atoms), quantum–chemical (WPSA-2 weighted PPSA), geometrical (XY shadow/XY rectangle), and electrostatic (minimal partial charge of an oxygen atom).

Four of the descriptors—relative number of sulfur atoms, XY shadow/XY rectangle, WPSA-2 weighted PPSA and minimal partial charge for a oxygen atom—have positive regression coefficients, which means that an increase in these descriptors will originate in an increase in growth inhibitory activity. As opposed to that, the average bonding information content (order 2) descriptor has a negative regression coefficient, demonstrating that the growth inhibitory activity of the molecules will decrease as the values of these descriptor rises.

The presence of sulfur, which is less electronegative and 60% larger than oxygen, contributes positively to the activity. Also, the activity increases with increasing the value of XY-Shadow/XY-Rectangle, which is a geometrical descriptor related to the size and shape of the molecule, meaning that a higher area of molecular shadow in the enclosing rectangle will benefit the activity. WPSA-2 weighted PPSA is a surface-weighted charged partial-positive charged surface area, and it is defined as (PPSA2 × TMSA)/1000, where PPSA2 is the total charge weighted by the partial positive surface area and TMSA is the total molecular surface area. Minimal partial charge for an oxygen atom, an electrostatic descriptor related to charge distribution, also positively contributes to activity [65]. A graph vertex complexity index descriptor—average bonding information content (order 2)—is predicted as contributing negatively to growth inhibitory activity. It is a symmetry descriptor (neighborhood symmetry of second order) related to the number of bonds counting bond orders, which provides information on the complexity of the molecule [66].

All of the aforementioned information points to the QSAR model’s applicability for growth inhibitory activity analysis, which raises the possibility that the model may be able to predict the activity of additional hits.

## 3. Materials and Methods

### 3.1. Chemistry

All reactions were monitored by thin-layer chromatography (TLC). Flash column chromatography (CC) and preparative TLC using Macherey–Nagel silica gel 60 (0.04–0.063 mm), and Macherey–Nagel silica gel 60 (GF254) plates, respectively, were used in the purification of the synthesized compounds. Melting points were obtained in a Köfler microscope and are uncorrected. ^1^H and ^13^C NMR spectra were taken in CDCl_3_ at room temperature on Bruker Avance 300 and 500 instruments (300.13 or 400.14 MHz for ^1^H and 75.47 or 100.63 MHz for ^13^C). Chemical shifts are expressed in δ (ppm) values relative to tetramethylsilane (TMS) used as an internal reference; ^13^C NMR assignments were made by HSQC and HMBC experiments (the long-range ^13^C-^1^H coupling constants were optimized to 7 Hz). The spectral treatment was executed using MestReNova v6.0.2-5475 software. High-resolution mass spectrometry (HRMS) was performed on an LTQ OrbitrapTM XL hybrid mass spectrometer (Thermo Fischer Scientific, Bremen, Germany) controlled by LTQ Tune Plus 2.5.5 and Xcalibur 2.1.0. at CEMUP—University of Porto, Portugal. Ions were generated using a Combi MALDI electrospray ionization (ESI) source. All reagents were purchased from Sigma Aldrich (St. Louis, MO, USA). The high performance liquid chromatography (HPLC) system consisted of a Thermo Scientific SpectraSystem P4000 pump, equipped with a degasser, a Thermo Scientific SpectraSystem AS3000 autosampler fitted with a maximum volume 100 µL loop, and a Thermo Scientific SpectraSystem UV8000 DAD detector (Waltham, MA, USA). Data acquisition was performed using ChromQuest 5.0 software, version 3.2.1. The column used in this study was ACE-C18 (150 × 4.6 mm I.D., particle size 5 µm) manufactured by Advanced Chromatography Technologies Ltd. (Aberdeen, Scotland, UK). The mobile phase composition was acetonitrile and water (70:30 *v*/*v* for compounds **3**–**17**, **19**–**24**, and **26**–**36**) or acetonitrile and water (70:30 *v*/*v;* 0.1%CH_3_CO_2_H for compounds **18**, **38**, and **40**); all were HPLC grade solvents obtained from Merck Life Science S.L.U. (Darmstadt, Germany). The flow rate was 1.0 mL/min and the UV detection wavelength was set at 254 nm. Analyses were performed at room temperature in isocratic mode in a 30 min run. Peak purity index was determined by total peak UV-Vis spectra between 210–650 nm with a step of 4 nm.

#### 3.1.1. General Procedure for the Synthesis of Compounds of Group A1 (**3**–**16**)

An aqueous solution of 40% sodium hydroxide was added to a solution of appropriate ketone (100–500 mg, 0.52–6.84 mmol, 1 eq.) in methanol until pH 13–14. Then, a solution of 4-chloro or 3,4,5-trimethoxybenzaldehyde (190–1340 mg, 0.97–6.84 mmol, 2eq.) in methanol was slowly added to the reaction mixture. The reaction was left at room temperature for 22 h–7 days and was monitored by TLC. After, crushed ice was added to the reaction mixture and neutralized with 5 M HCl solution. For the synthesis of compounds **11** and **15** after the addition of crushed ice, the solution was extracted with chloroform (3 × 50 mL), and the organic layers were collected, washed with water, dried with anhydrous sodium sulfate, and concentrated under reduced pressure. The obtained residue was purified as indicated below for the referred compounds. For the synthesis of compounds **3**–**10**, **12**–**14,** and **16,** the obtained solid was filtrated, washed with water, dried, and purified as indicated below for the referred compounds.

An experimental description of compounds **3**, **5**, **6**, **8**, **9**, **11**, and **13**–**16** is presented in the Supporting Information. NMR and HRMS data of compounds **4**, **7**, **10**, **12,** and **14** are described here for the first time, as indicated below.

**(*E*)-2-(3,4,5-trimethoxybenzylidene)-4,5-dimethoxy-2,3-dihydro-1*H*-inden-1-one** (**4**): Purified by crystallization from methanol. Yield: 86% as a white solid; 99.8% purity; **mp** 165–168 °C (methanol); **^1^H NMR** (CDCl_3_, 300.13 MHz) *δ*: 7.68 (d, *J* = 8.3 Hz, 1H, H-7), 7.54 (t, *J* = 2.2 Hz, 1H, H-1″), 7.02 (d, *J* = 8.4 Hz, 1H, H-6), 6.91 (s, 2H, H-2′,-6′), 3.99 (d, *J* = 2.0 Hz, 2H, H-3), 3.99 (s, 3H, 5-OCH_3_), 3.98 (s, 3H, 4-OCH_3_), 3.94 (s, 6H, 3′,5′-OCH_3_), 3.91 (s, 3H, 4′-OCH_3_) ppm; **^13^C NMR** (CDCl_3_, 75.47 MHz) *δ:* 192.9 (C=O), 157.7 (C-5), 153.5 (C-3′,-5′), 145.3 (C-4), 142.3 (C-9), 139.8 (C-4′), 134.3 (C-2), 133.6 (C-1′’), 133.1 (C-1′), 132.2 (C-8), 121.2 (C-7), 112.7 (C-6), 61.1 (4′-OCH_3_), 60.7 (4-OCH_3_), 56.5 (3′-,5′-OCH_3_), 56.4 (5-OCH_3_), 29.1 (C-3) ppm; **HRMS** (ESI^+^): *m*/*z* Anal. Calc. for C_21_H_22_O_6_ (M + H^+^) 371.14892, found 371.14859.
**(*E*)-2-(4-chlorobenzylidene)-4,5-dimethoxy-2,3-dihydro-1*H*-inden-1-one** (**7**): Purified by crystallization from methanol. Yield: 65% as a white solid; 99.9% purity; **mp** 206- 209 °C (methanol); **^1^H NMR** (CDCl_3_, 300.13 MHz) *δ*: 7.68 (d, *J* = 8.3 Hz, 1H, H-7), 7.61 (d, *J* = 8.6 Hz, 2H, H-2′, -6′), 7.56 (t, *J* = 2.2 Hz, 1H, H-1′′), 7.43 (d, *J* = 8.6 Hz, 2H, H-3′, -5′), 7.03 (d, *J* = 8.0 Hz, 1H, H-6), 3.98 (s, 3H, 5-OCH_3_), 3.97 (s, 3H, 4-OCH_3_), 3.96 (sl, 2H, H-3) ppm; **^13^C NMR** (CDCl_3_, 75.47 MHz) *δ:* 192.8 (C=O), 157.9 (C-5), 145.3 (C-4), 142.5 (C-9), 135.8 (C-4′), 135.7 (C-2), 134.0 (C-1′), 132.1 (C-8), 132.0 (C-1′’), 131.9 (C-2′, -6′), 129.3 (C-3′,-5′), 121.4 (C-7), 112.7 (C-6), 61.5 (4-OCH_3_), 57.4 (5-OCH_3_), 28.6 (C-3) ppm; **HRMS** (ESI^+^): *m*/*z* Anal. Calc. for C_18_H_16_ClO_3_ (M + H^+^) 315.07825, found 315.07798.
**(*E*)-2-(3,4,5-trimethoxybenzylidene)-6,7-dimethoxy-3,4-dihydronaphthalen-1(2*H*)-one** (**10**): Purified by crystallization from methanol. Yield: 35% as a light yellow solid; 99.9% purity; **mp** 145–147 °C (methanol); **^1^H NMR** (CDCl_3_, 300.13 MHz) *δ*: 7.75 (sl, 1H, H-1′′), 7.62 (s, 1H, H-8), 6.68 (s, 1H, H-5), 6.66 (s, 2H, H-2′,-6′), 3.95 (s, 6H, 6-, 7-OCH_3_), 3.89 (s, 3H, 4′-OCH_3_), 3.88 (s, 6H, 3′-,5′-OCH_3_), 3.15 (td, *J* = 6.4; 1.8 Hz, 2H, H-3), 2.91 (t, *J* = 6.4 Hz, 2H, H-4) ppm; **^13^C NMR** (CDCl_3_, 75.47 MHz) *δ*: 186.7 (C=O), 153.7 (C-7), 153.2 (C-3′,-5′), 148.4 (C-6), 138.6 (C-10), 138.2 (C-4′), 136.3 (C-1′′), 135.0 (C-2), 131.7 (C-1′), 126.7 (C-9), 110.0 (C-8), 109.7 (C-5), 107.3 (C-2′,-6′), 61.1 (4′-OCH_3_), 55.3 (6-, 7-OCH_3_), 55.2 (3′-,5′-OCH_3_), 28.7 (C-4), 27.7 (C-3) ppm. **HRMS** (ESI^+^): *m*/*z* Anal. Calc. for C_22_H_25_O_6_ (M + H^+^) 385.16156. found 385.16442.
**(*Z*)-3-(3,4,5-trimethoxybenzylidene)thiochroman-4-one** (**12**): Purified by crystallization with methanol. Yield: 81% as light orange; 99.8% purity; **mp** 115–118 °C (methanol); **^1^H NMR** (CDCl_3_, 300.13 MHz) *δ*: 8.60 (dt, *J* = 7.7; 1.4 Hz, 1H, H-8), 7.61–7.57 (m, 2H, H-6, -7). 7.56–7.52 (m, 1H, H-5), 7.42 (t, *J* = 1.2 Hz, 1H, H-1′′), 6.49 (s, 2H, H-2′, -6′), 3.94 (sl, 2H, H-3), 3.84 (s, 9H, 3′-,4′-,5′-OCH_3_) ppm; **^13^C NMR** (CDCl_3_, 75.47 MHz) *δ*: 179.2 (C=O), 153.5 (C-3′,-5′), 137.4 (C-10), 136.7 (C-4′), 134.6 (C-2), 134.4 (C-1′′), 131.7 (C-9), 131.2 (C-6), 129.2 (C-8), 127.7 (C-5), 126.6 (C-7), 125.2 (C-1′), 106.6 (C-2′,-6′), 61.0 (4′-OCH_3_), 56.3 (3′-, 5′-OCH_3_), 38.1 (C-3) ppm. **HRMS** (ESI^+^): *m*/*z* Anal. Calc. for C_19_H_19_O_4_S (M + H^+^) 343.09986, found 343.09779.
**(*E*)-2-(4-chlorobenzylidene)-6,7-dimethoxy-3,4-dihydronaphthalen-1(2*H*)-one** (**14**): Purified by crystallization from methanol. Yield: 73% as a light yellow solid; 99.8% purity; **mp** 124–125 °C (methanol); **^1^H NMR** (CDCl_3_, 300.13 MHz) *δ*: 7.75 (t, *J* = 1.8 Hz, 1H, H-1′′), 7.62 (s, 1H, H-8), 7.40–7.33 (m, 4H, H-2′, H-3′,-5′,-6′), 6.68 (s, 1H, H-5), 3.95 (s, 6H, 6-, 7-OCH_3_), 3.08 (td, *J* = 6.5; 1.8 Hz, 2H, H-3), 2.89 (t, *J* = 6.4 Hz, 2H, H-4) ppm; **^13^C NMR** (CDCl_3_, 75.47 MHz) *δ*: 186.8 (C=O), 153.8 (C-6), 148.4 (C-7), 138.3 (C-10), 136.1 (C-2), 134.7 (C-1′′), 134.6 (C-4′), 134.4 (C-1′), 131.2 (C-2′,-6′), 128.8 (C-3′,-5′), 126.6 (C-9), 110.9 (C-5), 109.7 (C-8), 58.1 (6-,7-OCH_3_), 28.3 (C-4), 27.2 (C-3) ppm. **HRMS** (ESI^+^): *m*/*z* Anal. Calc. for C_19_H_17_ClO_3_ (M + H^+^) 329.09390. found 329.09352.

#### 3.1.2. Synthesis of Compounds of Group A2

Synthesis of 1-(2-hydroxy-4-methoxy-3-propylphenyl) ethan-1-one (**25**)

1-(2-hydroxy-4-methoxy-3-propylphenyl) ethan-1-one (**25**) was synthesized (90% yield) and characterized according to the described procedure [23]. An experimental description of compound **25** is presented in the Supporting Information.

Synthesis of (*E*)-3-(3,4,5-trimethoxy-chlorophenyl)-1-(2-hydroxy-4-methoxy-3-propylphenyl)prop-2-en-1-one (**1**)

(*E*)-3-(3,4,5-trimethoxy-chlorophenyl)-1-(2-hydroxy-4-methoxy-3-propylphenyl)prop-2-en-1-one (**1**) was synthesized (10%) and characterized according to the described procedure [23]. An experimental description of compound **1** is presented in the Supporting Information.

Synthesis of (*E*)-1-(4-methoxy-2-((3-methylbut-2-en-1-yl)oxy)-3-propylphenyl)-3-(3,4,5-trimethoxyphenyl)prop-2-en-1-one (**26**)

To a solution of chalcone (*E*)-1-(2-hydroxy-4-methoxy-3-propylphenyl)-3-(3,4,5-trimethoxyphenyl)prop-2-en-1-one (100 mg, 259 µmol, 1 eq.) and tetrabutylammonium hydroxide 30-hydrate (414 mg, 518 µmol, 2 eq.) in chloroform/ toluene 10:7 (17 mL), 1-bromo-3-methylbut-2-ene (57.8 mg, 388 µmol, 1.5 eq.) was added, and the reaction was allowed to proceed at room temperature for 1.5 h under gentle stirring. The reaction mixture was then poured into a mixture of water and ice and extracted with chloroform (3x50 mL). The combined organic layers were washed with brine solution, rinsed with water, dried over anhydrous sodium sulfate, and evaporated under reduced pressure. The resulting crude product was purified by TLC chromatography (SiO2, dichloromethane: n-hexane, 6:4). A light yellow solid (66% yield) corresponding to (E)-1-(4-methoxy-2-((3-methylbut-2-en-1-yl)oxy)-3-propylphenyl)-3-(3,4,5-trimethoxyphenyl)prop-2-en-1-one (**26**) was obtained.

**(*E*)-1-(4-methoxy-2-((3-methylbut-2-en-1-yl)oxy)-3-propylphenyl)-3-(3,4,5-trimethoxyphenyl)prop-2-en-1-one** (**26**): **mp** 100–103 °C (dichloromethane); 99.9% purity; **^1^H NMR** (CDCl_3_, 300.13 MHz) *δ*: 7.64 (d, *J* = 15.7 Hz, 1H, H-β), 7.62 (d, *J* = 8.6 Hz, 1H, H-6), 7.50 (d, *J* = 15.7 Hz, 1H, H-α), 6.84 (s, 2H, H-2′,-6′), 6.73 (d, *J* = 8.7 Hz, 1H, H-5), 5.46–5.40 (m, 2H, H-2‴), 4.28 (d, *J* = 7.2 Hz, 2H, H-1‴), 3.90 (s, 6H, 3′-,5′-OCH_3_), 3.89 (s, 3H, 4′-OCH_3_), 3.88 (s, 3H, 4-OCH_3_), 2.66 (t, *J* = 7.8 Hz, 3H, H-2′’) 1.59–1.54 (m, 2H, H-1′’), 1.62 (s, 3H, H-4‴), 1.50 (s, 3H, H-5‴), 1.00 (t, *J* = 7.4 Hz, 3H, H-3′′) ppm; **^13^C NMR** (CDCl_3_, 75.47 MHz) *δ*: 191.5 (C=O), 162.0 (C-4), 157.9 (C-2), 153.4 (C-3′,-5′), 142.8 (C-β), 140.1 (C-4′), 138.3 (C-4‴), 131.0 (C-1′), 129.8 (C-6), 126.7 (C-1), 126.1 (C-α), 125.4 (C-3), 120.1 (C-2‴), 106.4 (C-5), 105.6 (C-2′,-6′), 73.1 (C-1‴), 61.1 (4′-OCH_3_), 56.3 (3′-,5′-OCH_3_), 55.8 (4-OCH_3_), 26.1 (C-1′′), 25.8 (C-4‴), 23.0 (C-2′′), 18.0 (C-5‴), 14.7 (C-3′′) ppm; **HRMS** (ESI^+^): *m*/*z* Anal. Calc. for C_27_H_34_O_6_K (M + K^+^) 493.19870, found 493.19794.

#### 3.1.3. General Procedure for the Synthesis of Compounds of Group A3 (**17**–**24**)

An experimental description and the NMR data of compounds **17**–**24** is presented in the Supporting Information.

#### 3.1.4. Synthesis of Compounds of Group B1

**Synthesis of 7-methoxy-8-propyl-2-(3,4,5-trimethoxyphenyl)-4H-chromen-4-one** (**27**)

To a solution of (*E*)-1-(2-hydroxy-4-methoxy-3-propylphenyl)-3-(3,4,5-trimethoxyphenyl)prop-2-en-1-one (**1**, 100 mg, 259 µmol, 1 eq.) in DMSO (1mL/mmol of chalcone), I_2_ (657 µg, 2.59 µmol, 0.01 eq.) was added. The mixture was heated at reflux for 24 h and then the mixture was poured into ice and 10% sodium thiosulfate solution was added. The obtained mixture was extracted with ethyl acetate, washed with water, and then dried over anhydrous sodium sulfate. The solvent was evaporated under reduced pressure to dryness and the obtained oil was purified by TLC (SiO_2_, dichloromethane: n-hexane (9:1)). A light yellow solid (60% yield) corresponding to 7-methoxy-8-propyl-2-(3,4,5-trimethoxyphenyl)-4*H*-chromen-4-one (**27**) was obtained.

**7-methoxy-8-propyl-2-(3,4,5-trimethoxyphenyl)-4*H*-chromen-4-one** (**27**): **mp** 153–155 °C (dichloromethane); 99.8% purity; **^1^H NMR** (CDCl_3_, 300.13 MHz) *δ*: 8.09 (d, *J* = 8.9 Hz, 1H, H-5), 7.16 (s, 2H, H-2′,-6′), 7.02 (d, *J* = 8.9 Hz, 1H, H-6), 6.72 (s, 1H, H-3), 3.96 (s, 3H, 4′-OCH_3_), 4.95 (s, 6H, 3′-5′-OCH_3_), 3.93 (s, 3H, 7-OCH_3_), 2.95 (t, *J* = 7.6 Hz, 2H, H-1′′), 1.75–1.67 (m, 2H, H-2′′), 1.03 (t, *J* = 7.4 Hz, 3H, H-3′′) ppm; **^13^C NMR** (CDCl_3_, 75.47 MHz) *δ*: 178.7 (C=O), 162.7 (C-2), 161.6 (C-7), 155.3 (C-9), 153.7 (C-3′, -5′), 141.1 (C-4′), 127.5 (C-1′), 124.6 (C-5), 118.7 (C-8), 117.9 (C-10), 109.0 (C-6), 106.5 (C-3), 103.4 (C-2′, -6′), 61.2 (4′-OCH_3_), 56.3 (3′-,5′-OCH_3_), 56.2 (4-OCH_3_), 25.6 (C-1′′), 22.7 (C-2′′), 14.6 (C-3′′) ppm; **HRMS** (ESI^+^): *m*/*z* Anal. Calc. for C_22_H_25_O_6_ (M + H^+^) 385.16456, found 385.16371.

#### 3.1.5. Synthesis of Compounds of Group B2

Synthesis of (E)-6-(1-hydroxy-3-(3,4,5-trimethoxyphenyl)allyl)-3-methoxy-2-propylphenol (28) and 3,5-bis((E)-4-chlorobenzylidene)tetrahydro-2H-pyran-4-ol (**30**)

A reaction was carried out in 50 mL round-bottom flasks with (*E*)-1-(2-hydroxy-4-methoxy-3-propylphenyl)-3-(3,4,5-trimethoxyphenyl)prop-2-en-1-one (**1,** 70 mg, 0.18 mmol, 1eq.) or (*E*)-3-(4-chlorobenzyl)-5-(4-chlorobenzylidene)tetrahydro-4*H*-pyran-4-one (**2**; 50 mg, 0.14 mmol, 1 eq.) in methanol (10 mL). Mild sonication was used initially to disperse the substrate. Solid NaBH_4_ (6.0–7.5 mg, 0.20 mmol, 1.1 eq.) was then added to the magnetically stirred solution at 30 °C for 2–20 h and the reaction was monitored by TLC. After cooling, the reaction was poured into crushed ice and neutralized with 5M HCl solution. For the synthesis of compound **28** after the addition of crushed ice, the solution was extracted with chloroform (3 × 50 mL), and the organic layers were collected, washed with water, dried over with anhydrous sodium sulfate, and concentrated under reduced pressure. The obtained residue was purified as indicated below for the referred compound. For the synthesis of compound **30,** the obtained solid was filtrated, washed with water, dried, and purified as indicated below for the referred compound.

**3,5-bis((*E*)-4-chlorobenzylidene)tetrahydro-2*H*-pyran-4-ol** (**30**): Purified by TLC (SiO_2_, n-hexane: ethyl acetate, 7:3). Yield: 88% as a white solid; 98.9% purity; **mp** 219–222 °C (methanol); **^1^H NMR** (CDCl_3_, 400.14 MHz) *δ*: 7.32 (d, *J* = 6.4 Hz, 4H, H-3′,-5′), 7.10 (d, *J* = 6.4 Hz, 4H, H-2′,-6′), 6.62 (sl, 2H, H-1′′), 4.91 (d, *J* = 4.0 Hz, 1-OH), 4.69 (dd, *J* = 10.8; 2.0 Hz, 2H, H-3ª,-5a), 4.51 (dd, *J* = 10.9; 2.0 Hz, 2H, H-3b,-5b) ppm; **^13^C NMR** (CDCl_3_, 100.63 MHz) *δ*: 139.4 (C-2), 134.6 (C-1′), 133.3 (C-4′), 130.2 (C-3′,-5′), 128.8 (C-2′,-6′), 124.3 (C-1′’), 75.5 (C-1), 66.8 (C-3,-5) ppm. **HRMS** (ESI^+^): *m*/*z* Anal. Calc. for C_19_H_16_Cl_2_O_2_ (M + k^+^) 385.01598. found 385.01545.

#### 3.1.6. Synthesis of Compounds of Group B3

Synthesis of 1-(2-hydroxy-4-methoxy-3-propylphenyl)-3-(3,4,5-trimethoxyphenyl)propan-1-one (**29**) and 3,5-bis(4-chlorobenzyl)tetrahydro-4*H*-pyran-4-one (**31**)

A 10 mL round-bottom flask with stir bar was charged with 0.2 equivalents of 10% wt Pd/C, (*E*)-1-(2-hydroxy-4-methoxy-3-propylphenyl)-3-(3,4,5-trimethoxyphenyl)prop-2-en-1-one (**1**; 20 mg, 52 µmol, 1 eq.) or 3,5-bis((*E*)-4-chlorobenzylidene)tetrahydro-4*H*-pyran-4-one (**2**, 200 mg, 579 µmol, 1 eq.) and 4 mL of toluene. The reaction mixture was left to stir in a H_2_ atmosphere at room temperature for 2–3.5 h. The final product was filtered through celite, and recovery with ethyl acetate was carried out to remove the Pd/C catalyst from the reaction mixture. The solvent was evaporated under reduced pressure. The obtained residues were purified as indicated below for the referred compounds.

**1-(2-hydroxy-4-methoxy-3-propylphenyl)-3-(3,4,5-trimethoxyphenyl)propan-1-one** (**29**): Purified by TLC (SiO_2_; dichloromethane: n-hexane, 8:2).Yield: 85% as a yellow gum; 99.8% purity; **mp** N.D. (gum); **^1^H NMR** (CDCl_3_, 300.13 MHz) *δ*: 12.78 (s, 1H, 2-OH), 7.60 (d, *J* = 9.0 Hz, 1H, H-6), 6.45 (s, 2H, H-2′,-6′), 6.43 (d, *J* = 9.0 Hz, 1H, H-5), 3.87 (s, 3H, 4-OCH_3_), 3.84 (s, 6H, 3′-,5′-OCH_3_), 3.82 (s, 3H, 4′-OCH_3_), 3.24 (t, *J* = 7.5 Hz, 2H, H-1‴), 3.00 (t, *J* = 7.7 Hz, 2H, H-2‴), 2.63 (t, *J* = 7.6 Hz, 2H, H-1′′), 1.58–1.48 (m, 2H, H-2′’), 0.94 (t, *J* = 7.4 Hz, 3H, H-3′′) ppm; **^13^C NMR** (CDCl_3_. 75.47 MHz) *δ*: 204.2 (C=O), 163.6 (C-4), 162.3 (C-2), 153.4 (C-3′, -5′), 137.0 (C-1′), 136.5 (C-4′), 129.4 (C-6),118.6 (C-3), 113.9 (C-1), 105.5 (C-2′, -6′), 102.1 (C-5), 61.0 (4′-OCH_3_), 56.2 (3′-,5′-OCH_3_), 55.8 (4-OCH_3_), 40.0 (C-1‴), 31.2 (C-2‴), 24.5 (C-1′′), 22.0 (C-2′′), 14.3 (C-3′′) ppm; **HRMS** (ESI^+^): *m*/*z* Anal. Calc. for C_22_H_29_O_6_ (M+H^+^) 389.19587, found 389.19580.
**3,5-bis(4-chlorobenzyl)tetrahydro-4*H*-pyran-4-one** (**31**): Purified by crystallization from methanol. Yield: 50% as a white solid; 98.2% purity; **mp** 145–146 °C (methanol); **^1^H NMR** (CDCl_3_, 300.13 MHz) *δ*: 7.25 (dt, *J* = 8.8; 2.3 Hz, 4H, H-3′,-5′), 7.09 (dt, *J* = 8.8; 2.3 Hz, 4H, H-2′,-6′), 4.14 (qd, *J* = 5.4; 1.9 Hz, 2H, H-3a), 3.34 (t, *J* = 11.1 Hz, 2H, H-3b), 2.94–2.83 (m, 2H, H-2), 3.17 (q, *J* = 7.2 Hz, 2H, H-1′′a), 2.36 (q, *J* = 7.2 Hz, 2H, H-1′′b) ppm; **^13^C NMR** (CDCl_3_. 75.47 MHz) *δ*: 208.0 (C=O), 137.7 (C-1′), 132.3 (C-4′), 130.3 (C-2′, -6′), 52.9 (C-2, -6), 128.7 (C-3′,-5′), 73.7 (C-3, -5), 30.5 (C-1′) ppm. **HRMS** (ESI^+^): *m*/*z* Anal. Calc. for C_19_H_18_Cl_2_O_2_ (M+k^+^) 387.03154. found 387.03260.

#### 3.1.7. General Procedure for the Synthesis of Compounds of Group C1 (**33**–**34**)

An aqueous solution of 40% sodium hydroxide was added to a solution of 1-(2-hydroxy-4-methoxy-3-propylphenyl)ethan-1-one (100 mg, 0.48 mmol, 1 eq.) in methanol until pH 13–14. Then, a solution of appropriate benzaldehyde (119 mg, 0.96 mmol, 2eq.) in methanol was slowly added to the reaction mixture. The reaction was left at reflux for 2 days and was monitored by TLC. After cooling, the reaction was poured into crushed ice and neutralized with 5M HCl solution. The organic layer was rinsed with brine and water, dried over anhydrous sodium sulfate, and evaporated under reduced pressure. The obtained residue was purified as indicated below for the referred compounds.

An experimental description of compound **32** is presented in the Supporting Information.

**(*E*)-3-(4-fluorophenyl)-1-(2-hydroxy-4-methoxy-3-propylphenyl)prop-2-en-1-one** (**33**): Purified by TLC (SiO_2_; n-hexane: ethyl acetate, 8:2). Yield: 12% as a yellow solid; 96.4% purity; **mp** 115–117 °C (ethyl acetate); **H NMR** (CDCl_3_, 300.13 MHz) *δ*: 13.33 (s, 1H, 2-OH), 7.84 (d, *J* = 15.4 Hz, 1H, H-β), 7.78 (d, *J* = 9.1 Hz, 1H, H-6), 7.67–7.62 (m, 2H, H-2′,-6′), 7.53 (d, *J* = 15.5 Hz, 1H, H-α), 7.16–7.08 (m, 2H, H-3′,-5′), 6.50 (d, *J* = 9.0 Hz, 1H, H-5), 3.90 (s, 3H, 4-OCH_3_), 2.66 (t, *J* = 7.7 Hz, 2H, H-1′’), 1.62–1.49 (m, 2H, H-2′’), 0.96 (t, *J* = 7.4 Hz, 3H, H-3′′) ppm; **^13^C NMR** (CDCl_3_, 75.47 MHz) *δ*: 192.2 (C=O), 163.7 (d, *J* = 27.7 Hz, C-4′), 163.8 (C-4), 163.4 (C-2), 142.9 (C-β), 130.5 (d, *J* = 8.5 Hz, C-2′, -6′), 131.3 (d, *J* = 3.3 Hz, C-1′), 129.2 (C-6), 120.5 (C-α), 118.8 (C-3), 114.5 (C-1), 102.2 (C-5), 116.3 (d, *J* = 21.9 Hz, C-3′,-5′), 56.9 (4-OCH_3_), 24.6 (C-1′′), 22.0 (C-2′′), 14.4 (C-3′′) ppm; **HRMS** (ESI^+^): *m*/*z* Anal. Calc. for C_19_H_19_FO_3_ (M + H^+^) 315.13910, found 315.13906.
**(*E*)-3-(4-bromophenyl)-1-(2-hydroxy-4-methoxy-3-propylphenyl)prop-2-en-1-one** (**34**): Purified by TLC (SiO_2_; dichloromethane: n-hexane, 5:5). Yield: 10% as an orange solid; 96.2% purity; **mp** 115–116 °C (dichloromethane); **^1^H NMR** (CDCl_3_, 300.13 MHz) *δ*: 13.29 (s, 1H, 2-OH), 7.80 (d, *J* = 15.5 Hz, 1H, H-β), 7.77 (d, *J* = 9.0 Hz, 1H, H-6), 7.59 (d, *J* = 15.5 Hz, 1H, H-α), 7.57–7.54 (m, 2H, H-2, -6), 7.52–7.49 (m, 2H, H-3, -5), 6.50 (d, *J* = 9.0 Hz, 1H, H-5), 3.90 (s, 3H, 4-OCH_3_), 2.66 (t, *J* = 7.7 Hz, 2H, H-1″), 1.59–1.49 (m, 2H, H-2″), 0.96 (t, *J* = 7.4 Hz, 3H, H-3″) ppm; **^13^C NMR** (CDCl_3_, 75.47 MHz) *δ*: 192.1 (C=O), 163.9 (C-4), 163.5 (C-2), 142.7 (C-β), 134.0 (C-1′), 132.4 (C-3′,-5′), 130.0 (C-2′,-6′), 129.2 (C-6), 125.0 (C-4′), 121.4 (C-α), 118.8 (C-3), 114.5 (C-1), 102.2 (C-5), 56.4 (4-OCH_3_), 24.6 (C-1″), 22.0 (C-2″), 14.4 (C-3″) ppm; **HRMS** (ESI^+^): *m*/*z* Anal. Calc. for C_19_H_20_BrO_3_ (M + H^+^) 375.05903, found 375.05889.

#### 3.1.8. General Procedure for the Synthesis of Compounds of Group C2 (**35** and **36**)

An aqueous solution of 40% sodium hydroxide was added to a solution of 1-(2-hydroxy-4-methoxy-3-propylphenyl)ethan-1-one (200 mg, 0.96 mmol, 1 eq.) in methanol until pH 13–14. Then, a solution of appropriate benzaldehyde (287–367 mg, 1.92 mmol, 2 eq.) in methanol was slowly added to the reaction mixture. The reaction was left at reflux for 2 days and was monitored by TLC. After cooling, the reaction was poured into crushed ice and neutralized with 5M HCl solution. The organic layer was rinsed with brine and water, dried over anhydrous sodium sulfate, and evaporated under reduced pressure. The obtained residue was purified as indicated below for the referred compounds.

**(*E*)-3-(4-(dimethylamino)phenyl)-1-(2-hydroxy-4-methoxy-3-propylphenyl)prop-2-en-1-one** (**35**): Purified by flash column chromatography (SiO_2_; n-hexane: ethyl acetate, 9:1). Yield: 15% as an orange solid; 98.7% purity; **mp** 126–128 °C (ethyl acetate); **^1^H NMR** (CDCl_3_, 300.13 MHz) *δ*: 13.71 (s, 1H, 2-OH), 7.87 (d, *J* = 15.2 Hz, 1H, H-β), 7.80 (d, *J* = 9.0 Hz, 1H, H-6), 7.56 (dt, *J* = 9.3; 2.2 Hz, 2H, H-2′,-6′), 7.41 (d, *J* = 15.2 Hz, 1H, H-α), 6.70 (dt, *J* = 9.4; 2.2 Hz, 2H, H-3′, -5′), 6.48 (d, *J* = 9.1 Hz, 1H, H-5), 3.89 (s, 3H, 4-OCH_3_), 3.05 (s, 6H, 4′-NCH_3_), 2.66 (t, *J* = 7.7 Hz, 2H, H-1″), 1.62–1.50 (m, 2H, H-2″), 0.97 (t, *J* = 7.3 Hz, 3H, H-3″) ppm; **^13^C NMR** (CDCl_3_, 75.47 MHz) *δ*: 192.5 (C=O), 163.3 (C-2), 163.2 (C-4), 152.2 (C-4′), 145.2 (C-β), 130.7 (C-2′, -6′), 128.8 (C-6), 122.8 (C-1′), 118.5 (C-3), 115.1 (C-α), 114.8 (C-1), 111.9 (C-3′,-5′), 101.8 (C-5), 55.8 (4-OCH_3_), 40.3 (4′-NCH_3_), 24.6 (C-1″), 22.1 (C-2″), 14.4 (C-3″) ppm; **HRMS** (ESI^+^): *m*/*z* Anal. Calc. for C_21_H_26_NO_3_ (M+H^+^) 340.19072, found 340.19093.
**(*E*)-1-(2-hydroxy-4-methoxy-3-propylphenyl)-3-(4-morpholinophenyl)prop-2-en-1-one** (**36**): Purified by flash column chromatography (SiO_2_; n-hexane: ethyl acetate, 9:1). Yield: 15% as an orange solid; 99.1% purity; **mp** 129–131 °C (ethyl acetate); **^1^H NMR** (CDCl_3_, 300.13 MHz) *δ*: 13.56 (s, 1H, 2-OH), 7.85 (d, *J* = 15.3 Hz, 1H, H-β), 7.80 (d, *J* = 9.1 Hz, 1H, H-6), 7.58 (d, *J* = 8.8 Hz, 2H, H-2′,-6′), 7.47 (d, *J* = 15.3 Hz, 1H, H-α), 6.90 (d, J=8.9 Hz, 2H, H-3′, -5′), 6.49 (d, *J* = 9.0 Hz, 1H, H-5), 3.90 (s, 3H, 4-OCH_3_), 3.87 (t, *J* = 4.8 Hz, 2H, H-8′), 3.28 (t, *J* = 4.9 Hz, 2H, H-7′), 2.66 (t, *J* = 7.7 Hz, 2H, H-1″), 1.59–1.52 (m, 2H, H-2″), 0.96 (t, *J* = 7.4 Hz, 3H, H-3″) ppm; **^13^C NMR** (CDCl_3_. 75.47 MHz) *δ*: 192.5 (C=O), 163.5 (C-4), 163.4 (C-2), 152.9 (C-4′), 144.4 (C-β), 130.3 (C-2′,-6′), 129.0 (C-6), 126.0 (C-1′), 118.6 (C-3), 117.1 (C-α), 114.8 (C-1, -3′, -5′), 102.0 (C-5), 66.8 (C-8′), 55.8 (4-OCH_3_), 48.1 (C-7′), 24.6 (C-1″), 22.1 (C-2″), 14.4 (C-3″) ppm. **HRMS** (ESI^+^): *m*/*z* Anal. Calc. for C_23_H_28_NO_4_ (M + H^+^) 382.20128, found 382.20104.

#### 3.1.9. General Procedure for the Synthesis of Compounds of Group C3

2-((3-methylbut-2-en-1-yl)oxy)benzaldehyde (**37**) and 4-((3-methylbut-2-en-1-yl)oxy)benzaldehyde (**39**) were synthesized (85% and 72% yield, respectively) and characterized according to the described procedures [67,68]. An axperimental description of compounds **37** and **39** are presented in the Supporting Information.

To a solution of tetrahydro-4*H*-pyran-4-one (100 mg, 0.999 mmol, 1 Eq) in methanol an aqueous solution of 40% sodium hydroxide was added until pH 13–14. Then, a solution of 2-((3-methylbut-2-en-1-yl)oxy)benzaldehyde (**37**; 570 mg, 3 mmol, 3 eq.) or 4-((3-methylbut-2-en-1-yl)oxy)benzaldehyde (**39**; 570 mg, 3 mmol, 3 eq.)) in methanol was slowly added to the reaction mixture. The reaction was left at room temperature for 26 h–2 days and was monitored by TLC. After completion, the reaction mixture was poured into ice and neutralized with diluted HCl. For the synthesis of compound **38** after the addition of crushed ice, the solution was extracted with chloroform (3 × 50 mL), and the organic layers were collected, washed with water, dried over with anhydrous sodium sulfate, and concentrated under reduced pressure. The crude material was purified as below for the referred compound. For the synthesis of compound **40,** the obtained solid was filtrated, washed with water, and purified as indicated below for the referred compound.

**3,5-bis((*E*)-2-((3-methylbut-2-en-1-yl)oxy)benzylidene)tetrahydro-4*H*-pyran-4-one** (**38**): Purified by TLC (SiO_2_, n-hexane: ethyl acetate, 8:2). Yield: 50% as an orange gum; 99.9% purity; **mp** N.D. (gum); **^1^H NMR** (CDCl_3_, 400.14 MHz) *δ*: 8.10 (sl, 2H, H-1′′), 7.32 (td, *J* = 7.9; 1.8 Hz, 2H, H-4′), 7.06 (dd, *J* = 7.5; 1.8 Hz, 2H, H-6′), 6.97–6.91 (m, 4H, H-3′, -5′), 5.51–5.47 (m, 2H, H-2‴), 4.80 (d, *J* = 2.0 Hz, 4H, H-3, -5), 4.57 (d, *J* = 6.8 Hz, 4H, H-1′’’), 1.79 (d, *J* = 1.3 Hz, 6H, H-4‴), 1.74 (d, *J* = 1.3 Hz, 6H, H-5‴) ppm; **^13^C NMR** (CDCl_3_, 100.13 MHz) *δ*: 185.9 (C=O), 157.9 (C-2′), 137.8 (C-3‴), 133.2 (C-2, -6), 132.7 (C-1′’), 130.9 (C-4′), 130.8 (C-6′), 124.5 (C-1′), 119.8 (C-2′’’), 120.1 (C-5′), 112.4 (C-3′), 69.1 (C-3, -5), 65.6 (C-1‴), 25.9 (C-4‴), 18.4 (C-5‴) ppm; **HRMS** (ESI^+^): *m*/*z* Anal. Calc. for C_29_H_31_O_4_ (M-H^+^) 443.22278, found 443.22146.
**3,5-bis((*E*)-4-((3-methylbut-2-en-1-yl)oxy)benzylidene)tetrahydro-4*H*-pyran-4-one** (**40**): Purified by crystallization from methanol. Yield: 25% as a yellow solid; 99.9% purity; **mp** 145–147 °C (methanol); **^1^H NMR** (CDCl_3_, 300.13 MHz) *δ*: 7.79 (sl, 2H, H-1′′), 7.28 (d, *J* = 8.7 Hz, 4H, H-2′,-6′), 6.95 (d, *J* = 8.7 Hz, 4H, H-3′,-5′), 5.52–5.47 (m, 2H, H2‴), 4.93 (d, *J* = 1.9 Hz, 4H, H-3,-5), 4.55 (d, *J* = 6.8 Hz, 4H, H-1‴), 1.89 (sl, 6H, H-4‴), 1.76 (sl, 6H, H-5‴) ppm; **^13^C NMR** (CDCl_3_, 75.47 MHz) *δ*: 185.6 (C=O), 160.0 (C-4′), 138.9 (C-3‴), 136.1 (C-1′′), 132.6 (C-2′,-6′), 131.3 (C-2, -6), 127.6 (C-1′), 119.3 (C-2‴), 115.0 (C-3′,-5′), 68.8 (C-3,-5), 65.5 (C-1‴), 26.0 (C-4‴), 18.4 (C-5‴) ppm; **HRMS** (ESI^+^): *m*/*z* Anal. Calc. for C_29_H_33_O_4_ (M + H^+^) 445.23734, found 445.23760.

### 3.2. Biological Activity

#### 3.2.1. Human Cell Lines and Growth Conditions

The colorectal adenocarcinoma HCT116 cells (HCT116 p53^+/+^) and its p53-null isogenic derivative (HCT116 p53^−/−^) were given by Prof. Vogelstein (The Johns Hopkins Kimmel Cancer Center, Baltimore, USA); the normal human fibroblasts HFF-1 cell lines were from ATCC (Rockville, MD, USA). Human cells were cultured in RPMI-1640 with UltraGlutamine (Lonza, VWR, Portugal) with 10% FBS (Gibco, Alfagene, Portugal) at 37 °C with 5% CO_2_. The SH-SY5Y cells were cultured in DMEM/F-12 (Lonza) and 10% FBS and 2mM L-glutamine (Gibco). The cells were incubated at 37 °C in a humidified atmosphere of 5% CO_2_. Mycoplasma was routinely checked using the MycoAlert™ PLUS kit (Lonza).

#### 3.2.2. Cell Proliferation and Viability Assays

In the sulforhodamine B (SRB) assay, the cells were seeded in 96-well plates at 5.0 × 10^3^ (HCT116, and HFF-1) cells/wells for 24 h, and the GI_50_ (the concentration that causes 50% of maximal growth inhibition) values of compounds were obtained as described [21]. In the yeast targeted screening assay, *Saccharomyces cerevisiae* (strain CG379) expressing human wt p53 alone and combined with human MDM2 were used as described [69]. For the expression of human proteins (routinely grown in minimal selective medium), the cells were diluted to 0.05 OD600 in the selective induction medium with 2% (*w*/*w*) galactose, 1% (*w*/*w*) raffinose, 0.7% (*w*/*w*) yeast nitrogen base without amino acids from Difco (Quilaban, Sintra, Portugal), and all the amino acids necessary for yeast growth (50 g/mL) except leucine and tryptophan. The yeast cells were incubated at 30 °C under continuous orbital shaking (200 rpm) with 0.1–50 M compounds or 0.1% DMSO only for over 42 h. Yeast growth was analyzed by counting the number of colony-forming units (CFUs) after 2 days of incubation at 30 °C on Sabouraud Dextrose Agar from Liofilchem (Frilabo, Porto, Portugal).

#### 3.2.3. Apoptosis Analysis

The 1.5 × 10^5^ HCT116 p53^+/+^ cells/well were seeded in 6-well plates and then treated with 5 µM **17** for 48 h. For the apoptosis analysis, an Annexin V-FITC Apoptosis Detection Kit I (BD Biosciences, Franklin Lakes, NJ, USA) was used following the manufacturer’s instructions. An AccuriTM C6 flow cytometer and the BD Accuri C6 software were used for data acquisition.

#### 3.2.4. Western Blot Analysis

For Western blotting, 1.5 × 10^5^ cells (per well) of HCT116 p53^+/+^ cells were seeded in six-well plates and allowed to adhere for 24 h, followed by treatment with 5 μM **17** for 48 h. Protein extracts obtained from the human cancer cells were quantified using the Pierce™ BCA Protein Assay Kit (Thermo Scientific, Taper, Sintra, Portugal), according to the manufacturer’s instructions. The proteins were then run in SDS-PAGE and transferred to a Whatman nitrocellulose membrane from Protan (VWR, Carnaxide, Portugal). Proteins were detected using the primary antibodies anti-p53 (Santa Cruz Biotechnology, Dallas, TX, USA, SC-126, 1:5000), anti-BCL-2 (Santa Cruz Biotechnology, SC-7382, 1:200), anti-PARP (Cell Signaling, #9542, 1:1000), and anti-GAPDH (Santa Cruz Biotechnology, SC-32233, 1:10,000), followed by HRP-conjugated secondary antibodies anti-mouse (Santa Cruz Biotechnology, SC-2005, 1:2500) and anti-rabbit (Santa Cruz Biotechnology, SC-2006, 1:2500). GAPDH was used as a loading control. The signal was detected with an ECL Amersham kit from GE Healthcare (VWR) and the ChemiDoc™ MP Imaging System (Bio-Rad, Amadora, Portugal).

#### 3.2.5. Statistical Analysis

Data analyzed using GraphPad Prism 7.0. Statistical tests were used according to the dataset; *p* values < 0.05: statistically significant.

### 3.3. In Silico Studies

#### 3.3.1. Predicting Druglikeness

The selected compounds were further investigated for druglikeness properties, namely molecular descriptors/physicochemical properties by SwissADME (http://www.swissadme.ch/ (accessed on 1 January 2023)). Medicinal chemistry rules (Ghose, Veber, Egan, Muegge, CMC-like, Lead-like, WDI-like, and MDDR-like rules were investigated by the SwissADME (http://www.swissadme.ch/ (accessed on 1 January 2023)) and PreADMET (https://preadmet.bmdrc.kr/ (accessed on 1 January 2023)) web servers. They provide a quick and detailed method for the calculation of a wide range of principal molecular descriptors that are useful in the prediction of physicochemical properties.

#### 3.3.2. ADMET Properties Prediction

The pharmacokinetic properties were evaluated using the SwissADME (http://www.swissadme.ch/ (accessed on 1 January 2023 and 8 September 2023)) [70] and preADMET (https://preadmet.bmdrc.kr/ (accessed on 1 January 2023)) web servers. SwissADME provides prediction of absorption (GI absorption), distribution (BBB permeant and P-gp substrate), and metabolism (ability to inhibit isoforms of CYP450). PreADMET provides a prediction of human intestinal absorption [71], membrane permeability using the Caco-2 cell model [72], the ability to inhibit isoforms of CYP450, and mutagenicity and cardiotoxicity, which is built from the data of the NTP (National Toxicology Program) and the US FDA.

#### 3.3.3. Docking Studies

The structures of compounds were drawn using ChemDraw 17.0. The structure of the known p53-MDM2 inhibitor nutlin-3A was obtained from Pubchem [73]. The three-dimensional (3D) structures of the compounds and control were minimized using the Austin Model 1 parameterization of the MNDO method (AM1) implemented in ArgusLab 4.0.1. The calculation was finished when the gradient between any two successive steps in the geometry search was less than 0.1 kcal^.^A^−1^mol^−1^; the maximum number of geometry steps were set to 1000. The calculation was run until the maximum steps were reached, or the convergence criteria was met, whichever came first. The 3D structure of MDM2 was obtained from the Protein Data Bank (PDB id (MDM2): 4HG7) and prepared for docking using AutoDockTools 1.5.7. Docking simulations between the MDM2 and small molecules were undertaken in PyRx 0.8 using AutoDock Vina [74]. Docking was run using an exhaustiveness of eight, engulfing the cavity occupied by the crystallographic nutlin-3A (PDB id: 4HG7) [75]. Nine conformations for each ligand were obtained. The top ranked conformations of the compounds were further analyzed concerning noncovalent interactions using Pymol 2.2.4 [76].

#### 3.3.4. Molecular Dynamics Simulation (MD)

A 12Å spherical droplet containing water molecules was placed surrounding compound **16** docked in MDM2. The X:MDM2 complex was then minimized using the MMFF94x force field until the RMS gradient < 0.1 kcal^.^Å^−1.^mol^−1^. The complex was then subjected to MD simulation using MOE-dynamic implemented in MOE 2014.09 (Chemical Computing Groups, Montreal, QC, Canada). The MD simulation was performed using an MMFF94x force field and NVT (N, total atom; V, volume; T, temperature) ensemble and the Nosé–Poincaré–Andersen (NPA) algorithm, with a 0.002 ps time step and sampling every 0.5 ps. The system was heated from 0 K to 300 K in 100 ps (heat stage), followed by a 5000 ps production stage at 300 K; the system was then cooled back to 0K in 100 ps (cooling stage). A potential energy analysis was used to keep track of the system’s behavior during the simulation. The intermolecular interactions between X and MDM2 were analyzed at the end of the simulation using Discovery Studio Visualizer v16 (Dassault Systèmes Corporate, Waltham, MA, USA).

#### 3.3.5. Quantitative Structure–Activity Relationship (QSAR)

The experimental data from the in vitro investigations (pGI_50_ on HCT116 p53^+/+^ cell line) were utilized to develop a QSAR model using thirty chalcones and diarylpentanoid derivatives. The QSAR study used pGI_50_ as a dependent variable. A training set (25 molecules) and a test set (5 molecules) were created from the 30 molecules at random. Constitutional, topological, geometrical, electrostatic, quantum–chemical, and thermodynamical molecular descriptors were computed using CODESSA software (version 2.7.10, University of Florida, USA). The optimal multilinear correlations with a wide range of descriptors were thoroughly searched by using heuristic multilinear regression methodology. The 2D-QSAR model with the best square of the correlation coefficient (R^2^), F-test (F), and squared standard error (S^2^) was selected. Utilizing the test set and internal leave-one-out (LOO) validation, the resulting model was further validated.

## 4. Conclusions

Aiming to perform the structural optimization of **CM-M345** (**1**) and **BP-C4** (**2**) and SAR studies, 33 compounds were planned, synthesized, and evaluated for their antiproliferative activity in human colorectal cancer HCT116 cells expressing *wt* p53 and its p53-null isogenic derivative HCT116 p53^−/−^ cells, as well as in human normal fibroblasts cells to assess their selectivity towards cancer cells. The majority of the compounds displayed GI_50_ values lower than 10 μM in HCT116 p53^+/+^ cells, showing diarylpentanoids **16**, **17**, **19,** and **20** (analogues of **BP-C4** (**2**)) and diarylpentanoids **22**–**24** (analogues of **CM-M345** (**1**)) lower values of GI_50_ than **CM-M345** (**1**) and **BP-C4** (**2**). When comparing the antiproliferative effect, in HCT116 p53^+/+^ cells, of **CM-M345** (**1**) and **BP-C4** (**2**) analogues, we may conclude that molecular rigidification and extension by prenylation were not favorable for antiproliferative activity. In general, most of the diarylpentanoids presented higher antiproliferative effects than chalcones. Additionally, the enone moiety seemed to be essential for a potent cytotoxic effect, with the reduction of this moiety being associated with a decrease in this activity. Moreover, the substitution of the 3,4,5-trimethoxyphenyl B ring by *para*-halogenated or *para*-aminated phenyl groups resulted in a decreased antiproliferative effect, with these results being in accordance with the results previously reported by our group [21.

Chalcones **16** and **36** were selective for p53^+/+^ HCT116 cells over p53-null cells, showing a selective index higher than **CM-M345** (**1**) and **BP-C4** (**2**), respectively. Notably, chalcone **16,** possessing a chlorine group at the *para* position on B aromatic ring, showed nine-fold higher antiproliferative activity as well as seven-fold increased selectivity for p53, compared to **BP-C4** (**2**), with this compound being identified as a p53–MDM2 interaction inhibitor by yeast cell-based assays.

Additionally, optimization of the hit compounds led to the development of diarylpentanoid **17**, with a GI_50_ value of 2.8 μM in colorectal cancer cells, and 12-fold lower cytotoxicity towards normal cells. The growth-inhibitory effect of diarylpentanoid **17** was associated with the induction of p53-independent apoptotic cell death mediated by caspases and a mitochondrial pathway. Interestingly, in silico analysis allowed to predict that chalcone **16** and diarylpentanoid **17** have adequate pharmacokinetic and druglikeness profiles. Moreover, from docking studies, it was predicted that compound **16** binds stably to the MDM2 binding pocket. After MD simulation, it was found that chalcone **16** entered more profoundly into the MDM2 binding groove, establishing π-stacking, π-alkyl, and alkyl interactions with residues already described as important in the binding of MDM2 to p53. Moreover, a QSAR model allowed us to conclude that topological, constitutional, quantum–chemical, geometrical, and electrostatic descriptors influence the growth inhibitory activity of the test compounds on the HCT116 p53^+/+^ cell line.

Further studies are required to explore the molecular mechanism of action of **16** and **17** as potential antitumor agents. The overall results obtained from this study will be valuable for the rational design of new and more selective and potent chalcones and diarylpentanoids with antiproliferative activity.

## Figures and Tables

**Table 1:** GI_50_ values of compounds **3**–**40** in human colorectal HCT116 p53^+/+^ and HCT116 p53^−/−^ cells and normal cells.

	Compound	GI_50_			SI1	SI2
		HCT116 p53^+/+^	HCT116 p53^−/−^	HFF-1		
	**CM-M345 (1)**	2.6 ± 0.2 *	3.4 ± 0.1 *	7.7 ± 0.8	1.31	2.96
Group A	**3**	4.6 ± 0.30	3.2 ± 0.47	8.1 ± 0.1	0.70	1.76
	**4**	3.8 ± 0.32	3.1 ± 0.22	5.1 ± 0.9	0.82	1.34
	**5**	6.1 ± 0.12	4.1 ± 0.36	3.2 ± 0.07	0.67	0.52
	**9**	5.9 ±1.28	7.8 ± 0.9	5.5 ± 0.03	1.32	0.93
	**10**	4.0 ± 0.23	2.9 ± 0.11	5.4 ± 0.1	0.73	1.35
	**11**	6.3 ±2.18	6.1 ± 1.0	8.3 ± 1.4	0.97	1.32
	**12**	5.7 ± 0.51	3.9 ± 0.26	6.7 ± 0.4	0.68	1.18
	**26**	6.0 ± 0.93	4.6 ± 1.7	7.3 ± 2.0	0.77	1.22
	**21**	>30	>30	>30	-----	-----
	**22**	0.09 ± 0.03	0.07 ± 0.01	0.135 ± 0.004	0.78	1.50
	**23**	2.3 ± 0.03	0.56 ± 0.07	1.65 ± 0.04	0.24	0.72
	**24**	0.31 ± 0.03	0.57 ± 0.009	0.23 ± 0.02	1.84	0.74
Group B	**27**	>30	>30	>30	----	----
	**28**	15 ± 1.73	12 ± 5.0	17 ± 4.4	0.80	1.13
	**29**	18.7 ± 2.19	11.18 ± 5.3	22.1 ± 7.2	0.60	1.18
Group C	**32**	9.5 ± 0.5	9.5 ± 0.1	11.12 ± 0.12	1	1.17
	**33**	9.25 ± 0.75	7.2 ± 0.32	8.23 ± 0.08	0.77	0.89
	**34**	18.51 ± 0.35	11.45 ± 0.145	14.89 ± 0.232	0.62	0.80
	**35**	10.7 ± 2.86	16.3 ± 2.6	14 ± 3.6	1.52	1.31
	**36**	5.1 ± 0.32	14.3 ± 1.1	15.7 ± 2.8	2.65	3.08
	**BP-C4 (2)**	6.25 ± 1.18 **	10.13 ± 0.47 **	36.2 ± 5.54 **	1.62	5.79
Group A	**6**	21.50 ± 1.19	>30	>30	-----	-----
	**7**	7.4 ± 0.56	4.5 ± 0.13	4.7 ± 0.5	0.61	0.64
	**8**	7.5 ± 0.45	4.5 ± 0.28	5.1 ± 0.9	0.60	0.68
	**13**	>30	>30	>30	-----	-----
	**14**	6.7 ± 0.21	4.3 ± 0.15	7.7 ± 0.2	0.64	1.15
	**15**	8.60 ± 0.50	5.29 ± 0.19	4.25 ± 0.32	0.62	0.49
	**16**	0.69 ± 0.07	7.95 ± 0.85	3.62 ± 0.99	11.5	5.25
	**17**	2.18 ± 0.59	3.55 ± 0.05	27.10 ± 1.25	1.63	12.43
	**18**	>30	>30	>30	-----	-----
	**19**	3.10 ± 0.10	2.95 ± 0.15	14.10 ± 0.12	0.95	4.55
	**20**	1.13 ± 0.17	1.05 ± 0.05	2.27 ± 0.09	0.93	2.01
Group B	**30**	>30	>30	>30	-----	-----
	**31**	>30	>30	>30	-----	-----
Group C	**38**	>30	>30	>30	-----	-----
	**40**	9.53 ± 0.25	5.8 ± 0.21	14.1 ± 0.08	0.61	1.48

SI1: Selective index 1 (GI_50_ of HCT116 p53^−/−^/GI_50_ of HCT116 p53^+/+^; SI2: Selective index 2 (GI_50_ of HFF-1/GI_50_ of HCT116 p53^+/+^; * results previously reported by Pereira et al. [23]; ** results previously reported by Moreira et al. [21]. The GI_50_ values were determined by the SRB assay after 48 h of treatment (growth obtained with vehicle was set as 100%). Data are the mean ± SEM of three to five independent experiments. Data are the mean ± SD and were calculated from three independent experiments. Etoposide was used as the positive control: GI_50_ (HCT116 p53^+/+^) = 0.71 ± 0.09 μM; GI_50_ (HFF-1) = 0.35 ± 0.1 μM.

**Table 2:** Druglikeness prediction of compounds **16** and **17** (molecular descriptors and physicochemical properties).

Molecular Descriptor	16	17	Guidelines
Formula	C_16_H_11_ClOS	C_17_H_12_Cl_2_O	-
MW	286.78	303.18	150 < MW < 500 * x^¯^ = 325.2 g mol^−1^ ** x^¯^ = 313 g mol^−1^ ***
Nº HA	19	20	-
Nº AHA	12	12	-
Far	0.63	0.60	-
Fsp^3^	0.06	0.00	0.25 < Fps^3^ < 1 **
RB	1	4	0 < RB < 9 **
Nº HBA	1	1	HBA ≤ 10 * x^¯^ of HBA = 4.8 **
Nº HBD	0	0	HBD ≤ 5 * x^¯^ of HBD = 1.6 **
TPSA ^(a)^	42.37	17.07	20 < PSA < 125 Å^2^ **
Log P (iLOGP) ^(a)^	3.02	3.39	Log P < 5 * 0 < Log P < 5 ***
Log P (XLOGP3) ^(a)^	4.50	5.51	
Log P (WLOGP) ^(a)^	4.60	5.07	
Log P (MLOGP) ^(a)^	4.08	4.86	
Log P (SILICOS-IT) ^(a)^	5.19	5.78	
Log P (Consensus) ^(a)^	4.28	4.92	
Mean of Log P values	4.28	4.92	-
Log S (ESOL) ^(a)^	−4.85	−5.37	Log S > −4 ***
Log S (Ali) ^(a)^	−5.11	−5.63	
Log S (SILICOS-IT) ^(a)^	−6.38	−6.26	
Mean of Log S values	−5.45	−5.75	-

* druglikeness guidelines according to Lipinski; ** druglikeness guidelines for synthetic drugs reported by Bade et al. (2010) [44]; *** druglikeness guidelines for orally bioavailable cancer drugs reported by Meanwell et al. (2011) [45]; ^(a)^ using SwissADME web server.

**Table 3:** Violations of medicinal chemistry rules by **16** and **17**, bioavailability score, and synthetic accessibility.

	16	17
Medicinal chemistry rules		
Lipinski ^(a)^	0	1
Ghose ^(a)^	0	0
Veber ^(a)^	0	0
Egan ^(a)^	0	0
Muegge ^(a)^	0	2
CMC-like ^(b)^	0	0
Lead-like ^(b)^	1	1
MDDR-like ^(b)^	1	2
WDI-like ^(b)^	0	2
Bioavailability score ^(a)^	0.55	0.55
Synthetic accessibility ^(a)^	3.17	2.48

^(a)^ Using the SwissADME web server; ^(b)^ using the PreADMET web server.

**Table 4:** Predictive ADMET properties of compounds **16** and **17**.

	16	17
Pharmacokinetic properties		
GI absorption ^(a)^	High	High
HIA (%) ^(b)^	97.88	100
Caco-2 cell (nm·s^−1^) ^(b)^	57.50	56.90
BBB permeant ^(a)^	Yes	Yes
Pgp substrate ^(a)^	No	No
PPB (%) ^(b)^	100	100
CYP1A2 Inhibitor ^(a)^	Yes	Yes
CYP2C19 Inhibitor ^(a)^	Yes	Yes
CYP2C9 Inhibitor ^(a)^	Yes	Yes
CYP2D6 Inhibitor ^(a)^	No	No
CYP3A4 Inhibitor ^(a)^	No	No
CYP2C19 Inhibitor ^(b)^	Yes	Yes
CYP2C9 Inhibitor ^(b)^	Yes	Yes
CYP2D6 Inhibitor ^(b)^	No	No
CYP3A4 Inhibitor ^(b)^	No	No
Carcinogenic activity mouse ^(b)^	Negative	Positive
Carcinogenic activity rat ^(b)^	Negative	Negative
hERG-inhibitor ^(b)^	Medium risk	Medium risk
Log K_p_ ^(a)^	−4.85 cm/s	−4.24 cm/s

^(a)^ Using the SwissADME web server; ^(b)^ u thesing PreADMET web server.

## Data Availability

Data is contained within the article.

## Supplementary Materials

The following supporting information can be downloaded at: https://www.mdpi.com/article/10.3390/ph16101354/s1, Figure S1. ^1^H and ^13^C NMR of compound **3**; Figure S2. ^1^H and ^13^C NMR of compound **4**; Figure S3. ^1^H and ^13^C NMR of compound **5**; Figure S4. ^1^H and ^13^C NMR of compound **6**; Figure S5. ^1^H and ^13^C NMR of compound **7**; Figure S6. ^1^H and ^13^C NMR of compound **8**; Figure S7. ^1^H and ^13^C NMR of compound **9**; Figure S8. ^1^H and ^13^C NMR of compound **10**; Figure S9. ^1^H and ^13^C NMR of compound **11**; Figure S10. ^1^H and ^13^C NMR of compound **12**; Figure S11. ^1^H and ^13^C NMR of compound **13**; Figure S121H and ^13^C NMR of compound **14**; Figure S13. ^1^H and ^13^C NMR of compound **15**; Figure S14. ^1^H and ^13^C NMR of compound **16**; Figure S15. ^1^H and ^13^C NMR of compound **17**; Figure S16. ^1^H and ^13^C NMR of compound **18**; Figure S17. ^1^H and ^13^C NMR of compound **19**; Figure S18. ^1^H and ^13^C NMR of compound **20**; Figure S19. ^1^H and ^13^C NMR of compound **21**; Figure S20. ^1^H and ^13^C NMR of compound **22**; Figure S211H and ^13^C NMR of compound **23**; Figure S22. ^1^H and ^13^C NMR of compound **24**; Figure S23. ^1^H and ^13^C NMR of compound **26**; Figure S24. ^1^H and ^13^C NMR of compound **27**; Figure S25. ^1^H and ^13^C NMR of compound **28**; Figure S26. ^1^H and ^13^C NMR of compound **29**; Figure S27. ^1^H and ^13^C NMR of compound **30**; Figure S28. ^1^H and ^13^C NMR of compound **31**; Figure S29. ^1^H and ^13^C NMR of compound **32**; Figure S30. ^1^H and ^13^C NMR of compound **33**; Figure S31. ^1^H and ^13^C NMR of compound **34**; Figure S32. ^1^H and ^13^C NMR of compound **35**; Figure S33. ^1^H and ^13^C NMR of compound **36**; Figure S34. ^1^H and ^13^C NMR of compound **38**; Figure S35. ^1^H and ^13^C NMR of compound **40**; Figure S36. HRMS of compound **4**; Figure S37. HRMS of compound **7**; Figure S38. HRMS of compound **10**; Figure S39. HRMS of compound **12**; Figure S40. HRMS of compound **14**; Figure S41. HRMS of compound **26**; Figure S42. HRMS of compound **27**; Figure S43. HRMS of compound **29**; Figure S44. HRMS of compound **30**; Figure S45. HRMS of compound **31**; Figure S46. HRMS of compound **33**; Figure S47. HRMS of compound **34**; Figure S48. HRMS of compound **35**; Figure S49. HRMS of compound **36**; Figure S50. HRMS of compound **38**; Figure S51. HRMS of compound **40**; Figure S52. HPLC chromatograms of compound **3**; Figure S53. HPLC chromatograms of compound **4**; Figure S54. HPLC chromatograms of compound **5**; Figure S55. HPLC chromatograms of compound **6**; Figure S56. HPLC chromatograms of compound **7**; Figure S57. HPLC chromatograms of compound **8**; Figure S58. HPLC chromatograms of compound **9**; Figure S59. HPLC chromatograms of compound **10**; Figure S60. HPLC chromatograms of compound **11**; Figure S61. HPLC chromatograms of compound **12**; Figure S62. HPLC chromatograms of compound **13**; Figure S63. HPLC chromatograms of compound **14**; Figure S64. HPLC chromatograms of compound **15**; Figure S65. HPLC chromatograms of compound **16**; Figure S66. HPLC chromatograms of compound **17**; Figure S67. HPLC chromatograms of compound **18**; Figure S68. HPLC chromatograms of compound **19**; Figure S69. HPLC chromatograms of compound **20**; Figure S70. HPLC chromatograms of compound **21**; Figure S71. HPLC chromatograms of compound **22**; Figure S72. HPLC chromatograms of compound **23**; Figure S73. HPLC chromatograms of compound **24**; Figure S74. HPLC chromatograms of compound **26**; Figure S75. HPLC chromatograms of compound **27**; Figure S76. HPLC chromatograms of compound **28**; Figure S77. HPLC chromatograms of compound **29**; Figure S78. HPLC chromatograms of compound **30**; Figure S79. HPLC chromatograms of compound **31**; Figure S80. HPLC chromatograms of compound **32**; Figure S81. HPLC chromatograms of compound **33**; Figure S82. HPLC chromatograms of compound **34**; Figure S83. HPLC chromatograms of compound **35**; Figure S84. HPLC chromatograms of compound **36**; Figure S85. HPLC chromatograms of compound **38**; Figure S86. HPLC chromatograms of compound **40**.
