# Supplementary material for: Structure–Activity Relationship Studies of Chalcones and Diarylpentanoids with Antitumor Activity: Potency and Selectivity Optimization
Source: Pharmaceuticals (Basel). 2023 Sep 25;16(10):1354. doi: 10.3390/ph16101354 (PMC10610188; doi:10.3390/ph16101354)
Supplement: Supplementary file 1 [file pharmaceuticals-16-01354-s001.zip › pharmaceuticals-2590543-supplementary.pdf]

pharmaceuticals

Supporting Information

Structure-Activity Relationship Studies of Chalcones and Diarylpentanoids with Antitumor Activity: Potency and Selectivity Optimization

Joana Moreira [1,2], Joana B. Loureiro [3], Danilo Correia [1], Andreia Palmeira [1,2], Madalena M. Pinto [1,2], Lucília Saraiva [3,*], and Honorina Cidade [1,2,*]

pharmaceuticals

Table of Contents

List of Figures 3

Experimental description of compounds of group A1 (3, 5, 6, 8-11, and 13-16) 5

Experimental description of compounds of group A2 (1 and 25) 6

Experimental description of compounds of group A3 (17-24) 7

Experimental description of compounds of group B2 (28) 8

Experimental description of compounds of group C1 (32) 9

Experimental description of compounds of group C3 (37 and 39) 9

NMR spectra 10

HRMS spectra 45

HPLC chromatograms 61

List of Figures

Figure S1. ^{1}H and ^{13}C NMR of compound **3**. 10
Figure S2. ^{1}H and ^{13}C NMR of compound **4**. 11
Figure S3. ^{1}H and ^{13}C NMR of compound **5**. 12
Figure S4. ^{1}H and ^{13}C NMR of compound **6**....13
Figure S5. ^{1}H and ^{13}C NMR of compound **7**. 14
Figure S6. ^{1}H and ^{13}C NMR of compound **8**. 15
Figure S7. ^{1}H and ^{13}C NMR of compound **9**. 16
Figure S8. ^{1}H and ^{13}C NMR of compound **10**. 17
Figure S9. ^{1}H and ^{13}C NMR of compound **11**. 18
Figure S10. ^{1}H and ^{13}C NMR of compound **12**. 19
Figure S11. ^{1}H and ^{13}C NMR of compound **13**. 20
Figure S12. ^{1}H and ^{13}C NMR of compound **14**. 21
Figure S13. ^{1}H and ^{13}C NMR of compound **15**. 22
Figure S14. ^{1}H and ^{13}C NMR of compound **16**. 23
Figure S15. ^{1}H and ^{13}C NMR of compound **17**. 24
Figure S16. ^{1}H and ^{13}C NMR of compound **18**. 25
Figure S17. ^{1}H and ^{13}C NMR of compound **19**. 26
Figure S18. ^{1}H and ^{13}C NMR of compound **20**. 27
Figure S19. ^{1}H and ^{13}C NMR of compound **21**. 28
Figure S20. ^{1}H and ^{13}C NMR of compound **22** 29
Figure S21. ^{1}H and ^{13}C NMR of compound **23**. 30
Figure S22. ^{1}H and ^{13}C NMR of compound **24**. 31
Figure S23. ^{1}H and ^{13}C NMR of compound **26**. 32
Figure S24. ^{1}H and ^{13}C NMR of compound **27**. 33
Figure S25. ^{1}H and ^{13}C NMR of compound **28**....34
Figure S26. ^{1}H and ^{13}C NMR of compound **29**....35
Figure S27. ^{1}H and ^{13}C NMR of compound **30**....36
Figure S28. ^{1}H and ^{13}C NMR of compound **31**. 37
Figure S29. ^{1}H and ^{13}C NMR of compound **32**. 38
Figure S30. ^{1}H and ^{13}C NMR of compound **33**. 39
Figure S31. ^{1}H and ^{13}C NMR of compound **34**. 40
Figure S32. ^{1}H and ^{13}C NMR of compound **35**. 41
Figure S33. ^{1}H and ^{13}C NMR of compound **36**. 42
Figure S34. ^{1}H and ^{13}C NMR of compound **38**. 43
Figure S35. ^{1}H and ^{13}C NMR of compound **40**. 44
Figure S36. HRMS of compound **4**....45
Figure S37. HRMS of compound **7**....46
Figure S38. HRMS of compound **10**....47
Figure S39. HRMS of compound **12**....48
Figure S40. HRMS of compound **14**....49
Figure S41. HRMS of compound **26**....50
Figure S42. HRMS of compound **27**....51
Figure S43. HRMS of compound **29**....52

Figure S44. HRMS of compound **30**......53
Figure S45. HRMS of compound **31**......54
Figure S46. HRMS of compound **33**......55
Figure S47. HRMS of compound **34**......56
Figure S48. HRMS of compound **35**......57
Figure S49. HRMS of compound **36**......58
Figure S50. HRMS of compound **38**......59
Figure S51. HRMS of compound **40**......60
Figure S52. HPLC chromatograms of compound **3**......61
Figure S53. HPLC chromatograms of compound **4**......62
Figure S54. HPLC chromatograms of compound **5**......63
Figure S55. HPLC chromatograms of compound **6**......64
Figure S56. HPLC chromatograms of compound **7**......65
Figure S57. HPLC chromatograms of compound **8**......66
Figure S58. HPLC chromatograms of compound **9**......67
Figure S59. HPLC chromatograms of compound **10**......68
Figure S60. HPLC chromatograms of compound **11**......69
Figure S61. HPLC chromatograms of compound **12**......70
Figure S62. HPLC chromatograms of compound **13**......71
Figure S63. HPLC chromatograms of compound **14**......72
Figure S64. HPLC chromatograms of compound **15**......73
Figure S65. HPLC chromatograms of compound **16**......74
Figure S66. HPLC chromatograms of compound **17**......75
Figure S67. HPLC chromatograms of compound **18**......76
Figure S68. HPLC chromatograms of compound **19**......77
Figure S69. HPLC chromatograms of compound **20**......78
Figure S70. HPLC chromatograms of compound **21**......79
Figure S71. HPLC chromatograms of compound **22**......80
Figure S72. HPLC chromatograms of compound **23**......81
Figure S73. HPLC chromatograms of compound **24**......82
Figure S74. HPLC chromatograms of compound **26**......83
Figure S75. HPLC chromatograms of compound **27**......84
Figure S76. HPLC chromatograms of compound **28**......85
Figure S77. HPLC chromatograms of compound **29**......86
Figure S78. HPLC chromatograms of compound **30**......87
Figure S79. HPLC chromatograms of compound **31**......88
Figure S80. HPLC chromatograms of compound **32**......89
Figure S81. HPLC chromatograms of compound **33**......90
Figure S82. HPLC chromatograms of compound **34**......91
Figure S83. HPLC chromatograms of compound **35**......92
Figure S84. HPLC chromatograms of compound **36**......93
Figure S85. HPLC chromatograms of compound **38**......94
Figure S86. HPLC chromatograms of compound **40**......95

Experimental description of compounds of group A1 (3, 5, 6, 8-11, and 13-16)

(*E*)-2-(3,4,5-trimethoxybenzylidene)-2,3-dihydro-1*H*-inden-1-one (**3**): Purified by crystallization from methanol. Yield: 80% as white solid; 99.9% purity; **mp** 160-163 °C (methanol); **^{1}H NMR** ($CDCl_3$, 300.13 MHz) δ: 7.91 (d, *J* = 7.7 Hz, 1H, H-7), 7.62 (td, *J* = 7.2; 1.3 Hz, 1H, H-5), 7.59 (sl, 1H, H-1′′), 7.57 (brd, *J* = 6.6 Hz, 1H, H-4), 7.43 (brt, *J* = 7.3 Hz, 1H, H-6), 6.91 (s, 2H, H-2′, -6′), 4.04 (sl, 2H, H-3), 3.94 (s, 6H, 3′-, 5′-OCH_3), 3.92 (s, 3H, 4′-OCH_3) ppm; **^{13}C NMR** ($CDCl_3$, 75.47 MHz) *δ:* 194.3 (C=O), 153.5 (C-3′, -5′), 149.5 (C-9), 139.9 (C-4′), 138.2 (C-8), 134.7 (C-1′′), 134.3 (C-5), 133.9 (C-2), 127.9 (C-6), 126.3 (C-4), 124.6 (C-7), 131.0 (C-1′), 108.3 (C-2′, -6′), 61.2 (4′-OCH_3), 56.4 (3′-, 5′-OCH_3), 32.4 (C-3) ppm.

(*E*)-2-(3,4,5-trimethoxybenzylidene)-5,6-dimethoxy-2,3-dihydro-1*H*-inden-1-one (**5**): Purified by crystallization from methanol. Yield: 85% as white solid; 99.7% purity; **mp** 156-159 °C (methanol); **^{1}H NMR** ($CDCl_3$, 300.13 MHz) δ: 7.50 (sl, 1H, H-1′′), 7.33 (s, 1H, H-7), 7.00 (s, 1H, H-4), 6.88 (s, 2H, H-2′, -6′), 3.99 (s, 3H, 6-OCH_3), 3.96 (sl, 2H, H-3), 3.94 (s, 3H, 4′-OCH_3), 3.93 (s, 6H, 3′-, 5′-OCH_3), 3.90 (s, 3H, 5-OCH_3) ppm; **^{13}C NMR** ($CDCl_3$, 75.47 MHz) *δ:* 193.2 (C=O), 155.5 (C-6), 153.5 (C-3′, -5′), 149.8 (C-5), 144.7 (C-9), 139.6 (C-4′), 134.6 (C-2), 132.7 (C-1′′), 131.2 (C-8, -1′), 108.0 (C-2′, -6′), 107.3 (C-4), 105.2 (C-7), 61.1 (4′-OCH_3), 56.4 (6-OCH_3), 56.3 (5-, 3′-, 5′-OCH_3), 32.1 (C-3) ppm.

(*E*)-2-(4-chlorobenzylidene)-2,3-dihydro-1*H*-inden-1-one (**6**): Purified by crystallization from methanol. Yield: 71% as yellow solid; 99.8% purity; **mp** 179-181 °C (methanol); **^{1}H NMR** ($CDCl_3$, 300.13 MHz) *δ*: 7.91 (dd, *J* = 7.7; 1.9 Hz, 1H, H-7), 7.66-7.59 (m, 4H, H-5, -2′, -6′, H-1′′), 7.56 (dt, *J* = 7.6; 1.2 Hz, 1H, H-4), 7.45-7.42 (m, 3H, H-3′, -5′, -6), 4.02 (d, *J* = 2.1 Hz, 2H, H-3) ppm; **^{13}C NMR** ($CDCl_3$, 75.47 MHz) *δ:* 194.3 (C=O), 149.6 (C-9), 138.0 (C-8), 135.8 (C-4′), 135.3 (C-2), 134.9 (C-5), 134.0 (C-1′′), 132.6 (C-1′′), 132.0 (C-2′, -6′), 129.4 (C-3′, -5′), 127.6 (C-6), 126.3 (C-4), 124.7 (C-7), 32.8 (C-3) ppm.

(*E*)-2-(4-chlorobenzylidene)-5,6-dimethoxy-2,3-dihydro-1*H*-inden-1-one (**8**): Purified by crystallization from methanol. Yield: 47% as light-yellow solid; 99.8% purity; **mp** 188-190 °C (methanol); **^{1}H NMR** ($CDCl_3$, 300.13 MHz) *δ*: 7.58 (d, *J* = 7.7 Hz, 2H, H-2′, -6′), 7.53 (sl, 1H, H-1′′), 7.41 (d, *J* = 7.6 Hz, 2H, H-3′, -5′), 7.33 (s, 1H, H-7), 6.98 (s, 1H, H-4), 4.00 (s, 3H, 5-OCH_3), 3.93 (sl, 2H, H-3), 3.95 (s, 3H, 6-OCH_3) ppm; **^{13}C NMR** ($CDCl_3$, 75.47 MHz) δ: 193.0 (C=O), 155.7 (C-5), 149.9 (C-6), 144.9 (C-9), 136.0 (C-2), 135.1 (C-4′), 134.2 (C-1′), 131.7 (C-2′, -6′), 131.1 (C-1′′), 129.3 (C-3′, -5′), 124.6 (C-9), 107.3 (C-5), 105.2 (C-7), 56.5 (5-, 6- OCH_3), 32.2 (C-3) ppm.

(*E*)-2-(3,4,5-trimethoxybenzylidene)-3,4-dihydronaphthalen-1(2*H*)-one (9): Purified by crystallization from methanol. Yield: 76% as light-yellow solid; 100% purity; **mp** 116-118 °C (methanol); **^{1}H NMR** ($CDCl_3$, 300.13 MHz) *δ*: 8.12 (dd, *J* = 7.8; 2.0 Hz, 1H, H-8), 7.81 (sl, 1H, H-1′′), 7.49 (td, *J* = 7.5; 1.6 Hz, 1H, H-6), 7.37 (dd, *J* = 7.6; 1.3 Hz, H-7), 6.68 (s, 2H, H-2′, -6′), 3.90 (s, 3H, 4′-OCH_3), 3.89 (s, 6H, 3′-, 5′-OCH_3), 3.19 (td, *J*= 6.5; 1.8 Hz, 2H, H-3), 2.97 (t, *J* = 6.5 Hz, 2H, H-4) ppm; **^{13}C NMR** ($CDCl_3$, 75.47 MHz) *δ*: 187.9 (C=O), 153.2 (C-3′, -5′), 143.2 (C-10), 138.7 (C-4′), 137.0 (C-1′′), 135.0 (C-2), 133.4 (C-6), 133.6 (C-9), 131.6 (C-1′), 107.4 (C-2′, -6′), 128.3 (C-5), 128.2 (C-8), 127.2 (C-7), 61.1 (4′-OCH_3), 56.3 (3′, 5′-OCH_3), 29.0 (C-4), 27.5 (C-3) ppm.

(*E*)-3-(3,4,5-trimethoxybenzylidene)chroman-4-one (11): Purified by HPLC (column ACE - C18 (150 × 4.6 mm I.D., particle size 5 μm), acetonitrile : water (70:30)). Yield: 30% as light-yellow solid; 99.7% purity; **mp** 109-111 °C (acetonitrile); **^{1}H NMR** ($CDCl_3$, 300.13 MHz) *δ*: 8.02 (dd, *J* = 7.9; 1.7 Hz, 1H, H-8), 7.81 (sl, 1H, H-1′′), 7.53-7.43 (m, 1H, H-6), 7.11-7.04 (m, 1H, H-7), 6.97 (dd, *J* = 8.3; 1.1 Hz, 1H, H-5), 6.53 (s, 2H, H-2′, -6′), 5.39 (d, *J* = 1.9 Hz, 2H, H-4), 3.90 (s, 3H, 4′-OCH_3), 3.89 (s, 6H, 3′-, 5′-OCH_3) ppm; **^{13}C NMR** ($CDCl_3$. 75.47 MHz) *δ*: 182.2 (C=O), 161.2 (C-10), 153.4 (C-3′, -5′), 139.5 (C-4′), 137.8 (C-1′′), 136.0 (C-6), 130.4 (C-2), 130.0

(C-1′), 128.1 (C-8), 122.1 (C-7), 121.5 (C-9), 107.6 (C-2′, -6′), 118.0 (C-5), 61.2 (4′-OCH_3). 56.4 (3′-, 5′-OCH_3), 67.8 (C-3) ppm.

(*E*)-2-(4-chlorobenzylidene)-3,4-dihydronaphthalen-1(2*H*)-one (**13**): Purified by crystallization from methanol. Yield: 59% as yellow solid; 99.9% purity; **mp** 134-136 °C (methanol); **^{1}H NMR** ($CDCl_3$, 300.13 MHz) δ: 8.13 (dd, *J* = 7.8; 2.0 Hz, 1H, H-8), 7.80 (t, *J* = 1.8 Hz, 1H, H-1′′), 7.50 (td, *J* = 7.5; 2.5 Hz, 1H, H-6), 7.42-7.35 (m, 5H, H-2′, -3′, -5′, -6′, -7), 7.25 (dd, *J* = 7.5; 2.0 Hz, H-5), 3.13-3.08 (m, 2H, H-3), 2.95 (t, *J* = 6.4 Hz, 2H, H-4) ppm; **^{13}C NMR** ($CDCl_3$, 75.47 MHz) δ: 187.8 (C=O), 143.3 (C-10), 136.0 (C-2), 135.4 (C-1′′), 134.6 (C-4′), 134.4 (C-1′), 133.6 (C-6), 133.5 (C-9), 131.3 (C-2′,-6′), 128.9 (C-3′, -5′), 128.4 (C-8), 128.3 (C-5), 127.2 (C-7), 28.9 (C-4), 27.3 (C-3) ppm.

(*E*)-3-(4-chlorobenzylidene)chroman-4-one (**15**): Purified by TLC (n-hexane: ethyl acetate, 7:3); Yield: 59% as light-yellow solid; 99.9% purity; **mp** 128-129 °C (methanol); **^{1}H NMR** ($CDCl_3$, 300.13 MHz) δ: 8.02 (dd, *J* = 7.8; 2.0 Hz, H-8), 7.81 (sl, 1H, H-1′′), 7.50 (td, *J* = 7.7; 2.4 Hz, H-6), 7.43 (d, *J* = 8.5 Hz, 2H, H-3′, -5′), 7.25 (d, *J* = 8.4 Hz, 2H, H-2′, -6′), 7.08 (t, *J* = 7.5 Hz, 1H, H-7), 6.97 (d, *J* = 8.3 Hz, 1H, H-5), 5.31 (d, *J* = 1.9 Hz, 2H, H-3) ppm; **^{13}C NMR** ($CDCl_3$, 75.47 MHz) *δ:* 182.1 (C=O), 161.1 (C-10), 136.2 (C-6, -1′′), 135.7 (C-4′), 132.9 (C-1′), 131.5 (C-2′), 131.3 (C-2′, -6′), 129.2 (C-3′, -5′), 128.1 (C-8), 122.2 (C-7), 122.0 (C-9), 118.1 (C-5), 67.1 (C-3) ppm.

(*Z*)-3-(4-chlorobenzylidene)thiochroman-4-one (**16**): Purified by crystallization from methanol. Yield: 23% as yellow solid; 99.9% purity; **mp** 139-141 °C (methanol); **^{1}H NMR** ($CDCl_3$, 300.13 MHz) δ: 8.19 (dd, *J* = 7.9; 2.0 Hz, 1H, H-8), 7.70 (s, 1H, H-1′′), 7.44-7.38 (m, 3H, H-3′, -5′, -6), 7.35-7.32 (m, 2H, H-2′, -6′), 7.29 (dd, J = 8.3; 1.4 Hz, 1H, H-5), 7.25 (dd, J =7.1; 1.9 Hz, 1H, H-7), 4.07 (d, J = 1.2 Hz, 2H, H-3) ppm; **^{13}C NMR** ($CDCl_3$, 75.47 MHz) δ: 185.8 (C=O), 141.1 (C-10), 136.3 (C-1′′), 135.1 (C-1′), 133.6 (C-6), 133.3 (C-2, -4′), 132.4 (C-9), 130.9 (C-2′, -6′), 130.5 (C-8), 129.2 (C-3′, -5′), 128.0 (C-5), 126.0 (C-7), 29.3 (C-3) ppm.

Experimental description of compounds of group A2 (1 and 25)

Synthesis of 1-(2-hydroxy-4-methoxy-3-propylphenyl) ethan-1-one (25)

To a solution of 1-(2,4-dihydroxy-3-propylphenyl)ethan-1-one (5g, 0.026 mol, 1 eq.) in anhydrous acetone (100 ml) was added potassium carbonate (10.8 g, 0.078 mol, 1.6 eq.). Dimethyl sulfate (3.90 ml) was added dropwise, and the mixture was allowed to stir for 2 h at reflux and under nitrogen atmosphere. The reaction was quenched by the addition of ice and acetone was evaporated. After, an aqueous solution of 1M HCl was added until pH 2-3 and the solution was extracted with ethyl acetate (3x100 ml). The organic phases were washed with brine (2x50 ml), dried over with anhydrous sodium sulfate, and purified by crystallization with ethyl acetate. A white solid (90% yield) corresponding to 1-(2-hydroxy-4-methoxy-3-propylphenyl)ethan-1-one (**25**) was obtained.

1-(2-hydroxy-4-methoxy-3-propylphenyl)ethan-1-one (**25**) **^{1}H NMR** ($CDCl_3$, 300.13 MHz) δ: 12.73 (s, 1H, 2-OH), 7.60 (d, *J* = 8.9 Hz, 1H, H-6), 6.45 (d, *J* = 8.9 Hz, 1H, H-5), 3.88 (s, 3H, 4-OCH_3), 2.62 (t, *J* = 6.0 Hz, 2H, H-1′), 2.57 (s, 3H, 1-CO$\underline{H_3}$), 1.56-1.46 (m, 2H, H-2′), 0.94 (t, *J* = 7.4 Hz, 3H, H-3′) ppm; **^{13}C NMR** ($CDCl_3$, 75.47 MHz) δ: 202.9 (1-$\underline{C}OCH_3$), 163.5 (C-2), 162.0 (C-4), 130.0 (C-6), 118.3 (C-1), 114.2 (C-3), 101.9 (C-5), 55.7 (4-OCH_3), 26.3 (1-CO$\underline{C}H_3$), 24.3 (C-1′), 21.9 (C-2′), 14.2 (C-3′).

Synthesis of (*E*)-3-(3,4,5-trimethoxy-chlorophenyl)-1-(2-hydroxy-4-methoxy-3-ropylphenyl)prop-2-en-1-one (1)

To a solution of 1-(2-hydroxy-4-methoxy-3-propylphenyl)ethan-1-one (**25**, 700 mg, 3.36 mmol, 1 eq.) in methanol was added an aqueous solution of 40% sodium hydroxide to pH 13–14. Then, a solution of 3,4,5-trimethoxybenzaldehyde (1.32 g, 6.72 mmol, 2 eq.) in methanol was slowly added to the reaction mixture. The reaction was refluxed for 18 h. After cooling, the reaction was poured onto crushed ice and the pH was adjusted to 6-8, with 5M HCl solution and extracted with ethyl acetate. The organic layer was rinsed with brine and water, dried over with anhydrous sodium sulfate, and evaporated under reduced pressure. The obtained residue was purified by flash column chromatography (SiO_2; dichloromethane, 100%). An orange solid (40% yield) corresponding to (*E*)-1-(2-hydroxy-4-methoxy-3-propylphenyl)-3-(3,4,5-trimethoxyphenyl)prop-2-en-1-one (**1**) was obtained.

(*E*)-1-(2-hydroxy-4-methoxy-3-propylphenyl)-3-(3,4,5-trimethoxyphenyl)prop-2-en-1-one (**1**) **^{1}H NMR** ($CDCl_3$, 300.13 MHz) δ: 7.64 (d, *J* = 15.7 Hz, 1H, H-β), 7.62 (d, *J* = 8.6 Hz, 1H, H-6), 7.50 (d, *J* = 15.7 Hz, 1H, H-α), 6.84 (s, 2H, H-2′, -6′), 6.73 (d, *J* = 8.7 Hz, 1H, H-5), 5.46-5.40 (m, 2H, H-2′′′), 4.28 (d, *J* = 7.2 Hz, 2H, H-1′′′), 3.90 (s, 6H, 3′-, 5′-OCH_3), 3.89 (s, 3H, 4′-OCH_3), 3.88 (s, 3H, 4-OCH_3), 2.66 (t, *J* = 7.8 Hz, 3H,H-2′′), 1.59-1.54 (m, 2H, H-1′′), 1.62 (s, 3H, H-4′′′), 1.50 (s, 3H, H-5′′′), 1.00 (t, *J* = 7.4 Hz, 3H, H-3′′) ppm; **^{13}C NMR** ($CDCl_3$, 75.47 MHz) δ: 191.5 (C=O), 162.0 (C-4), 157.9 (C-2), 153.4 (C-3′, -5′), 142.8 (C-β), 140.1 (C-4′), 138.3 (C-4′′′), 131.0 (C-1′), 129.8 (C-6), 126.7 (C-1), 126.1 (C-α), 125.4 (C-3), 120.1 (C-2′′′), 106.4 (C-5), 105.6 (C-2′, -6′), 73.1 (C-1′′′), 61.1 (4′-OCH_3), 56.3 (3′-,5′-OCH_3), 55.8 (4-OCH_3), 26.1 (C-1′′), 25.8 (C-4′′′), 23.0 (C-2′′), 18.0 (C-5′′′), 14.7 (C-3′′) ppm.

Experimental description of compounds of group A3 (17-24)

An aqueous solution of 40% sodium hydroxide was added to a solution of appropriate ketone (100-200 mg, 0.99-1.72 mmol, 1 eq.) in methanol until pH 13-14. Then, a solution of appropriate benzaldehyde (0.4-2.03 g, 2.99 mmol, 3 eq.) in methanol was slowly added to the reaction mixture. The reaction was left at room temperature for 22 h-7 days and was monitored by TLC. After, crushed ice was added to the reaction mixture and neutralized with 5 M HCl solution. For the synthesis of the diarylpentanoids **17** and **19** after the addition of crushed ice, the solution was extracted with chloroform (3x50 mL), and the organic layers were collected, washed with water, dried over with anhydrous sodium sulfate and concentrated under reduced pressure. For the synthesis of compounds **18**, **20,** and **21-24** the obtained solid was filtrated, washed with water, dried, and purified.

(1*E*,4*E*)-1,5-bis(4-chlorophenyl)penta-1,4-dien-3-one (**17**): Purified by crystallization from methanol. Yield: 81% as yellow solid; 99.8% purity; **mp** 191-194 °C (methanol); **^{1}H NMR** ($CDCl_3$, 300.13 MHz) δ: 7.68 (d, *J* = 15.9 Hz, 2H, H-β), 7.54 (d, *J* = 8.5 Hz, 4H, H-2′, -6′), 7.39 (d, *J* = 8.5 Hz, 4H, H-3′, -5′), 7.03 (d, *J* = 15.9 Hz, 2H, H-α) ppm; **^{13}C NMR** ($CDCl_3$, 75.47 MHz) *δ:* 188.5 (C=O), 142.2 (C-β), 136.7 (C-4′), 133.3 (C-1′), 129.7 (C-2′, -6′), 129.4 (C-3′, -5′), 126.3 (C-α) ppm.

2,5-bis((*E*)-4-chlorobenzylidene)cyclopentan-1-one (**18**): Purified by TLC (n-hexane : ethyl acetate, 7:3). Yield: 90% as yellow solid; 95.4% purity; **mp** 185-188 °C (ethyl acetate); **^{1}H NMR** ($CDCl_3$, 300.13 MHz) δ: 7.54 (sl, 2H, H-1′′), 7.52 (d, *J* = 8.6 Hz, 4H, H-2′, -6′), 7.41 (d, *J* = 8.6 Hz, 4H, H-3′, -5′), 3.09 (sl, 4H, H-3, -4) ppm; **^{13}C NMR** ($CDCl_3$, 75.47 MHz) δ: 196.1 (C=O), 137.5 (C-2, -5), 135.6 (C-4′), 134.3 (C-1′), 132.8 (C-1′′), 132.0 (C-2′, -6′), 129.2 (C-3′, -5′), 26.6 (C-3, -4) ppm.

2,6-bis((*E*)-4-chlorobenzylidene)cyclohexan-1-one (**19**): Purified by crystallization from methanol. Yield: 33% as yellow solid; 96.1% purity; **mp** 144-146 °C (methanol); **^{1}H NMR** ($CDCl_3$, 300.13 MHz) δ: 7.72 (t, *J* = 2.1 Hz, 2H, H-1′′), 7.38 (sl, 8H, H-2′, -3′, -5′, -6′), 2.89 (td, *J* = 6.2; 2.0 Hz, 4H, H-3, -5), 1.34 (quintet, *J* = 6.0

Hz, 2H, H-4) ppm; **^{13}C NMR** ($CDCl_3$, 75.47 MHz) δ: 190.8 (C=O), 136.5 (C-2,-6), 135.9 (C-1′′), 134.8 (C-4′), 134.4 (C-1′), 131.7 (C-2′, -6′), 128.8 (C-3′, -5′), 29.0 (C-3, -5), 23.4 (C-4) ppm.

3,5-bis((*Z*-4-chlorobenzylidene)tetrahydro-4*H*-thiopyran-4-one (**20**): Purified by crystallization from methanol. Yield: 31% as yellow solid; 99.9% purity; **mp** 126-128 °C (methanol); **^{1}H NMR** ($CDCl_3$, 300.13 MHz) δ: 7.71 (s, 2H, H-1′′), 7.40 (d, *J* = 8.6 Hz, 4H, H-3′, -5′), 7.32 (d, *J* = 8.6 Hz, 4H, H-2′, -6′), 3.87 (sl, 4H, H-3, -5) ppm; **^{13}C NMR** ($CDCl_3$, 75.47 MHz) δ: 188.7 (C=O), 135.9 (C-1′′), 135.2 (C-4′), 134.3 (C-2, -6), 133.6 (C-1′), 131.0 (C-2′, -6′), 129.1 (C-3′, -5′), 30.7 (C-3, -5) ppm.

(1*E*,4*E*)-1,5-bis(3,4,5-trimethoxyphenyl)penta-1,4-dien-3-one (**21**): Purified by crystallization from methanol. Yield: 49% as yellow solid; 99.5% purity; **mp** 127-129 °C (methanol); **^{1}H NMR** ($CDCl_3$, 300.13 MHz) δ: 7.66 (d, *J* = 15.8 Hz, 2H, H-β), 6.98 (d, *J* = 15.8 Hz, 2H, H-α), 6.85 (s, 4H, H-2′, -6′), 3.93 (s, 6H, 3′-, 5′-OCH_3), 3.90 (s, 3H, 3′-, 5′-OCH_3) ppm**;** **^{13}C NMR** ($CDCl_3$, 75.47 MHz) δ: 188.6 (C=O), 153.6 (C-3′, -5′), 143.5 (C-β), 140.5 (C-4′), 130.4 (C-1′), 124.9 (C-α), 105.7 (C-2′, -6′), 61.2 (4′-OCH_3), 56.3 (3′-, 5′-OCH_3), ppm.

2,5-bis((*E*)-3,4,5-trimethoxybenzylidene)cyclopentan-1-one (**22**): Purified by crystallization from methanol. Yield: 90% as yellow solid; 99.5% purity; **mp** 203-205 °C (methanol); **^{1}H NMR** ($CDCl_3$, 300.13 MHz) δ: 7.52 (sl, 2H, H-1′′), 6.84 (s, 4H, H-2′, -6′), 3.91 (s, 6H, 3′-, 5′-OCH_3), 3.90 (s, 3H, 3′-, 5′-OCH_3) 3.14 (sl, 4H, H-3, -4) ppm; **^{13}C NMR** ($CDCl_3$, 75.47 MHz) δ: 196.0 (C=O), 153.4 (C-2′, -6′), 139.7 (C-4′), 136.2 (C-2, -5), 134.2 (C-1′′), 131.5 (C-1′), 61.1 (4′-OCH_3), 56.3 (3′-, 5′-OCH_3), 26.6 (C-3, -4) ppm.

2,6-bis((E)-3,4,5-trimethoxybenzylidene)cyclohexan-1-one (**23**): Purified by crystallization from methanol. Yield: 60% as yellow solid; 98.9% purity; **mp** 196-198 °C (methanol); **^{1}H NMR** ($CDCl_3$, 300.13 MHz) δ: 7.72 (sl, 2H, H-1′′), 6.71 (s, 4H, H-2′, -6′), 3.89 (s, 6H, 3′-, 5′-OCH_3), 3.88 (s, 3H, 4′-OCH_3), 2.95 (td, *J* = 6.2; 2.0 Hz, 4H, H-3, -5), 1.34 (quintet, *J* = 6.0 Hz, 2H, H-4) ppm; **^{13}C NMR** ($CDCl_3$, 75.47 MHz) δ: 190.1 (C=O), 153.1 (C-3′, -5′), 138.9 (C-4′), 137.3 (C-1′′), 135.5 (C-2, -6), 131.6 (C-1′), 108.0 (C-2′, -6′), 61.1 (4′-OCH_3), 56.3 (3′-, 5′-OCH_3), 28.6 (C-3, -5), 23.1 (C-4) ppm.

3,5-bis((*Z*)-3,4,5-trimethoxybenzylidene)tetrahydro-4*H*-thiopyran-4-one (**24**): Purified by crystallization from methanol. Yield: 55% as yellow solid; 96.7% purity; **mp** 219-222 °C (methanol); **^{1}H NMR** ($CDCl_3$, 300.13 MHz) δ: 7.71 (sl, 2H, H-1′′), 6.63 (s, 4H, H-2′, -6′), 3.89 (s, 3H, 4′-OCH_3), 3.88 (s, 6H, 3′-, 5′-OCH_3), 3.95 (sl, 4H, H-3, -5) ppm; **^{13}C NMR** ($CDCl_3$, 75.47 MHz) δ: 188.9 (C=O), 153.3 (C-3′, -5′), 139.1 (C-4′), 137.3 (C-1′′), 133.4 (C-2, -6), 130.7 (C-1′), 107.6 (C-2′, -6′), 61.1 (4′-OCH_3), 56.4 (3′-, 5′-OCH_3), 30.2 (C-3, -5) ppm.

Experimental description of compounds of group B2 (28)

(*E*)-6-(1-hydroxy-3-(3,4,5-trimethoxyphenyl)allyl)-3-methoxy-2-propylphenol (**28**): Purified by TLC (SiO_2; n-hexane: ethyl acetate, 9:1). Yield: 15% yellow gum; 99.8% purity; **mp** 219-222 °C (methanol); **^{1}H NMR** ($CDCl_3$, 300.13 MHz) δ: 6.96 (d, J = 8.3 Hz, 1H, H-6), 6.57 (s, 2H, H-2′, -6′), 6.46 (d, *J* = 9.0 Hz, 1H, H-5), 6.43 (d, *J* = 16.0 Hz, 1H, H-3′′′), 6.33-6.23 (m, 1H, H-2′′′), 5.01 (sl, 1H, 1′′′-OH), 3.85 (s, 6H, 3′-, 5′-OCH_3), 3.84 (s, 3H, 4′-OCH_3), 3.80 (s, 3H, 4-OCH_3), 3.50 (d, *J* = 6.4 Hz, 1H, H-1′′), 2.61 (t, *J* = 7.6 Hz, 2H, H-1′′), 1.59-1.48 (m, 2H, H-2′′), 0.96 (t, *J* = 7.4 Hz, 3H, H-3′′) ppm; **^{13}C NMR** ($CDCl_3$, 75.47 MHz) δ: 157.6 (C-4), 153.4 (C-3′, -5′), 153.1 (C-2), 137.7 (C-4′), 132.9 (C-1′), 131.3 (C-3′′′), 128.2 (C-2′′′), 127.5 (C-6), 117.9 (C-1), 117.6 (C-3), 103.3 (C-2′, -6′), 103.0 (C-5), 61.1 (4′-OCH_3), 56.2 (3′-, 5′-OCH_3), 55.7 (4-OCH_3), 34.4 (C-1′′′), 26.3 (C-1′′), 22.5 (C-2′′), 14.4 (C-3′′) ppm

Experimental description of compounds of group C1 (32)

(*E*)-3-(4-chlorophenyl)-1-(2-hydroxy-4-methoxy-3-propylphenyl)prop-2-en-1-one (**32**): Purified by TLC (SiO_2; n-hexane: dichloromethane, 5:5). Yield: 10% as orange solid; 99.2% purity; **mp** 109-112 °C (dichloromethane); **^{1}H NMR** ($CDCl_3$, 300.13 MHz) δ: 13.30 (s, 1H, 2-OH), 7.82 (d, *J* = 15.2 Hz, 1H, H-β), 7.78 (d, *J* = 8.7 Hz, 1H, H-6), 7.58 (dt, *J* = 8.6; 2.0 Hz, 2H, H-2′, -6′), 7.57 (d, *J* = 15.5 Hz, 1H, H-α), 7.40 (dt, *J* = 8.8; 2.0 Hz, 2H, H-3′, -5′), 6.50 (d, *J* = 9.0 Hz, 1H, H-5), 3.90 (s, 3H, 4-OCH_3), 2.66 (t, *J* = 7.7 Hz, 2H, H-1′′), 1.61-1.49 (m, 2H, H-2′′), 0.96 (t, *J* = 7.4 Hz, 3H, H-3′′) ppm; **^{13}C NMR** ($CDCl_3$, 75.47 MHz) δ: 192.1 (C=O), 163.9 (C-4), 163.5 (C-2), 142.7 (C-β), 136.6 (C-4′), 133.5 (C-1′), 129.8 (C-2′, -6′), 129.4 (C-3′, -5′), 129.2 (C-6), 121.3 (C-α), 118.8 (C-3), 114.5 (C-1), 102.2 (C-5), 56.9 (4-OCH_3), 24.6 (C-1′′), 22.0 (C-2′′), 14.4 (C-3′′) ppm.

Experimental description of compounds of group C3 (37 and 39)

Synthesis of 2-((3-methylbut-2-en-1-yl)oxy)benzaldehyde (37)

To a solution of 2-hydroxybenzaldehyde (200 mg, 171 μL, 1.64 mmol, 1 eq.) and tetrabutylammonium hydroxide 30-hydrate (2.62 g, 3.28 mmol, 2 eq.) in chloroform / toluene 10:7 (17 mL) was added 1-bromo-3-methylbut-2-ene (57.8 mg, 44.8 μL, 388 μmol, 1.5 eq.) and the reaction was allowed to proceed at room temperature for 1.5h under gentle stirring. The reaction mixture was then poured into an ice, toluene was evaporated, and the crude was extracted with chloroform (3x50mL). The combined organic layers were washed with brine solution, rinsed with water, dried over with anhydrous sodium sulfate, and evaporated under reduced pressure. The resulting crude was purified by TLC (SiO_2, dichloromethane : n-hexane, 6:4). An orange gum corresponding to 2-((3-methylbut-2-en-1-yl)oxy)benzaldehyde (**37**, 85% yield) was obtained.

Synthesis of 4-((3-methylbut-2-en-1-yl)oxy)benzaldehyde (39)

A mixture of 4-hydroxybenzaldehyde (500 mg, 4.09 mmol, 1 eq.), 1-bromo-3-methylbut-2-ene (976 mg, 757 μL, 6.55 mmol, 1.6 eq.) and anhydrous K_2CO_3 (1.3 g, 8.19 mmol, 2 eq.) in anhydrous acetone was left at reflux for 1.5 h and was monitored by TLC. After cooling, the solvent was removed under reduced pressure to afford the crude product. The solid obtained was dissolved in chloroform and the solution was extracted with water (3x50 mL). The organic layers were collected, washed with water, dried over with anhydrous sodium sulfate, and concentrated under reduced pressure. The crude material was purified by TLC (SiO_2, n-hexane : ethyl acetate, 8:2). A white gum (72% yield) corresponding to 4-((3-methylbut-2-en-1-yl)oxy)benzaldehyde (**39**) was obtained.

NMR spectra

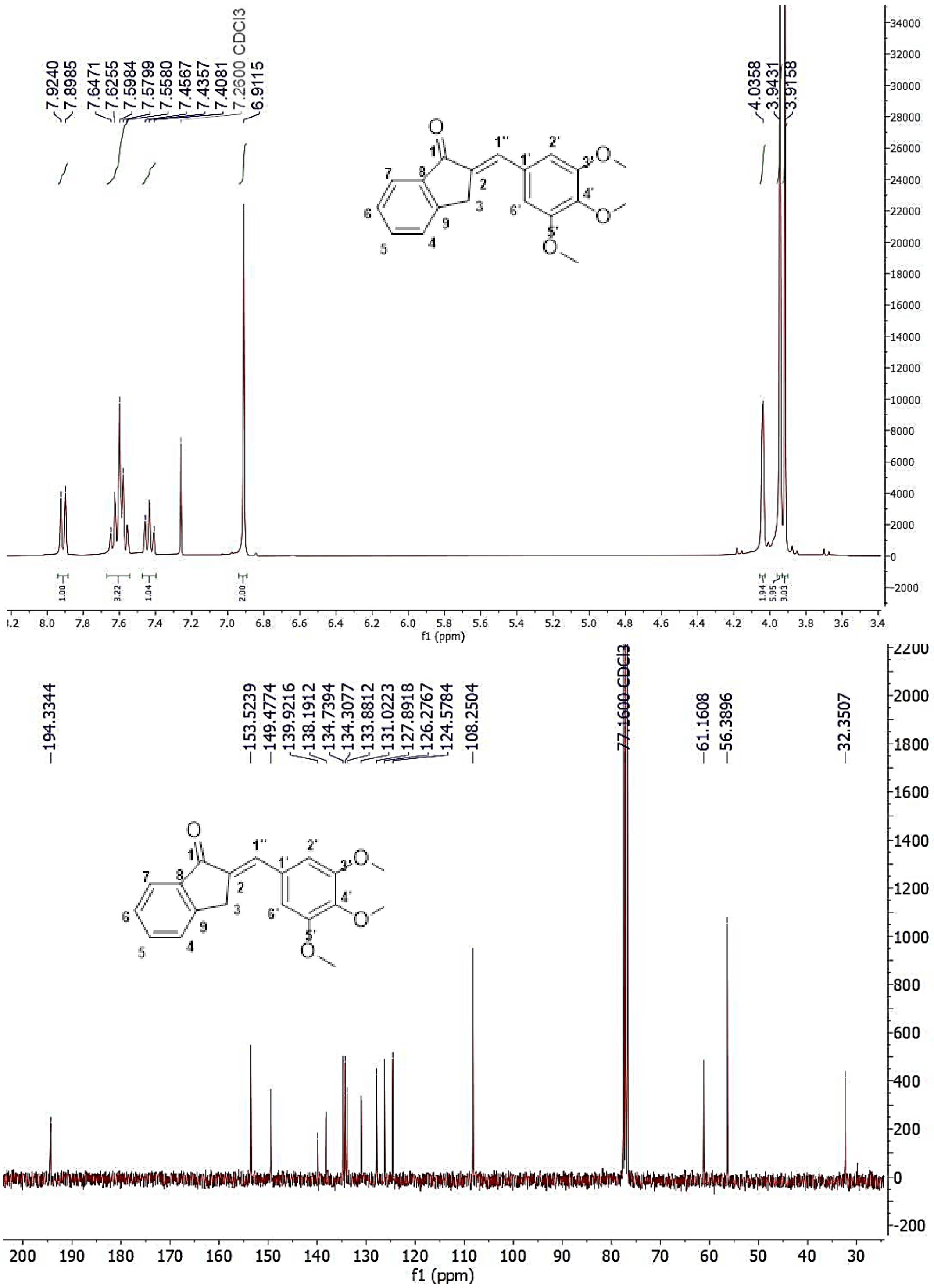

Figure S1. ^{1}H and ^{13}C NMR of compound **3**.

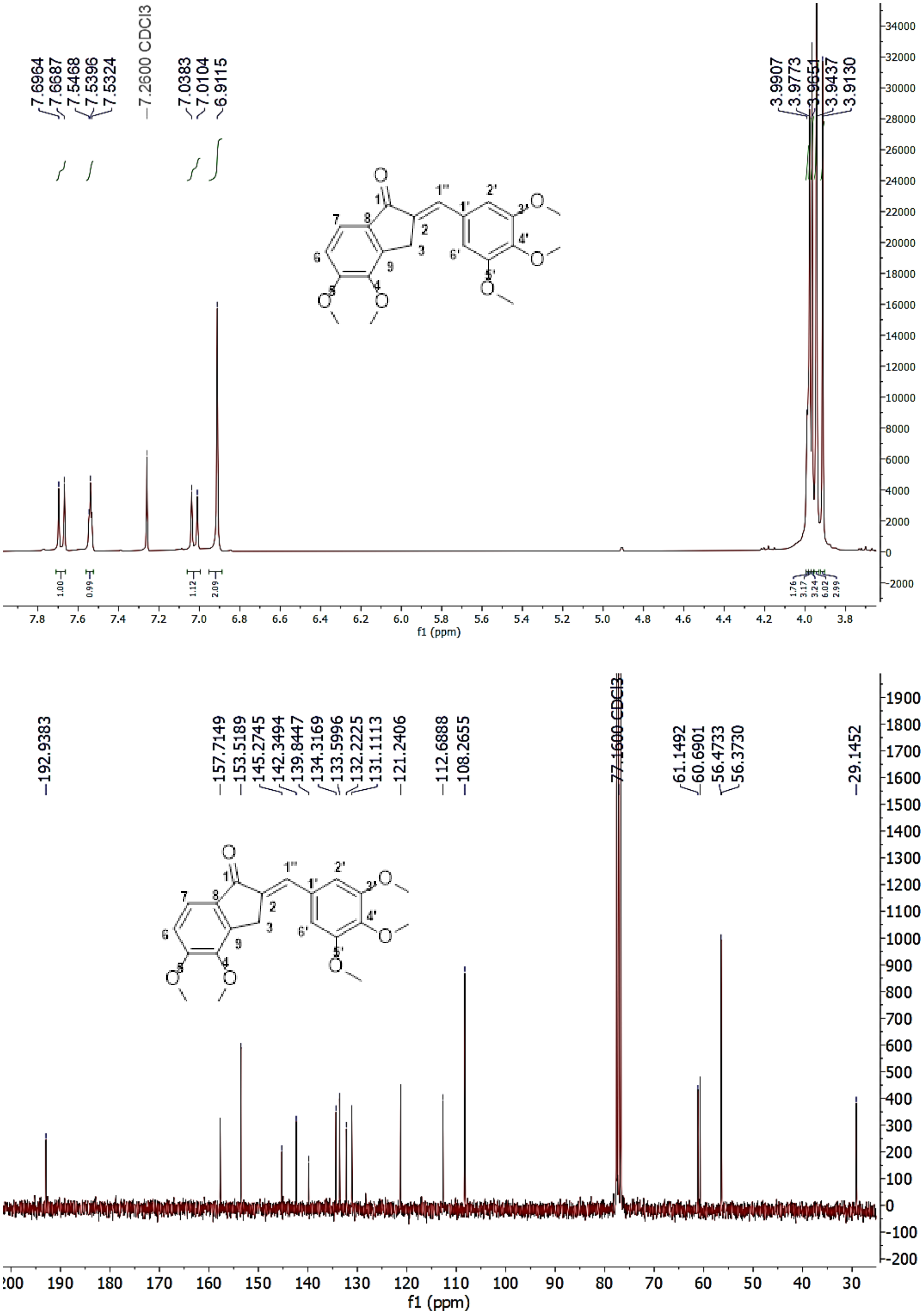

Figure S2. ^{1}H and ^{13}C NMR of compound **4**.

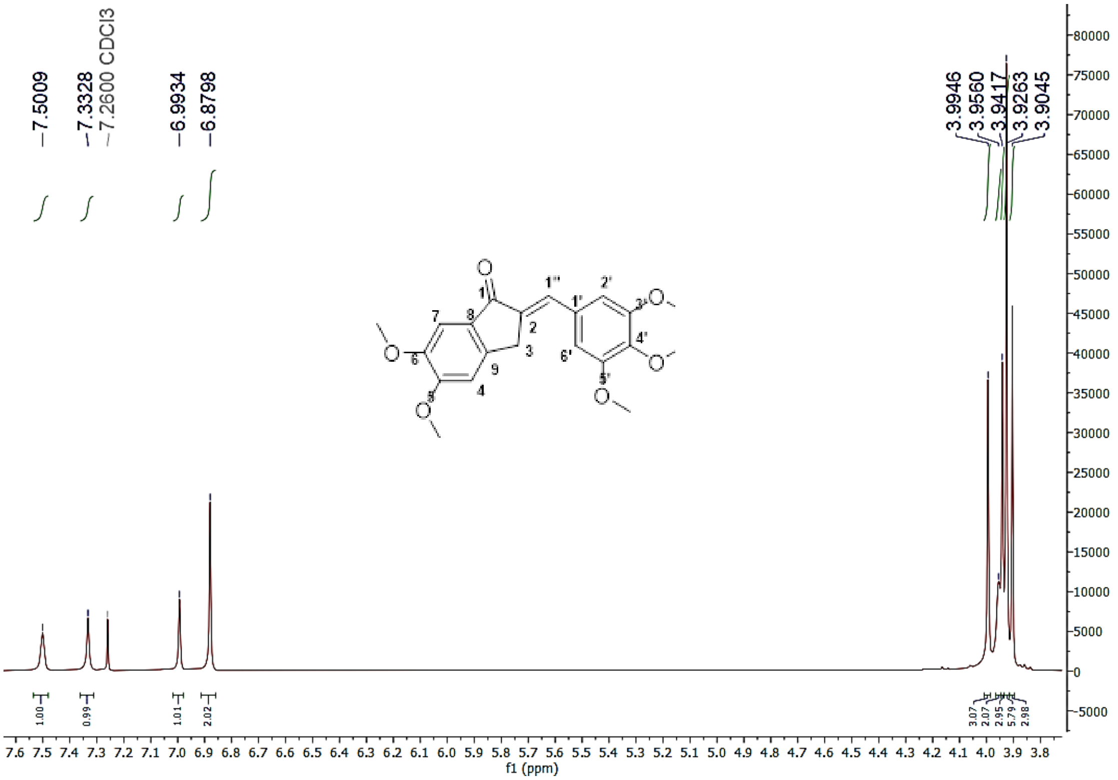

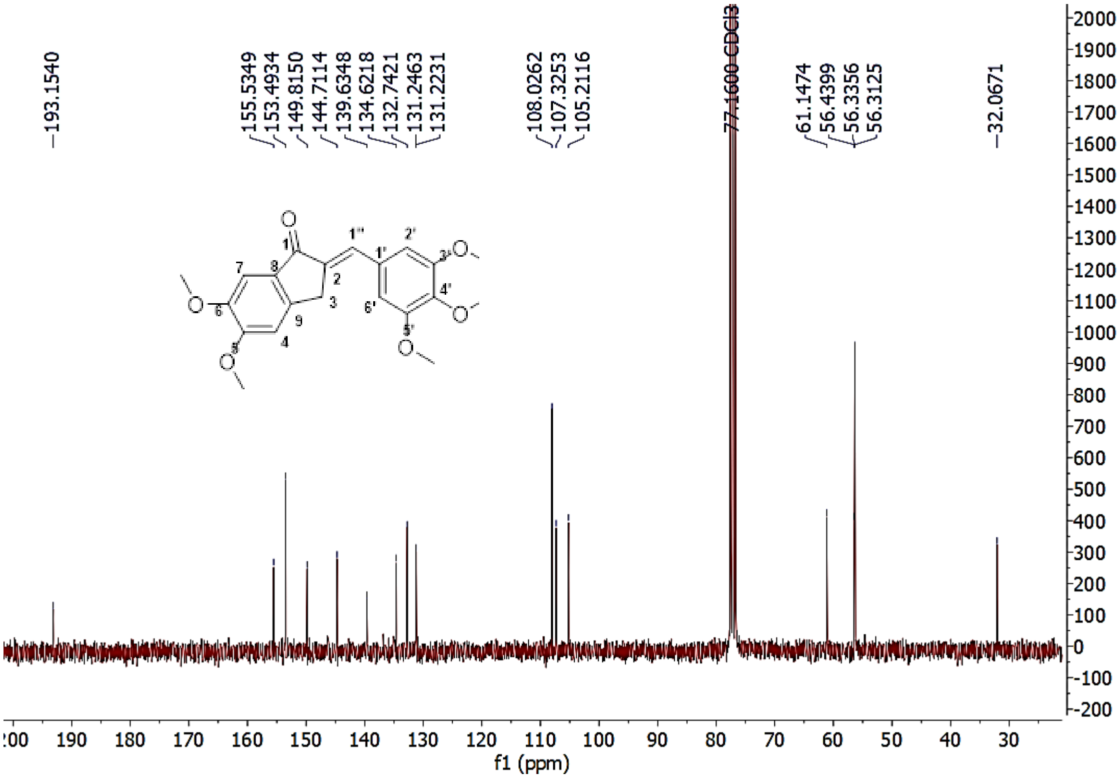

Figure S3. ^{1}H and ^{13}C NMR of compound **5**.

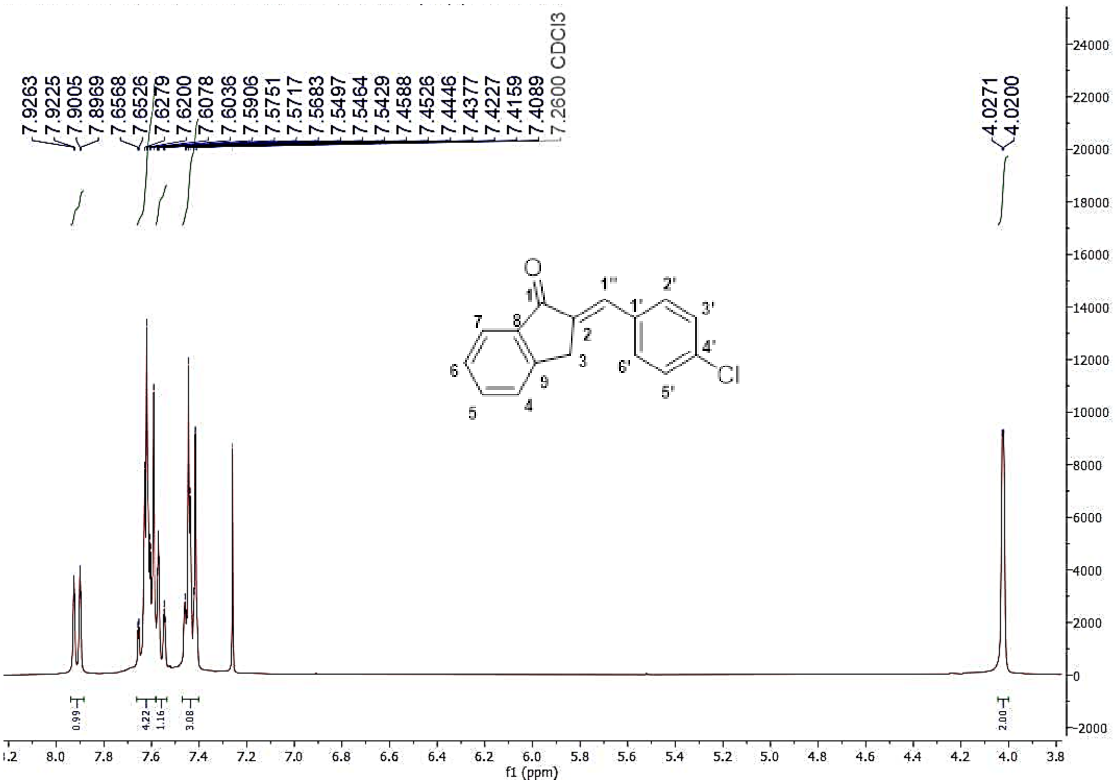

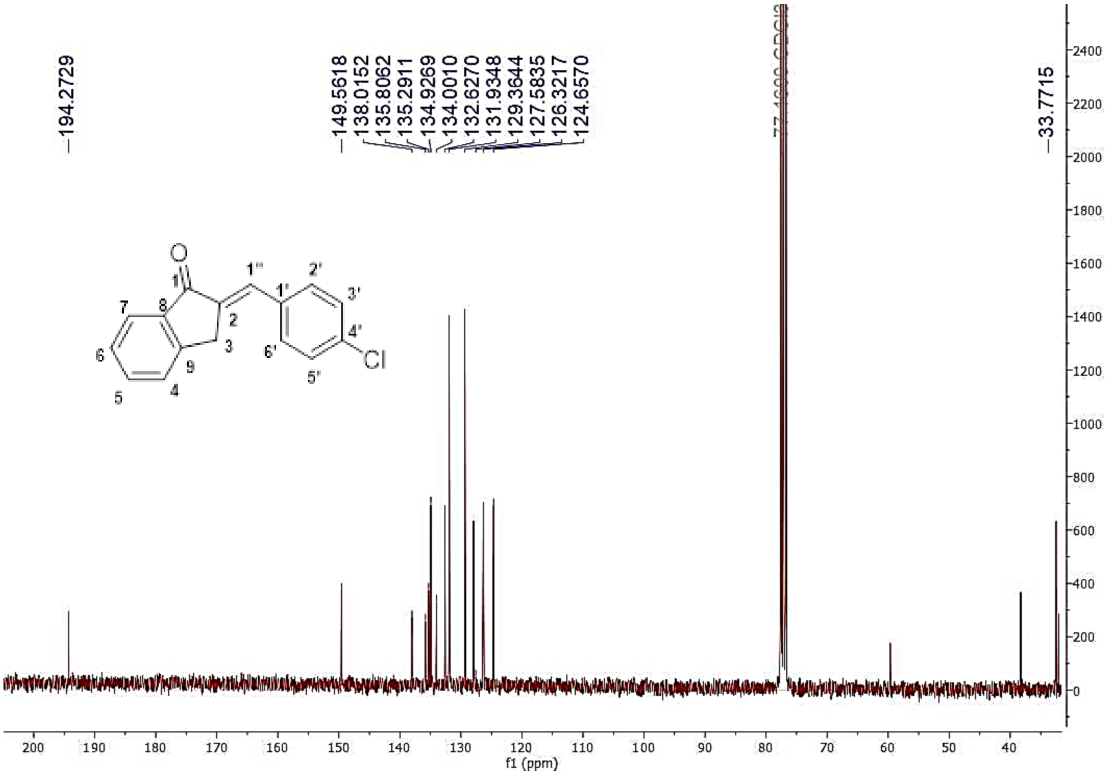

Figure S4. ^{1}H and ^{13}C NMR of compound **6**.

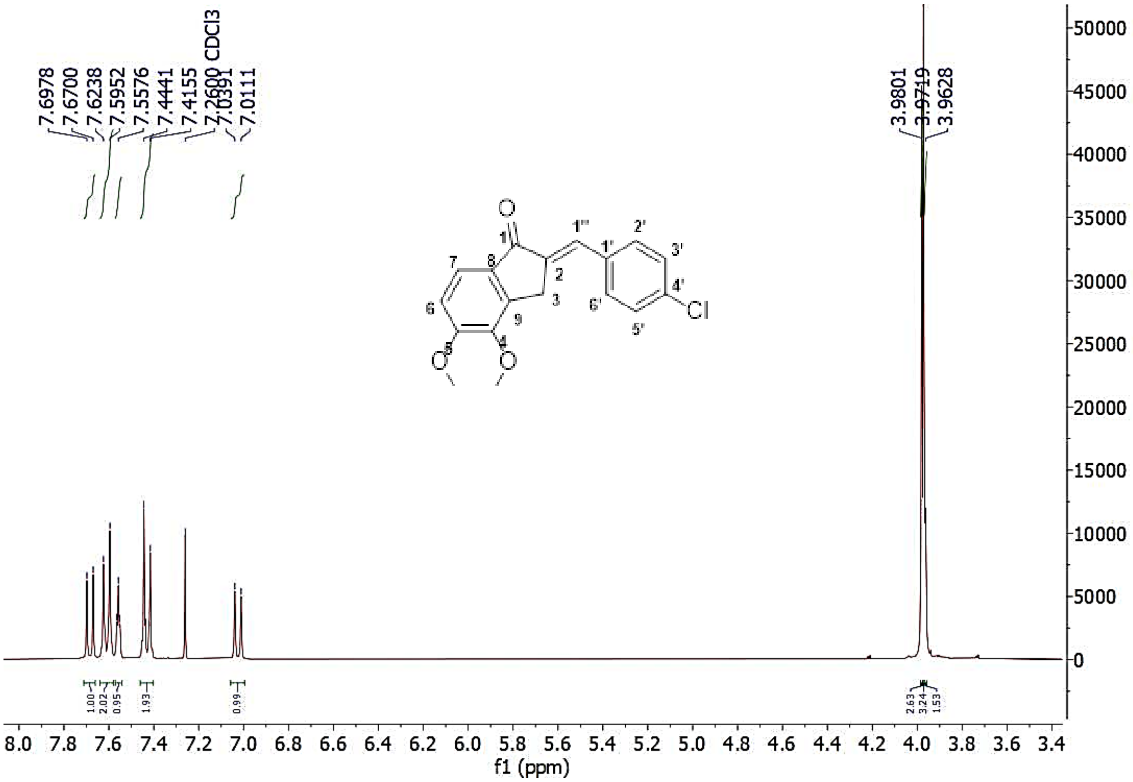

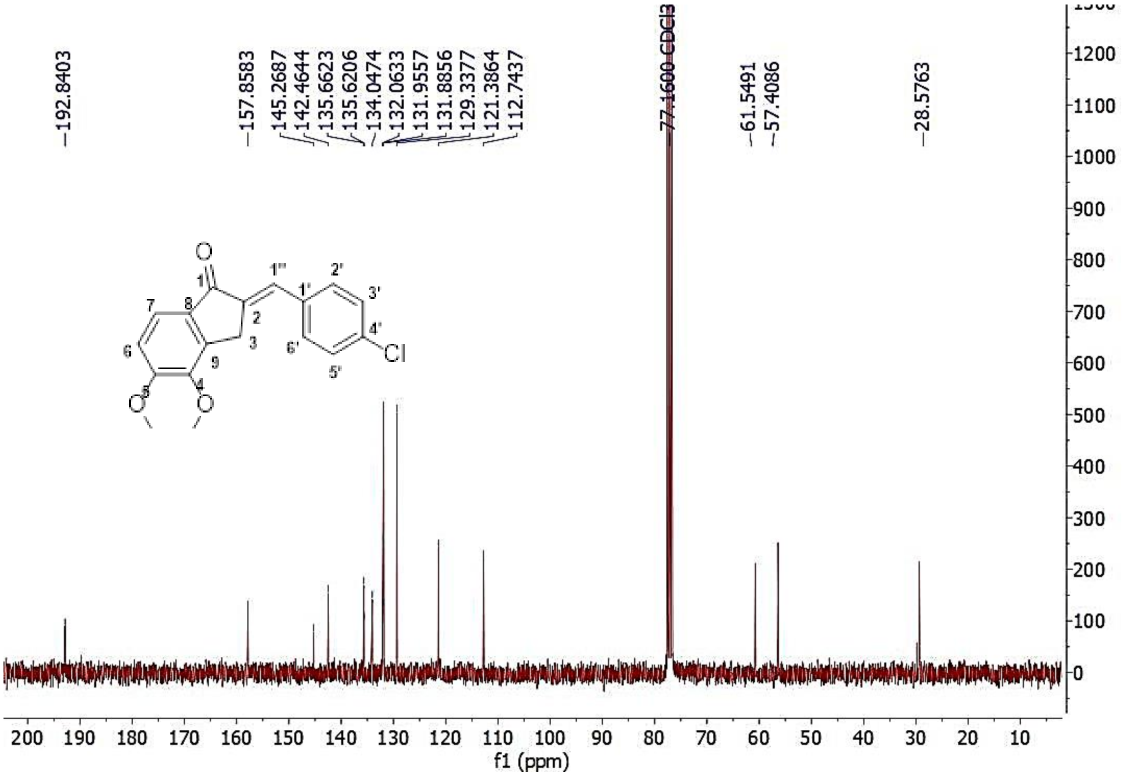

Figure S5. ^{1}H and ^{13}C NMR of compound **7**.

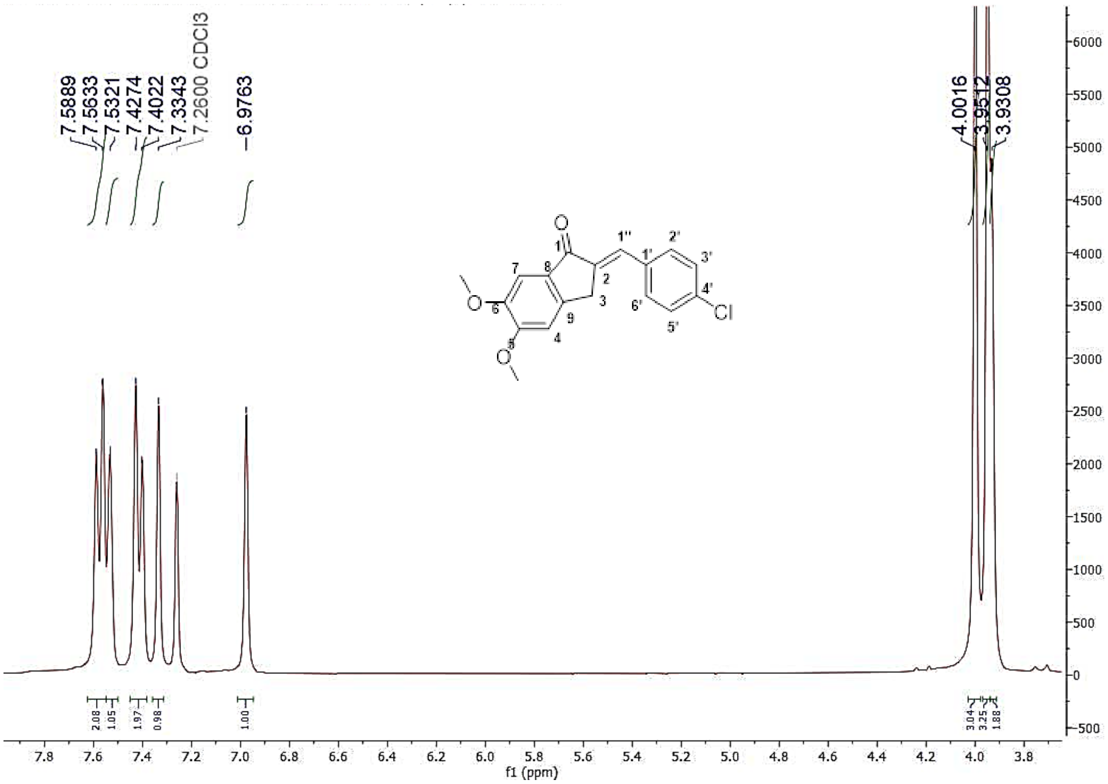

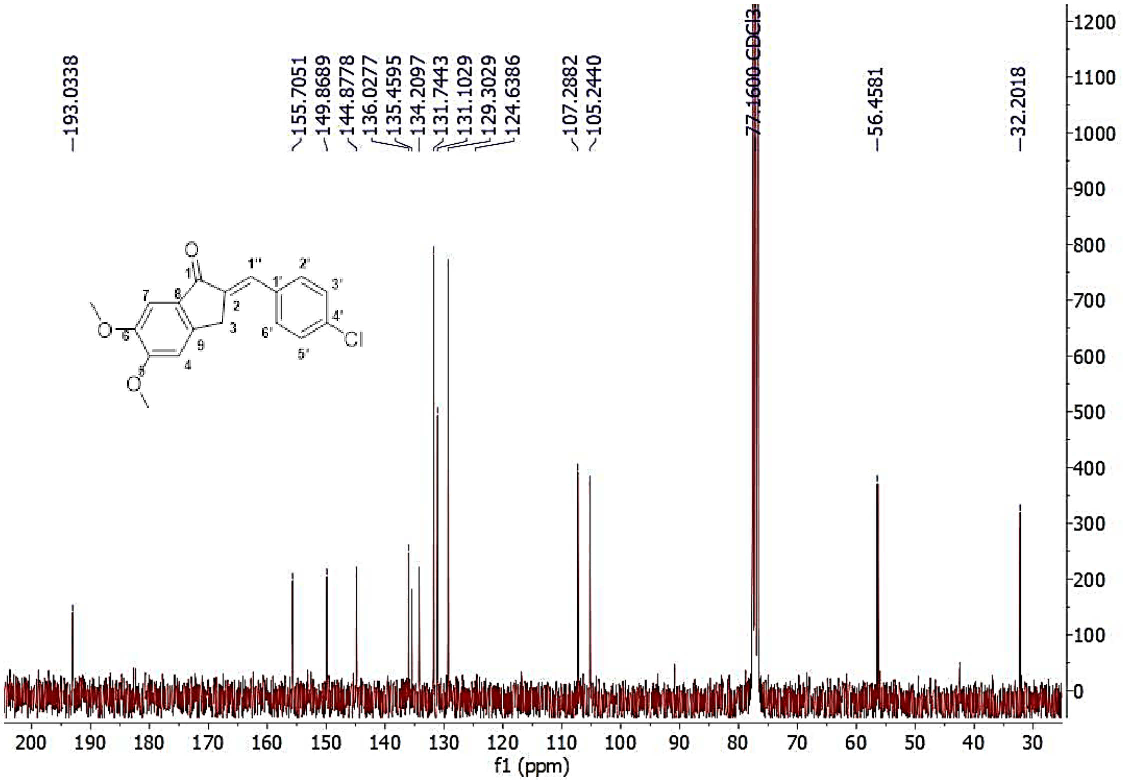

Figure S6. ^{1}H and ^{13}C NMR of compound **8**.

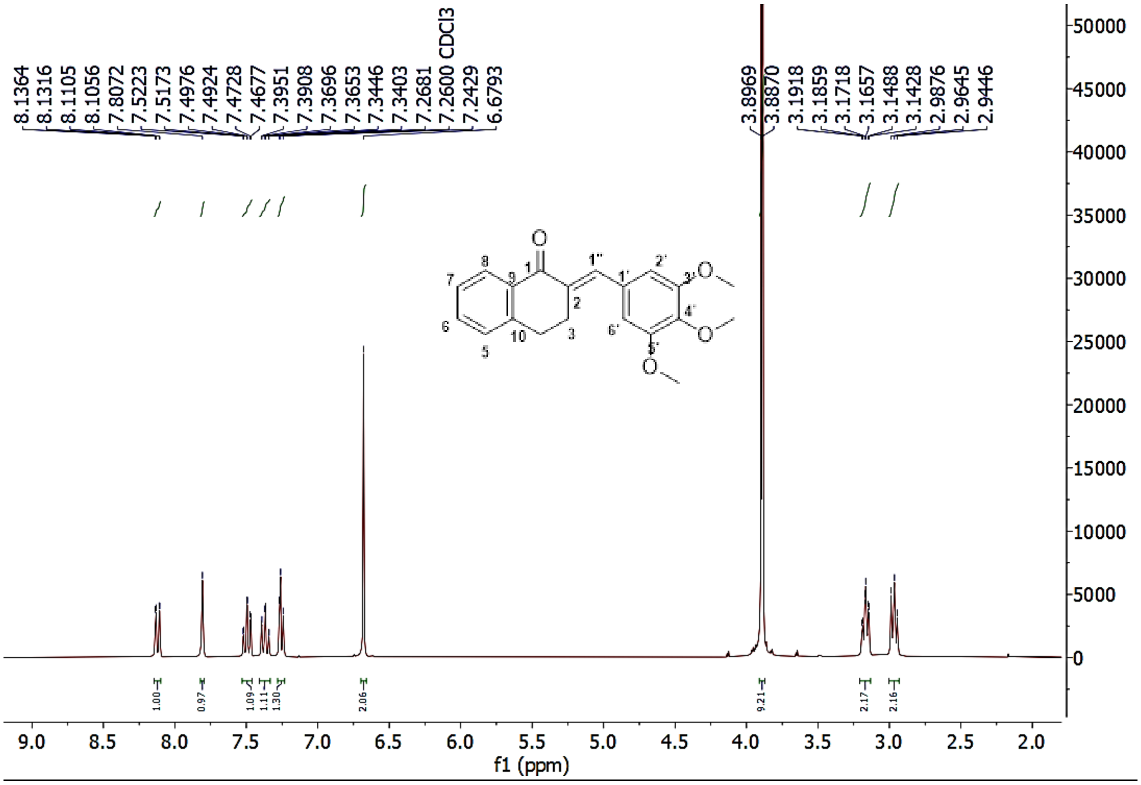

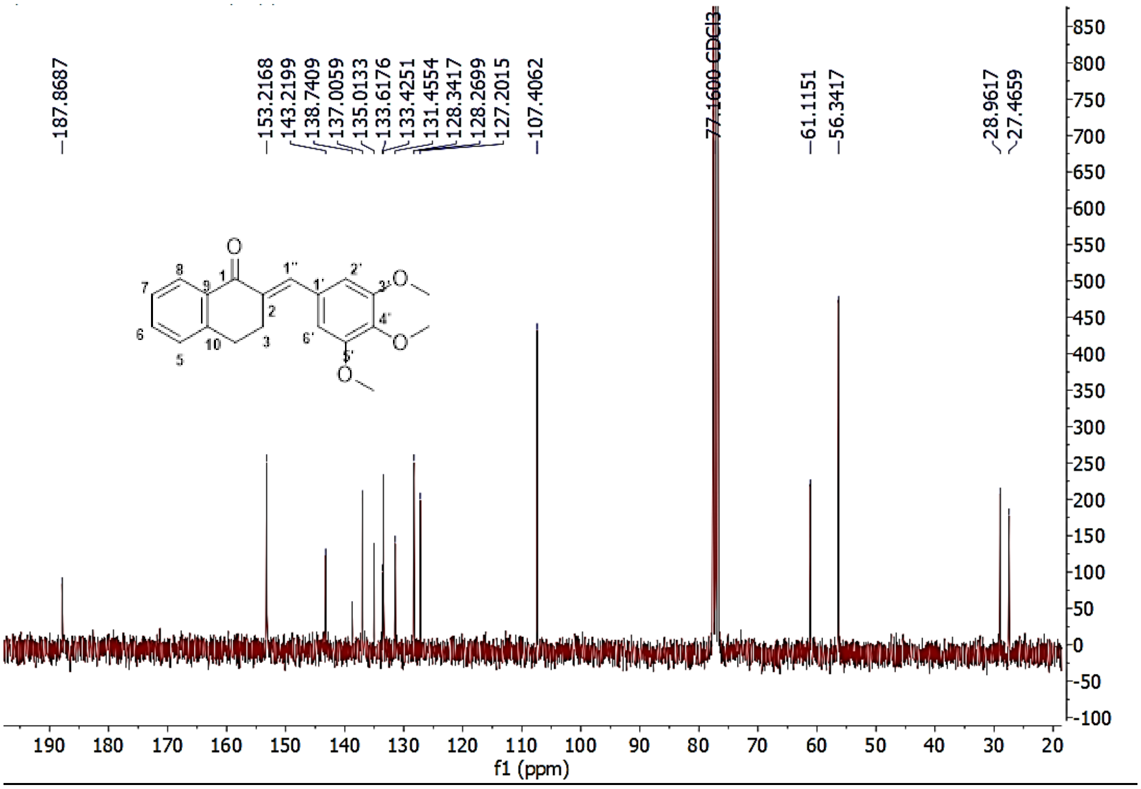

Figure S7. ^{1}H and ^{13}C NMR of compound **9**.

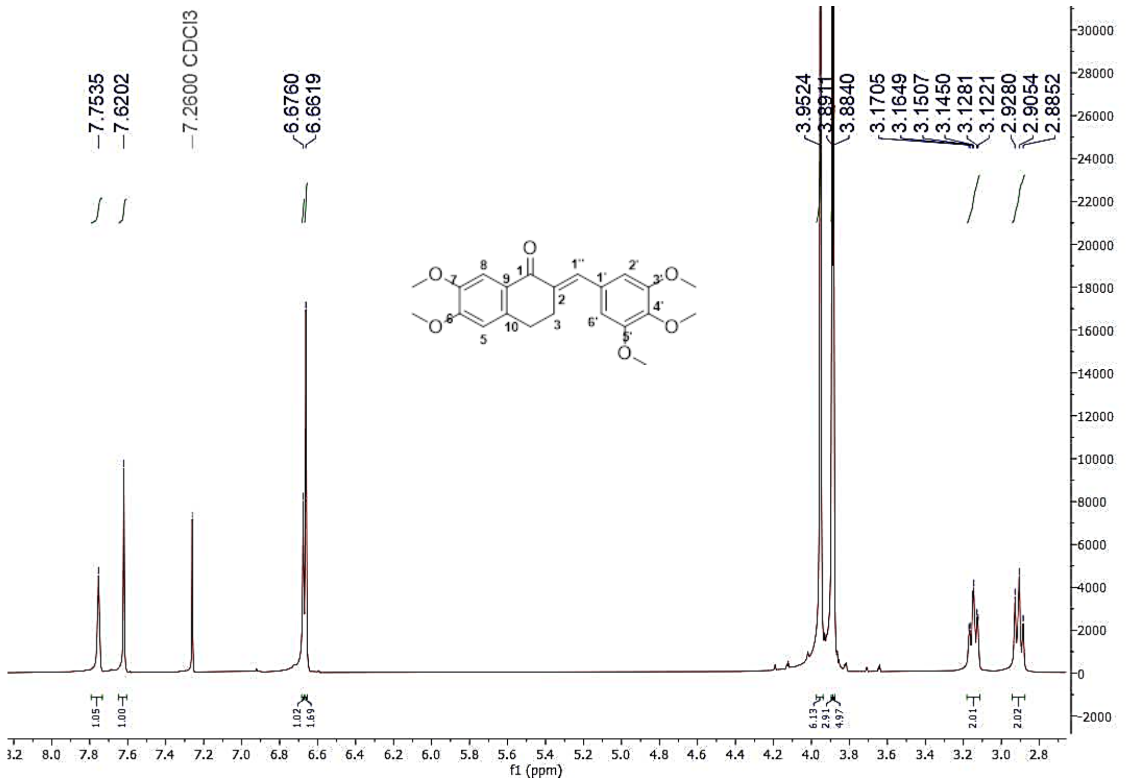

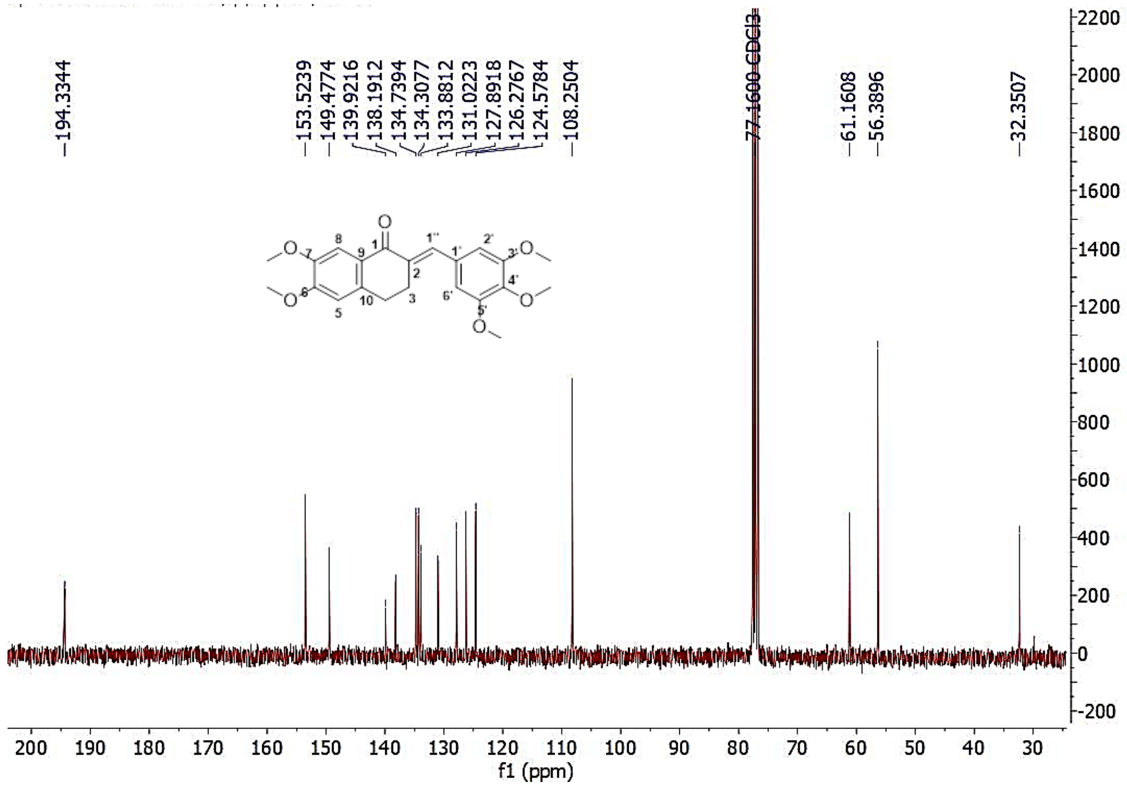

Figure S8. ^{1}H and ^{13}C NMR of compound **10**.

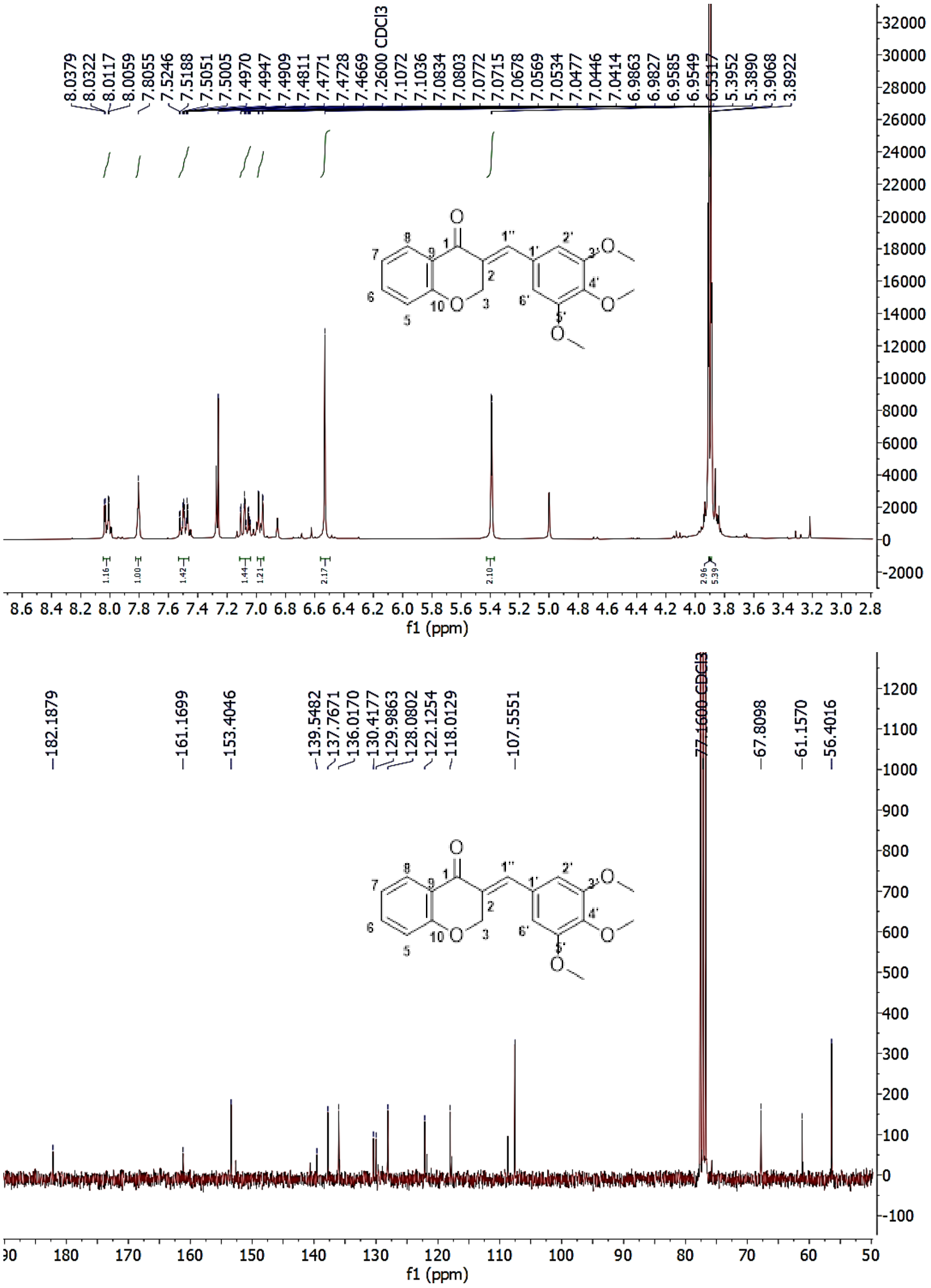

Figure S9. ^{1}H and ^{13}C NMR of compound **11**.

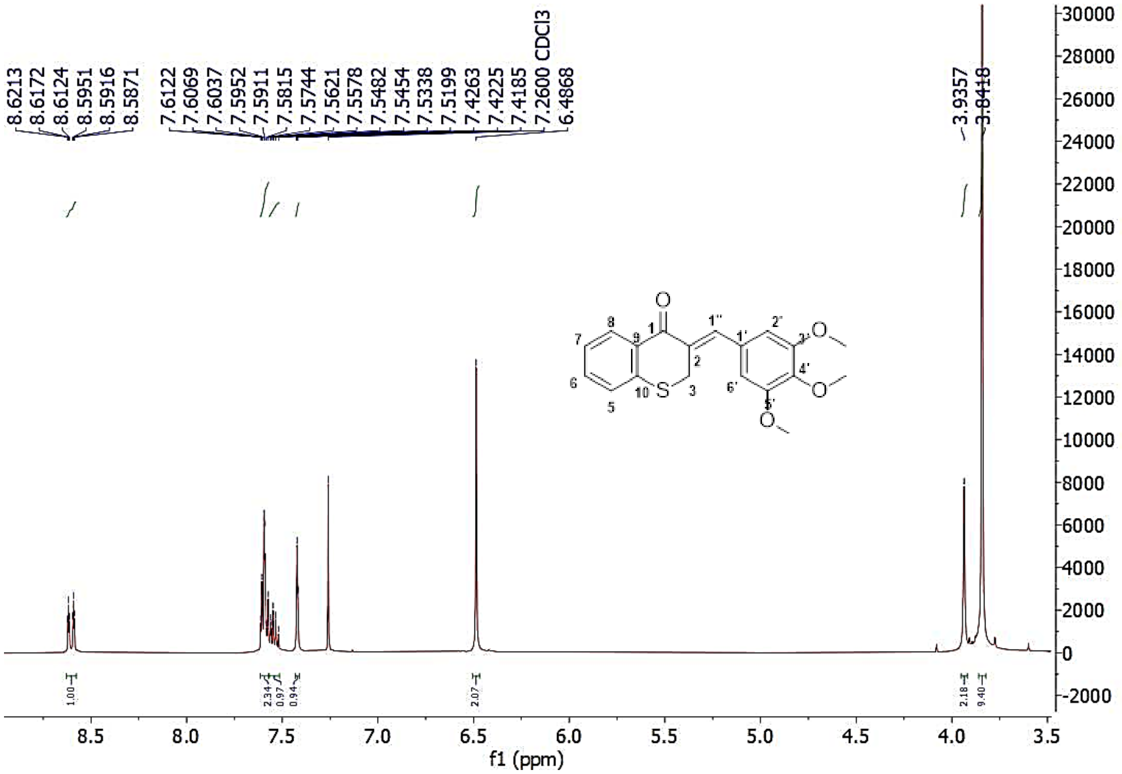

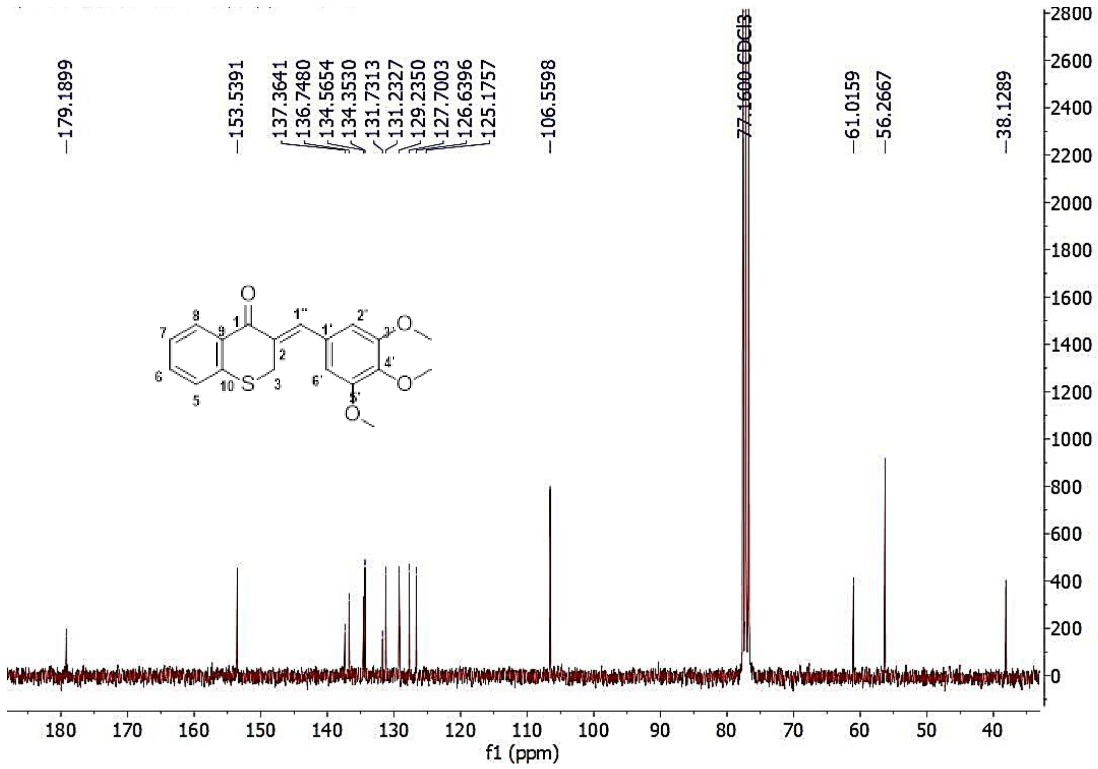

Figure S10. ^{1}H and ^{13}C NMR of compound **12**.

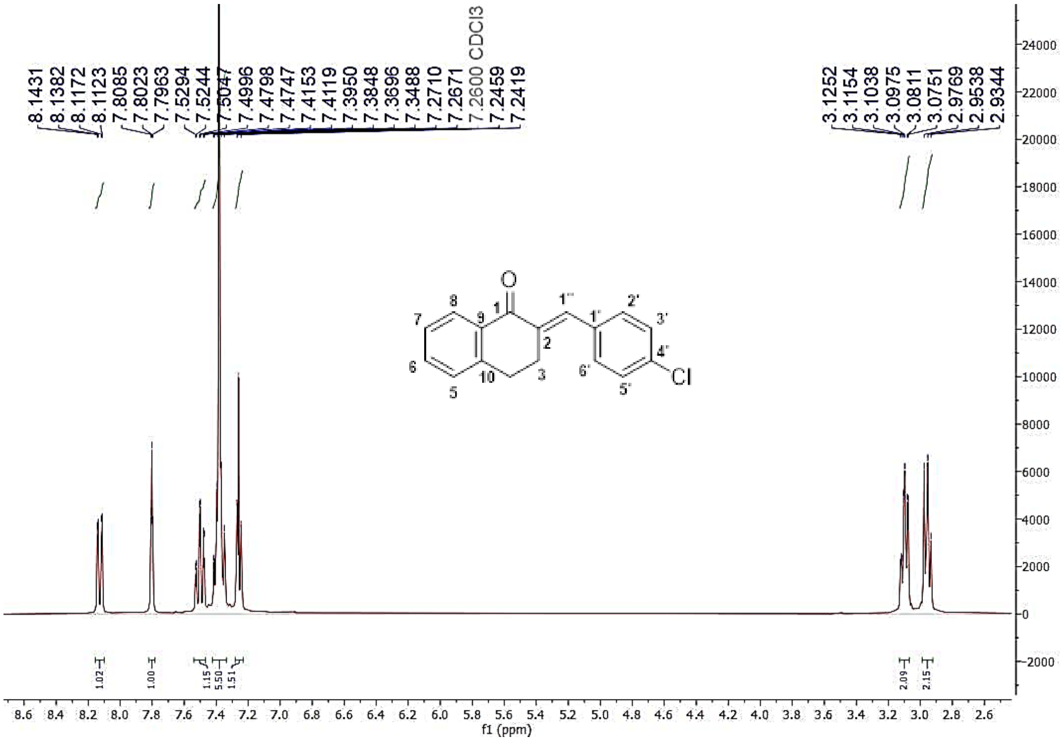

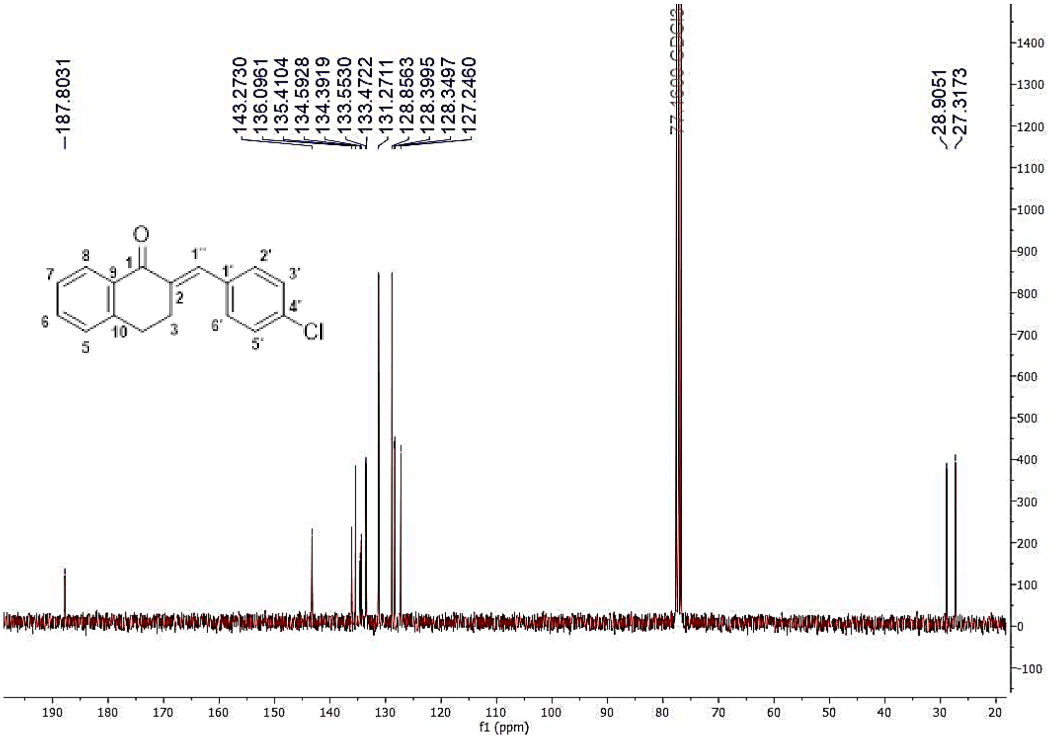

Figure S11. ^{1}H and ^{13}C NMR of compound **13**.

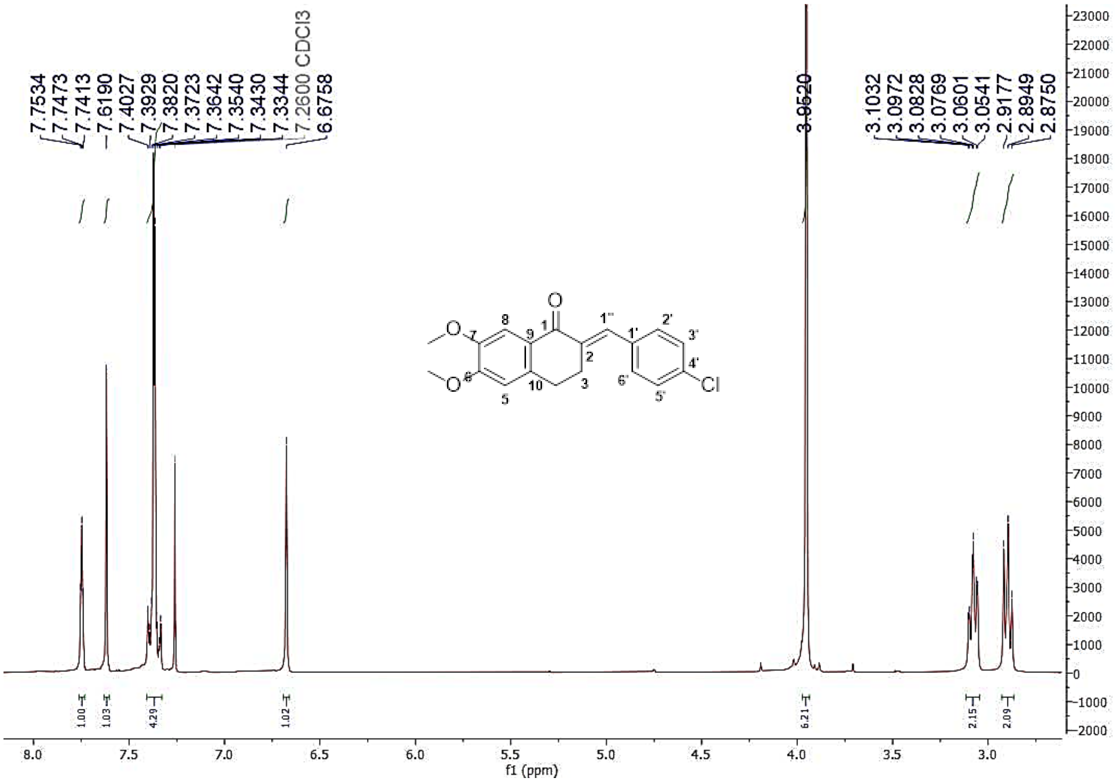

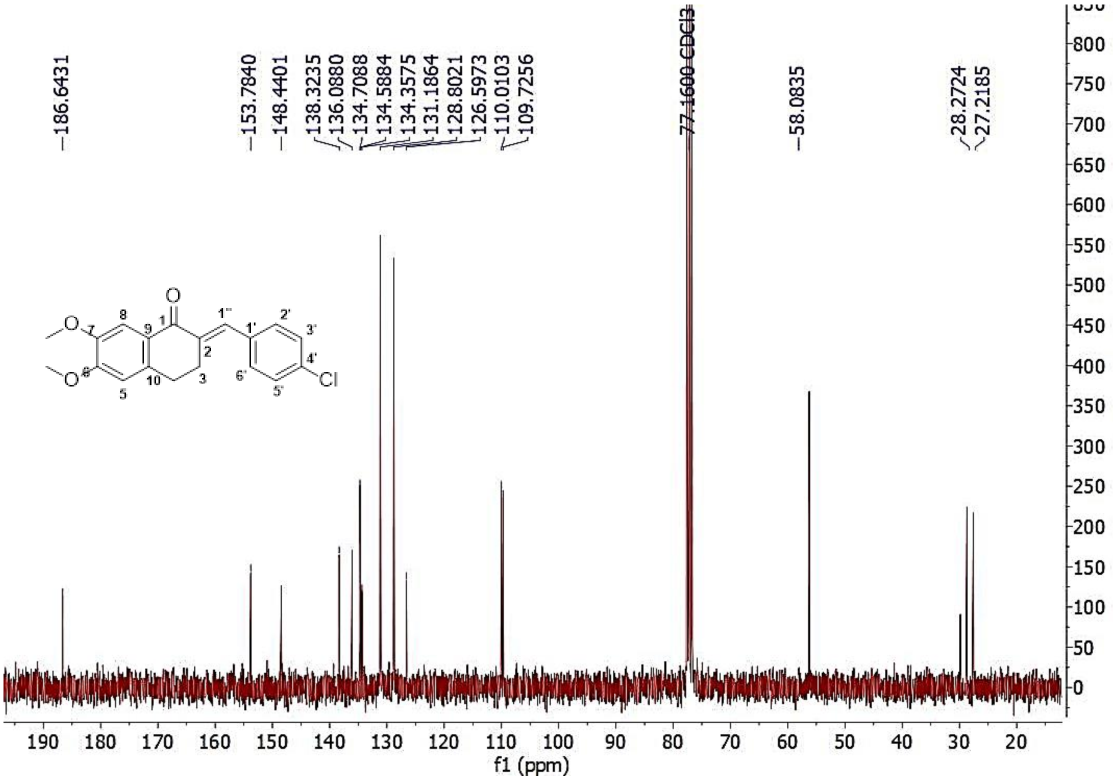

Figure S12. ^{1}H and ^{13}C NMR of compound **14**.

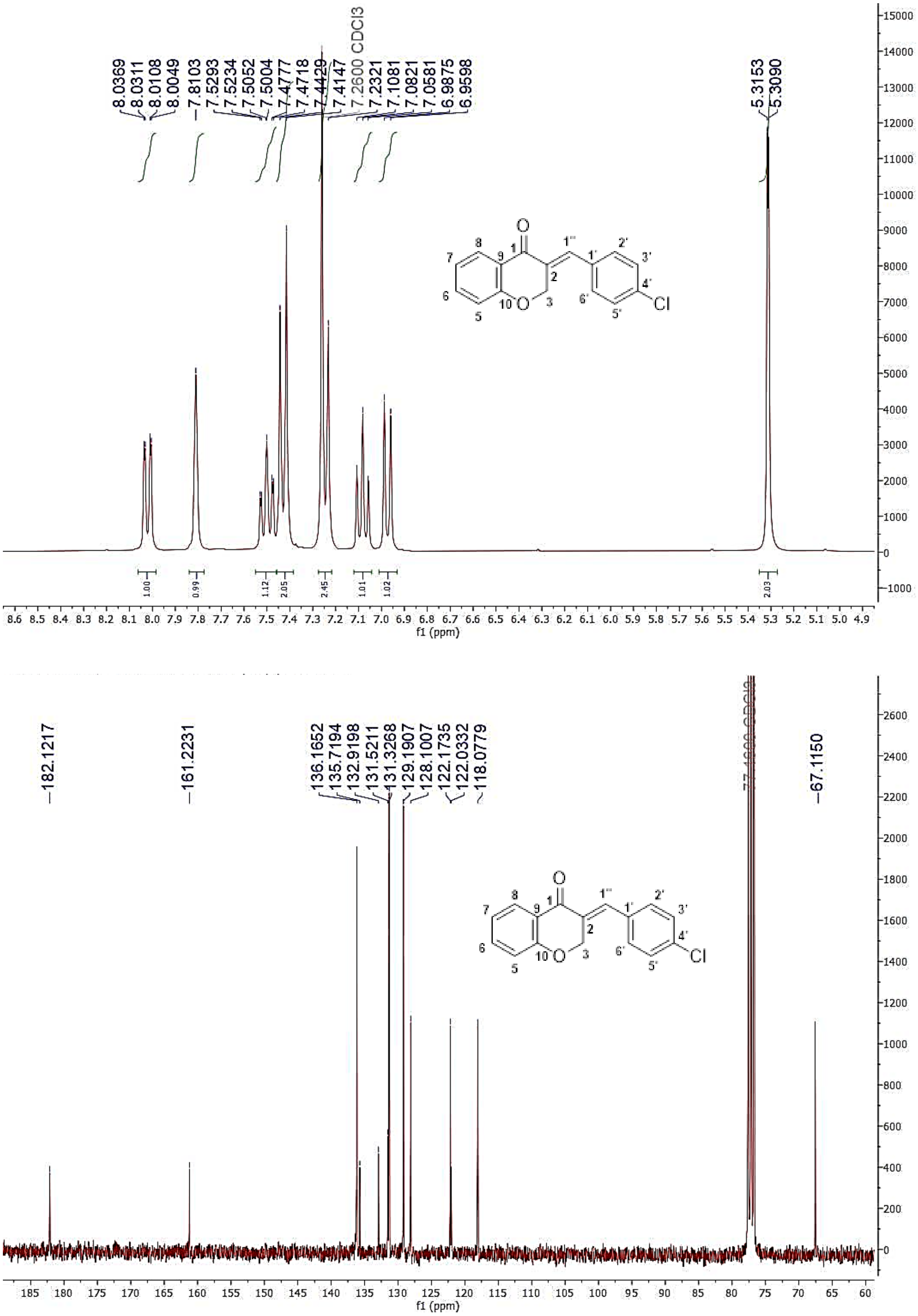

Figure S13. ^{1}H and ^{13}C NMR of compound **15**.

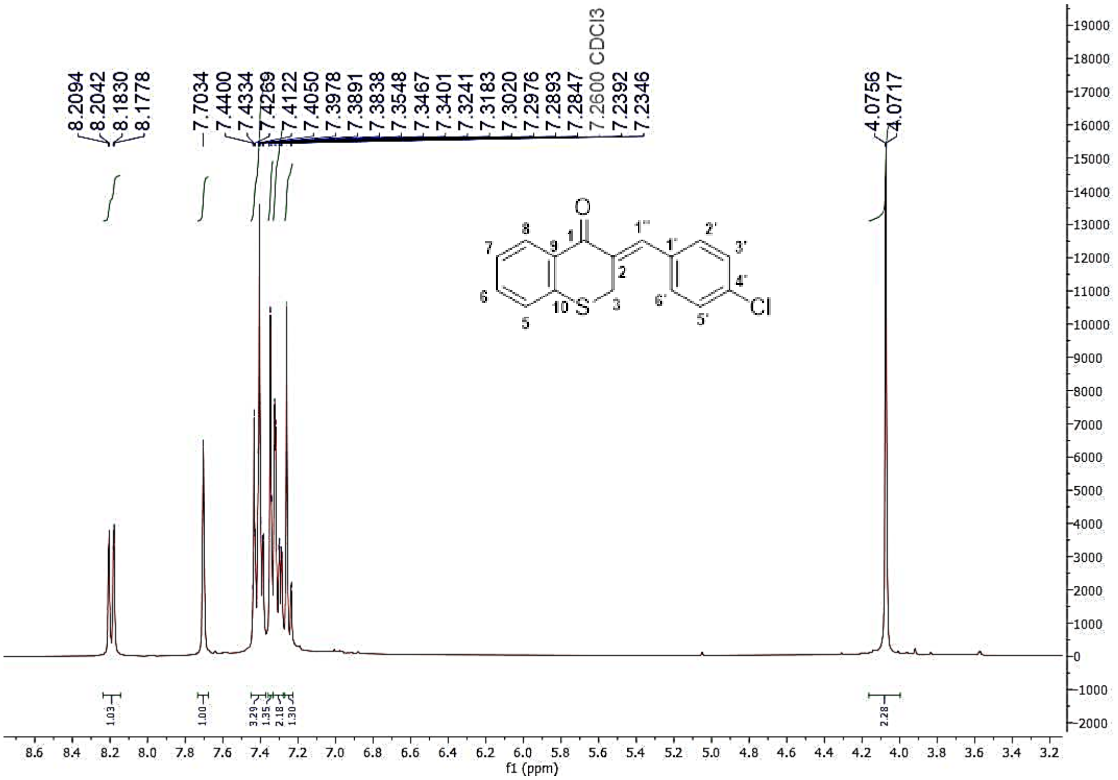

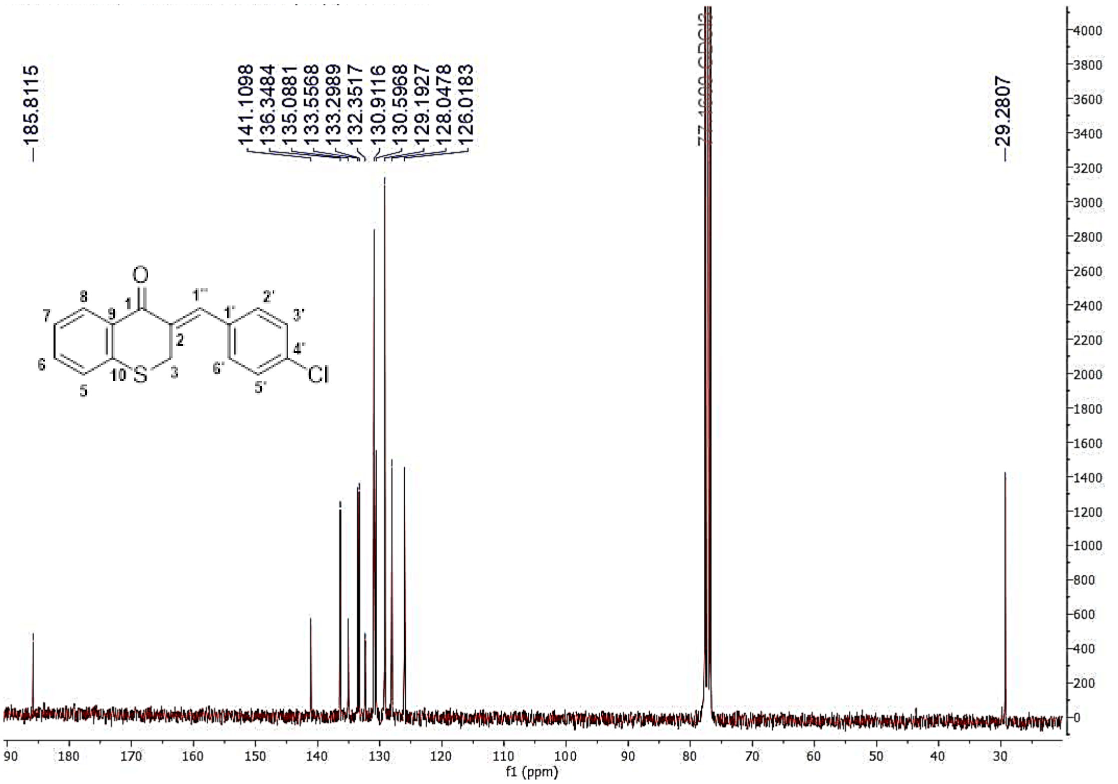

Figure S14. ^{1}H and ^{13}C NMR of compound **16**.

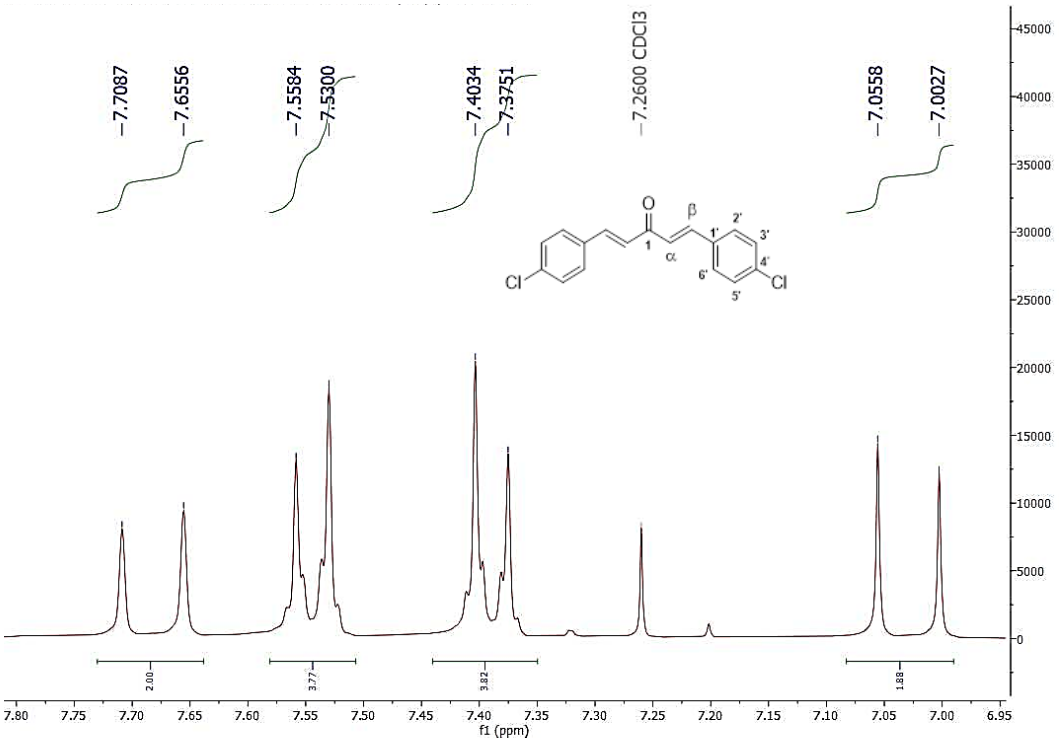

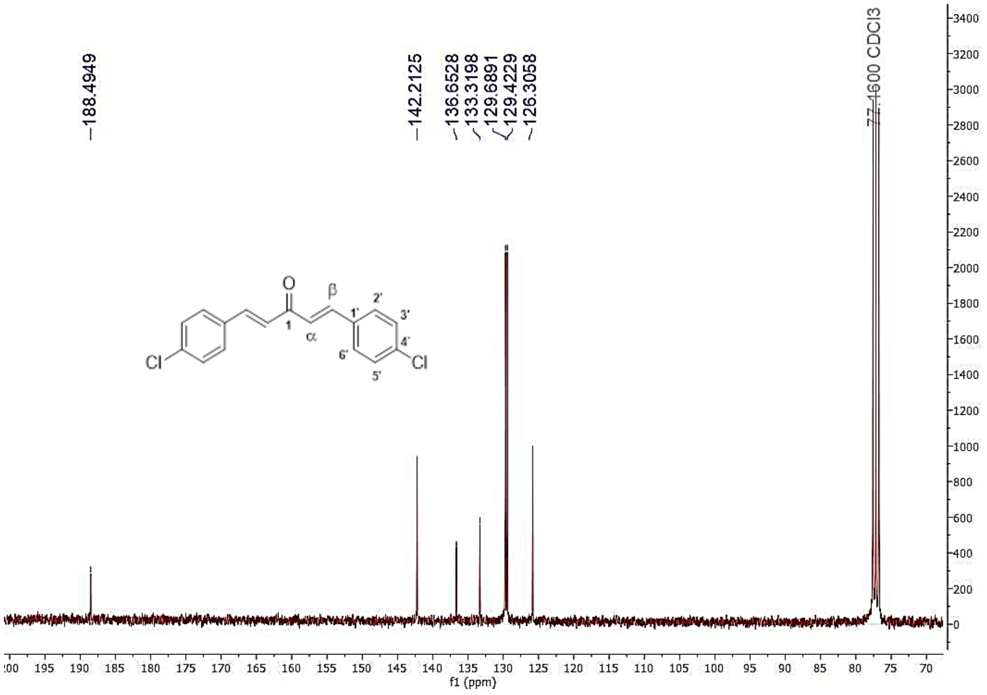

Figure S15. ^{1}H and ^{13}C NMR of compound **17**.

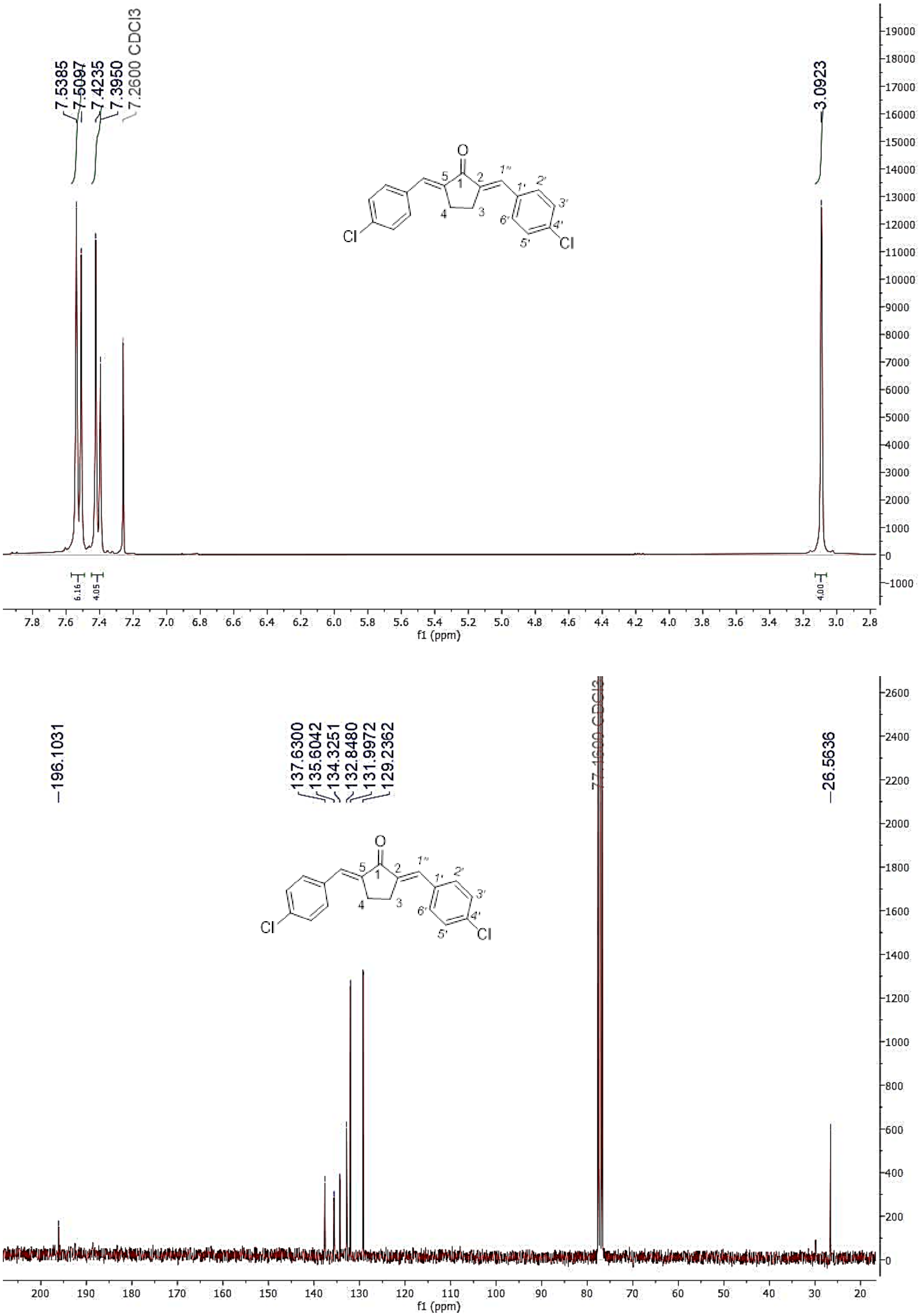

Figure S16. ^{1}H and ^{13}C NMR of compound **18**.

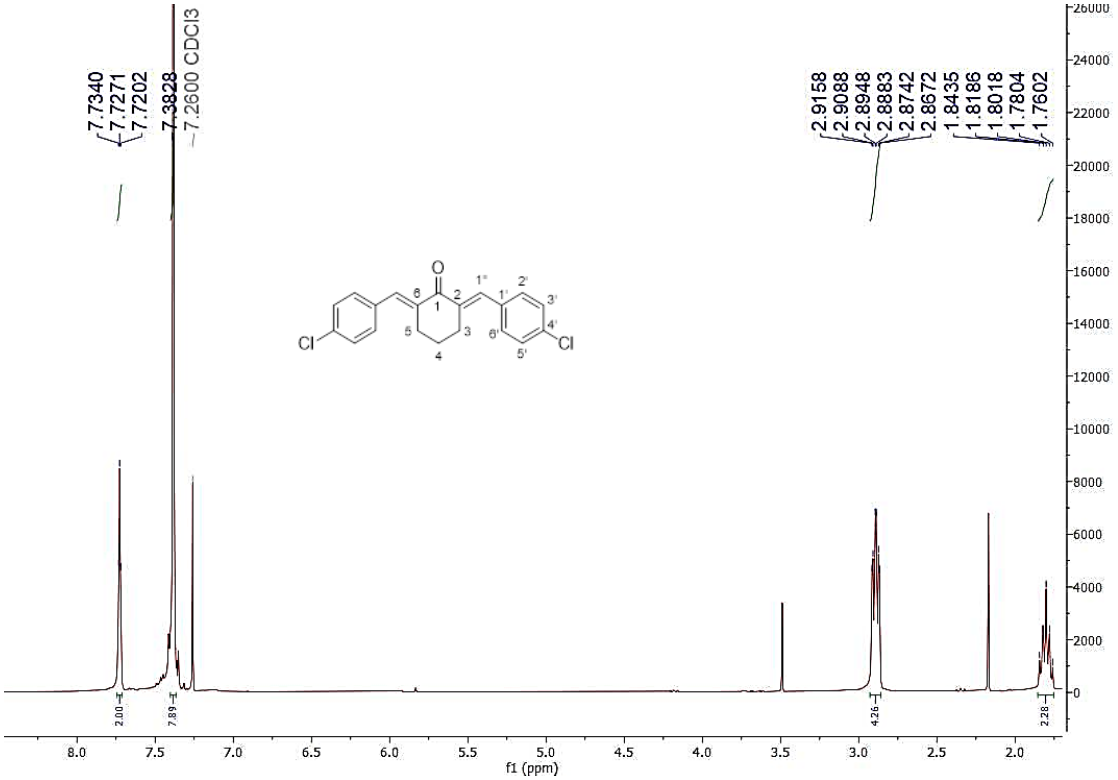

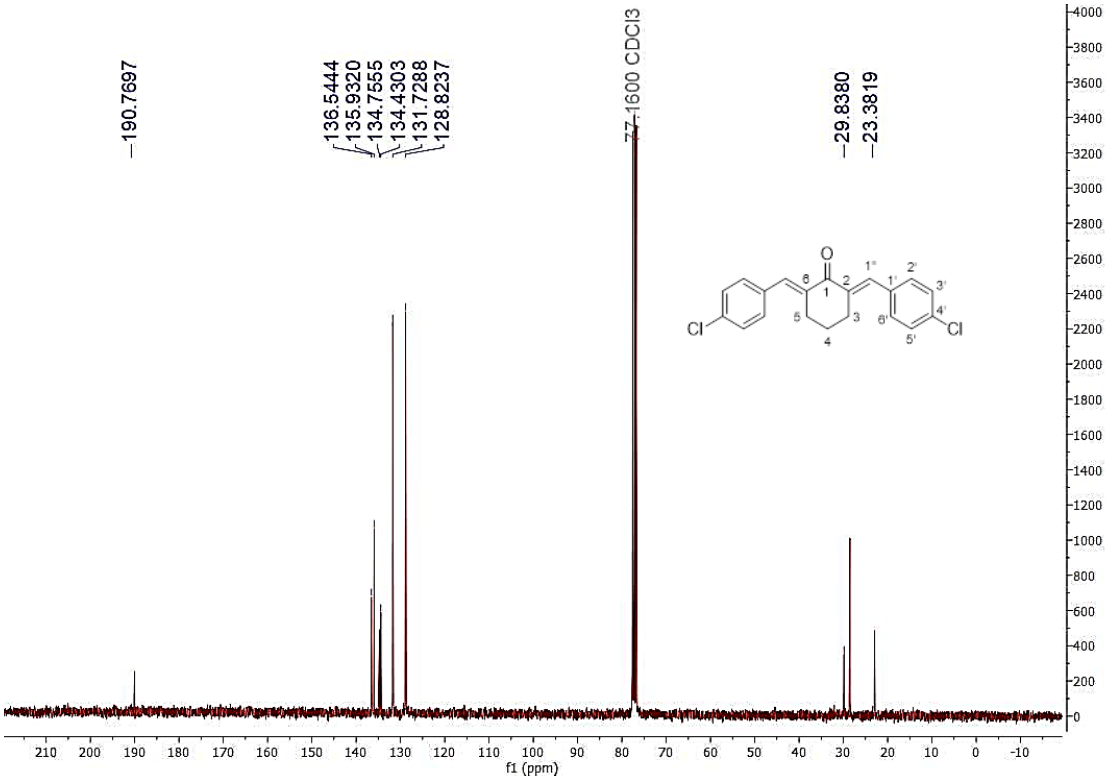

Figure S17. ^{1}H and ^{13}C NMR of compound **19**.

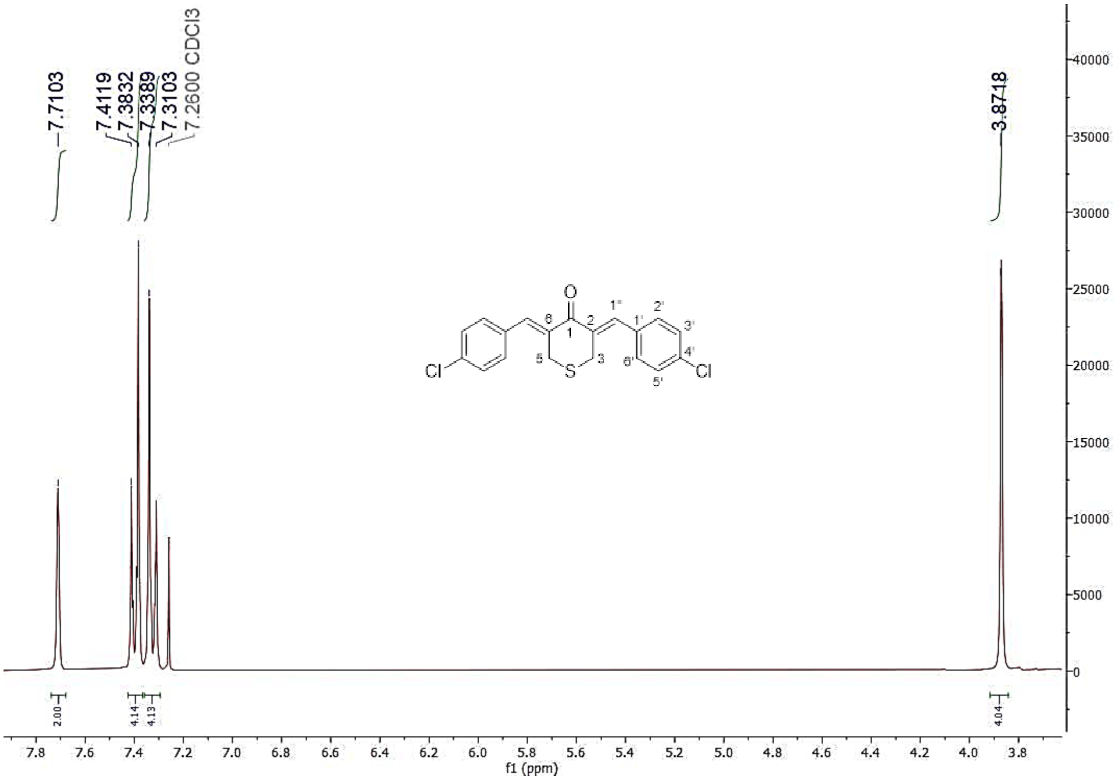

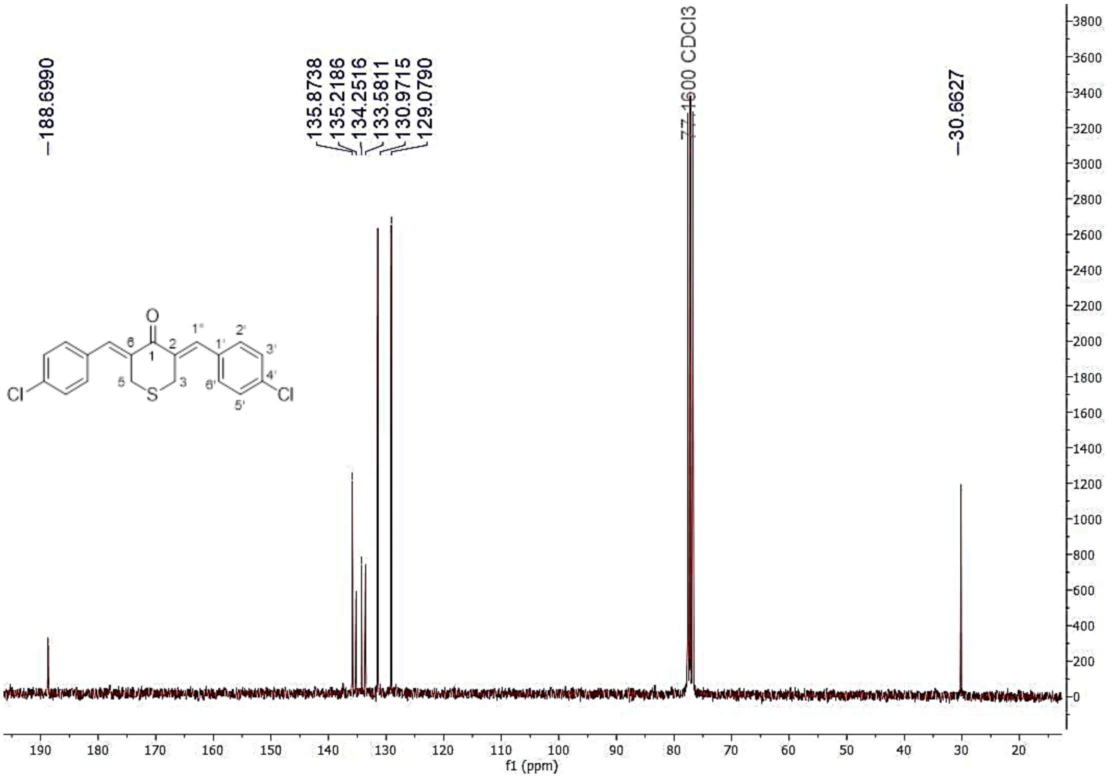

Figure S18. ^{1}H and ^{13}C NMR of compound **20**.

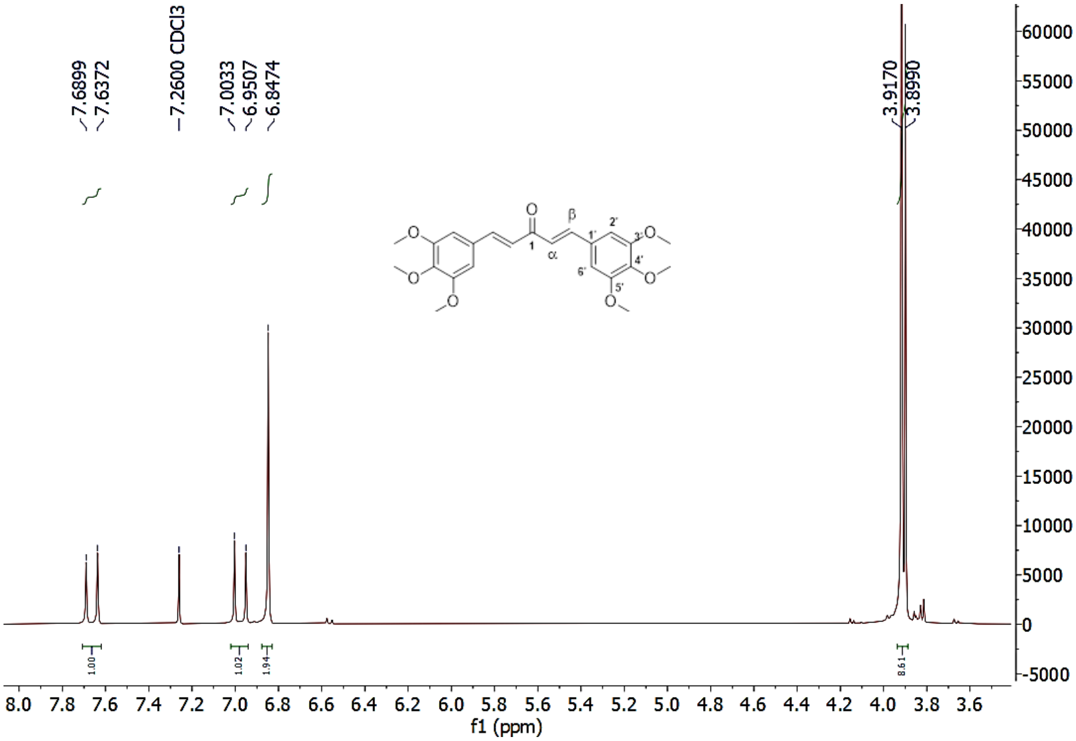

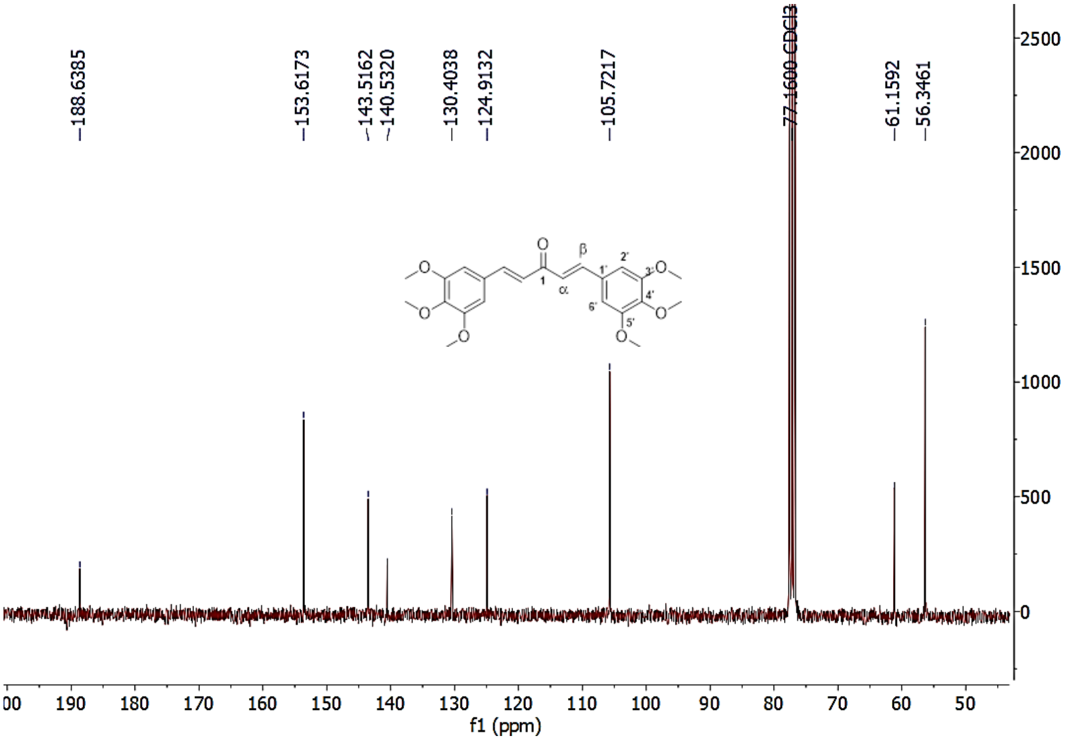

Figure S19. ^{1}H and ^{13}C NMR of compound **21**.

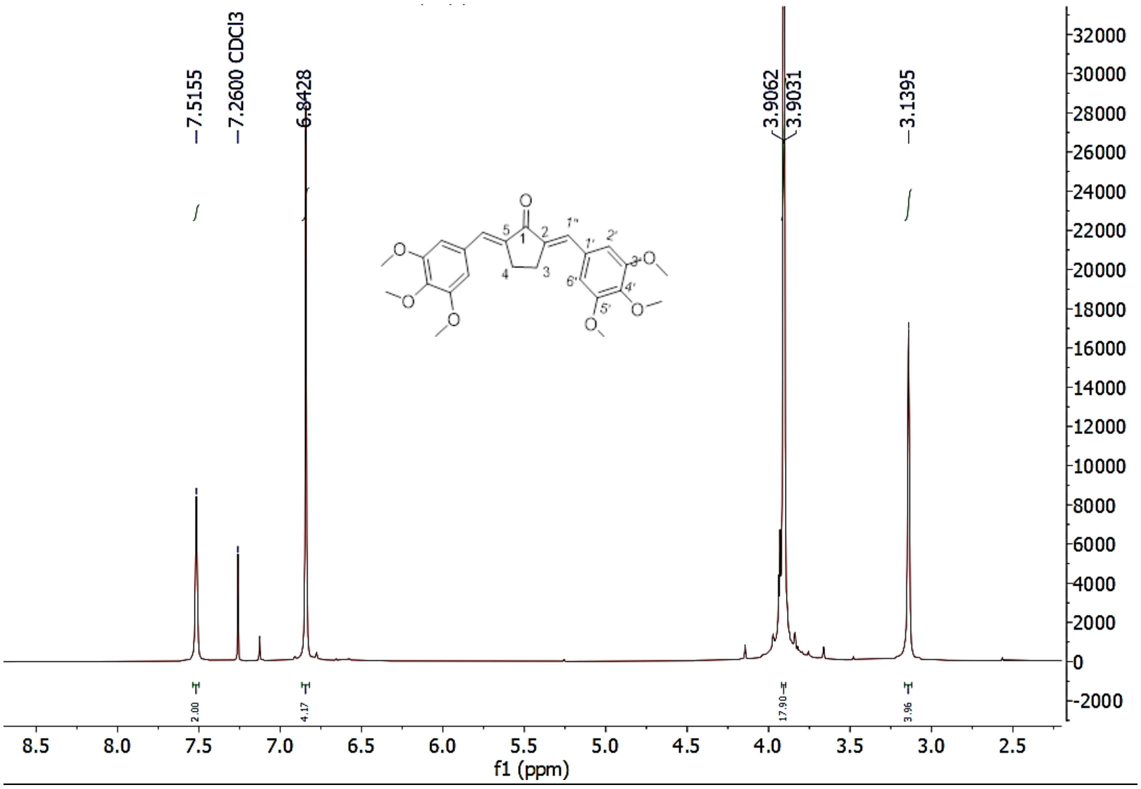

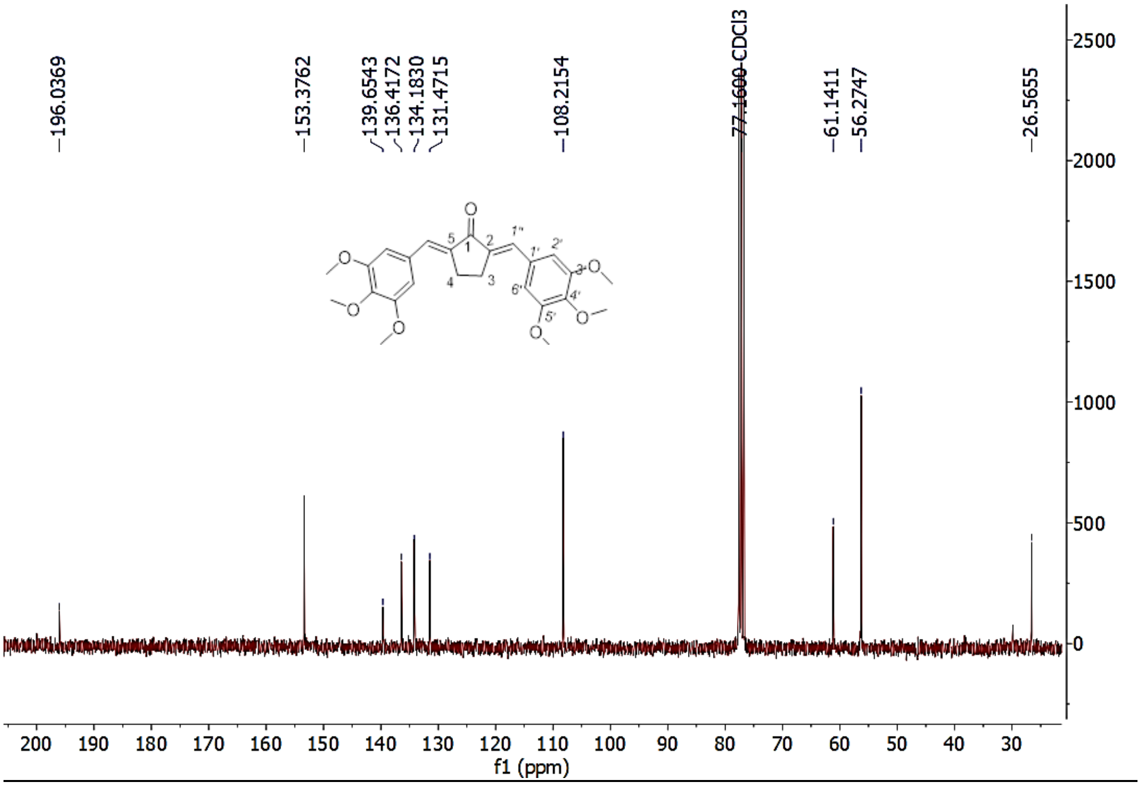

Figure S20. ^{1}H and ^{13}C NMR of compound **22**

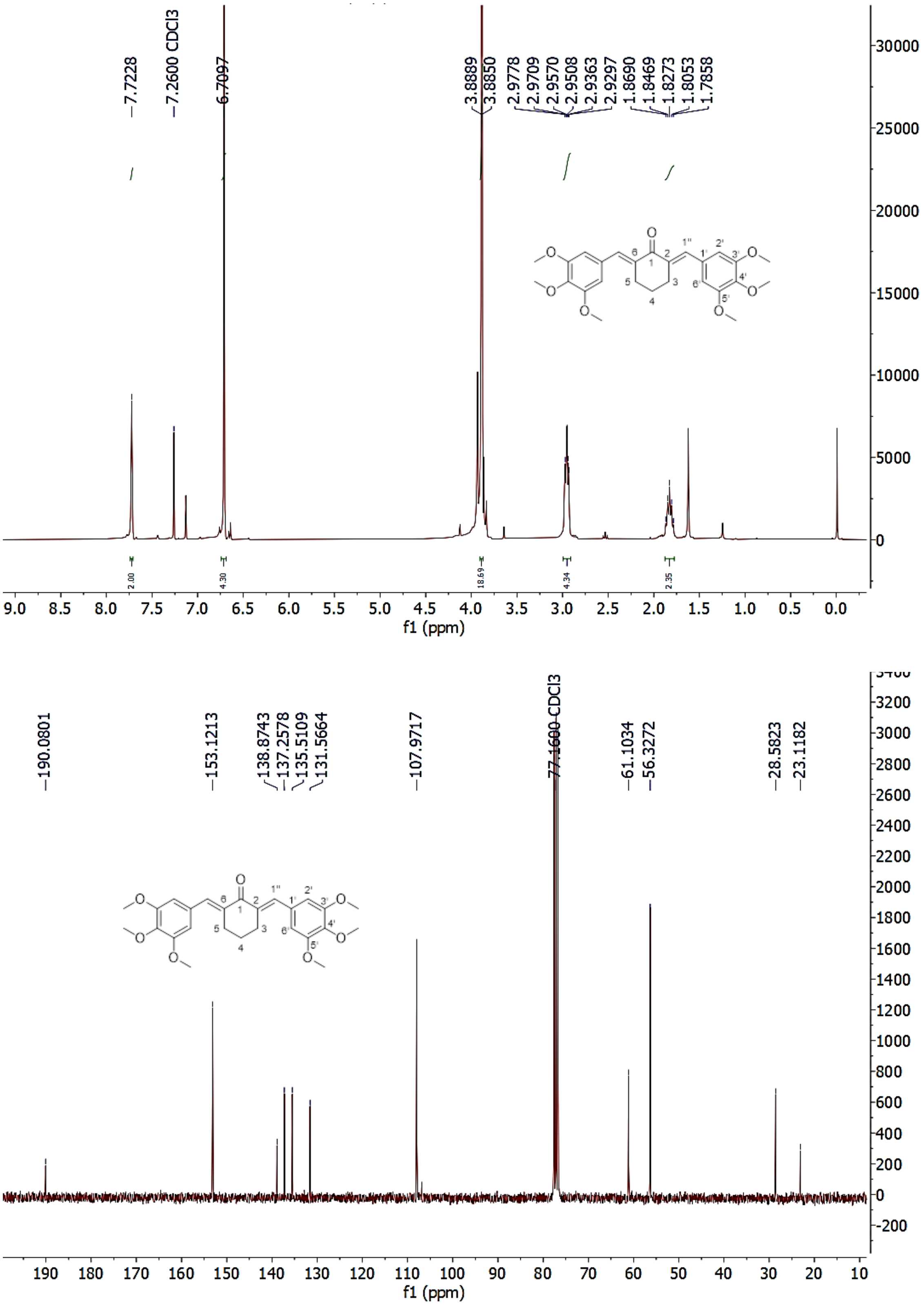

Figure S21. ^{1}H and ^{13}C NMR of compound **23**.

pharmaceuticals

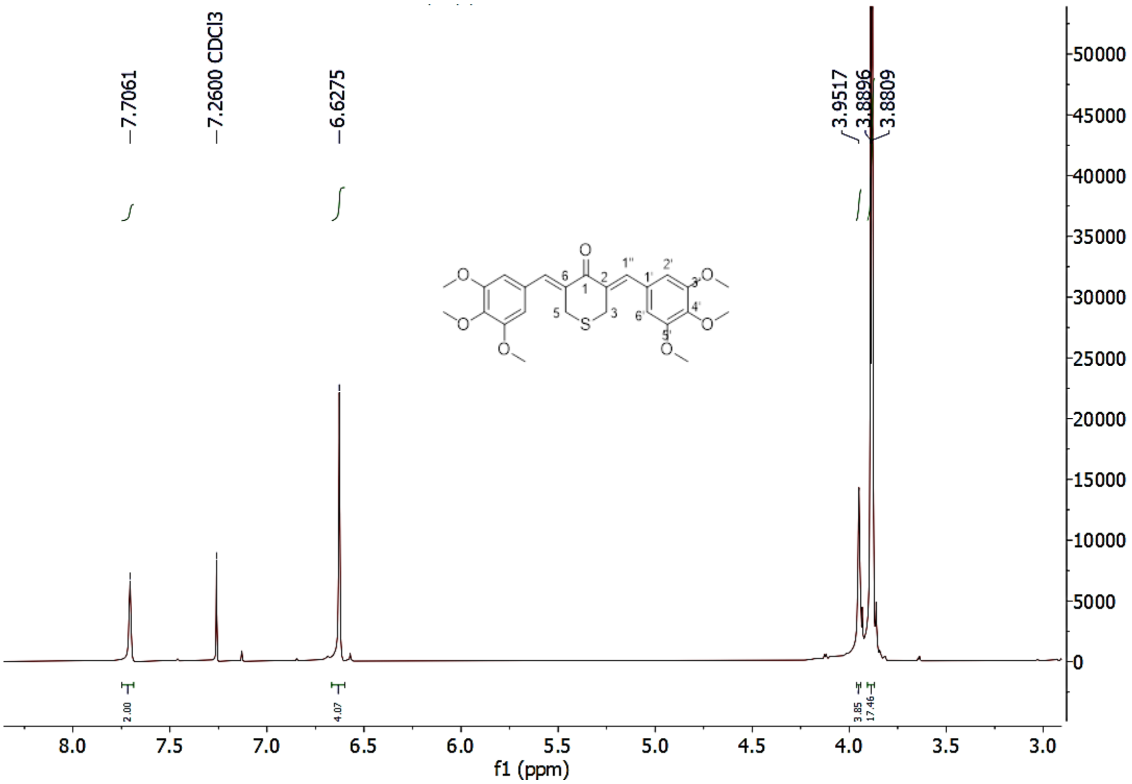

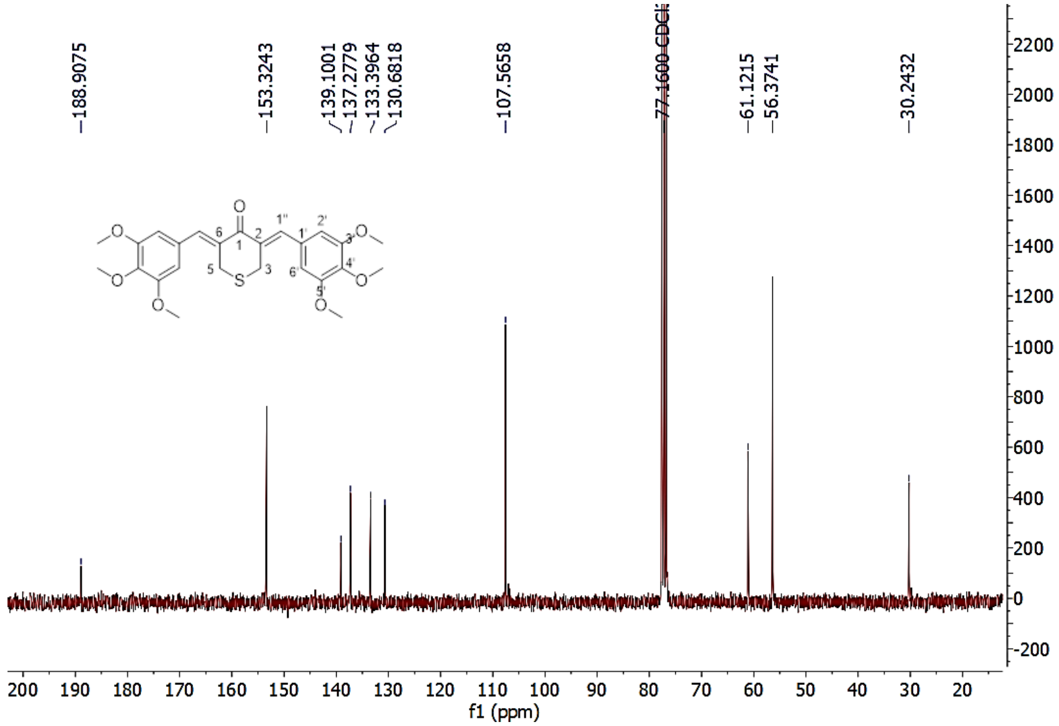

Figure S22. ^{1}H and ^{13}C NMR of compound **24**.

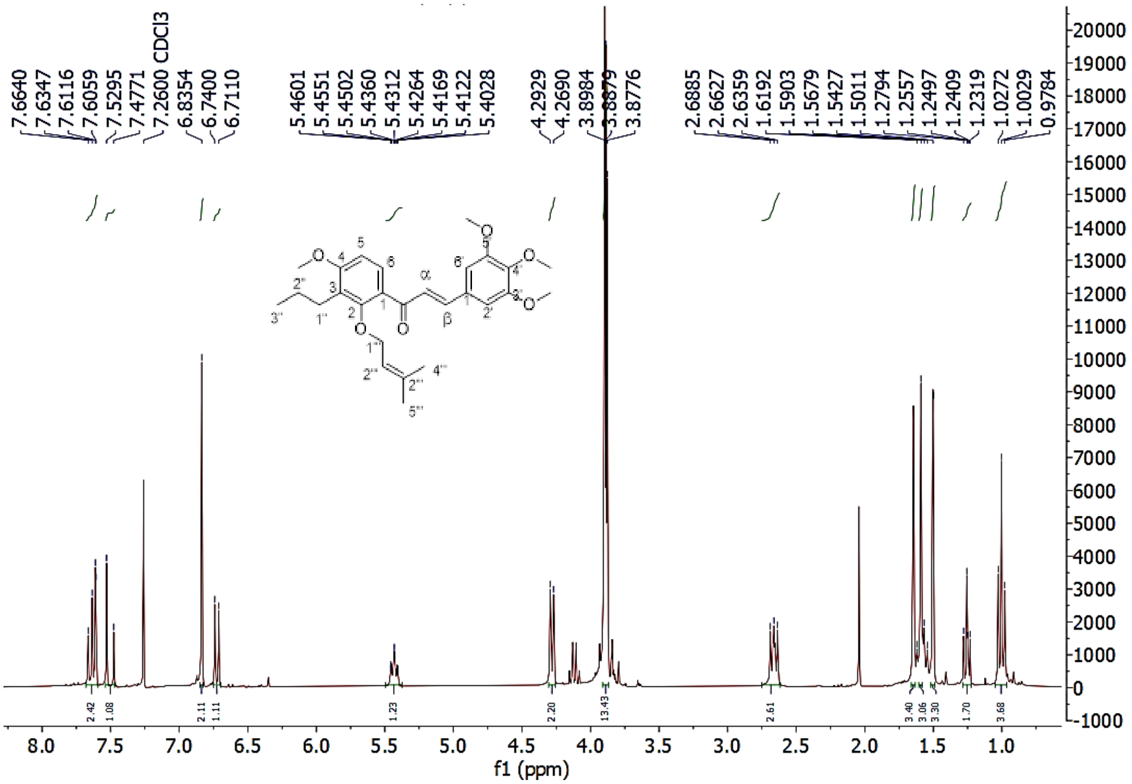

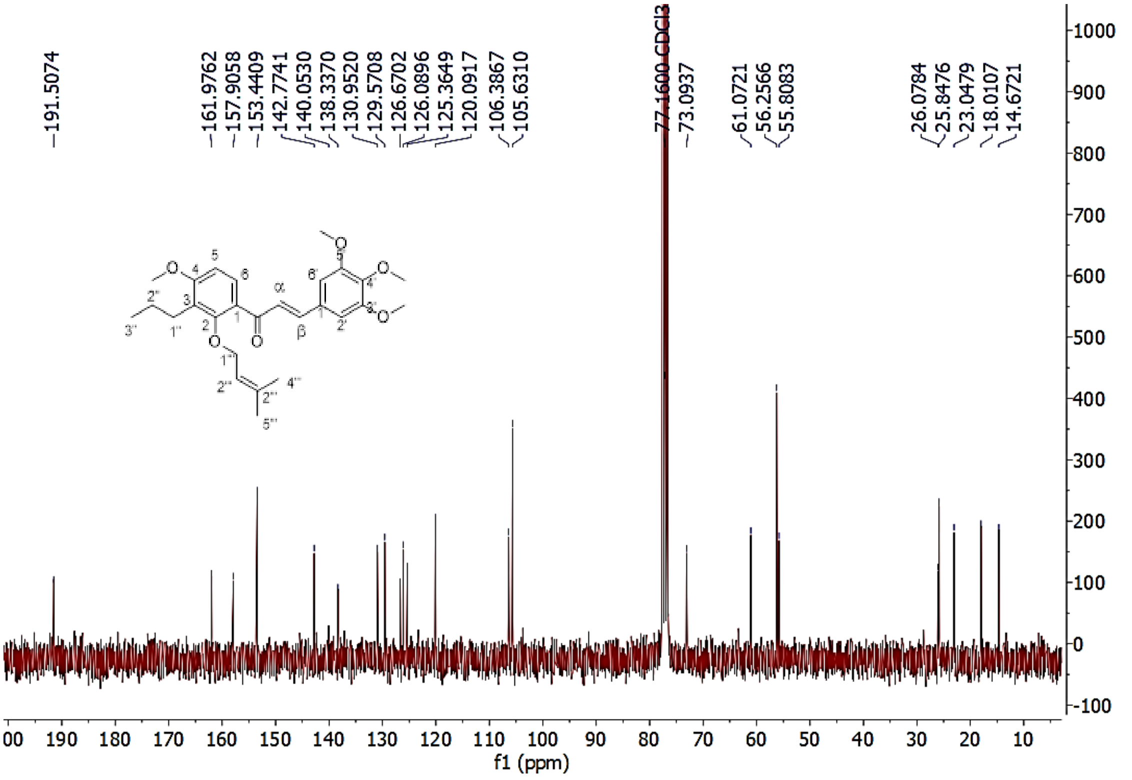

Figure S23. ^{1}H and ^{13}C NMR of compound **26**.

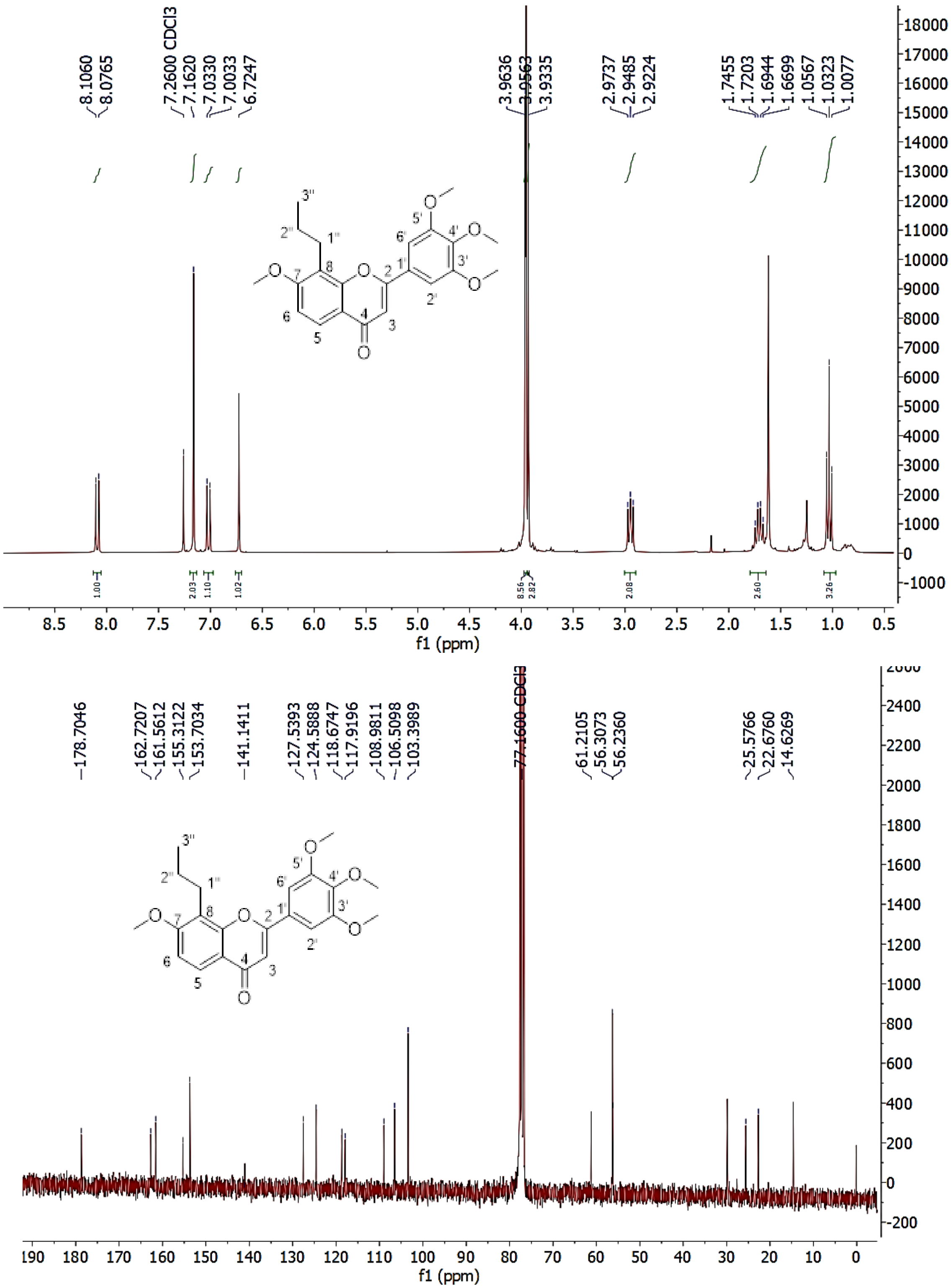

Figure S24. ^{1}H and ^{13}C NMR of compound **27**.

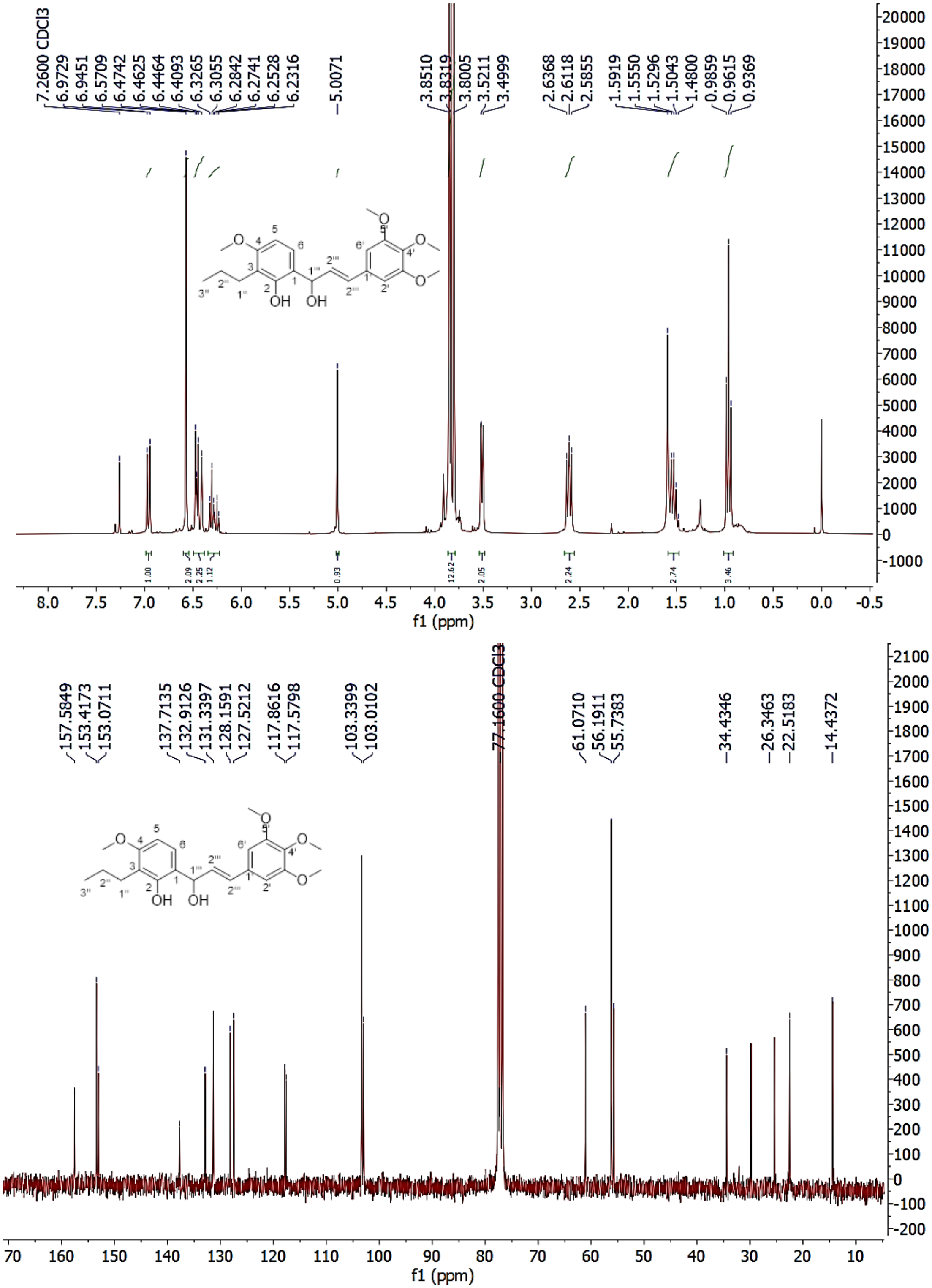

Figure S25. ^1H and ^{13}C NMR of compound **28**.

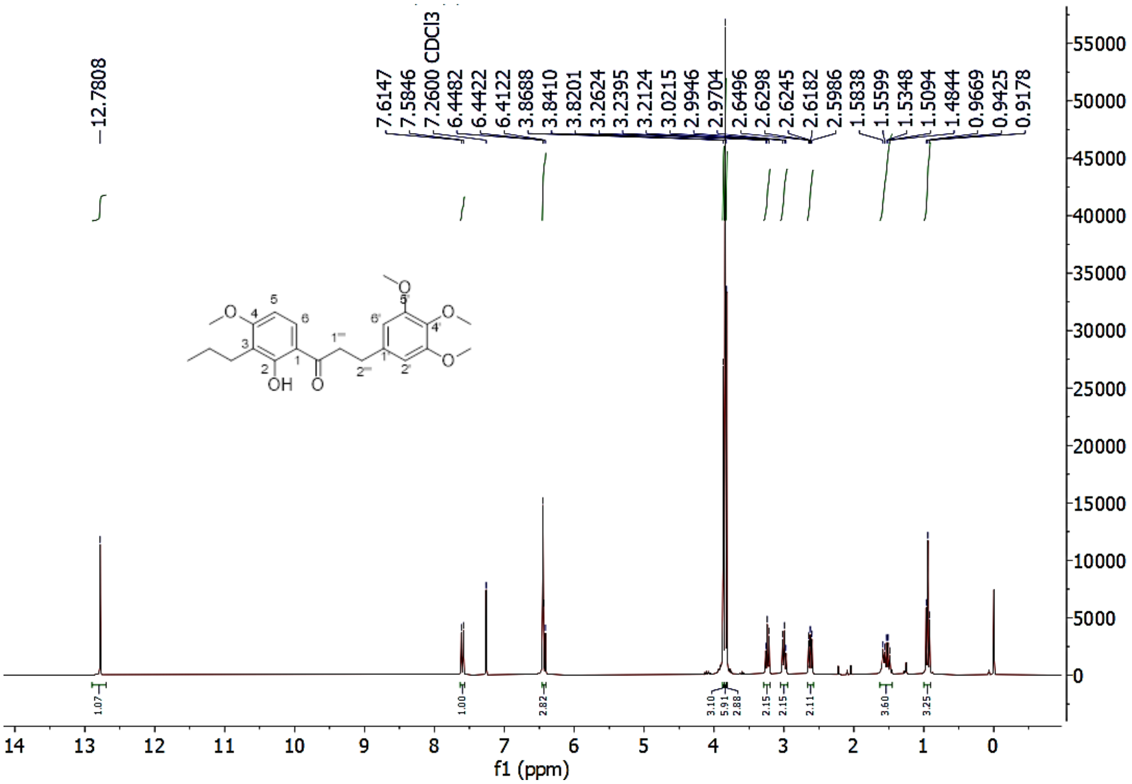

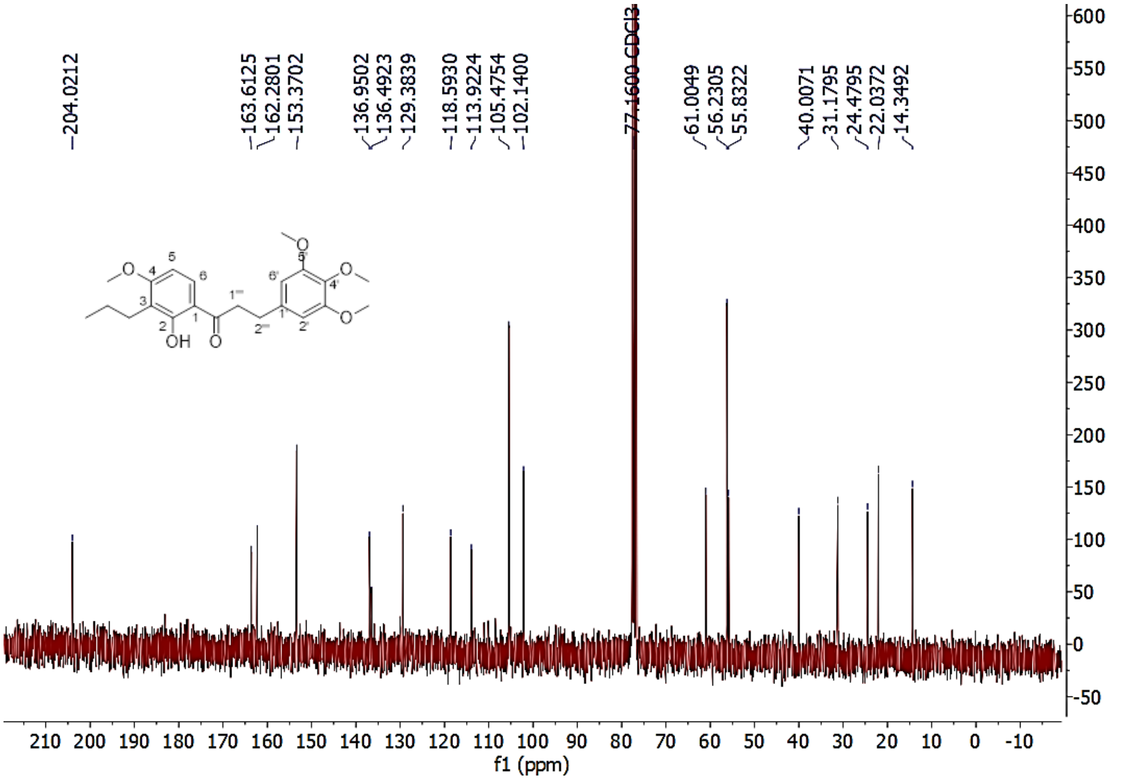

Figure S26. ^{1}H and ^{13}C NMR of compound **29**.

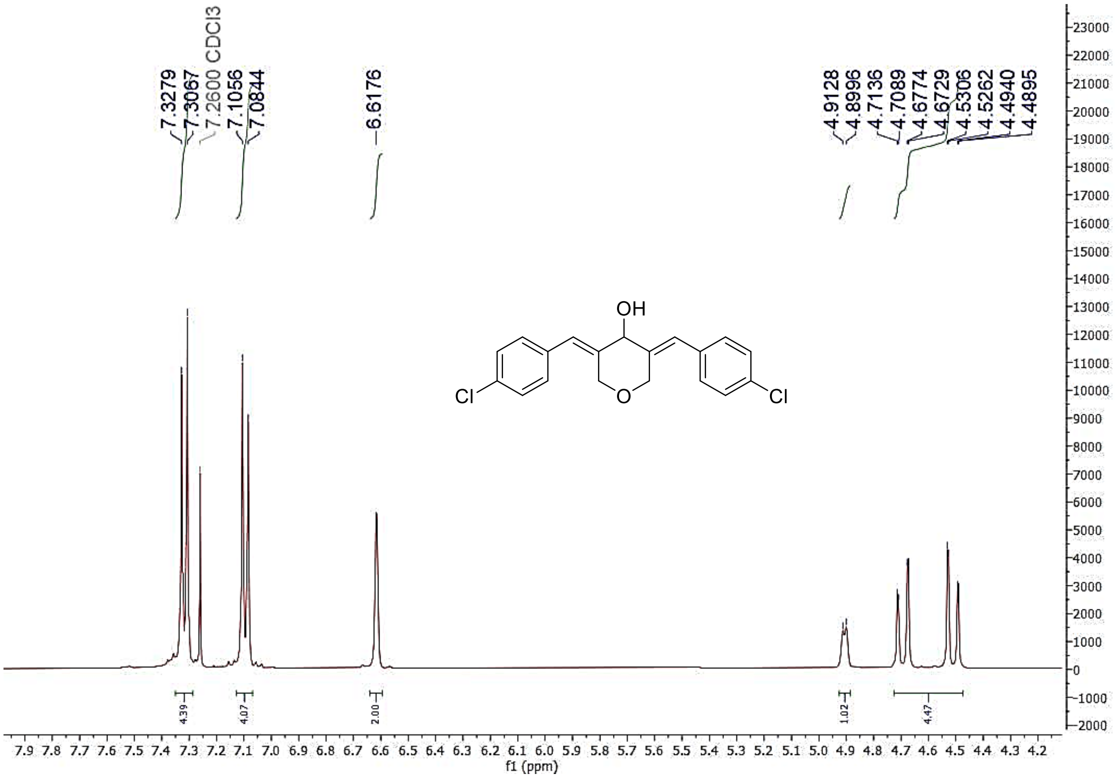

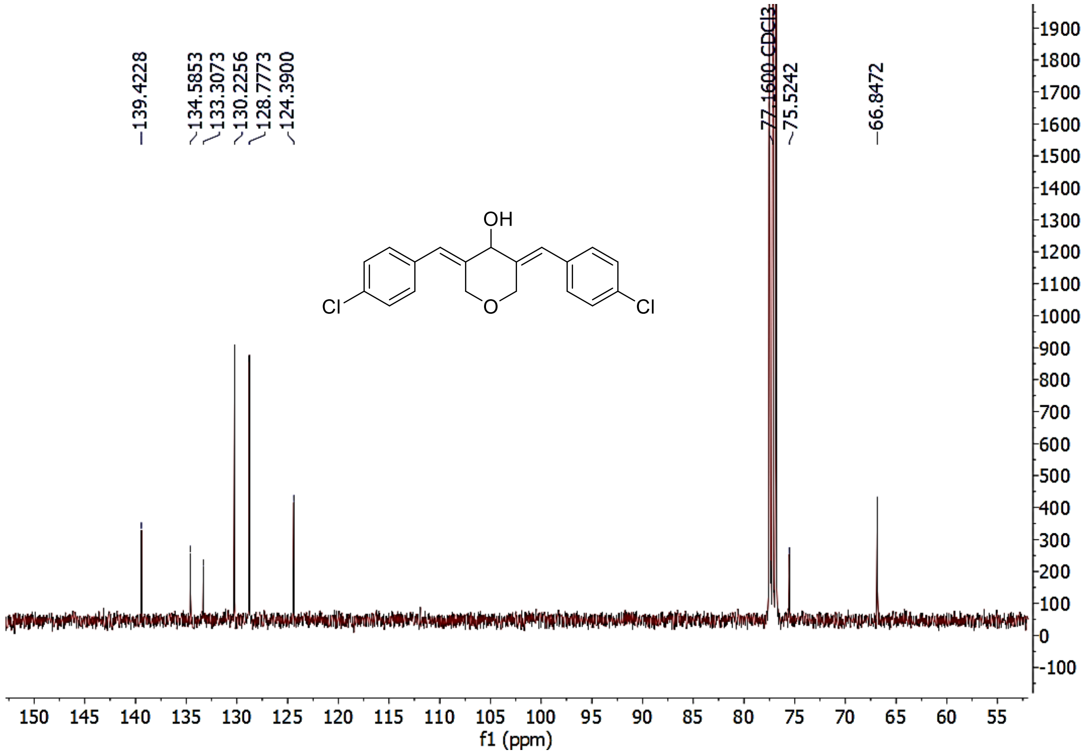

Figure S27. ^{1}H and ^{13}C NMR of compound **30**.

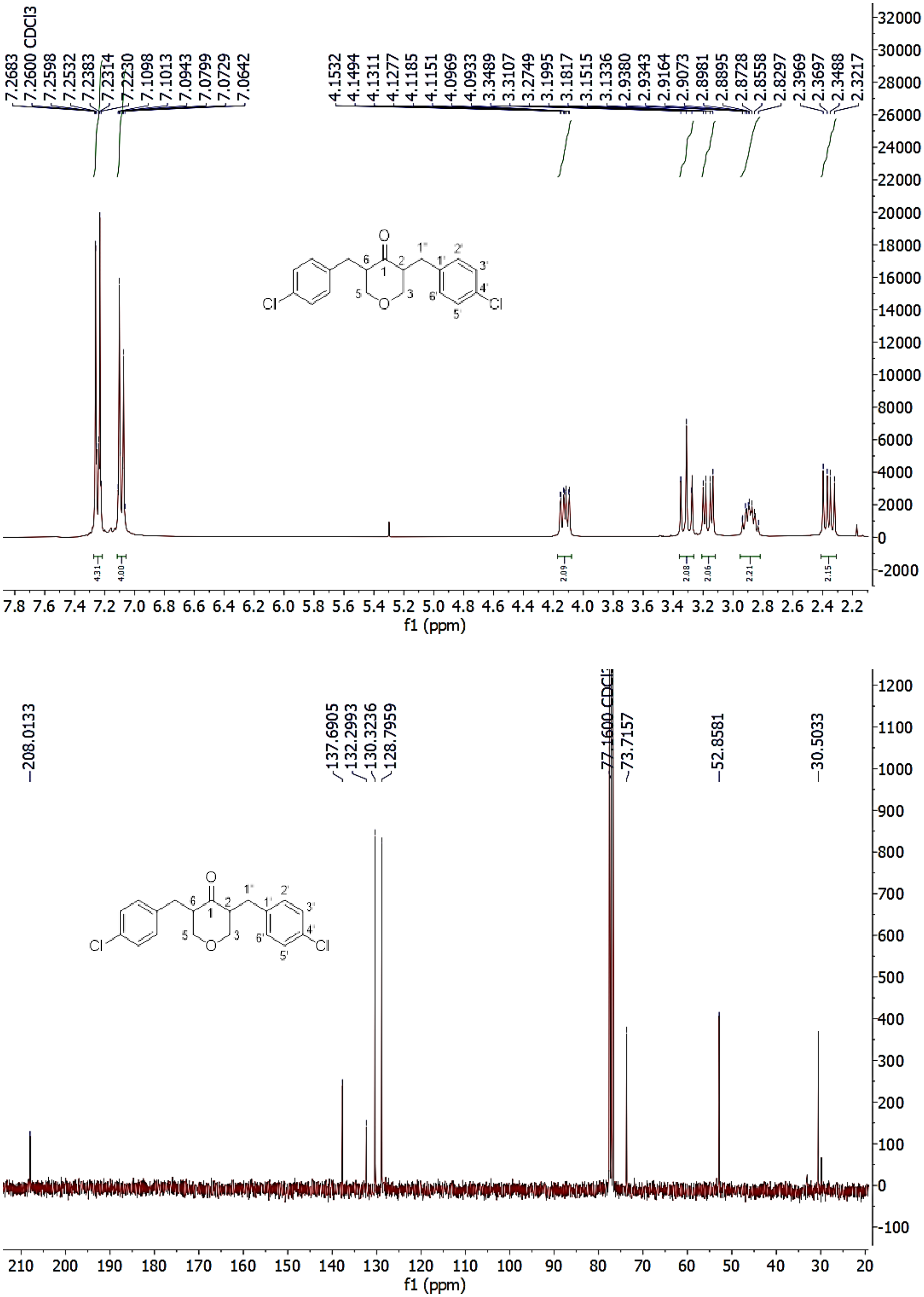

Figure S28. ^{1}H and ^{13}C NMR of compound **31**.

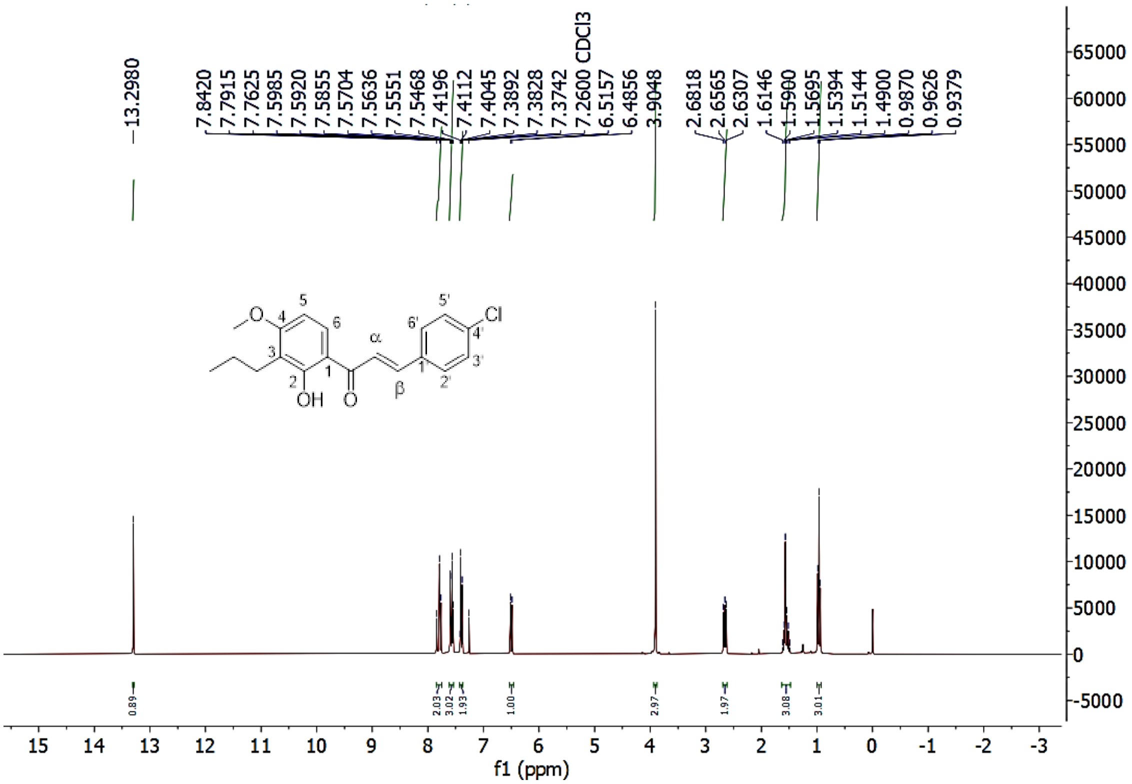

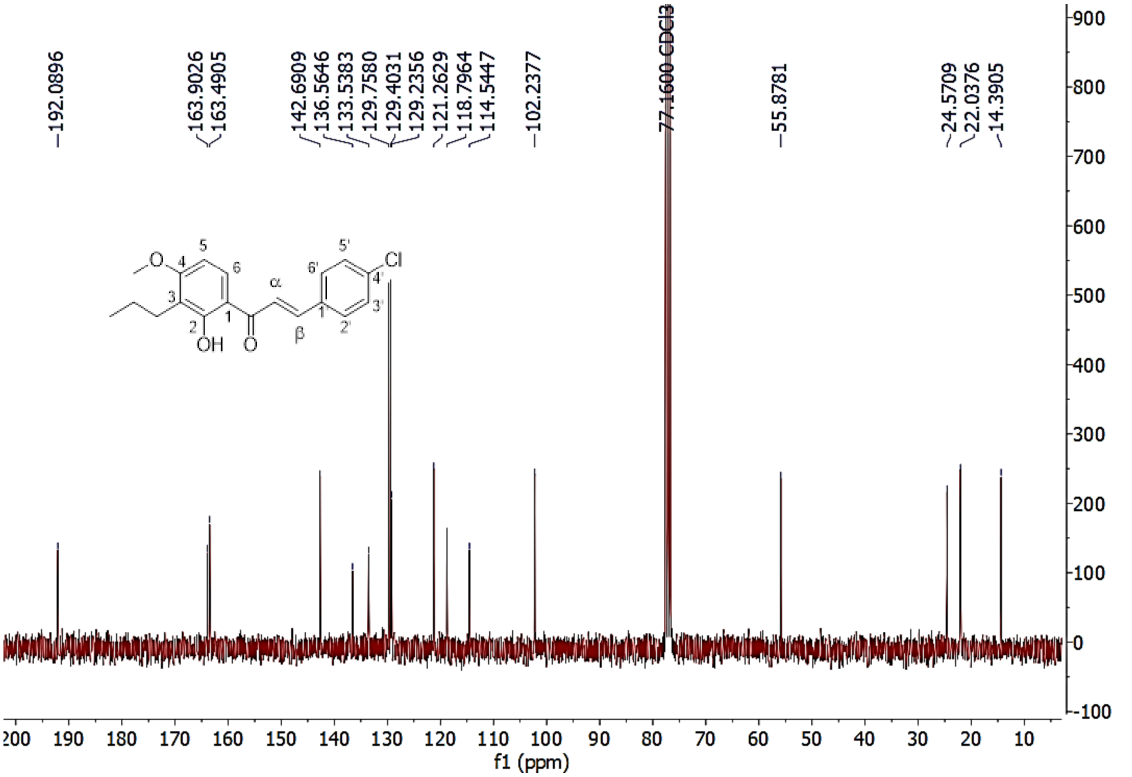

Figure S29. ^{1}H and ^{13}C NMR of compound **32**.

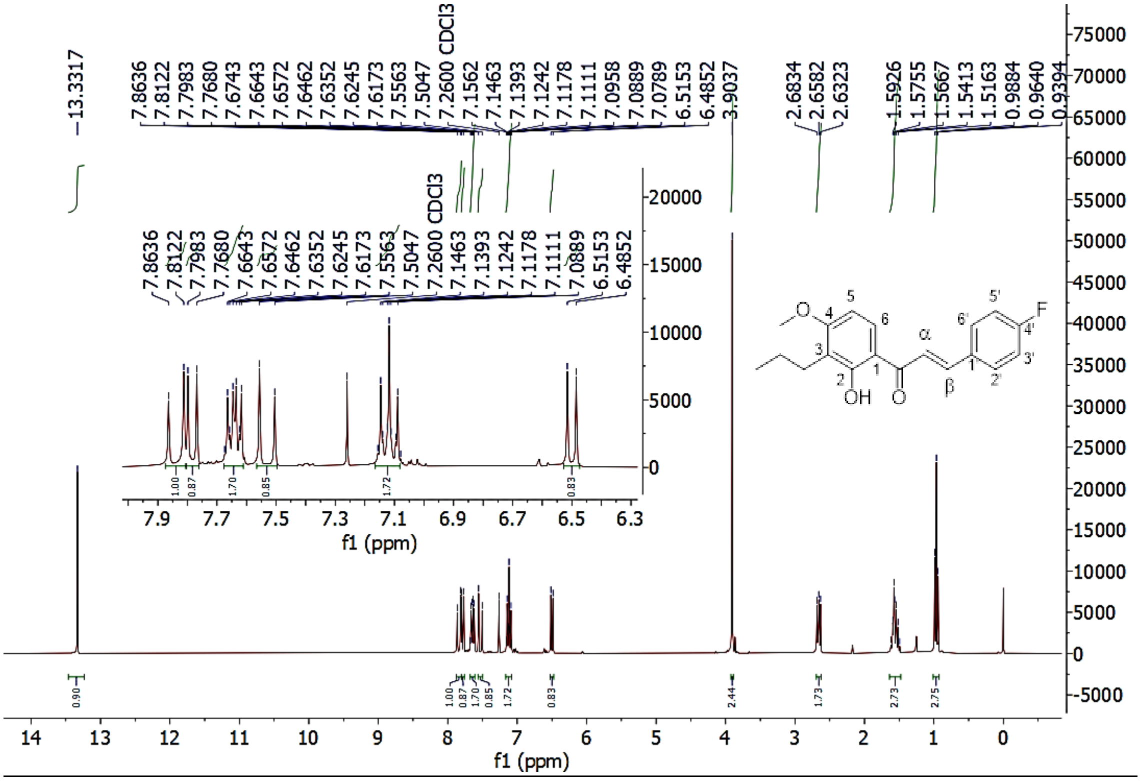

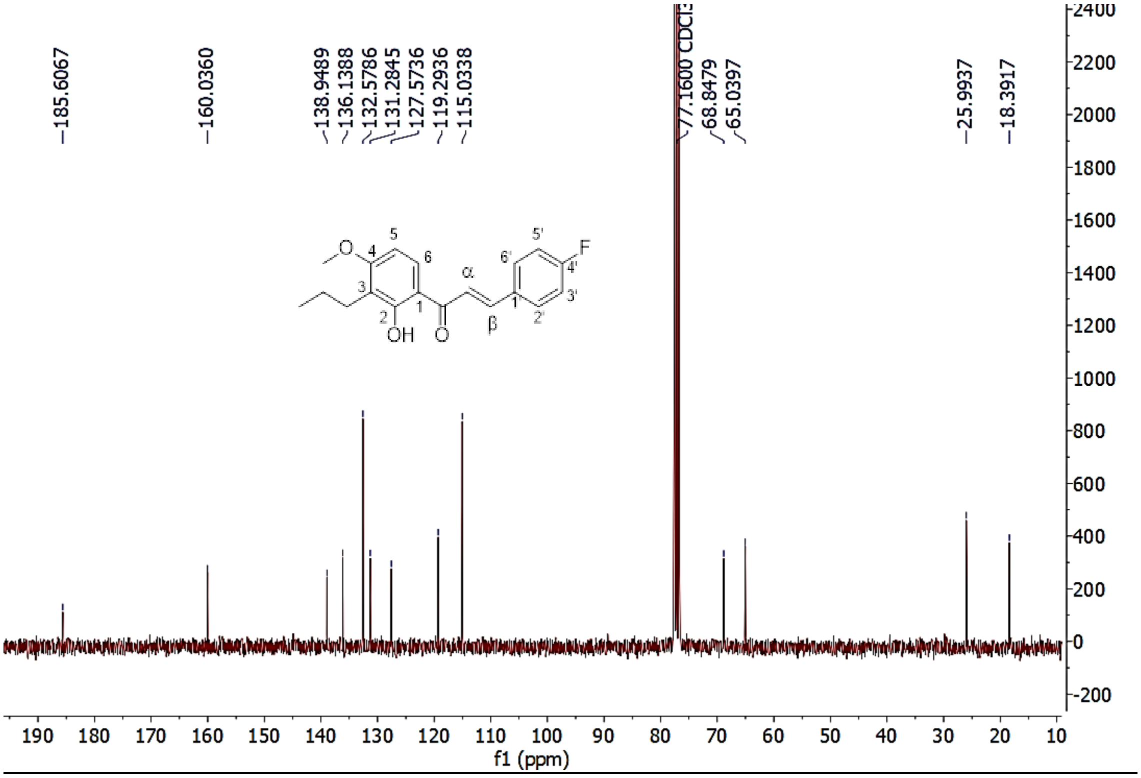

Figure S30. ^{1}H and ^{13}C NMR of compound **33**.

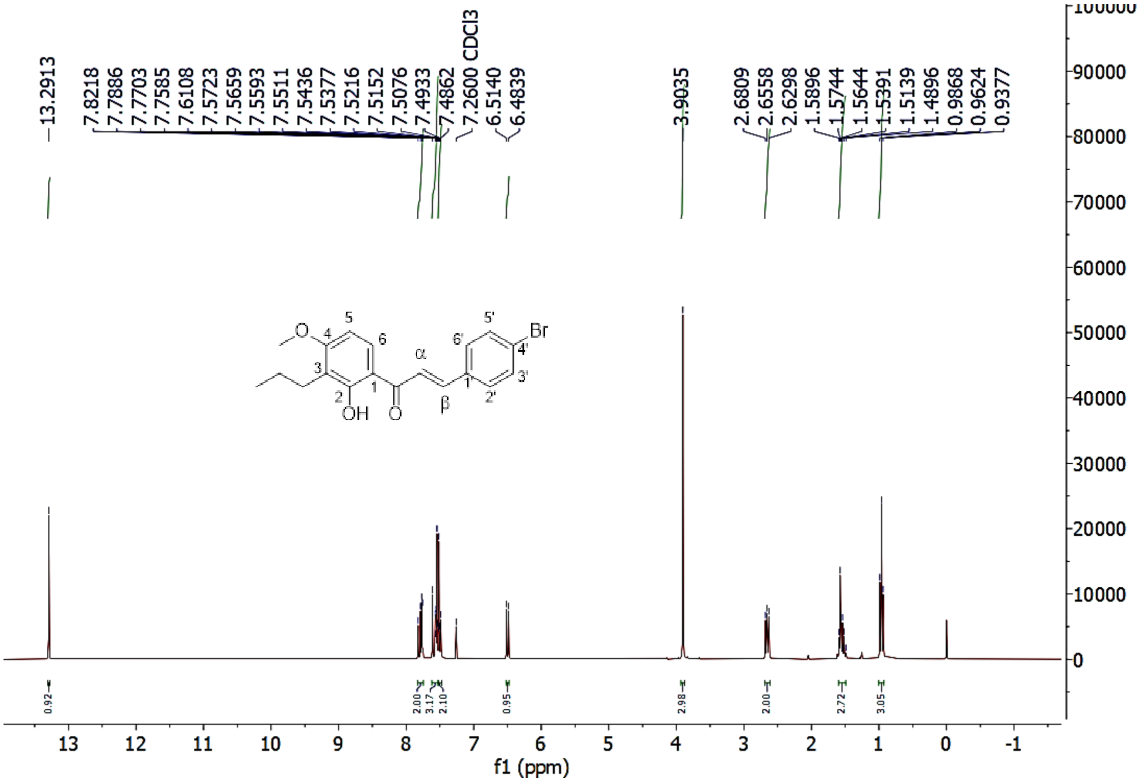

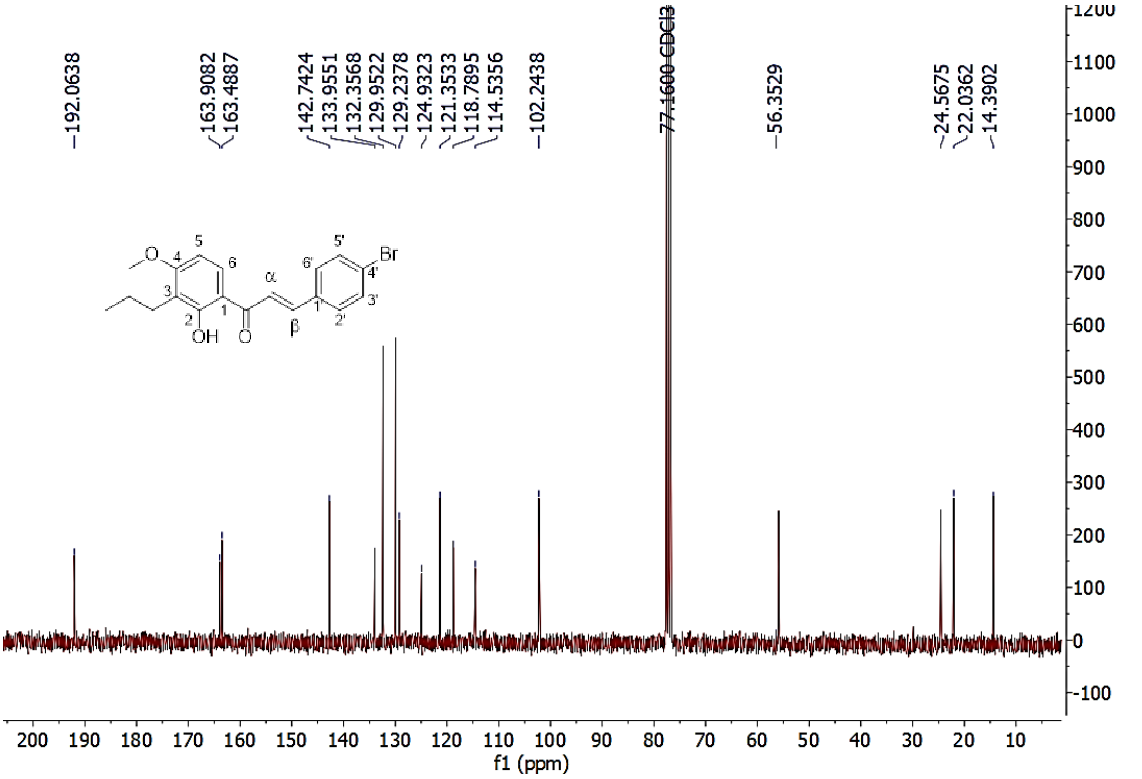

Figure S31. ^{1}H and ^{13}C NMR of compound **34**.

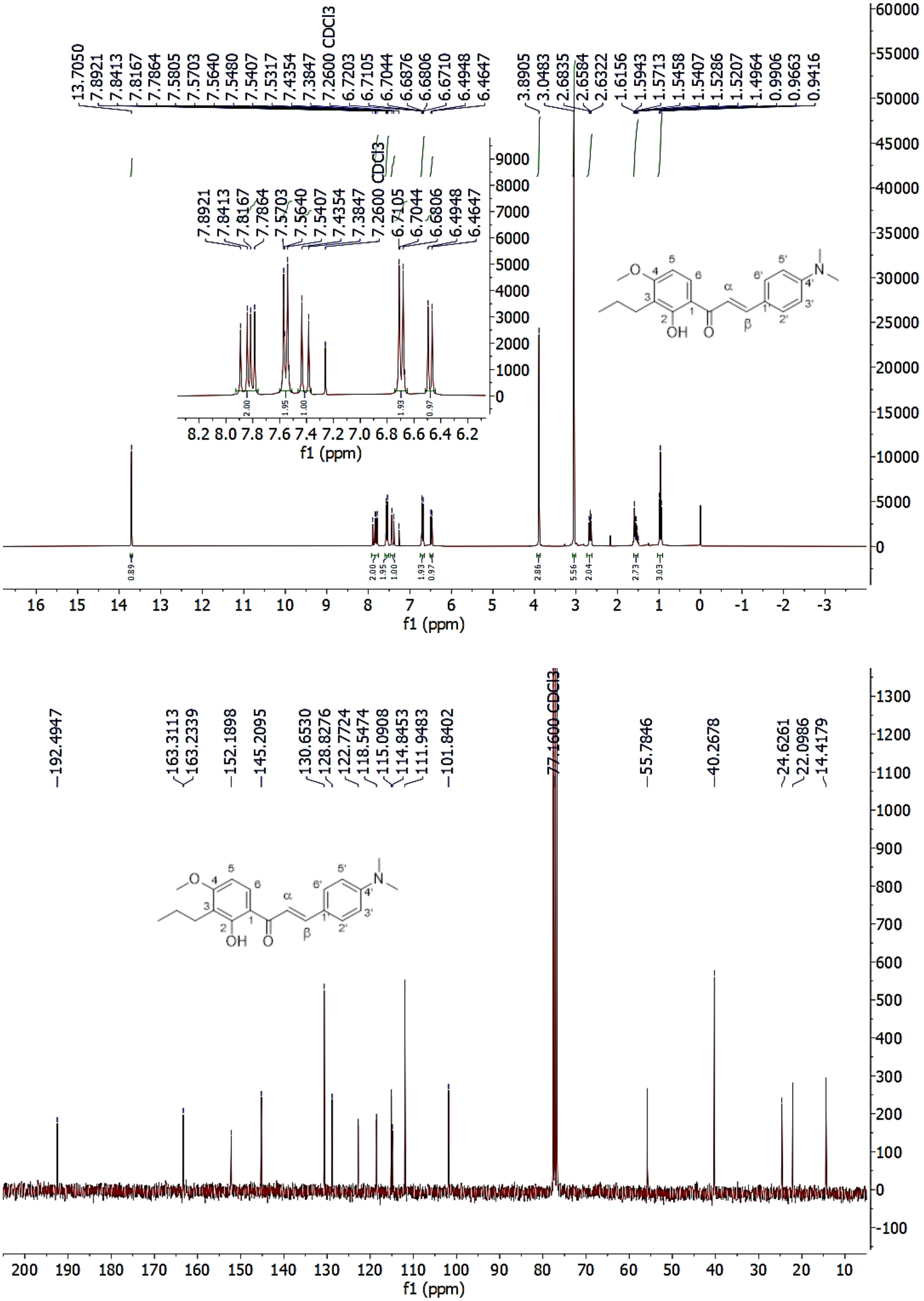

Figure S32. ^{1}H and ^{13}C NMR of compound **35**.

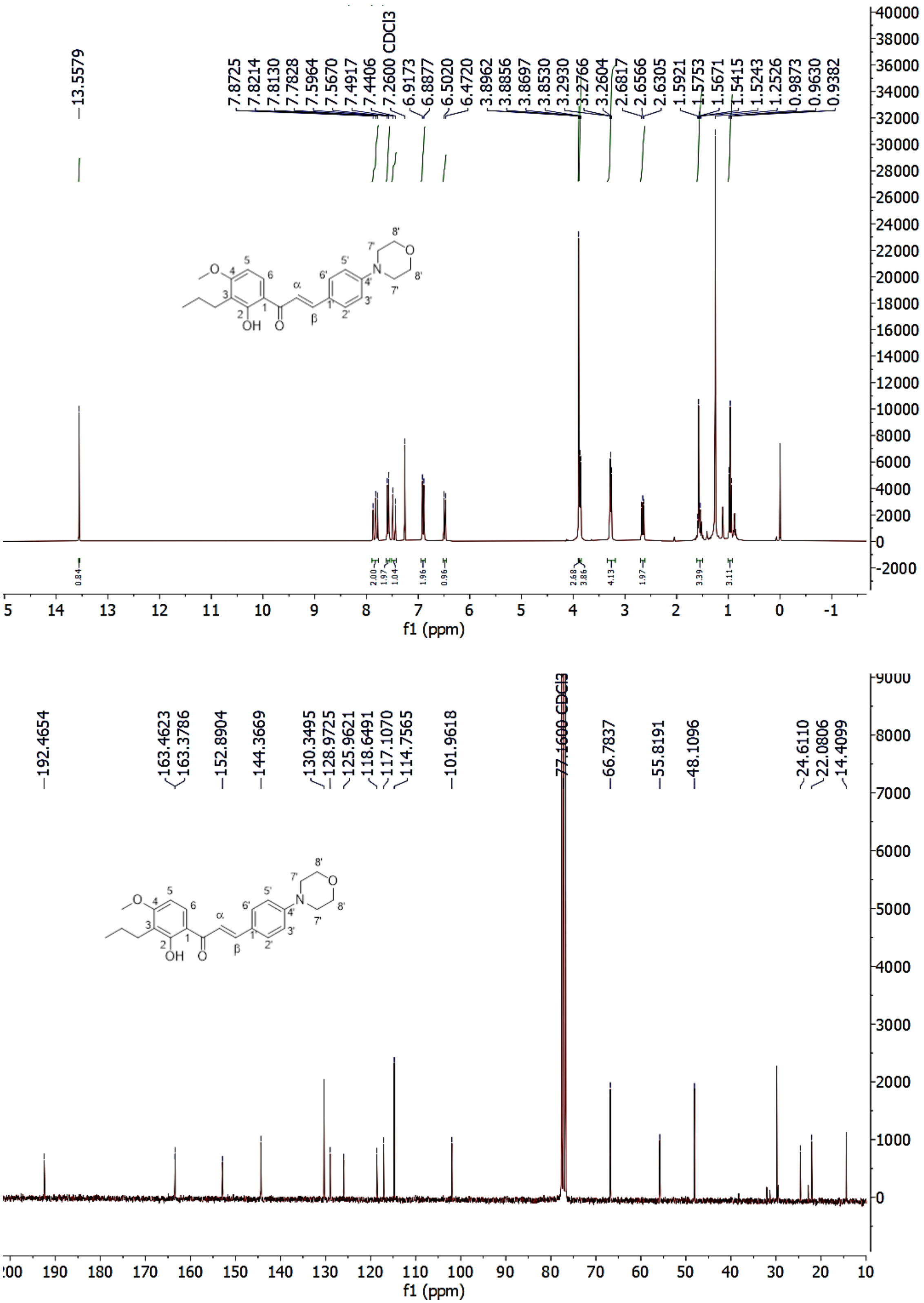

Figure S33. ^{1}H and ^{13}C NMR of compound **36**.

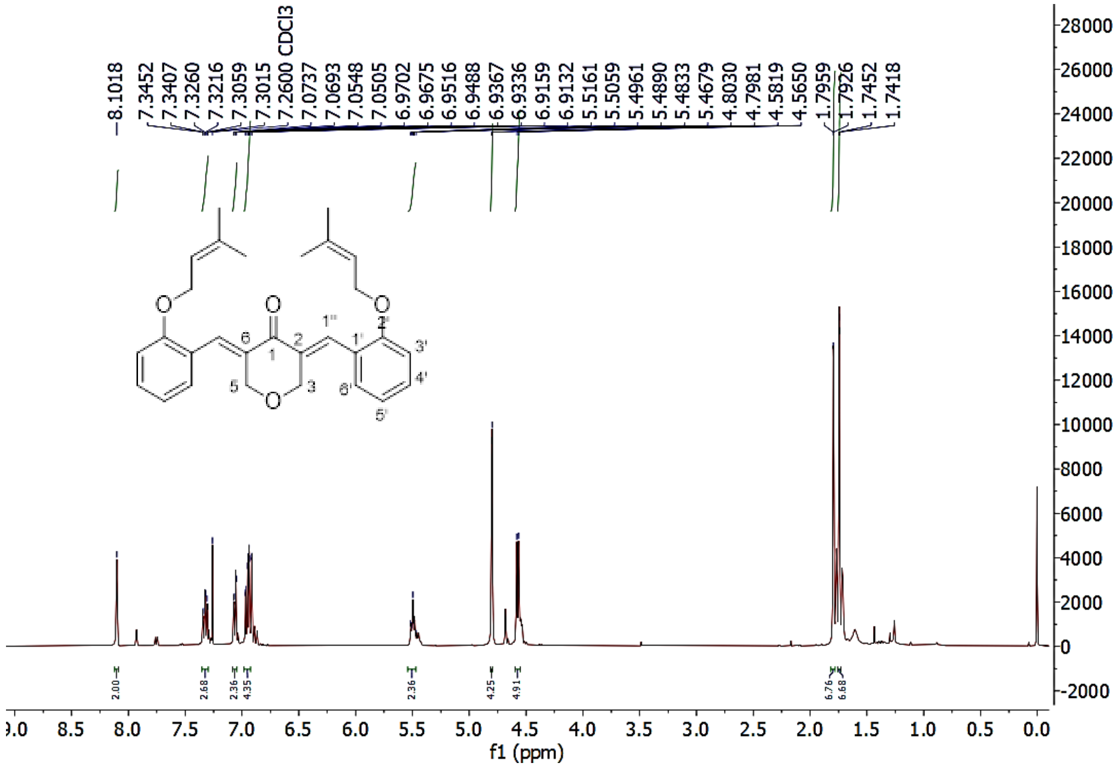

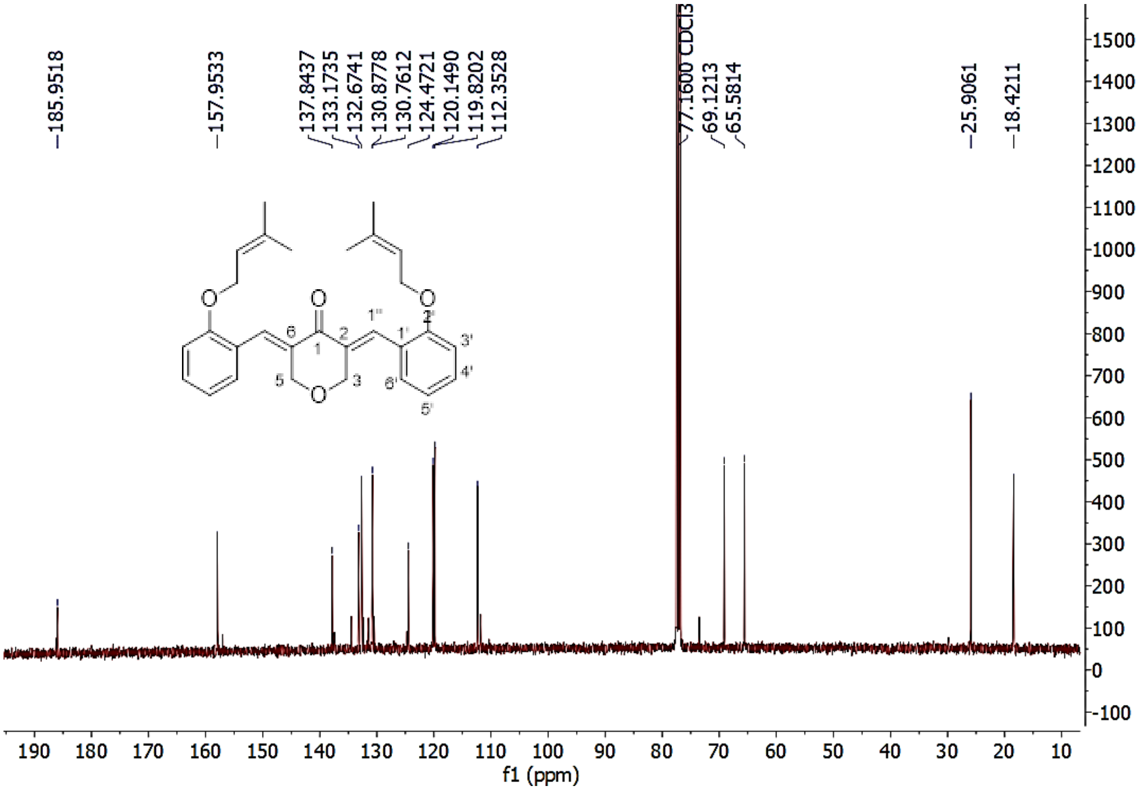

Figure S34. ^{1}H and ^{13}C NMR of compound **38**.

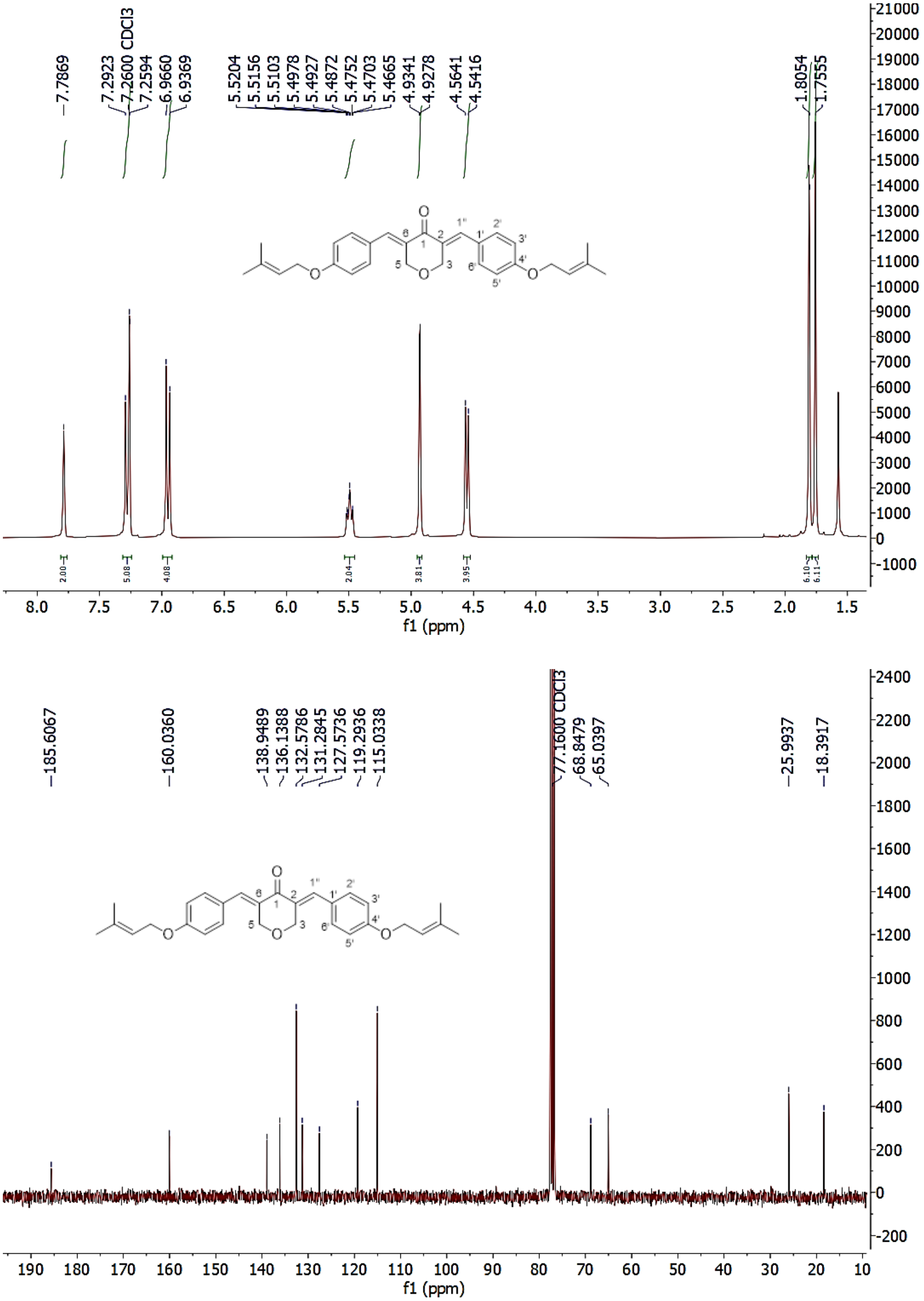

Figure S35. ^{1}H and ^{13}C NMR of compound **40**.

HRMS spectra

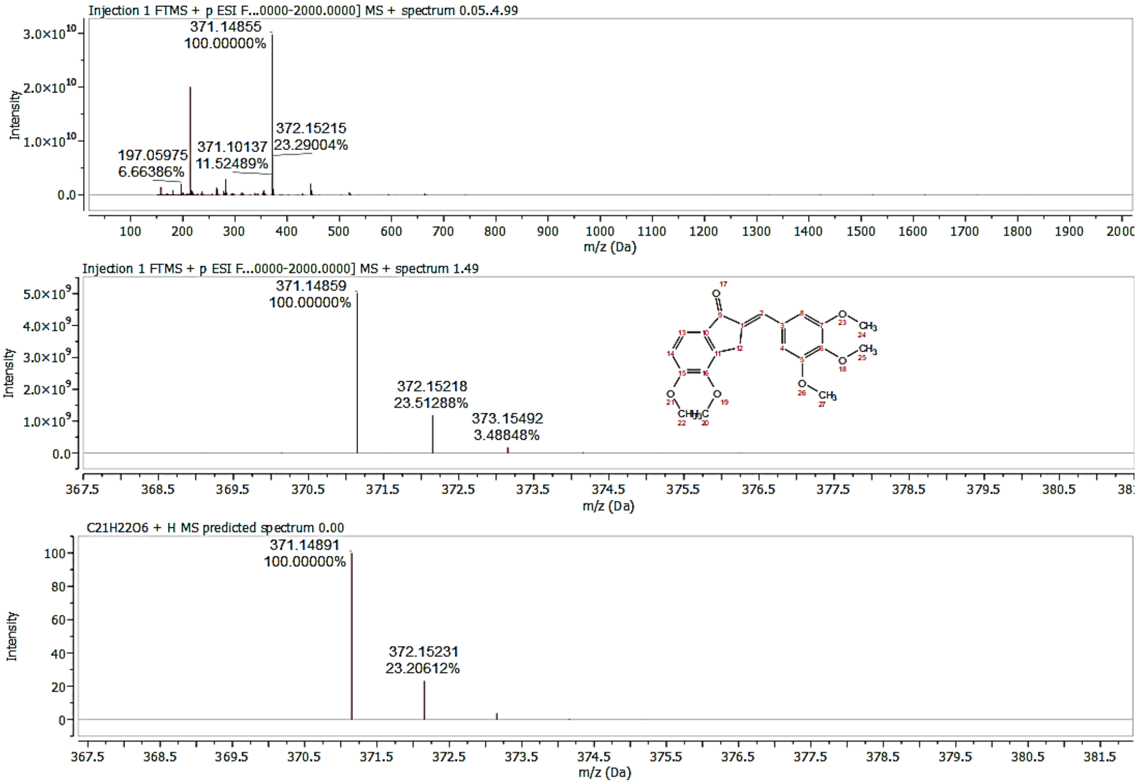

Meas. m/z	Ion formula	m/z	Err [ppm]	Err [mDa]
371.14859	$C_{21}H_{22}O_6$	371.14892	0.88	0.33

Figure S36. HRMS of compound **4**.

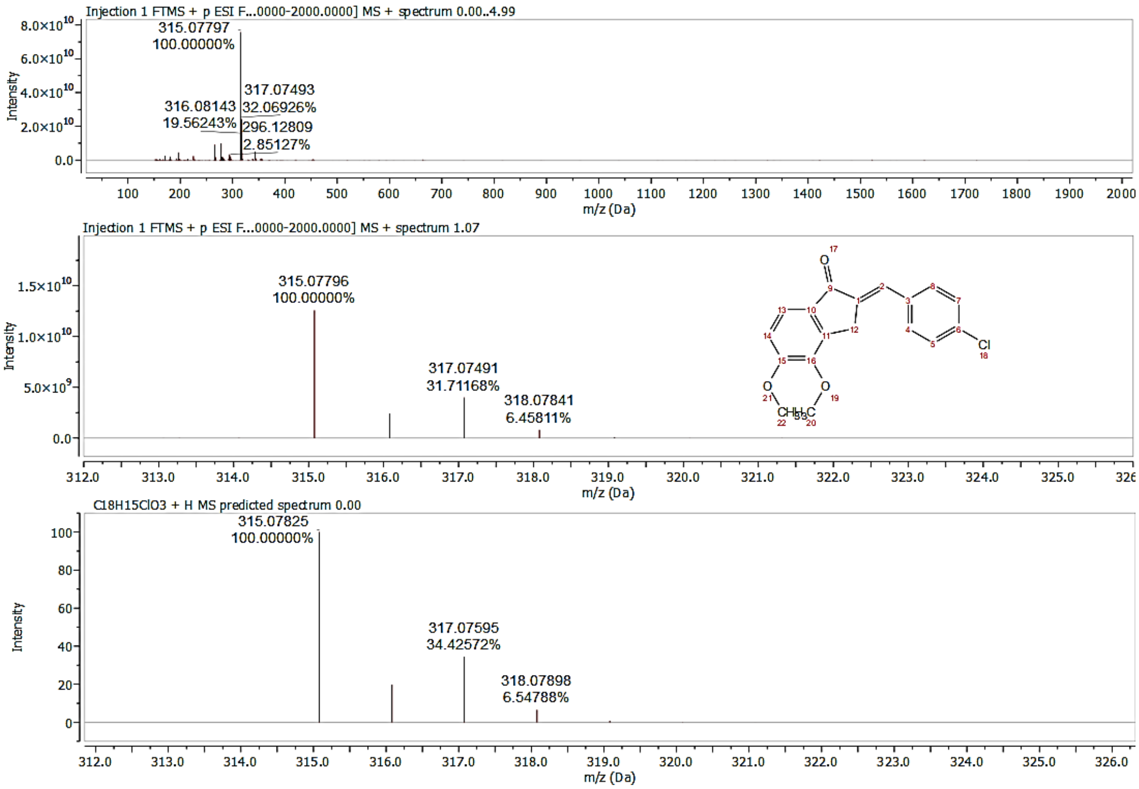

Meas. m/z	Ion formula	m/z	Err [ppm]	Err [mDa]
315.07798	$C_{18}H_{15}ClO_3$	315.07825	0.92	0.29

Figure S37. HRMS of compound **7**.

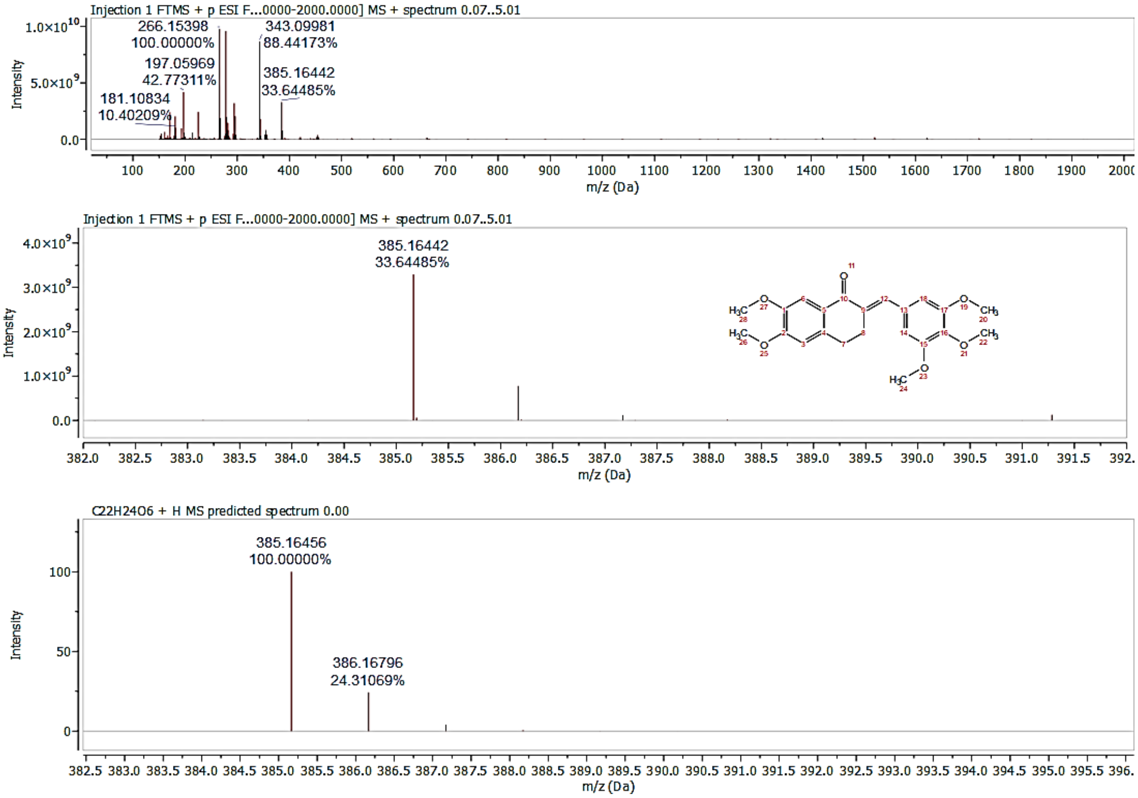

Meas. m/z	Ion formula	m/z	Err [ppm]	Err [mDa]
385.16442	$C_{22}H_{25}O_6$	385.16156	0.38	0.15

Figure S38. HRMS of compound **10**.

pharmaceuticals

MDPI

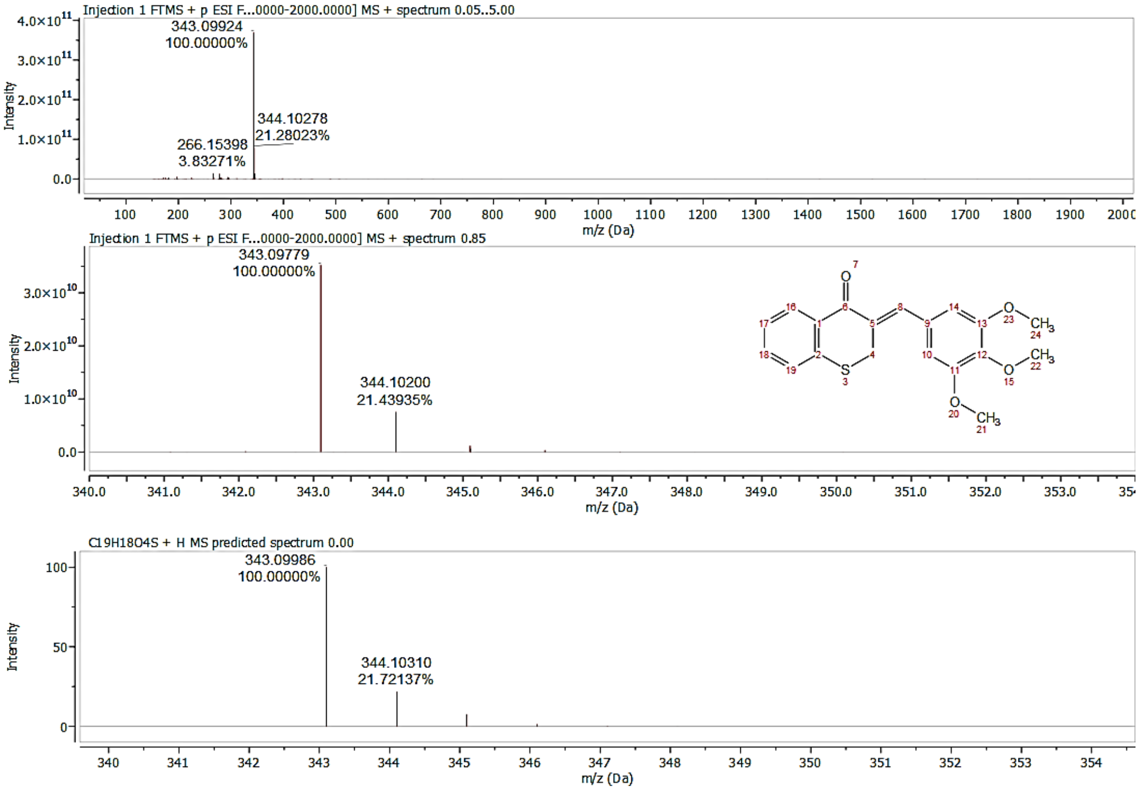

Meas. m/z	Ion formula	m/z	Err [ppm]	Err [mDa]
343.09779	$C_{19}H_{18}O_4S$	343.09986	1.80	0.62

Figure S39. HRMS of compound **12**.

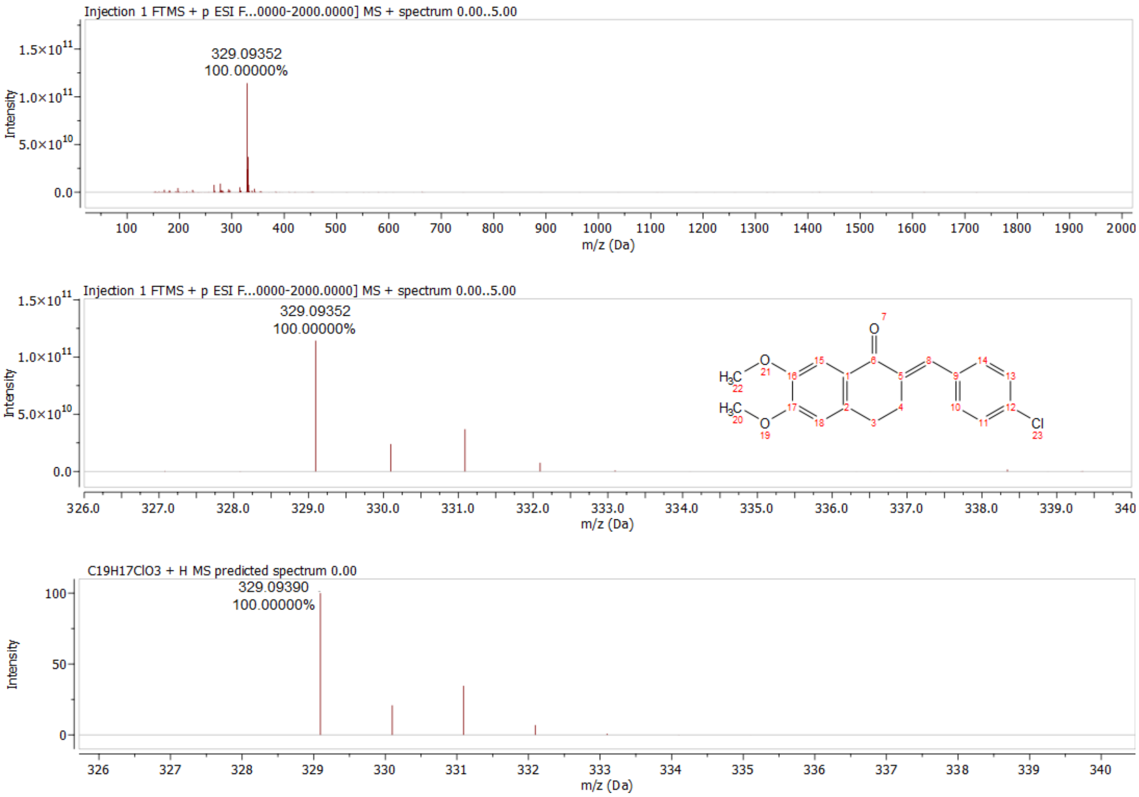

Meas. m/z	Ion formula	m/z	Err [ppm]	Err [mDa]
329.09352	$C_{19}H_{17}ClO_3$	329.09390	1.15	0.38

Figure S40. HRMS of compound **14**.

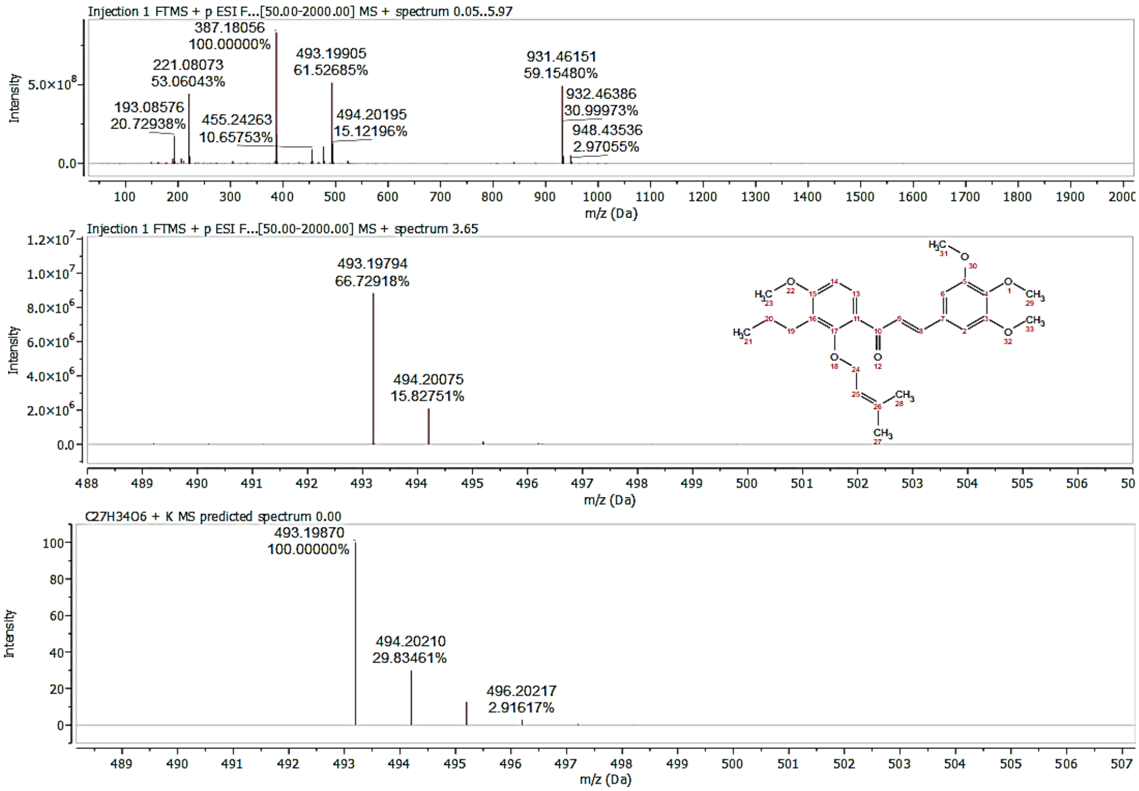

Meas. m/z	Ion formula	m/z	Err [ppm]	Err [mDa]
493.19794	$C_{27}H_{34}O_6$	493.19870	1.54	0.76

Figure S41. HRMS of compound **26**.

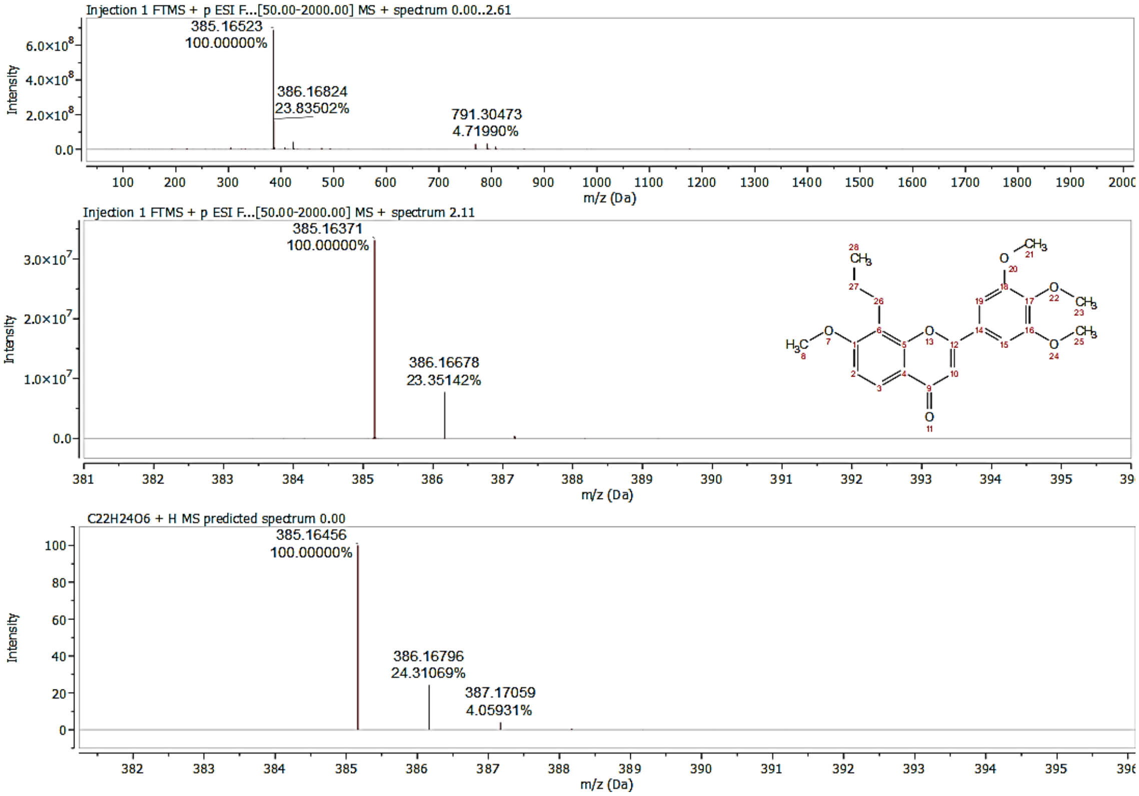

Meas. m/z	Ion formula	m/z	Err [ppm]	Err [mDa]
385.16371	$C_{22}H_{24}O_6$	385.16456	2.22	0.86

Figure S42. HRMS of compound **27**.

pharmaceuticals MDPI

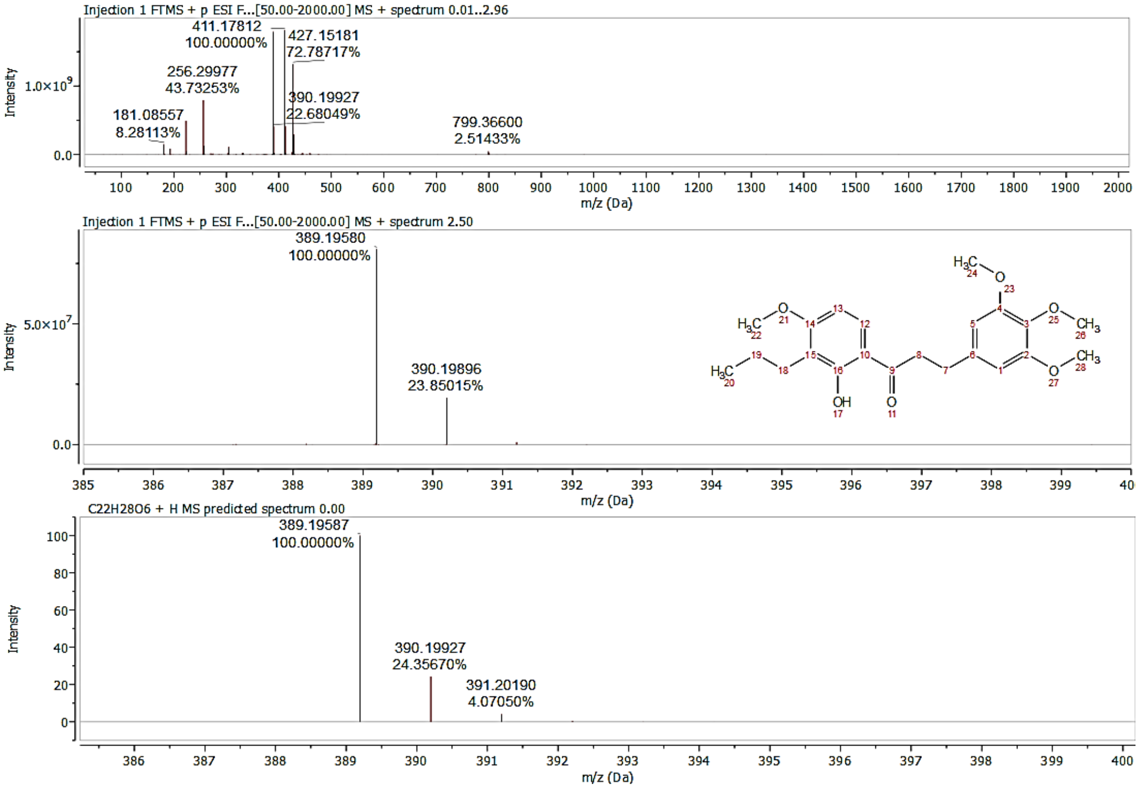

Meas. m/z	Ion formula	m/z	Err [ppm]	Err [mDa]
389.19580	$C_{22}H_{28}O_6$	389.19587	0.17	0.07

Figure S43. HRMS of compound **29**.

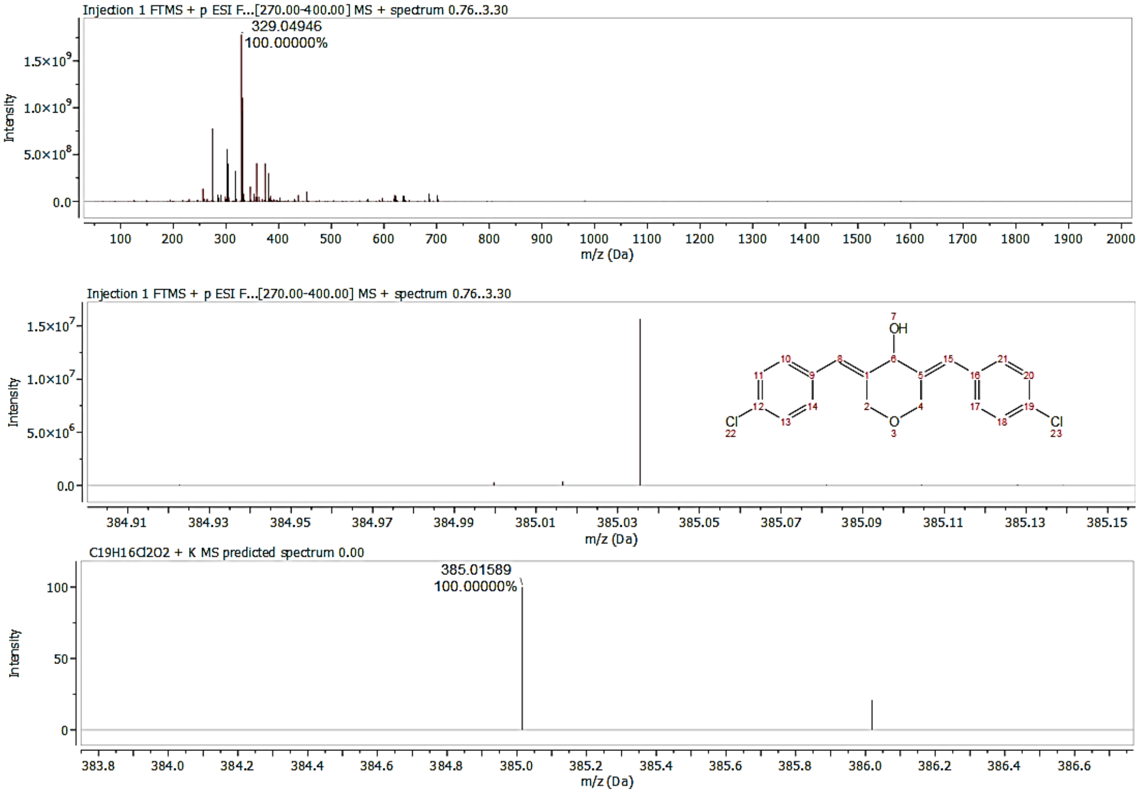

Meas. m/z	Ion formula	m/z	Err [ppm]	Err [mDa]
385.01545	$C_{19}H_{16}Cl_2O_2$	385.01598	1.15	0.44

Figure S44. HRMS of compound **30**.

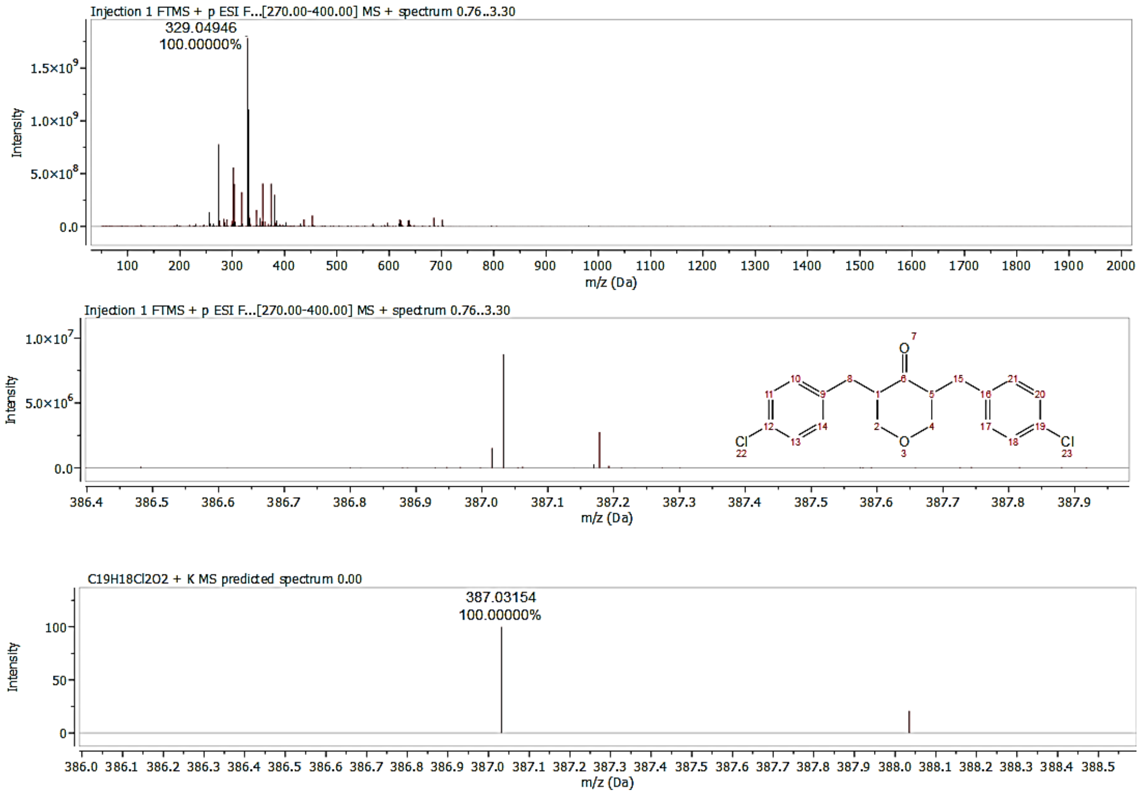

Meas. m/z	Ion formula	m/z	Err [ppm]	Err [mDa]
387.03260	$C_{19}H_{18}Cl_2O_2$	387.03154	-2.73	-1.06

Figure S45. HRMS of compound **31**.

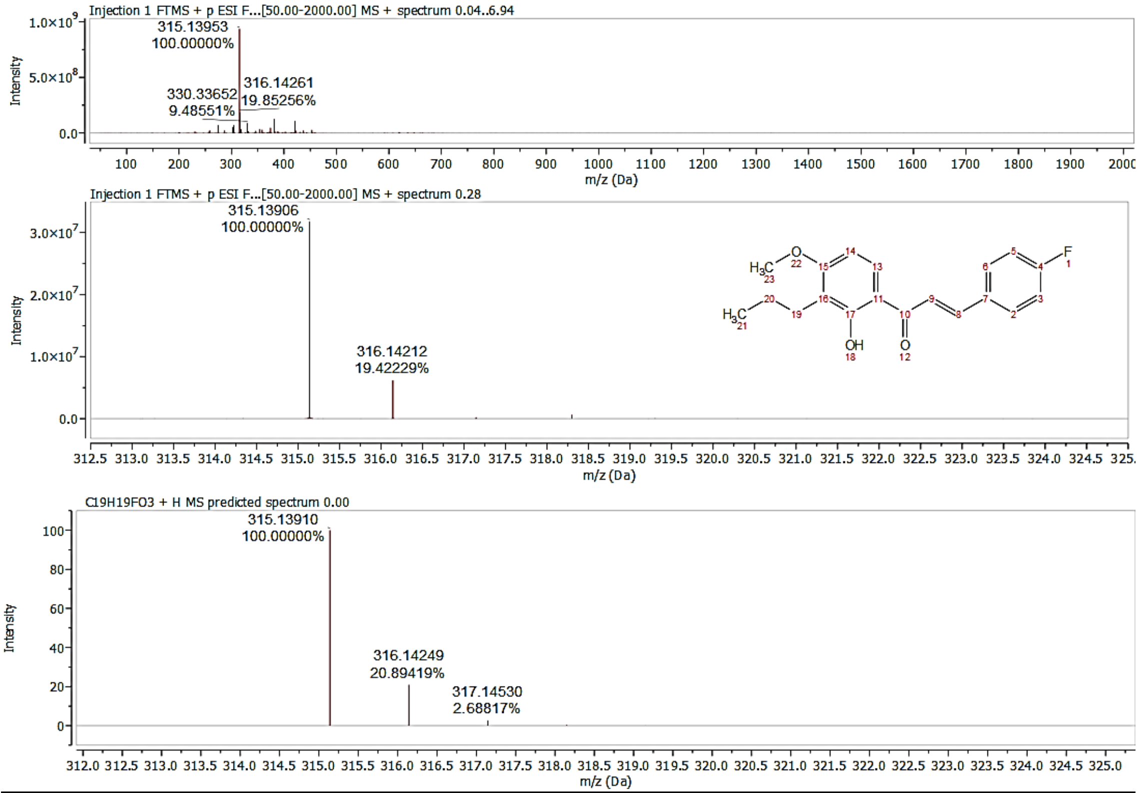

Meas. m/z	Ion formula	m/z	Err [ppm]	Err [mDa]
315.13906	$C_{19}H_{19}FO_3$	315.13910	0.13	0.04

Figure S46. HRMS of compound **33**.

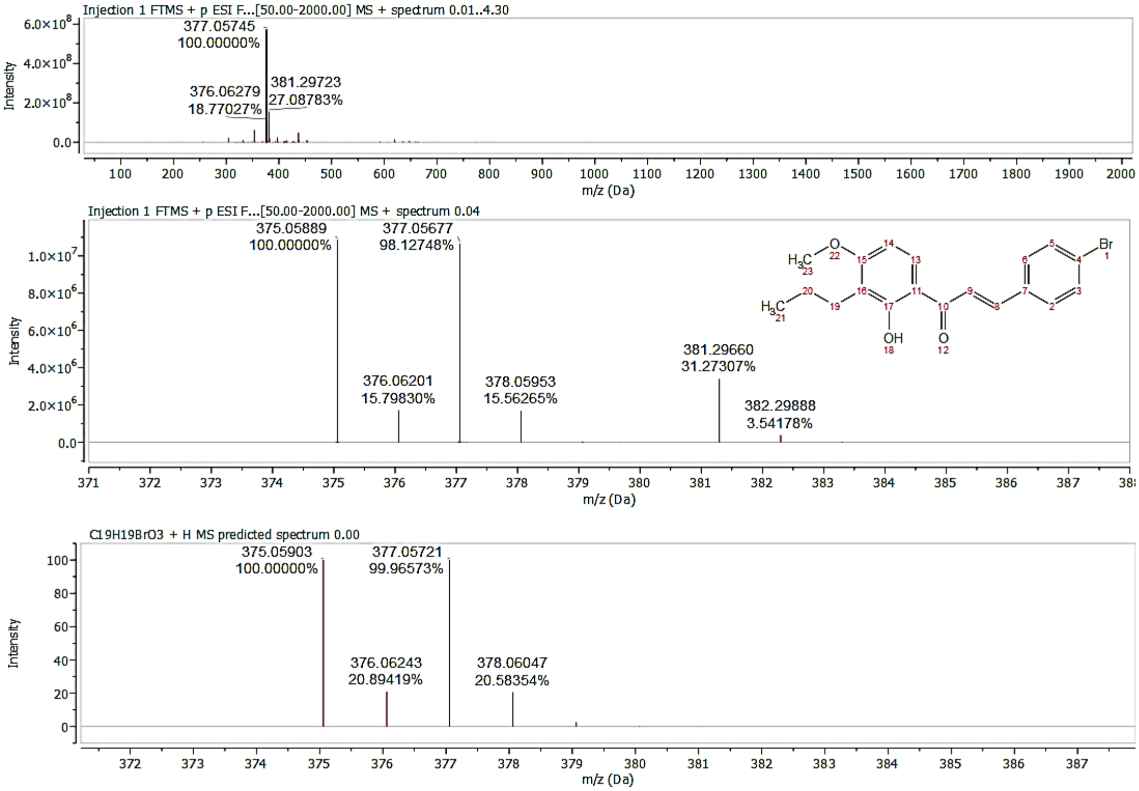

Meas. m/z	Ion formula	m/z	Err [ppm]	Err [mDa]
375.05889	$C_{19}H_{19}BrO_3$	375.05903	0.38	0.14

Figure S47. HRMS of compound **34**.

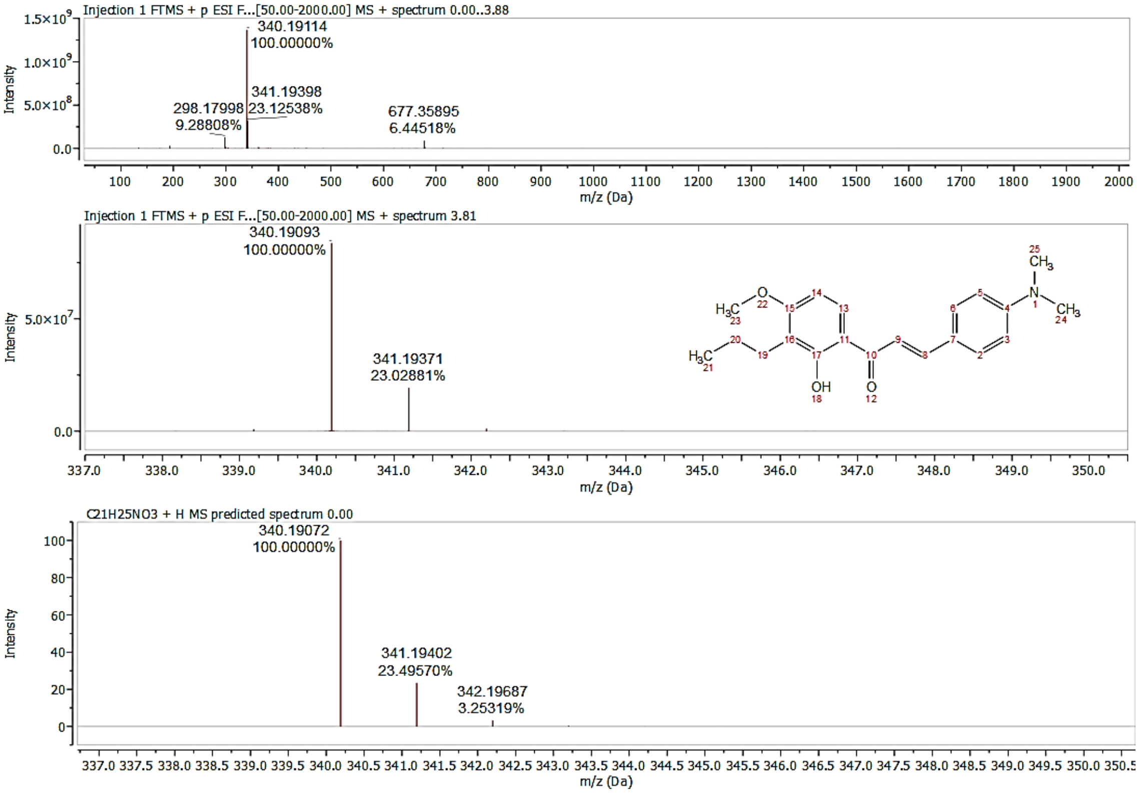

Meas. m/z	Ion formula	m/z	Err [ppm]	Err [mDa]
340.19093	$C_{21}H_{25}NO_3$	340.19072	-0.62	-0.21

Figure S48. HRMS of compound **35**.

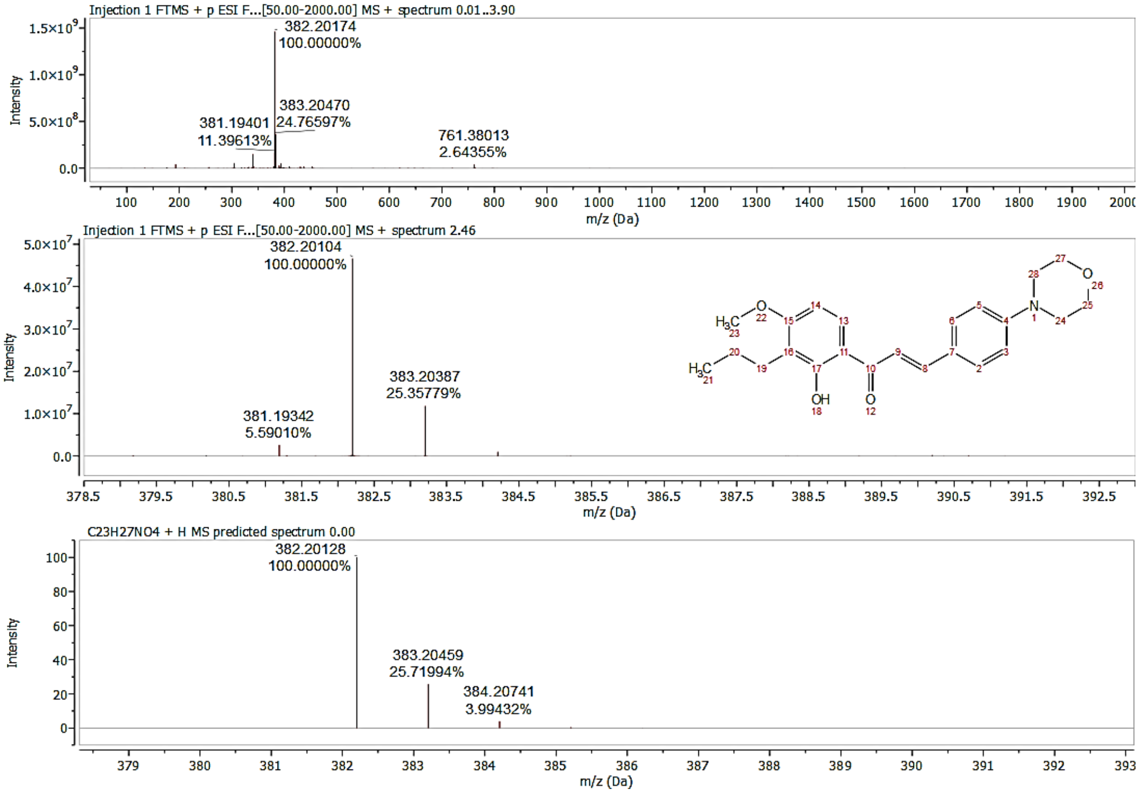

Meas. m/z	Ion formula	m/z	Err [ppm]	Err [mDa]
382.20104	$C_{23}H_{27}NO_4$	382.20128	0.64	0.25

Figure S49. HRMS of compound **36**.

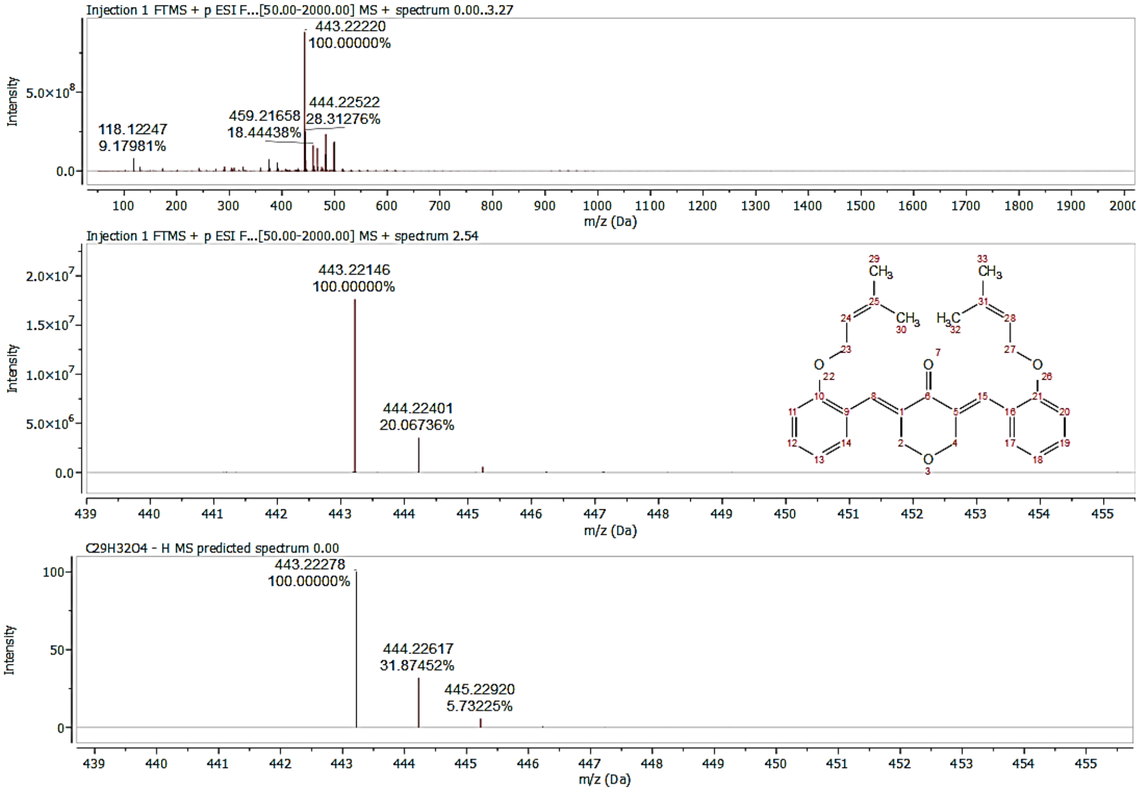

Meas. m/z	Ion formula	m/z	Err [ppm]	Err [mDa]
443.22146	$C_{29}H_{32}O_4$	443.22278	2.99	1.32

Figure S50. HRMS of compound **38**.

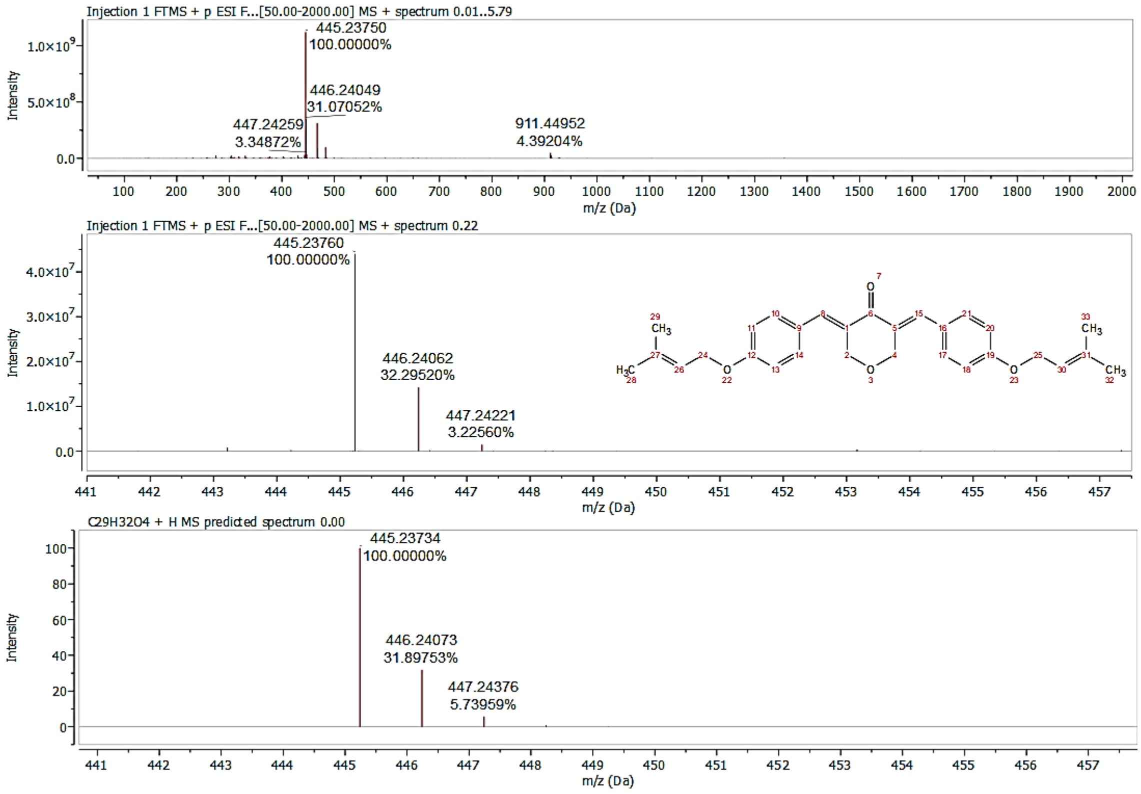

Meas. m/z	Ion formula	m/z	Err [ppm]	Err [mDa]
445.23760	$C_{29}H_{32}O_4$	445.23734	-0.59	-0.26

Figure S51. HRMS of compound **40**.

HPLC chromatograms

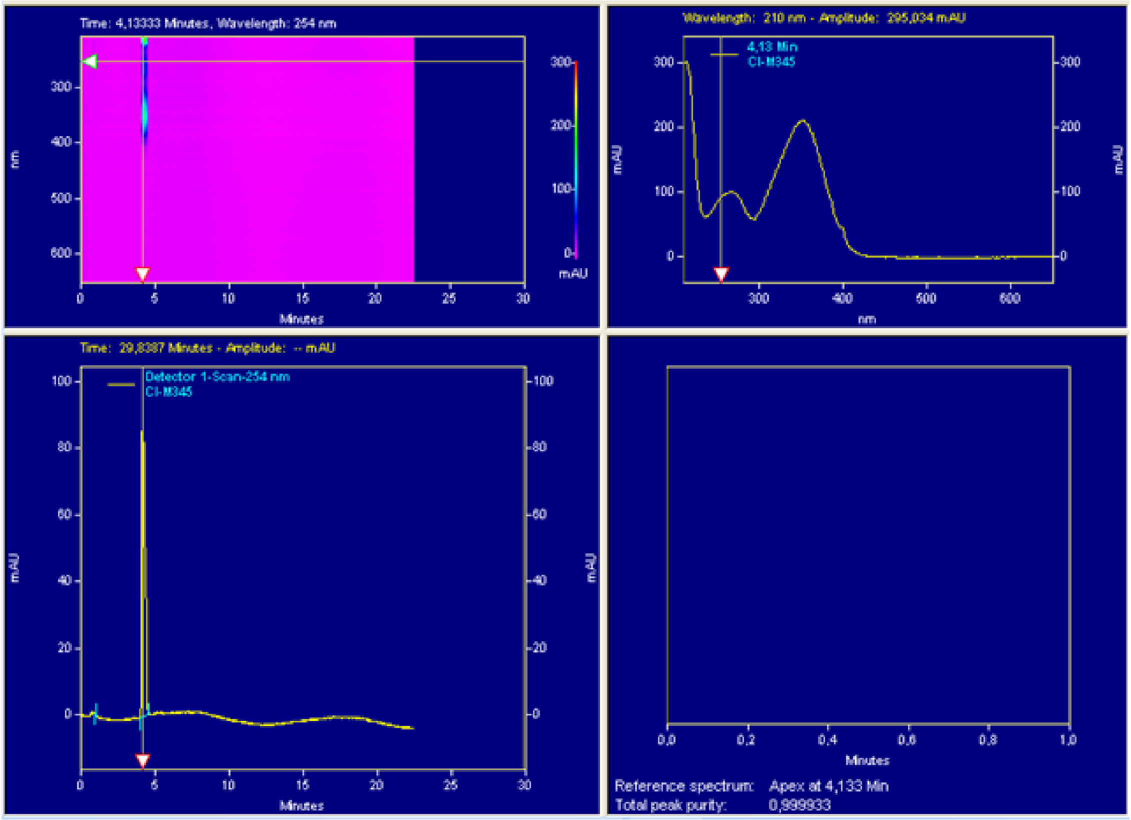

Retention Time	Area	Area %	Height	Height %
0.900	1424	0.10	372	0.40
4.133	1391952	99.90	92997	99.60
Totals	1393376	100.00	93369	100.00

Figure S52. HPLC chromatograms of compound **3**.

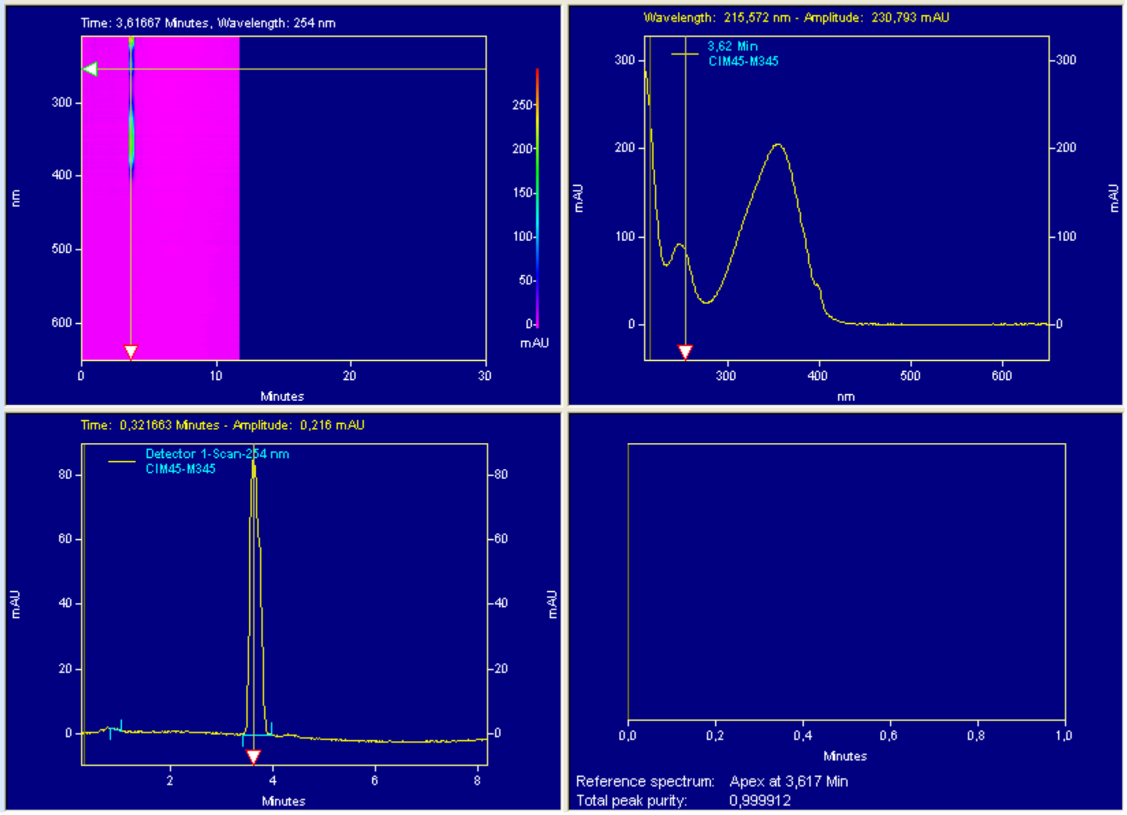

Retention Time	Area	Area %	Height	Height %
0.850	2616	0.23	195	0.23
3.617	1142225	99.77	85221	99.77
Totals	1144841	100.00	85416	100.00

Figure S53. HPLC chromatograms of compound **4**.

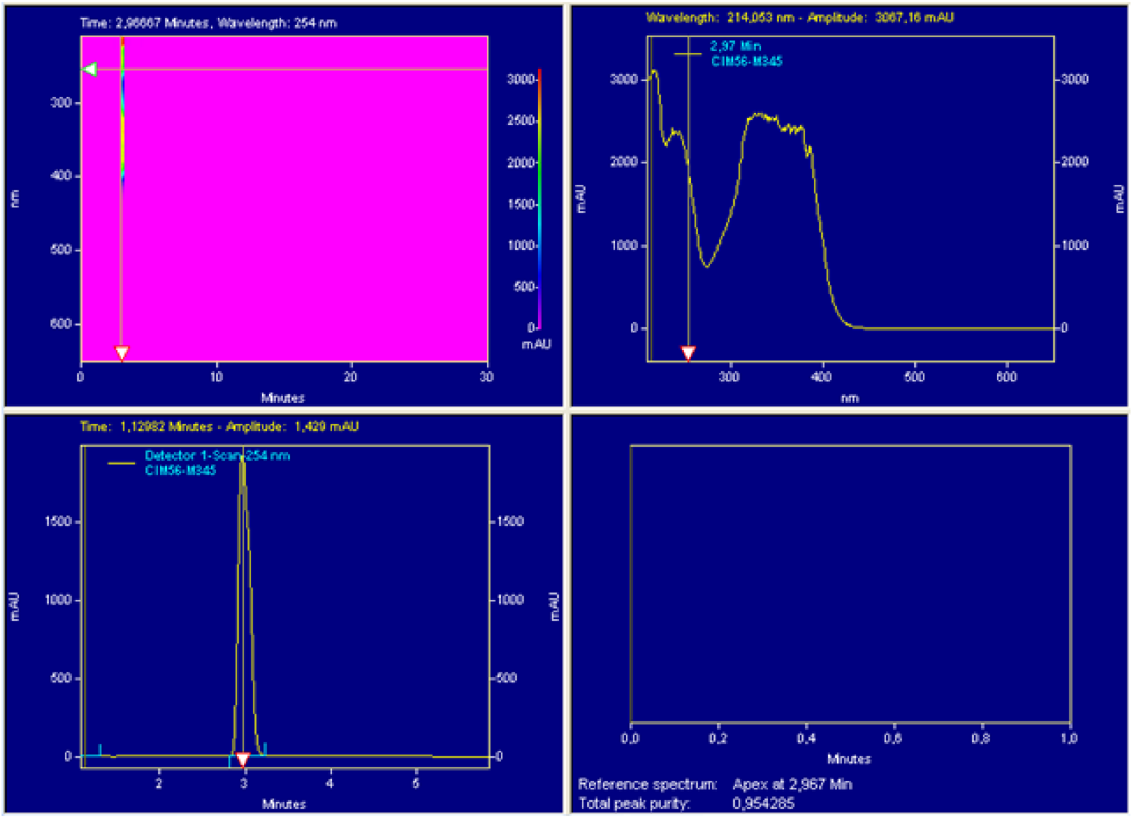

Retention Time	Area	Area %	Height	Height %
1.000	49480	0.29	1710	0.09
2.967	16814680	99.71	1948883	99.91
Totals	16864160	100.00	1950593	100.00

Figure S54. HPLC chromatograms of compound **5**.

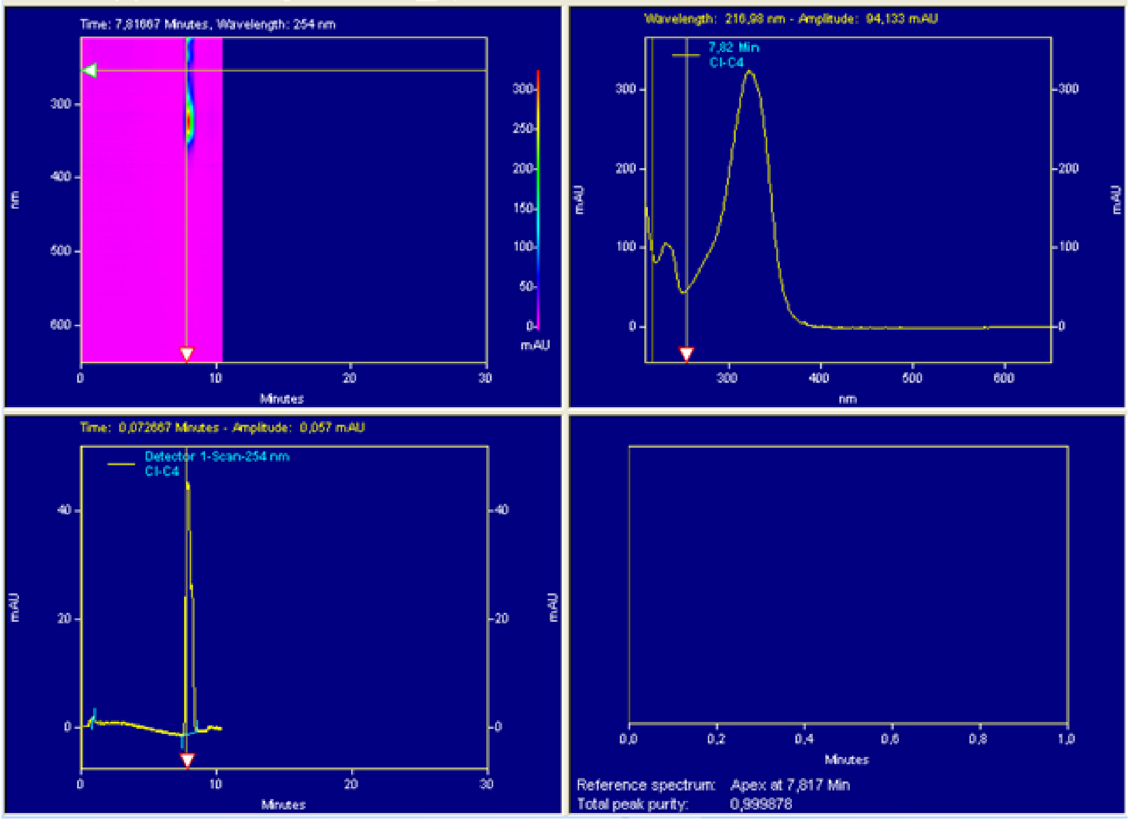

Retention Time	Area	Area %	Height	Height %
0.917	2313	0.17	576	1.20
7.817	1367307	99.83	47333	98.80
Totals	1369620	100.00	47909	100.00

Figure S55. HPLC chromatograms of compound **6**.

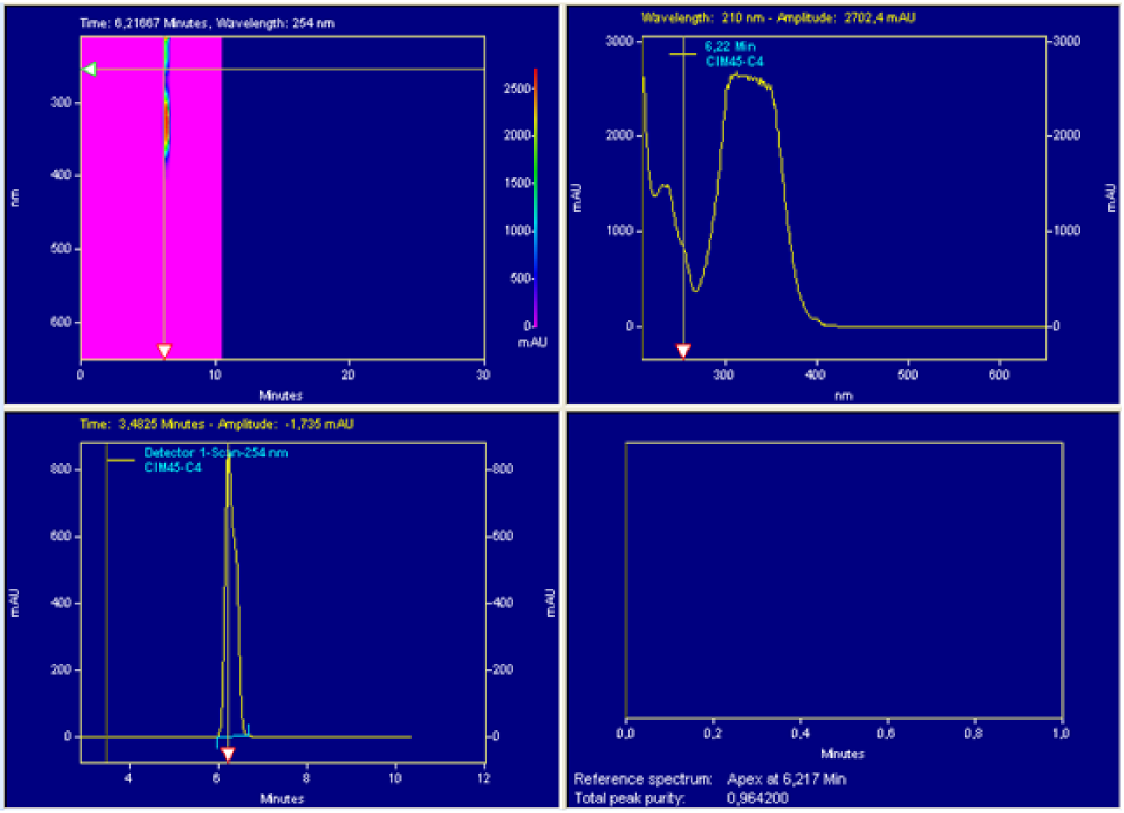

Retention Time	Area	Area %	Height	Height %
0.900	3303	0.02	714	0.08
6.217	14209543	99.98	846375	99.92
Totals	14212846	100.00	847089	100.00

Figure S56. HPLC chromatograms of compound **7**.

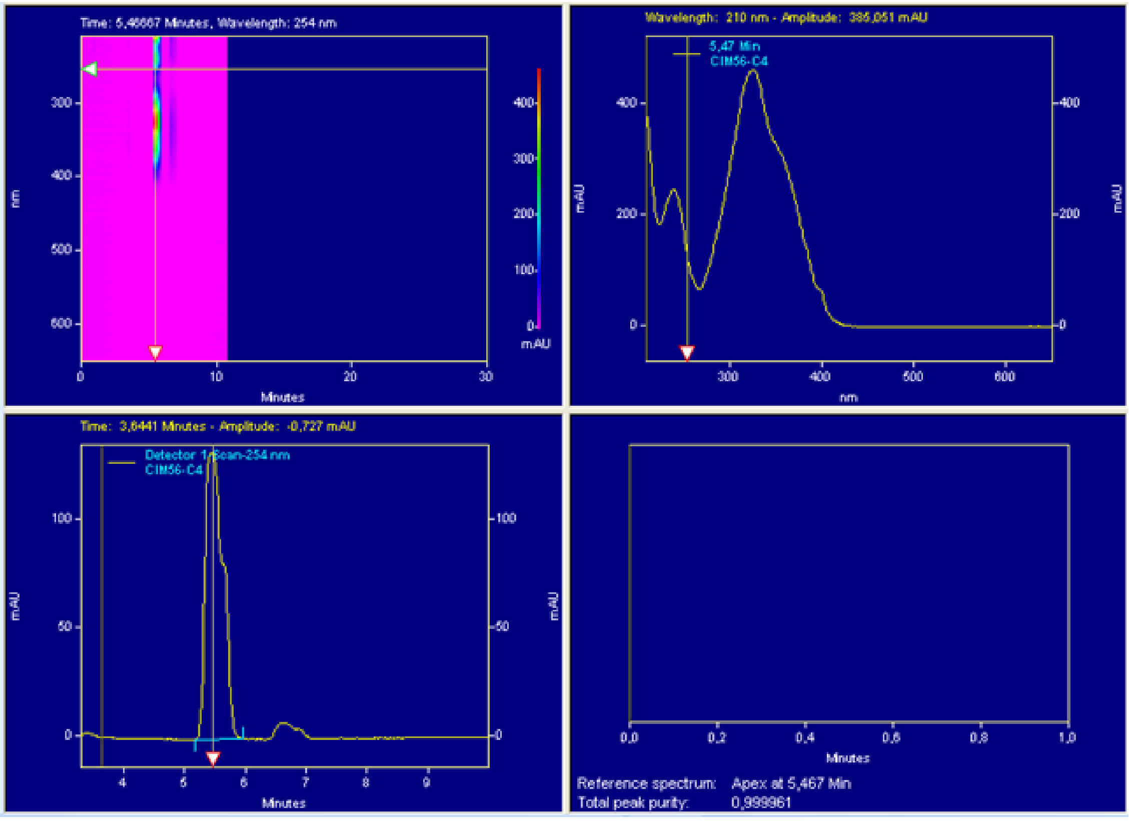

Retention Time	Area	Area %	Height	Height %
0.900	3149	0.12	393	0.30
5.467	2700329	99.88	132323	99.70
Totals	2703478	100.00	132716	100.00

Figure S57. HPLC chromatograms of compound **8**.

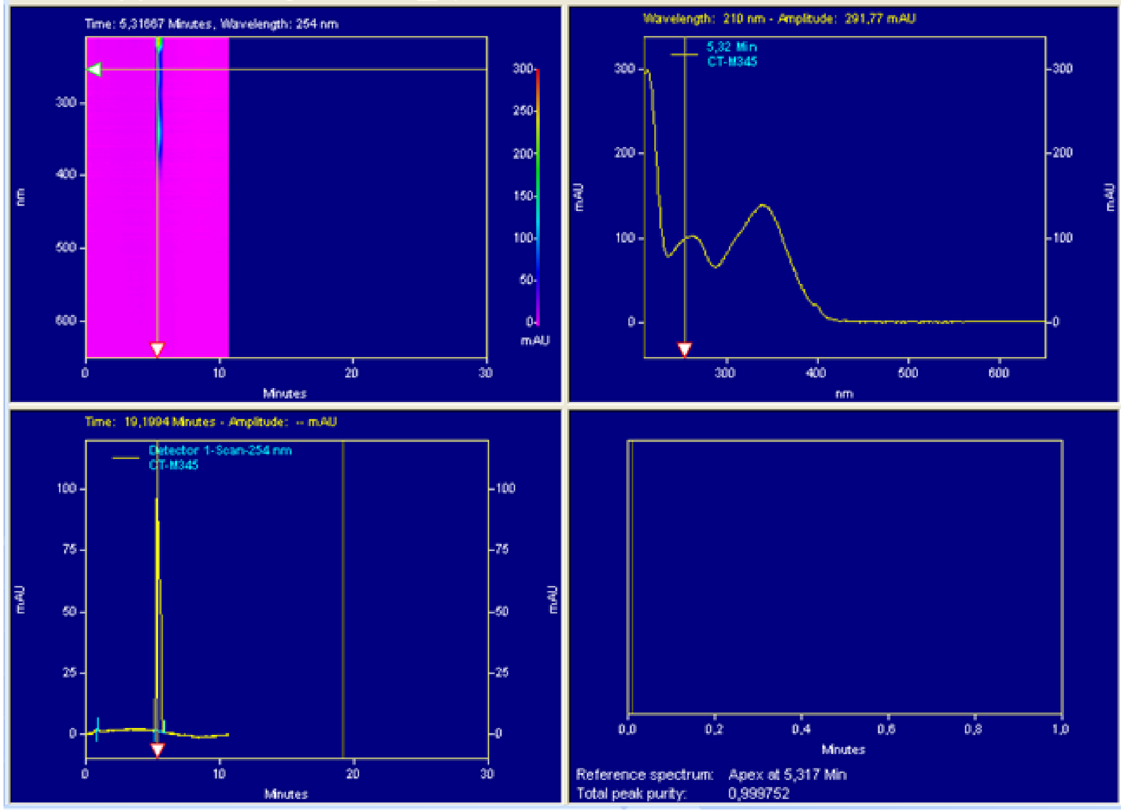

Retention Time	Area	Area %	Height	Height %
0.833	0	0.00	0	0.00
5.317	1860919	100.00	97687	100.00
Totals	1860919	100.00	97687	100.00

Figure S58. HPLC chromatograms of compound **9**.

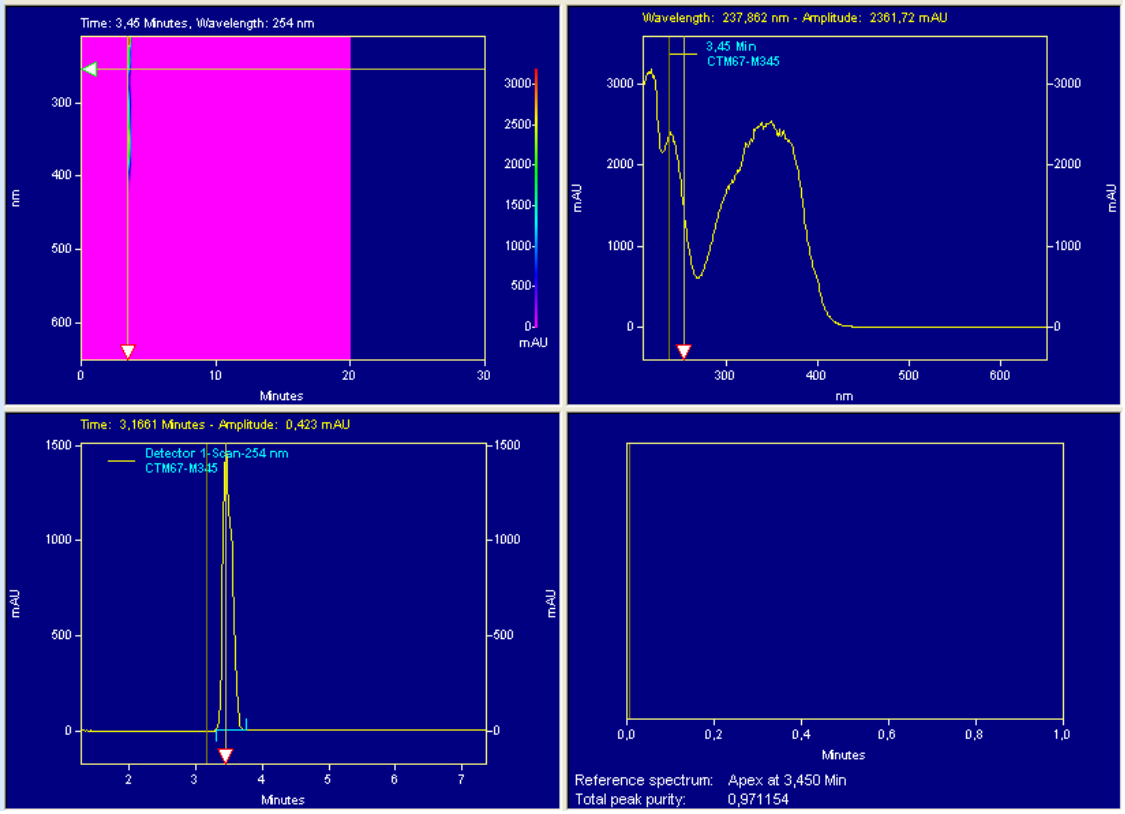

Retention Time	Area	Area %	Height	Height %
0.917	6421	0.04	1630	0.11
3.450	14388566	99.96	1468133	99.89
Totals	14394987	100.00	1469763	100.00

Figure S59. HPLC chromatograms of compound **10**.

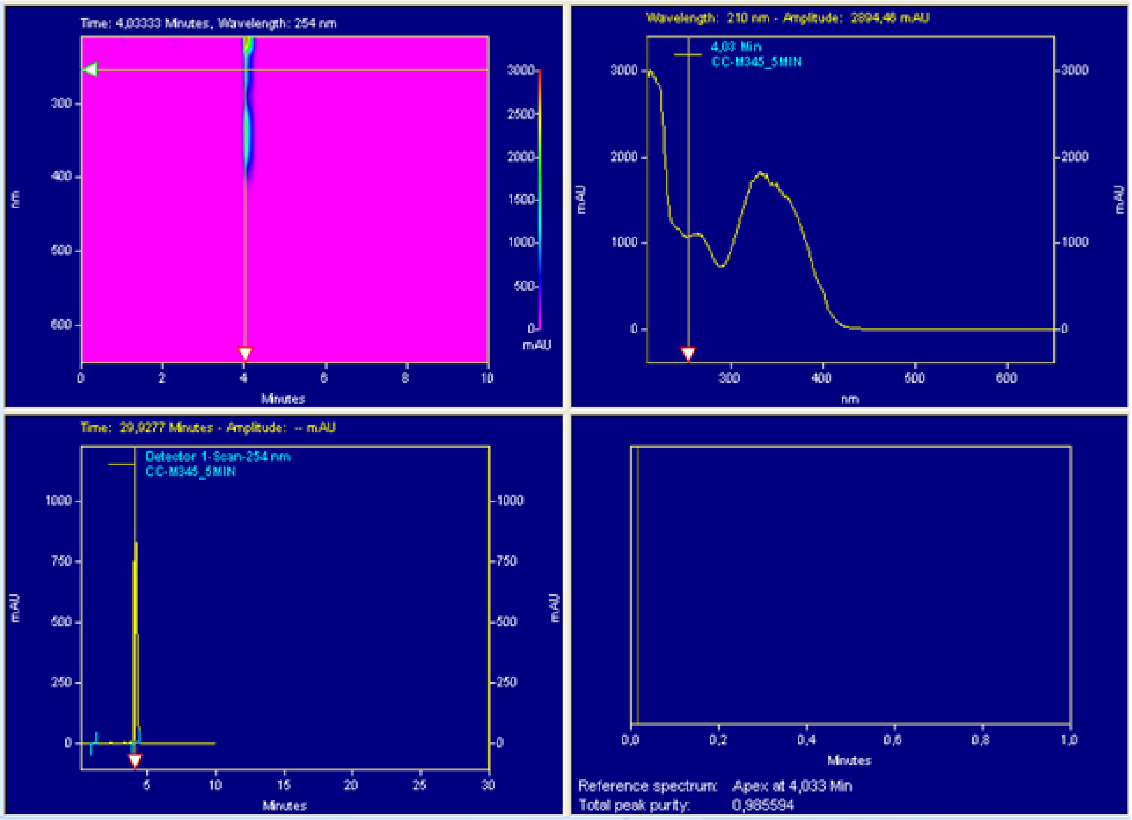

Retention Time	Area	Area %	Height	Height %
1.000	33939	0.27	2999	0.28
4.033	12632138	99.73	1069640	99.72
Totals	12666077	100.00	1072639	100.00

Figure S60. HPLC chromatograms of compound **11**.

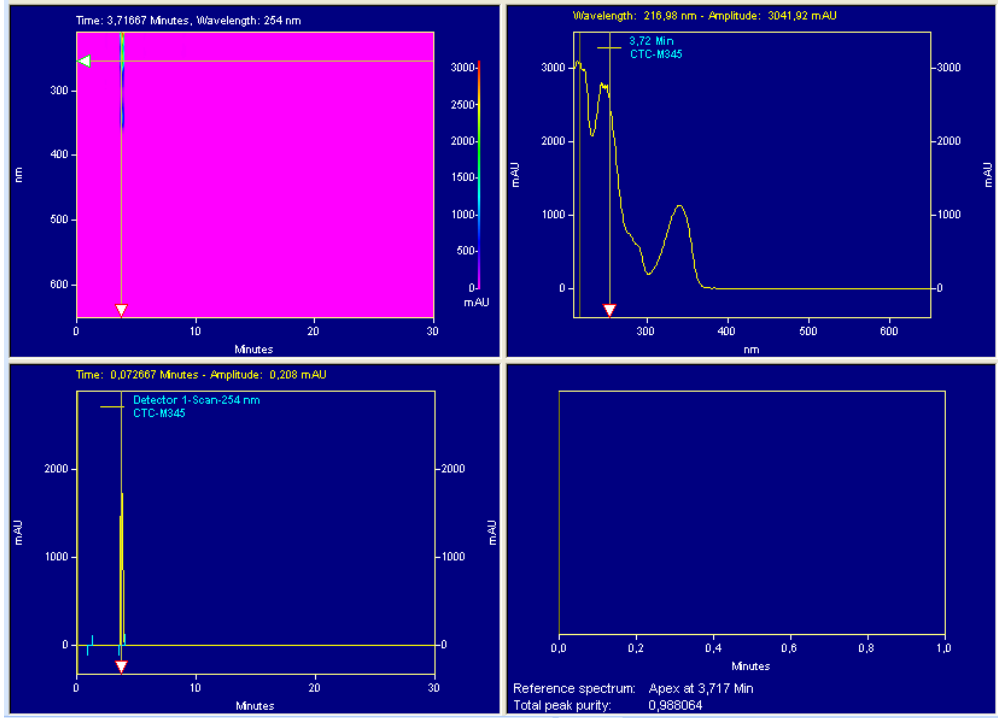

Retention Time	Area	Area %	Height	Height %
1.050	50627	0.20	2061	0.08
3.717	24957627	99.80	2561260	99.92
Totals	25008254	100.00	2563321	100.00

Figure S61. HPLC chromatograms of compound **12**.

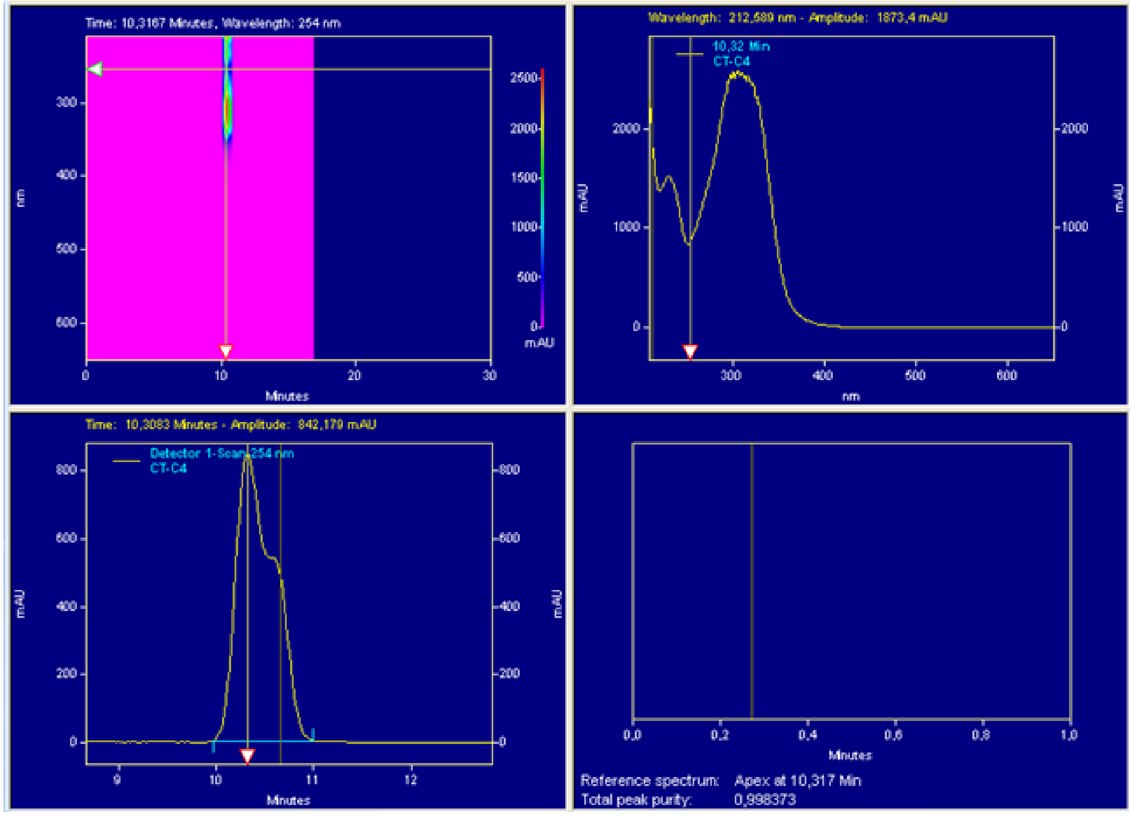

Retention Time	Area	Area %	Height	Height %
0.900	3256	0.01	1028	0.12
10.317	23084443	99.99	845811	99.88
Totals	23087699	100.00	846839	100.00

Figure S62. HPLC chromatograms of compound **13**.

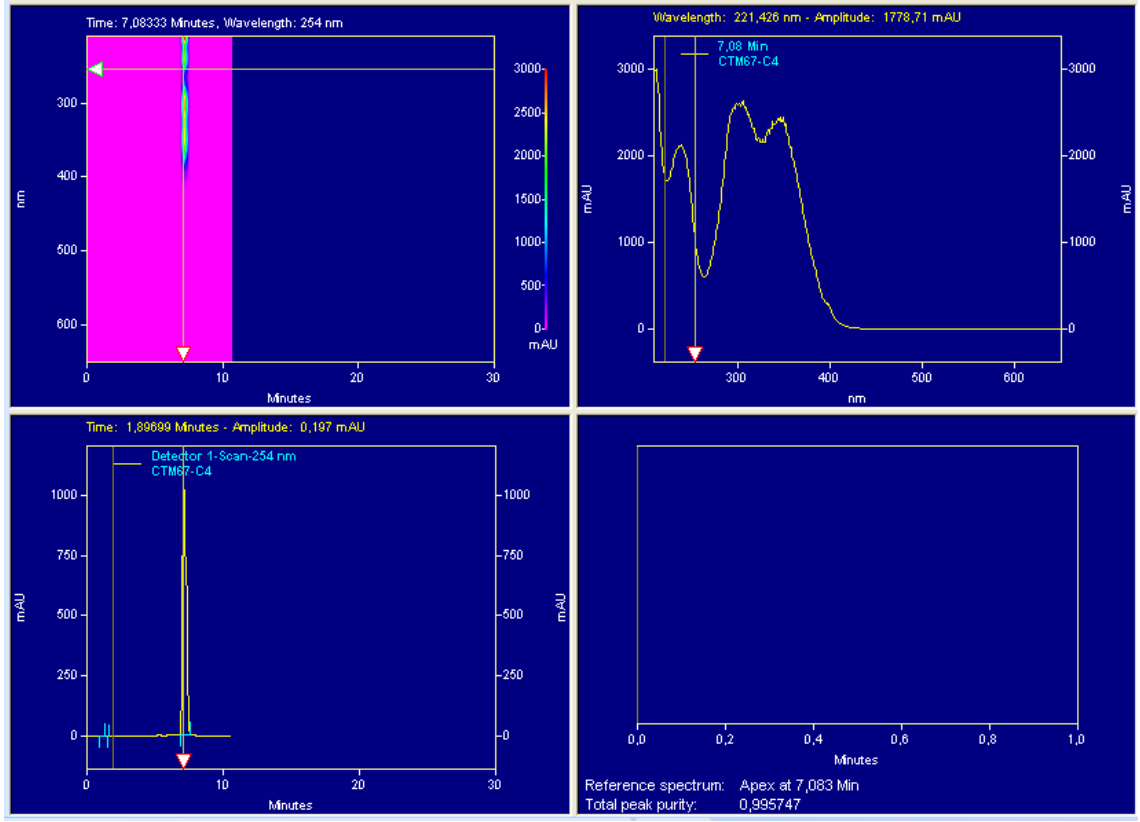

Retention Time	Area	Area %	Height	Height %
0.950	34353	0.17	1204	0.11
1.550	679	0.00	304	0.03
7.083	20365893	99.83	1063338	99.86
Totals	20400925	100.00	1064846	100.00

Figure S63. HPLC chromatograms of compound **14**.

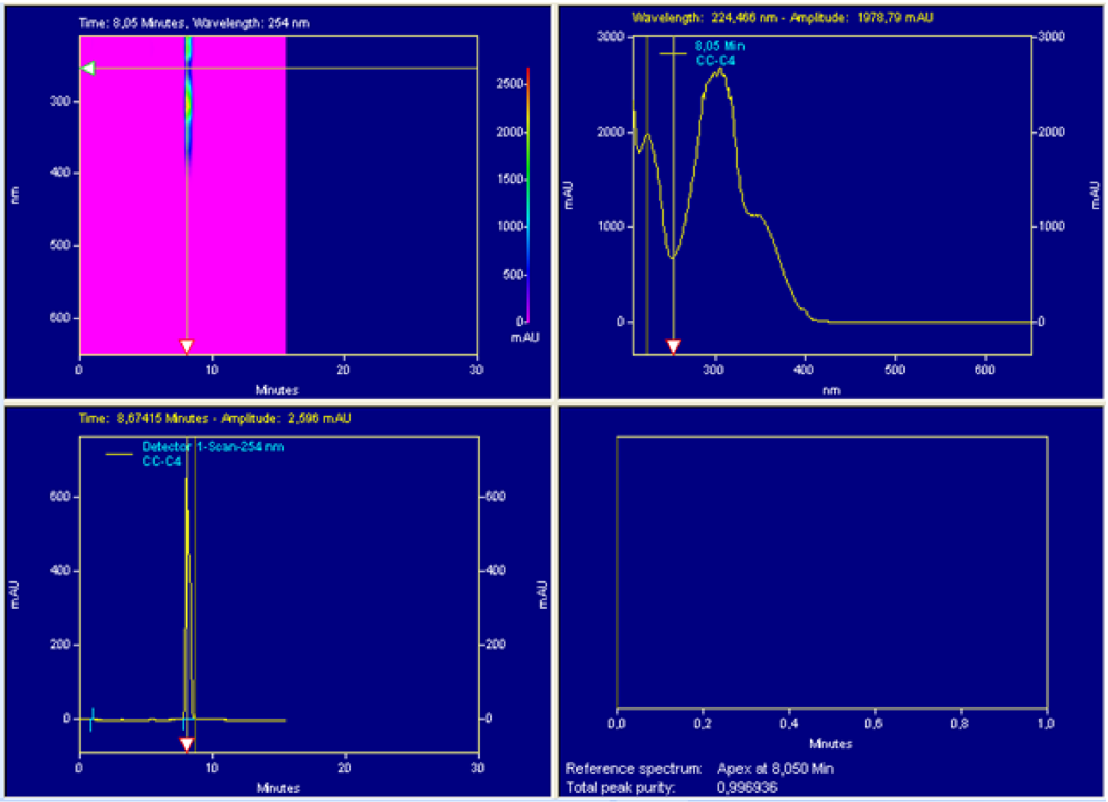

Retention Time	Area	Area %	Height	Height %
0.900	5145	0.04	1313	0.19
8.050	14415785	99.96	673782	99.81
Totals	14420930	100.00	675095	100.00

Figure S64. HPLC chromatograms of compound **15**.

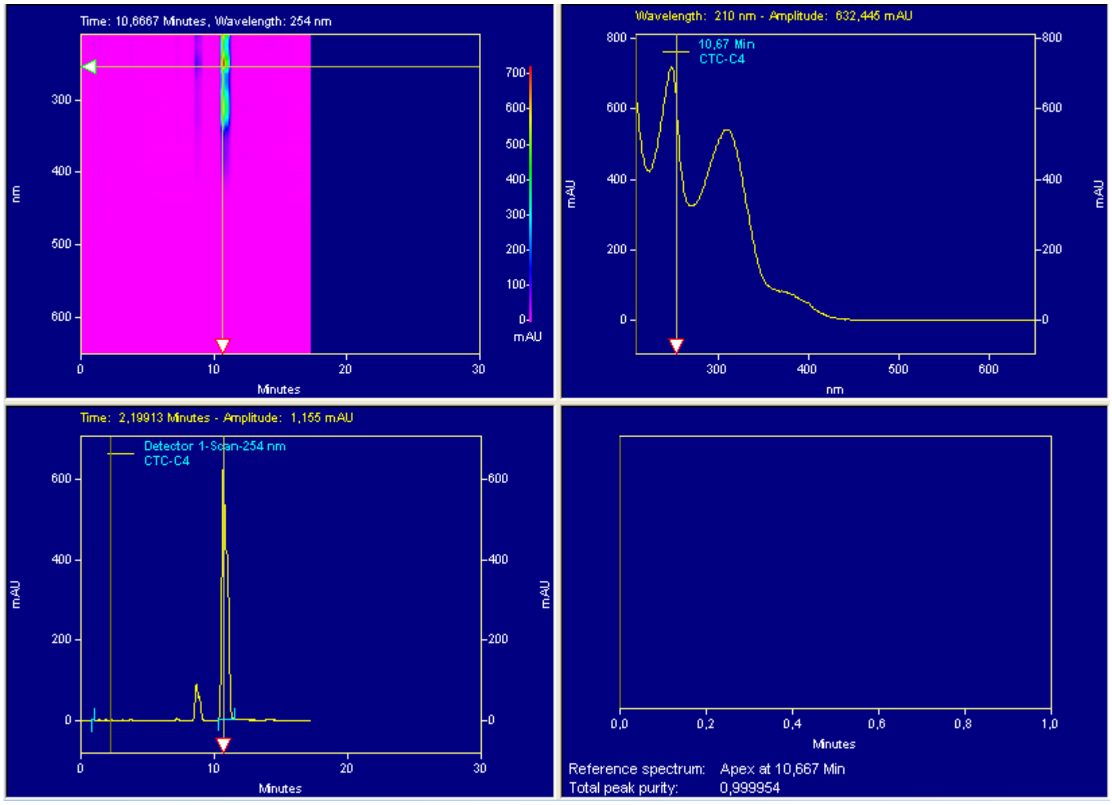

Retention Time	Area	Area %	Height	Height %
0.883	4713	0.03	857	0.14
10.667	17800466	99.97	625218	99.86
Totals	17805179	100.00	626075	100.00

Figure S65. HPLC chromatograms of compound **16**.

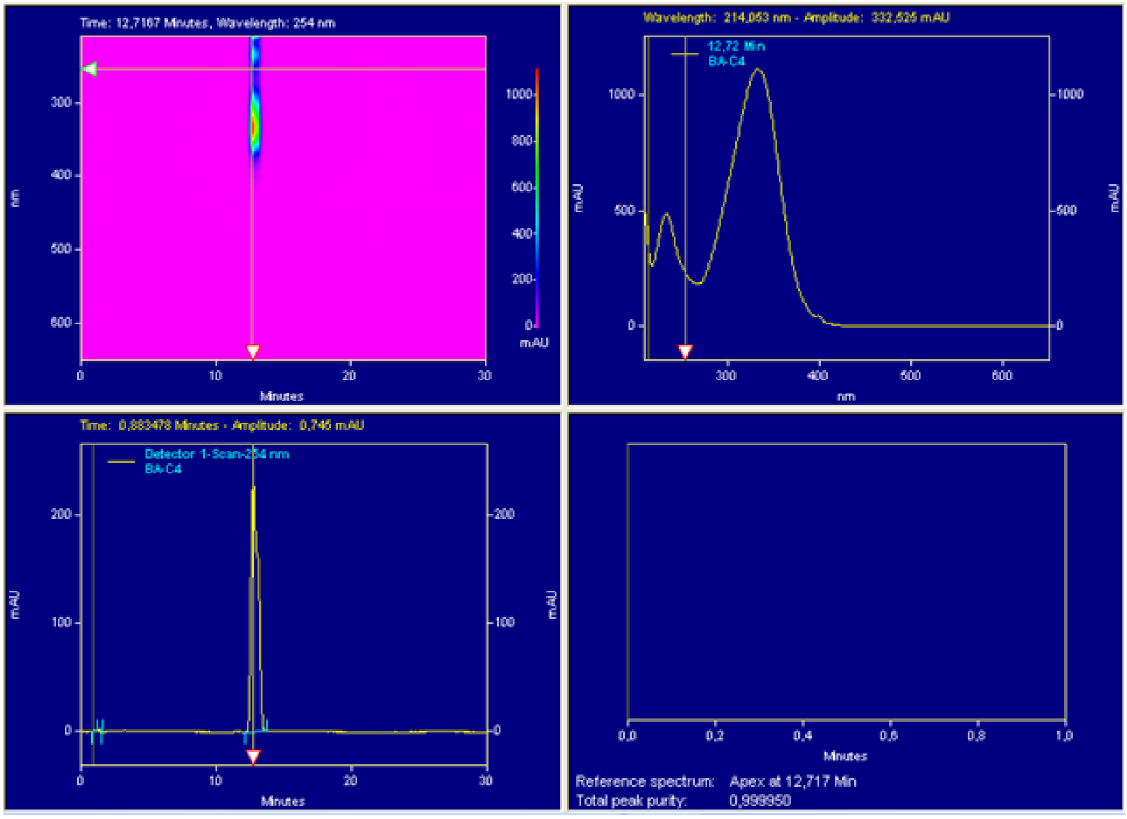

Retention Time	Area	Area %	Height	Height %
0.933	12287	0.15	3091	1.29
1.550	718	0.01	246	0.10
12.717	8192194	99.84	235789	98.60
Totals	8205199	100.00	239126	100.00

Figure S66. HPLC chromatograms of compound **17**.

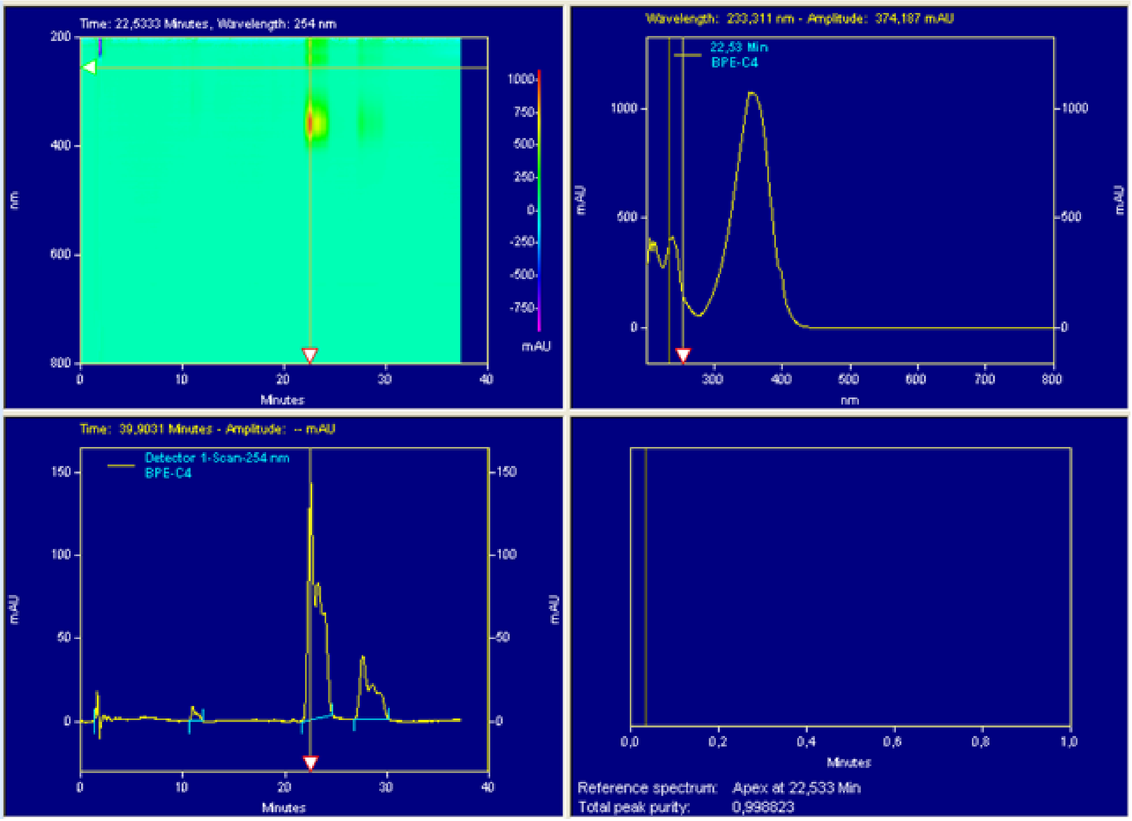

Retention Time	Area	Area %	Height	Height %
1.283	102	0.00	102	0.05
10.967	307343	2.19	8638	4.53
22..533	13725459	97.81	182109	95.42
Totals	14032904	100.00	190849	100.00

Figure S67. HPLC chromatograms of compound **18**.

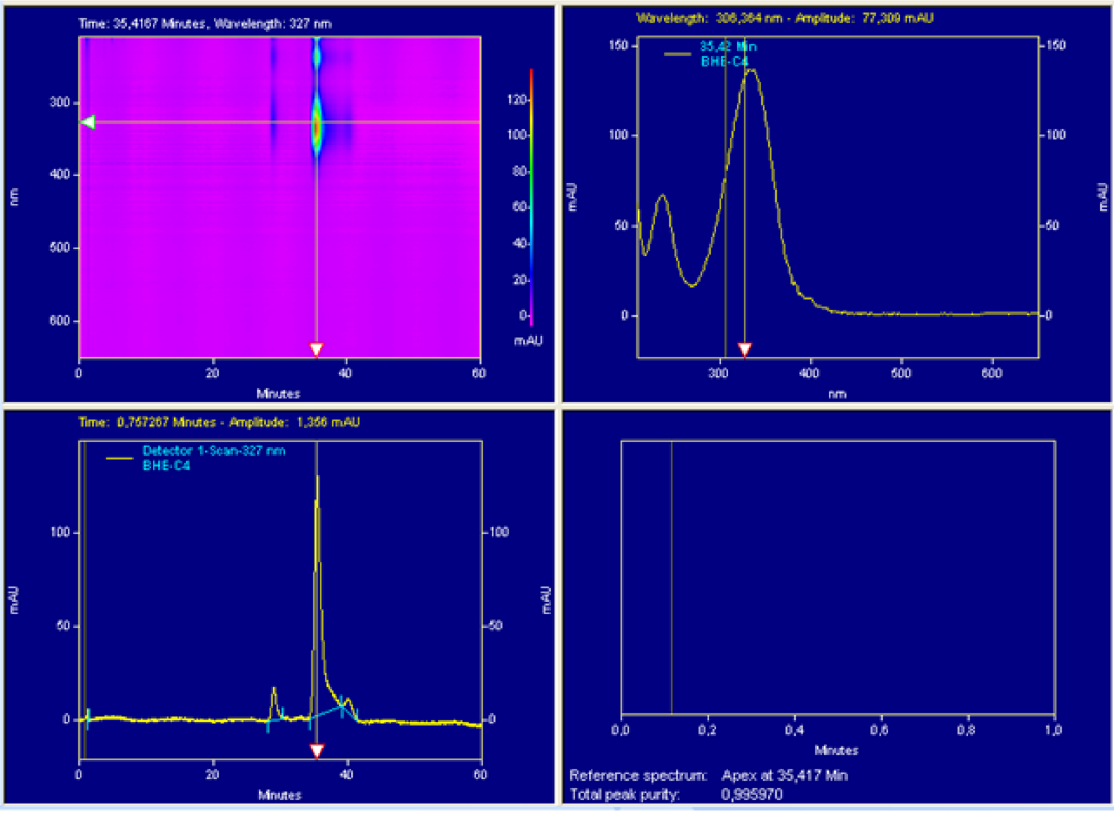

Retention Time	Area	Area %	Height	Height %
28.967	223021	2.02	4383.3	2.75
35.417	10677707	96.87	153533.4	96.14
40.050	122834	1.11	1777.3	1.11
Totals	11023562	100.00	159694	100.00

Figure S68. HPLC chromatograms of compound **19**.

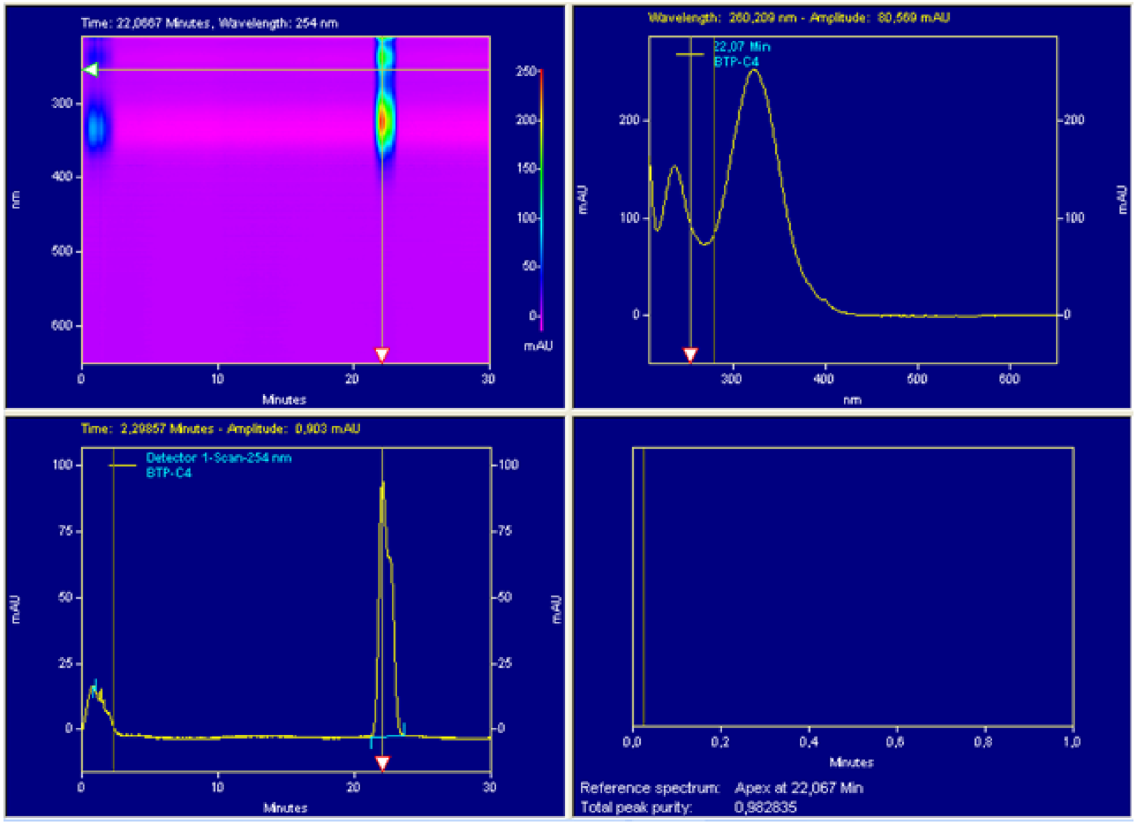

Retention Time	Area	Area %	Height	Height %
0.867	2865	0.05	694	0.71
22.067	5927590	99.95	97023	99.29
Totals	5930455	100.00	97717	100.00

Figure S69. HPLC chromatograms of compound **20**.

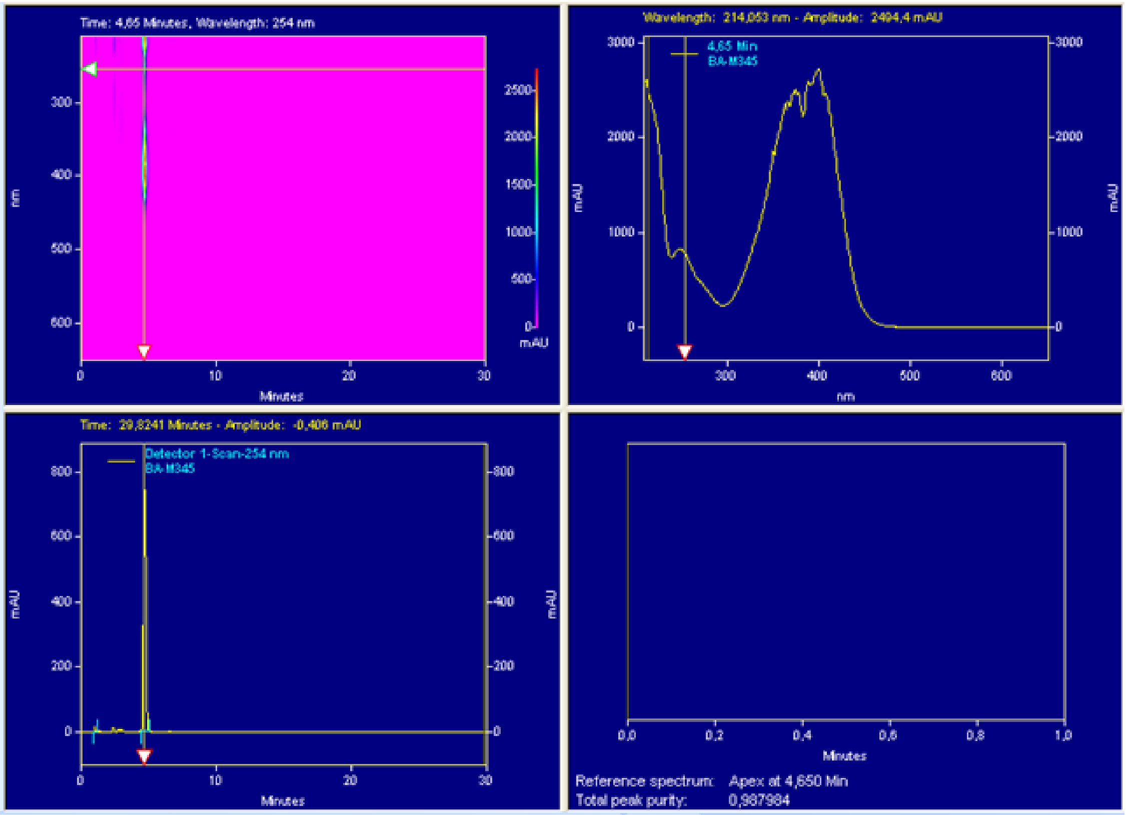

Retention Time	Area	Area %	Height	Height %
1.017	47698	0.45	17153	2.14
4.650	10511125	99.55	783993	97.86
Totals	1.017	47698	0.45	17153

Figure S70. HPLC chromatograms of compound **21**.

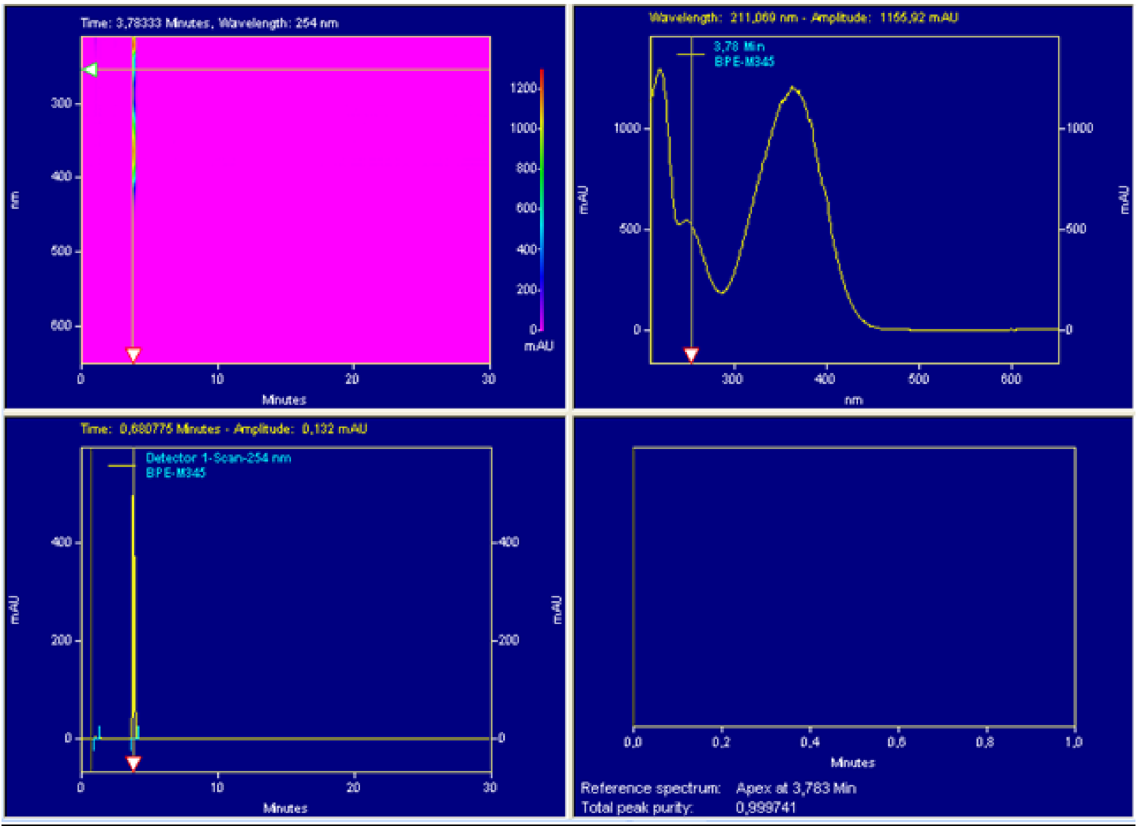

Retention Time	Area	Area %	Height	Height %
0.983	23362	0.41	6624	1.24
3.783	5716778	99.59	526936	98.76
Totals	5740140	100.00	533560	100.00

Figure S71. HPLC chromatograms of compound **22**.

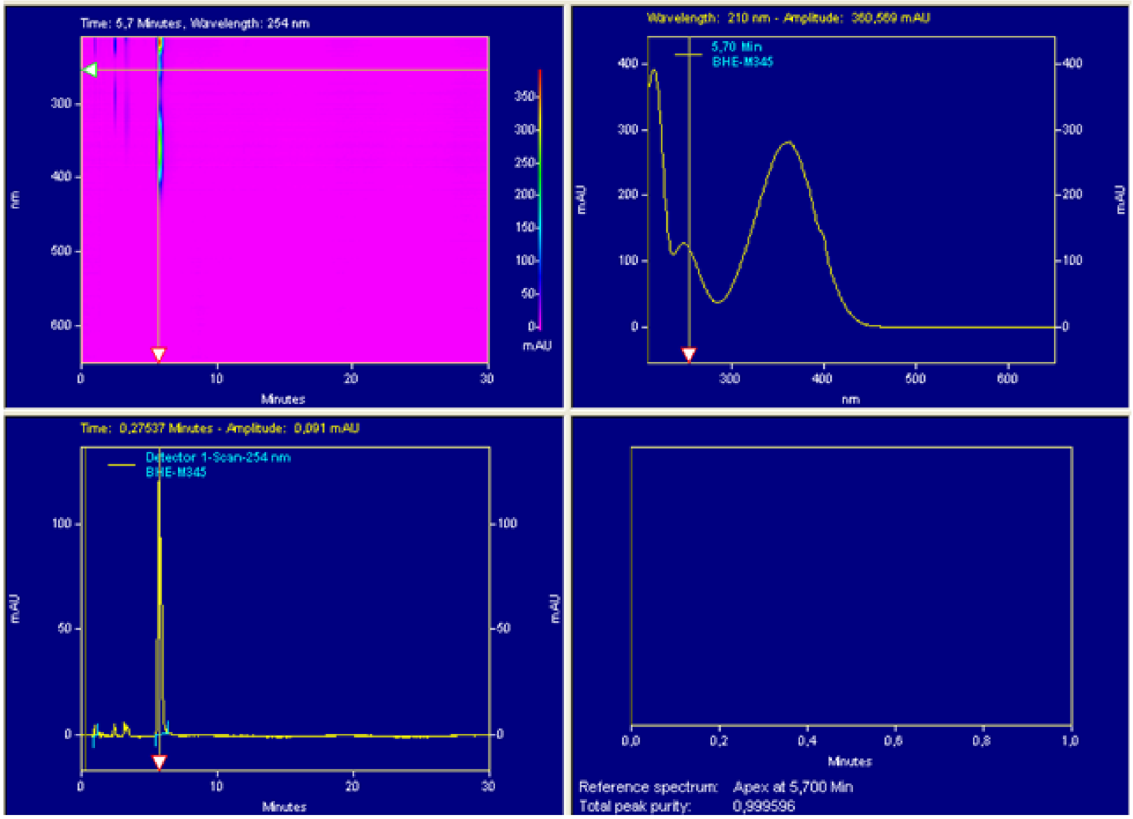

Retention Time	Area	Area %	Height	Height %
0.967	21613	1.03	5243	4.17
5.700	2069200	98.97	120581	95.83
Totals	2090813	100.00	125824	100.00

Figure S72. HPLC chromatograms of compound **23**.

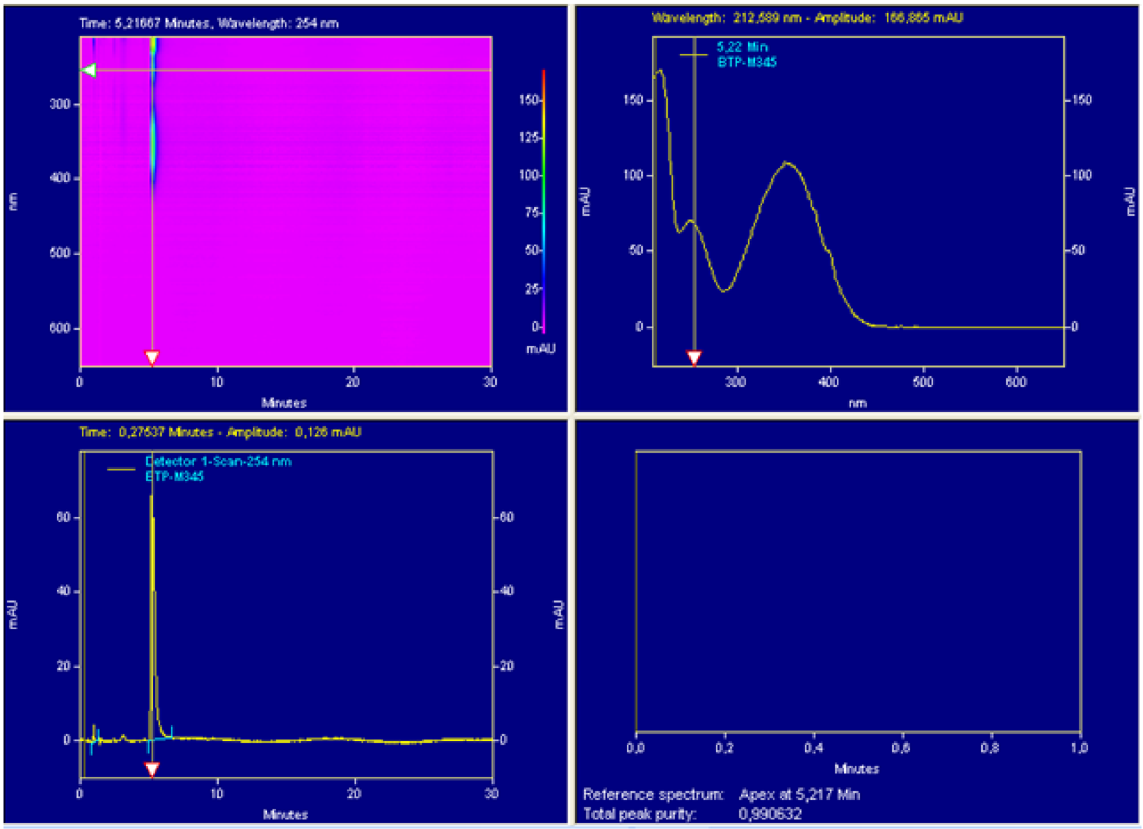

Retention Time	Area	Area %	Height	Height %
0.967	20869	1.51	4685	3.35
5.217	1362960	98.49	69143	96.65
Totals	1383829	100.00	73828	100.00

Figure S73. HPLC chromatograms of compound **24**.

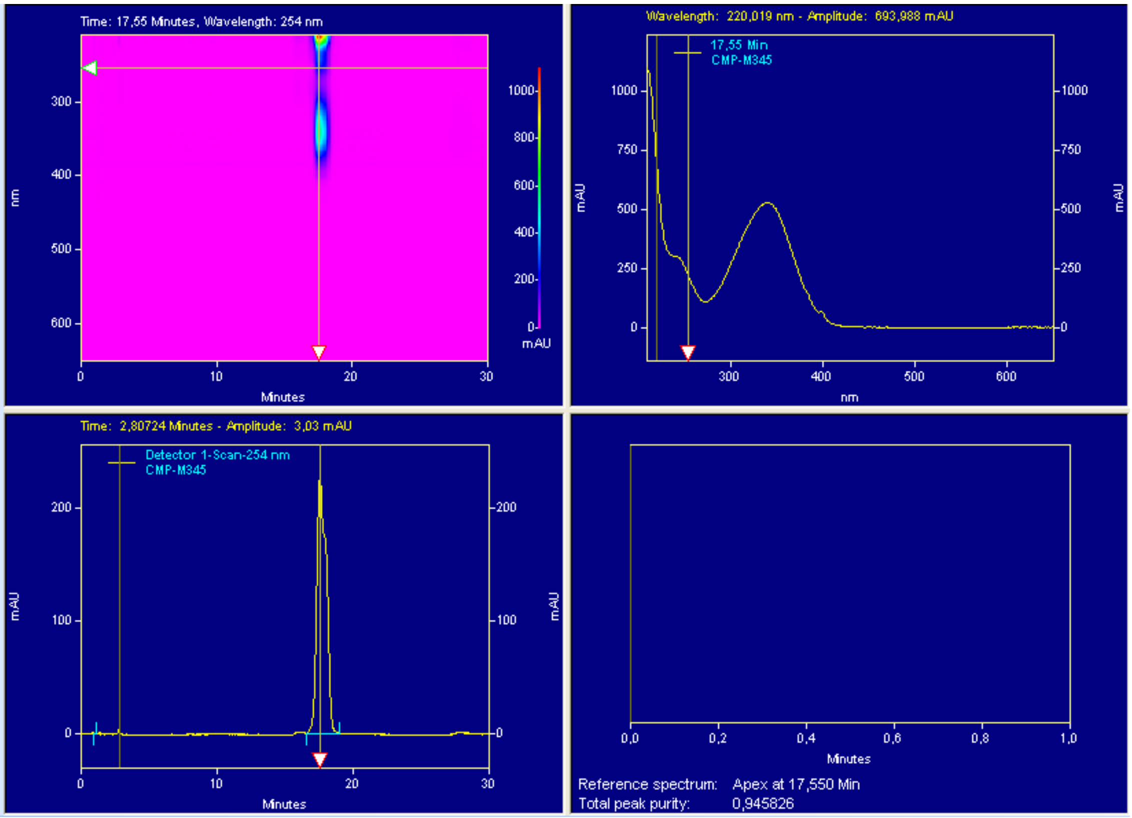

Retention Time	Area	Area %	Height	Height %
0.900	5302	0.05	1228	0.54
17.550	11328259	99.95	226418	99.46
Totals	11333561	100.00	227646	100.00

Figure S74. HPLC chromatograms of compound **26**.

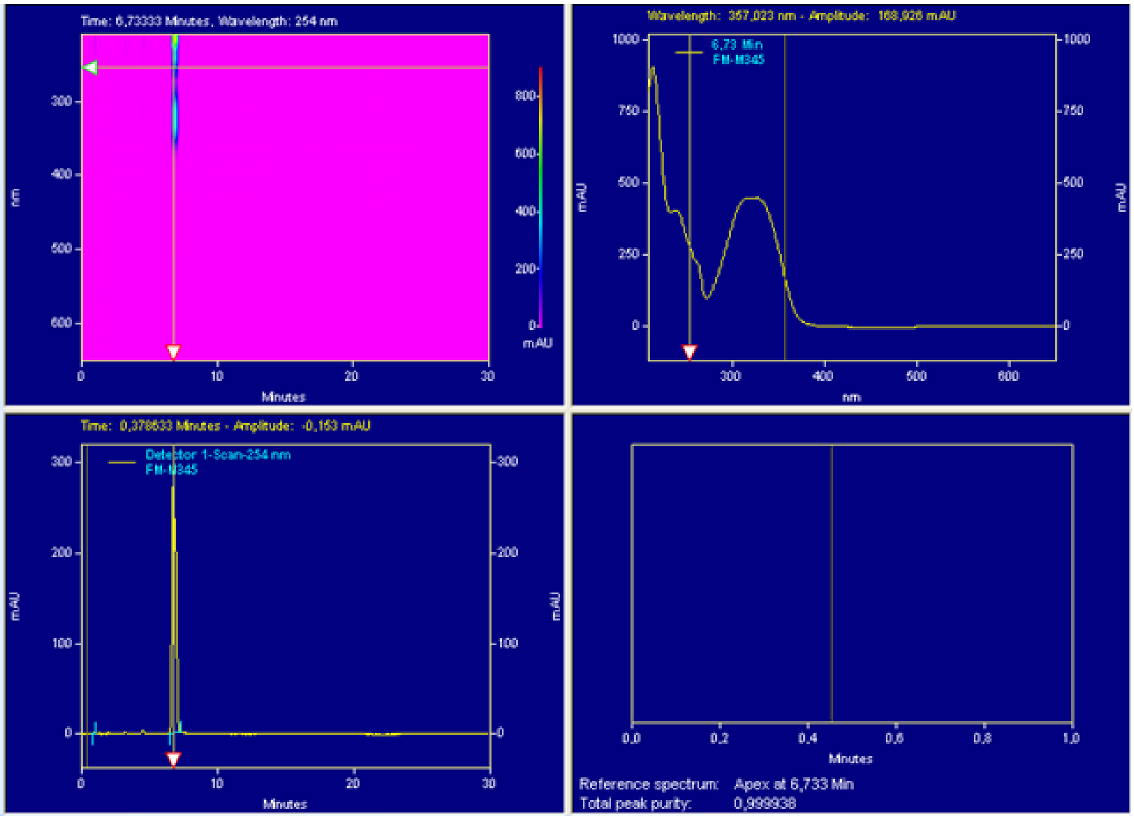

Retention Time	Area	Area %	Height	Height %
0.917	10451	0.19	2253	0.79
6.733	5459800	99.81	283109	99.21
Totals	5470251	100.00	285362	100.00

Figure S75. HPLC chromatograms of compound **27**.

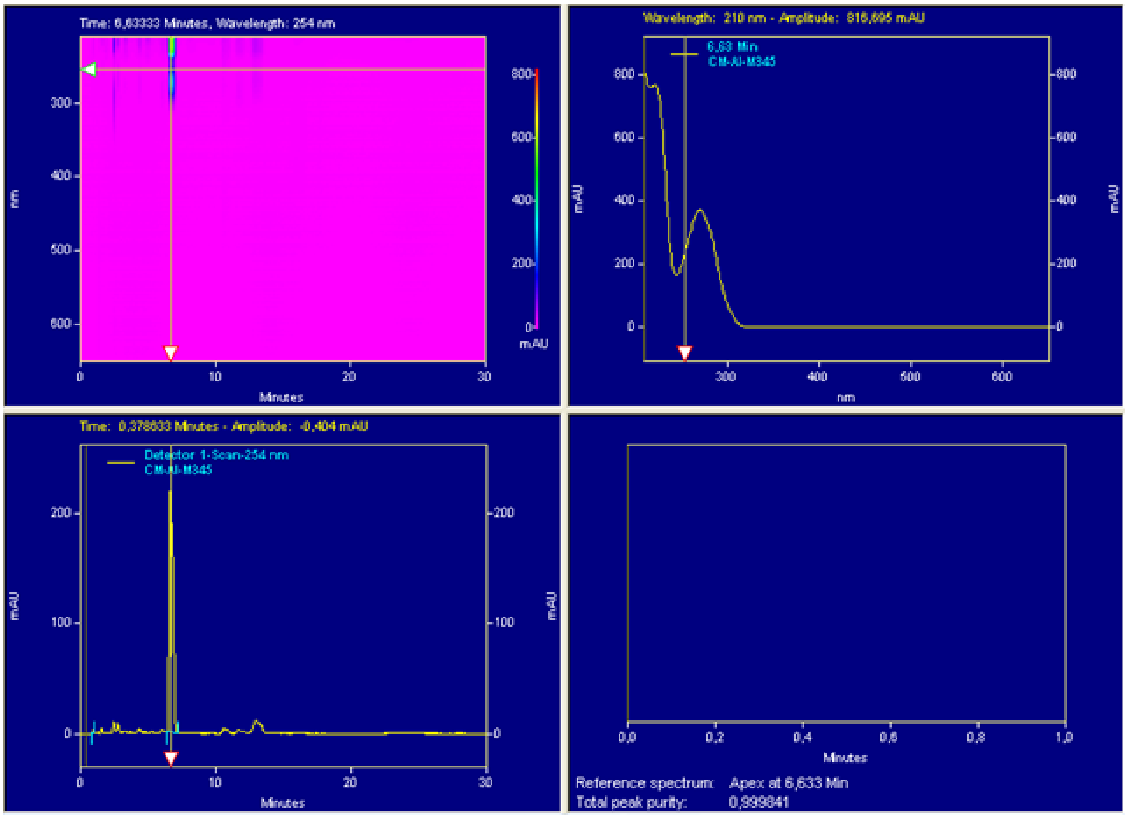

Retention Time	Area	Area %	Height	Height %
0.883	7342	0.17	1902	0.82
6.633	4339688	99.83	231323	99.18
Totals	4347030	100.00	233225	100.00

Figure S76. HPLC chromatograms of compound **28**.

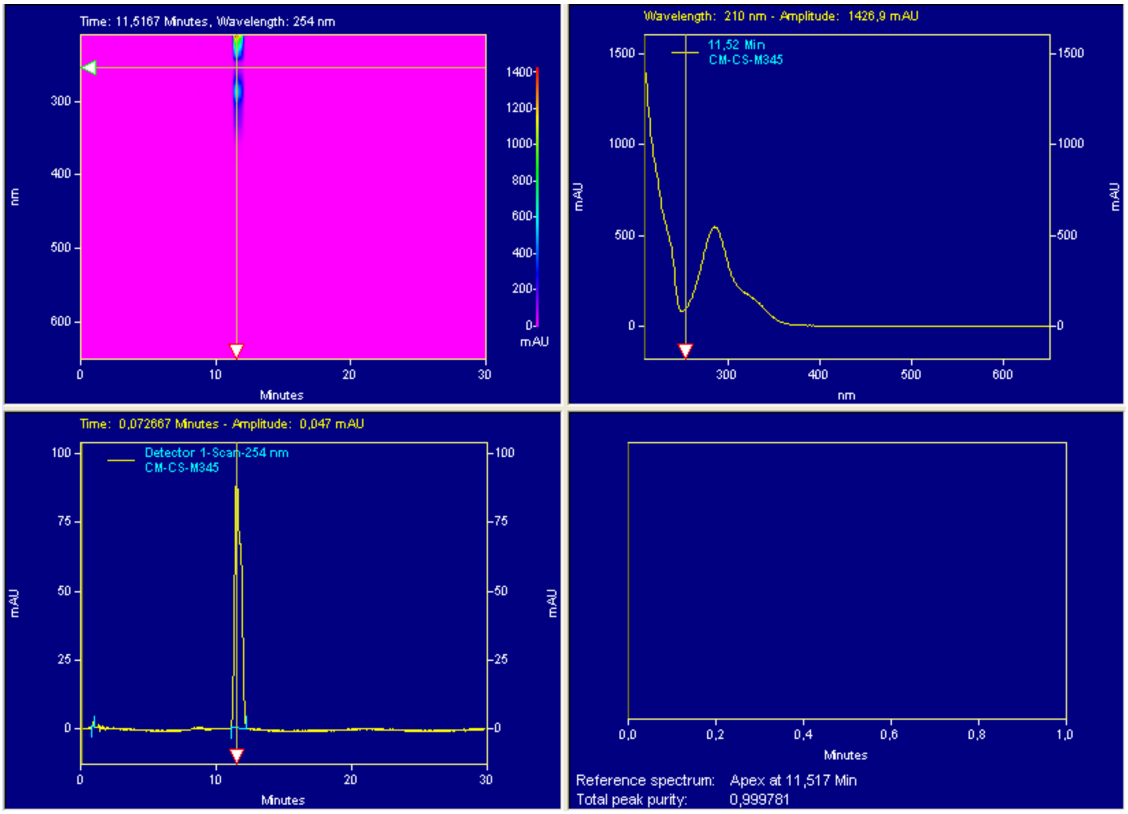

Retention Time	Area	Area %	Height	Height %
0.883	5257	0.18	1392	1.49
11.517	2959126	99.82	91793	98.51
Totals	2964383	100.00	93185	100.00

Figure S77. HPLC chromatograms of compound **29**.

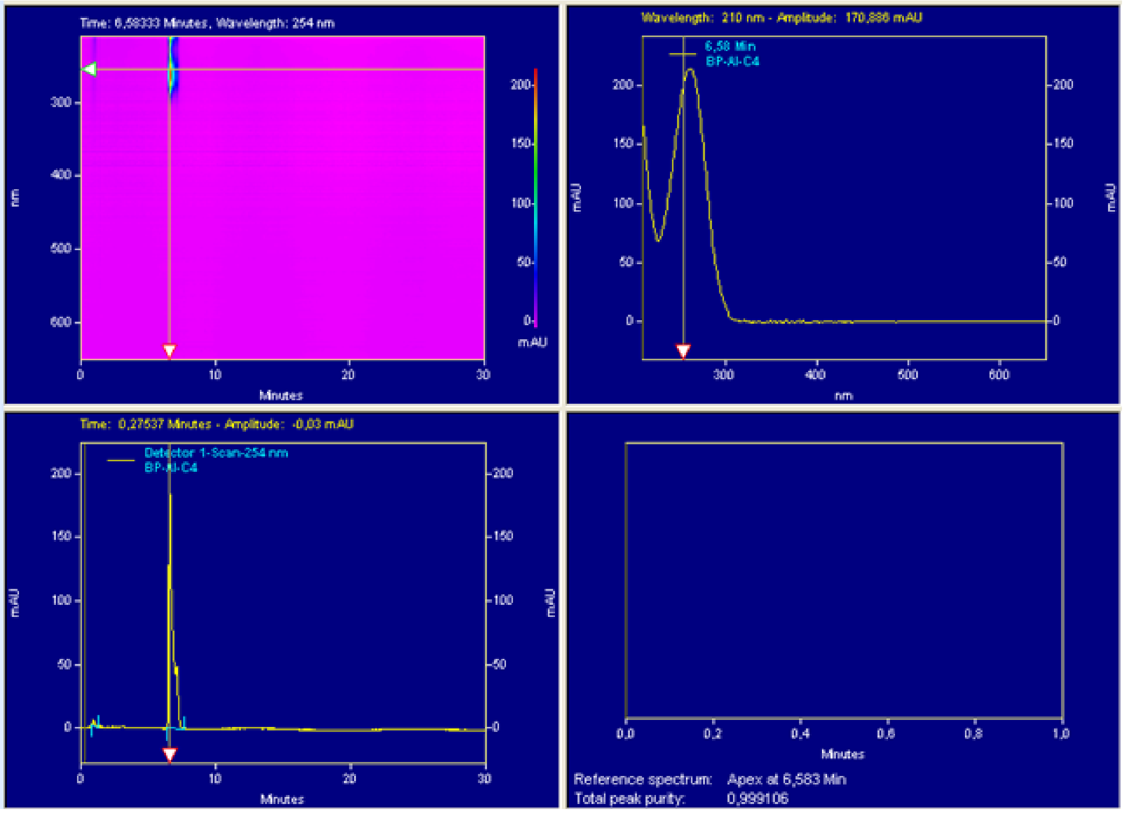

Retention Time	Area	Area %	Height	Height %
0.917	44732	1.07	4789	2.35
6.583	4146238	98.93	199051	97.65
Totals	4190970	100.00	203840	100.00

Figure S78. HPLC chromatograms of compound **30**.

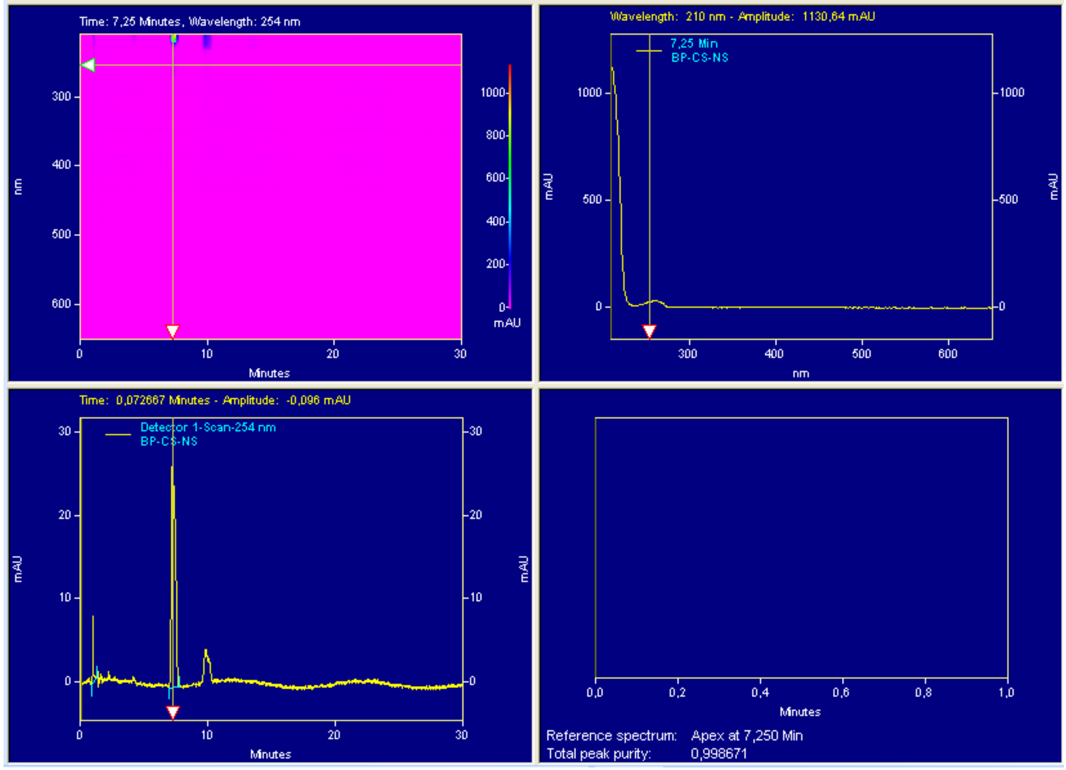

Retention Time	Area	Area %	Height	Height %
1.000	23647	3.97	8047	1.85
7.250	572030	96.03	28773	98.15
Totals	595677	100.00	36820	100.00

Figure S79. HPLC chromatograms of compound **31**.

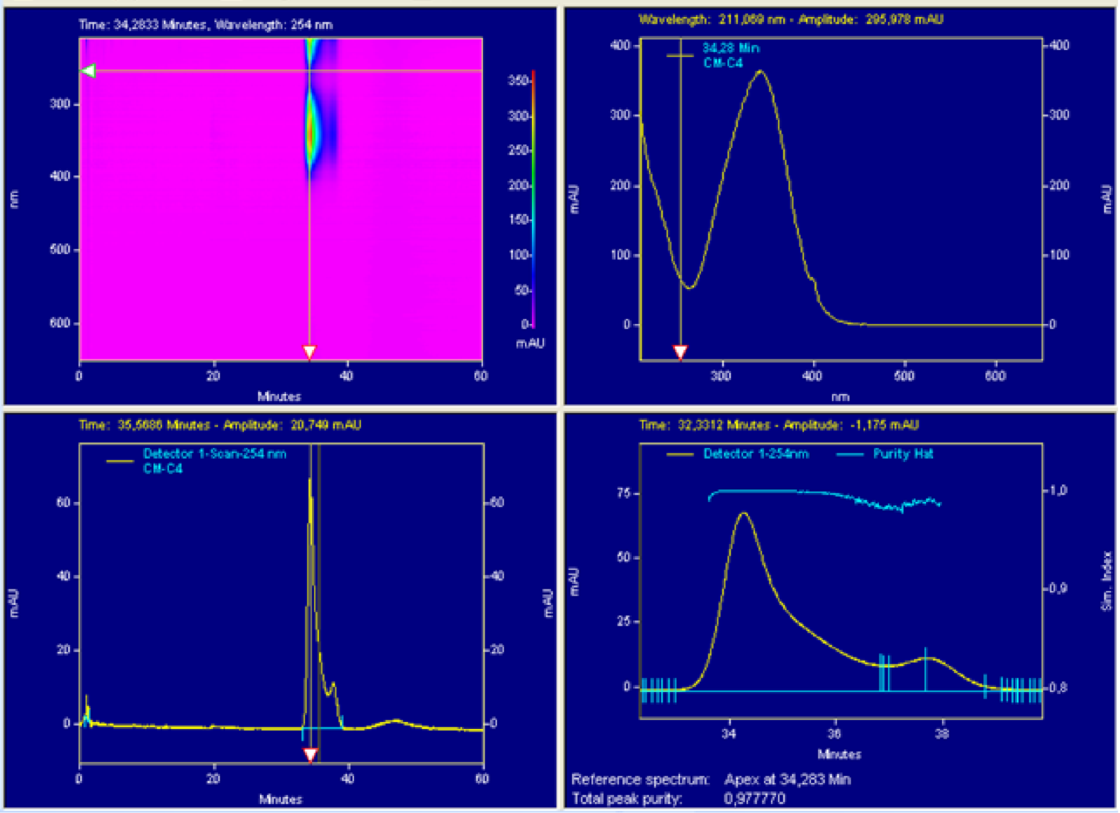

Retention Time	Area	Area %	Height	Height %
0.983	56794	0.80	6033	4.07
34.283	7012429	99.20	68754	95.93
Totals	7069223	100.00	74787	100.00

Figure S80. HPLC chromatograms of compound **32**.

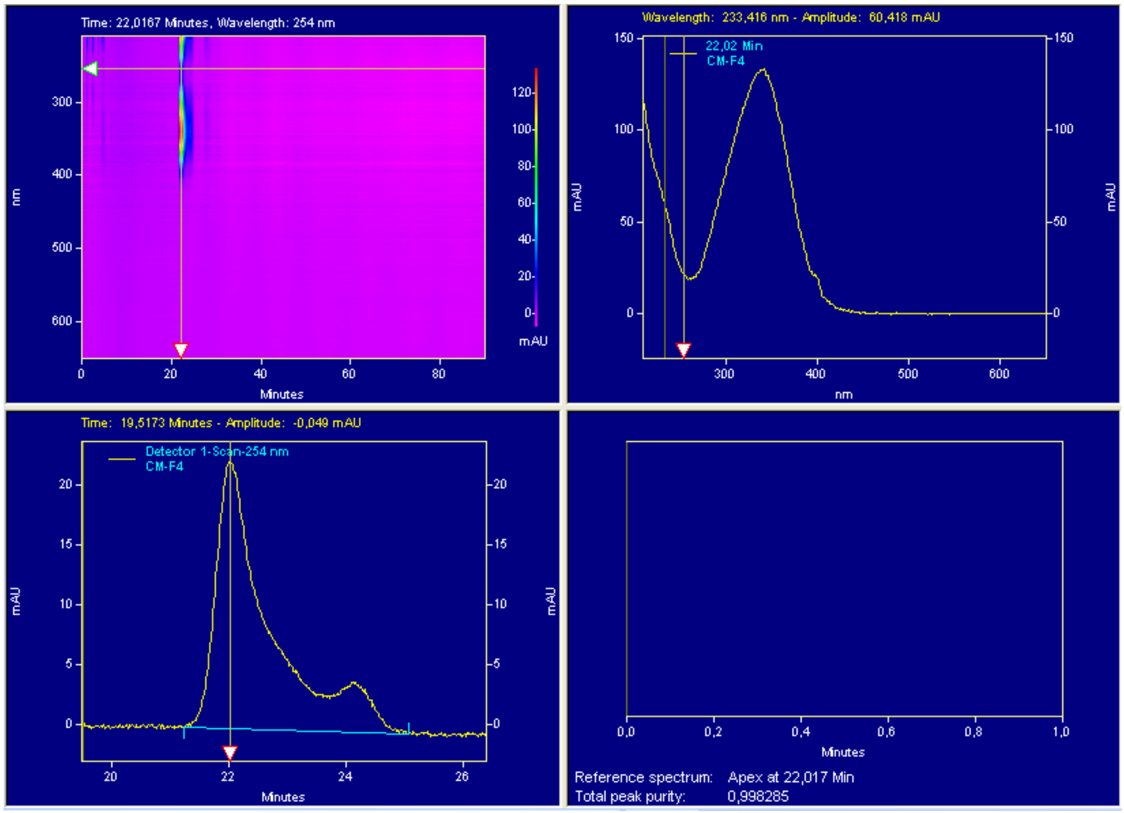

Retention Time	Area	Area %	Height	Height %
0.967	28254	1.94	4474	3.59
22.017	1429675	98.06	22490	96.41
Totals	1457929	100.00	26964	100.00

Figure S81. HPLC chromatograms of compound **33**.

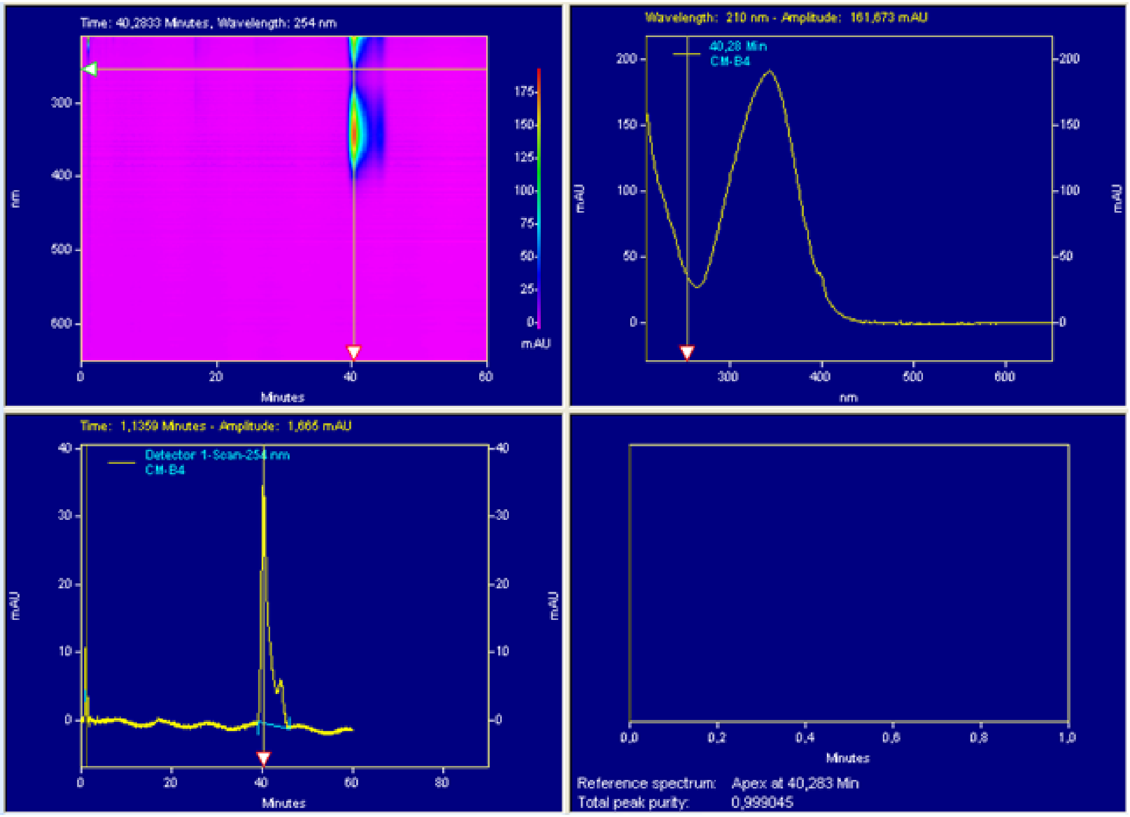

Retention Time	Area	Area %	Height	Height %
0.950	42824	0.99	7354	3.85
40.283	4297208	99.01	36286	96.15
Totals	4340032	100.00	43640	100.00

Figure S82. HPLC chromatograms of compound **34**.

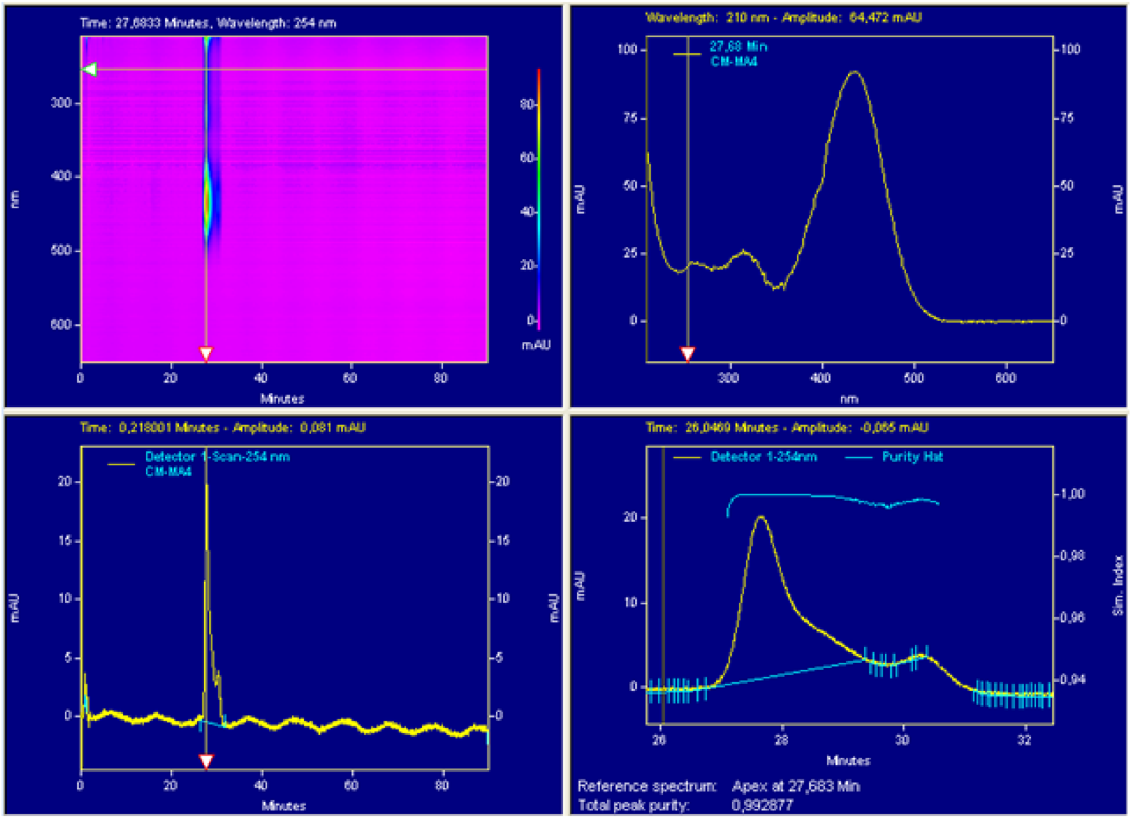

Retention Time	Area	Area %	Height	Height %
27.683	1787594	99.95	23103	98.72
89.867	903	0.05	300	1.28
Totals	1788497	100.00	23403	100.00

Figure S83. HPLC chromatograms of compound **35**.

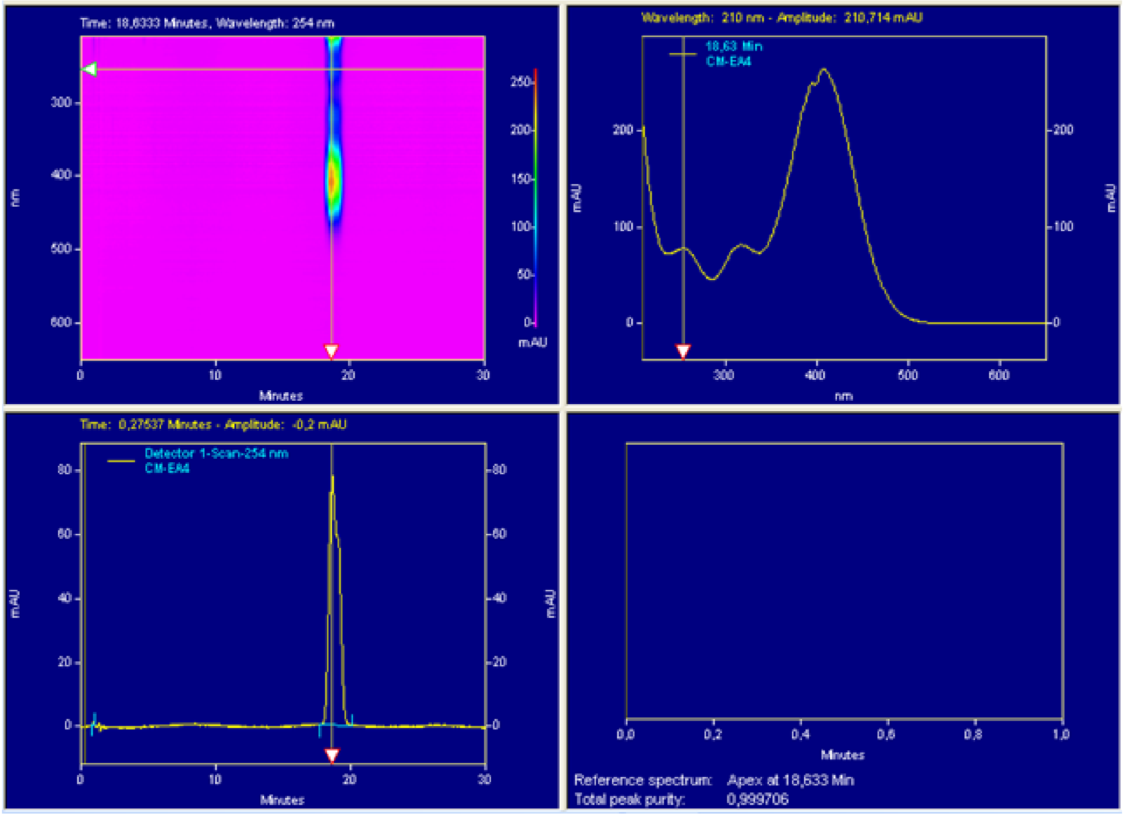

Retention Time	Area	Area %	Height	Height %
0.900	3643	0.09	1184	1.49
18.633	4076638	99.91	78183	98.51
Totals	4080281	100.00	79367	100.00

Figure S84. HPLC chromatograms of compound **36**.

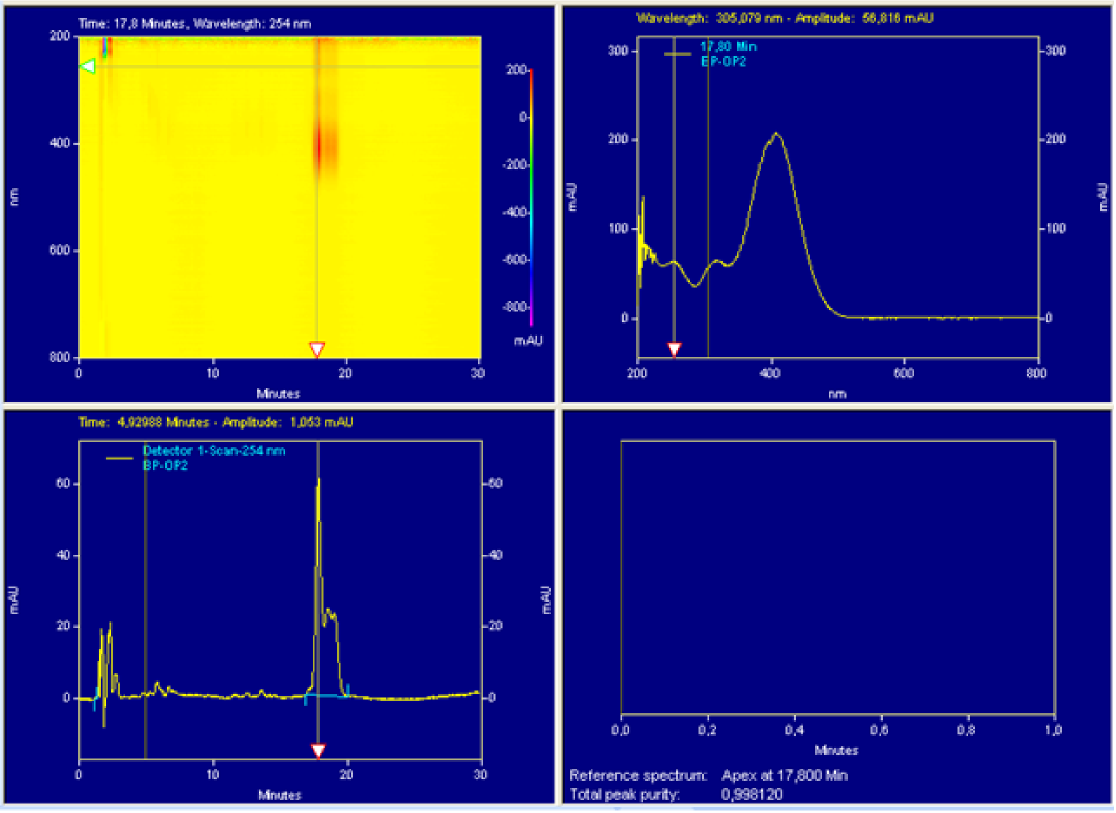

Retention Time	Area	Area %	Height	Height %
1.250	3282	0.10	498	0.79
17.800	3362216	99.90	62309	99.21
Totals	3365498	100.00	62807	100.00

Figure S85. HPLC chromatograms of compound **38**.

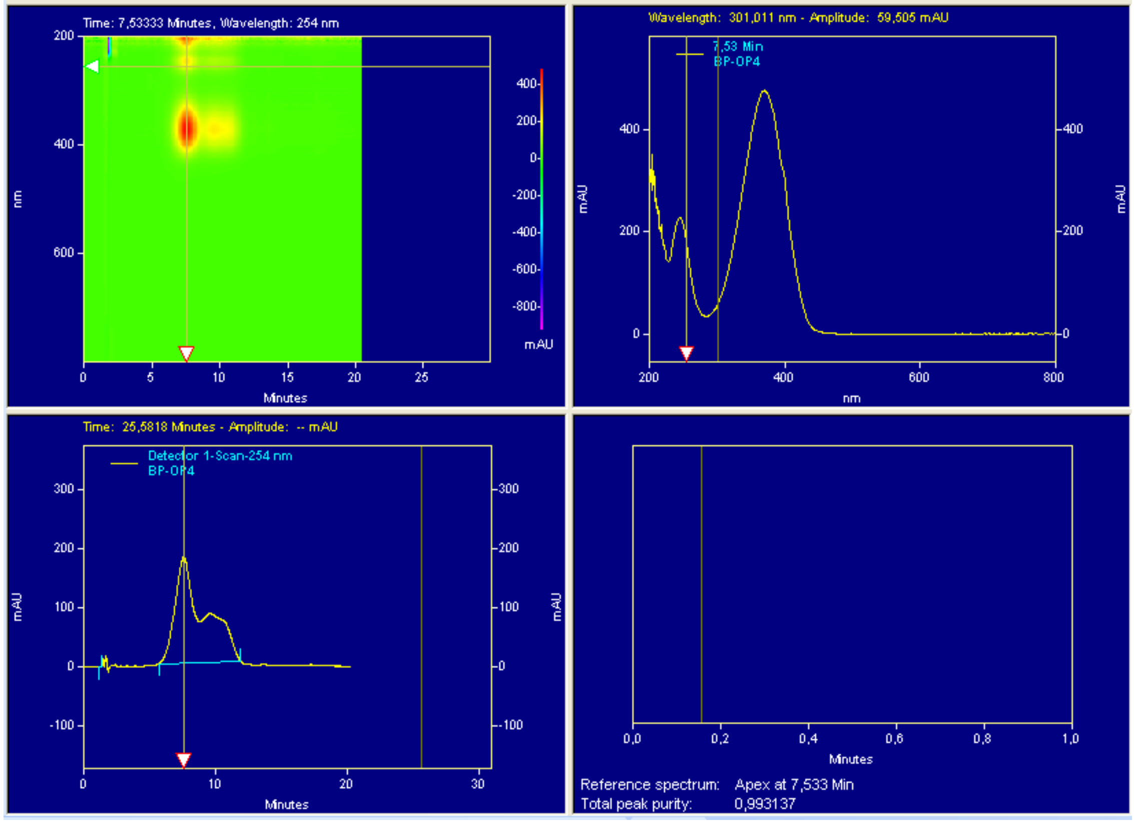

Retention Time	Area	Area %	Height	Height %
1.183	6447	0.02	800	0.45
7.533	27309498	99.98	178260	99.55
Totals	27315945	100.00	179060	100.00

Figure S86. HPLC chromatograms of compound **40**.
